# Supplementary material for: Should I stay or should I go? A retrospective propensity score-matched analysis using administrative data of hospital-at-home for older people in Scotland
Source: BMJ Open. 2019 May 9;9(5):e023350. doi: 10.1136/bmjopen-2018-023350 (PMC6527981; doi:10.1136/bmjopen-2018-023350)

Appendix 1. Calculation of admission avoidance hospital-at-home in each site

	**Site one**					
				**PERIOD**		
		**from:**	01/08/2014	**Until:**	01/01/2016	**17**
			(dd/mm/yyyy)	(dd/mm/yyyy)		Months
				Source of		
	**Number of HAH admissions (in period)**	1771		ISD IPD data (1/8/14-31/12/15)		
	**Length of HAH stay per episode (in**	5.53886	Mean	ISD IPD data (1/8/14-31/12/15)		
		0.125605	Standard error			
	**HAH bed days (period)**	9809				
**A.1.**	**Staff costs**					
No	Profession	WTEs	Gross	Summary salary	Source of	**Total**
a)	Medical staff					
1	Consultant	1.50	£151,596		Business	£227,394
2	Agency consultant	0.16	£156,926		Business	£25,651
3	Consultant	1.07	£119,710		Business	£127,767
b)	Nursing and pharmacy services					
1	Band 3 nurse	3.00	£24,790		Business	£74,369
2	Band 6 nurse	1.49	£41,425		Business	£61,740
3	Band 5 Bank nurse	0.71	£32,885		Business	£23,399
4	Band 6 Bank nurse	0.36	£38,471		Business	£13,687
5	Band 7 pharmacist	0.71	£55,491		Business	£39,484
6	Band 5 nurse	0.16	£37,036		Business	£6,054
7	Band 6 nurse	1.42	£42,342		Business	£60,303
8	Band 7 nurse	1.00	£42,444		Business	£42,444
9	Band 8a nurse	0.71	£53,126		Business	£37,801
c)	Allied health professions					
1	Band 6 occupational therapist	2.59	£35,489		Business	£91,793
2	Band 6 physiotherapist	1.16	£46,585		Business	£54,200
3	Band 4 assistant practitioners for rehab	3.59	£24,660		Business	£88,444
4	Band 6 physiotherapy	0.71	£46,848		Business	£33,334
d)	Administration, ICT and management					
1	Band 2 admin/clerical	0.30	£19,346		Business	£5,804
2	Band 3 admin/clerical	1.00	£23,948		Business	£23,948
3	Band 3 admin/clerical	0.71	£21,353		Business	£15,193
e)	Support services staff					
1						£0
	**Total**					£1,052,80
**A.2.**	**Trainning costs**<br>Note: the time to attend a course should be included in					
No.	Profession	Number of	Cost per	Summary costs	Source of	**Total**
1	Acute urgent care course	20	£250			£5,000
2	Prescribing course	3	£310			£930
	**Total**					£5,930
**A.3.**	**Transport costs**					
No.	Cost item	Number of	Cost per item	Summary costs	Source of	**Total**
1	Travel and subsistence			£37,918	Business	£37,918
	**Total**					£37,918
**A.4.**	**Information and communication costs**<br>**(e.g. brochures and leaflets for patients and their**					
No.	Cost item	Number of	Cost per item	Summary costs	Source of	**Total**
1						£0
	**Total**					£0
**A.5.**	**Clinical materials/equipment and**					
No.	Cost item	Number of	Cost per item	Summary costs	Source of	**Total**
1	Instruments and sundries			£2,867	Business	£2,867

2	Equipment repairs clinical			£585	Business	£585
3	Surgical appliances			£104	Business	£104
4	Drugs			£1,693	Business	£1,693
5	Equipment purchase clinical			£298	Business	£298
	**Total**					£5,546

A.6. Support services supplies

No.	Cost item	Number of	Cost per item	Summary costs	Source of	**Total**
1	Catering			£177	Business	£177
2	Uniforms			£552	Business	£552
3	Printing and stationery			£737	Business	£737
4	Dressings			£473	Business	£473
5	general services			£16	Business	£16
	**Total**					£1,955

A.7. Labs and diagnostics

No.	Cost item	Number of	Cost per item	Summary costs	Source of	**Total**
1	Diagnostic supplies			£559	Business	£559
						£559

A.8. Overhead costs

No.	Cost item	Number of	Cost per item	Summary costs	Source of	**Total**
1	Telephone			£3,794	Business	£3,794
2	Building			£119	Business	£119
3	Miscellaneous			£34	Business	£34
	**Total**					£3,947

A.9. Other costs

No.	Cost item	Number of	Cost per item	Summary costs	Source of	**Total**
1	Equipment purchase non medical			£3,354	Business	£3,354
2	postage			£772	Business	£772
	**Total**					£4,126

A.10. Additional costs

No.	Cost item	Number of	Cost per item	Summary costs	Source of	**Total**
1						£0
	**Total**					£0

**TOTAL**	**£1,112,79**
**Unit cost of HAH admission**	**£628.34**
**Unit cost of HAH bed day**	**£113.44**

Site two

			**PERIOD**			
**from:**	01/01/2015		**Until:**	01/01/2017		**24**
	(dd/mm/yyyy)		(dd/mm/yyyy)			Months

			Source of	
**Number of HAH admissions (in period)**	1547		ISD IPD data	
**Length of HAH stay per episode (in**	7.35	Mean	ISD IPD data	
	0.14	Standard error		
**HAH bed days (period)**	11376			

**A.1.**	**Staff costs**					
No	Profession	WTEs	Gross	Summary salary	Source of	**Total**
a)	Medical staff					
1	Senior medical			£82,099	Business	£82,099
2	Professional fees and charges			£124,391	Business	£124,391
b)	Nursing and pharmacy services					
1	Nursing & Midwifery-trained			£2,904,576	Business	£2,904,57
2	Nursing & Midwifery-untrained			£627,532	Business	£627,532
3	Pharmacists			£43,715	Business	£43,715
4	Pharmacy Technicians			£14,471	Business	£14,471
c)	Allied health professions					
1					Business	£0
d)	Administration, ICT and management					
1	Admin Clerical			£126,018	Business	£126,018
e)	Support services staff					
1						£0
	**Total**					£3,922,80

**A.2.**	**Trainning costs**<br>Note: the time to attend a course should be included in					
No.	Profession	Number of	Cost per	Summary costs	Source of	**Total**
1	Trainning costs			£1,512		£1,512
	**Total**					£1,512

**A.3.**	**Transport costs**					
No.	Cost item	Number of	Cost per item	Summary costs	Source of	**Total**
1	Transport			£25,711	Business	£25,711
2	Travel And Subsistence			£340,388		£340,388
	**Total**					£366,099

**A.4.**	**Information and communication costs**<br>**(e.g. brochures and leaflets for patients and their**					
No.	Cost item	Number of	Cost per item	Summary costs	Source of	**Total**
1						£0
	**Total**					£0

**A.5.**	**Clinical materials/equipment and**					
No.	Cost item	Number of	Cost per item	Summary costs	Source of	**Total**
1	Drugs			£203,900	Business	£203,900
2	Equipment			£14,589	Business	£14,589
3	Paramedical Supplies			£3,015	Business	£3,015
4	Surgical Appliances			£18	Business	£18
5	Surgical Sundries			£80,855	Business	£80,855
	**Total**					£302,377

**A.6.**	**Support services supplies**					
No.	Cost item	Number of	Cost per item	Summary costs	Source of	**Total**
1	Bedding And Linen			£112	Business	£112
2	Cleaning			£8,251	Business	£8,251
3	General Services			£2,595		£2,595
	**Total**					£10,958

**A.7.**	**Labs and diagnostics**					
No.	Cost item	Number of	Cost per item	Summary costs	Source of	**Total**
1	Cssd/diagnostic Supplies			£3,783		£3,783

						£3,783
**A.8.**	**Overhead costs**					
No.	Cost item	Number of	Cost per item	Summary costs	Source of	**Total**
1	Post Carriage And Telephones			£5,224		£5,224
2	Printing And Stationery			£5,737	Business	£5,737
3	Property Maintenance			£1,174		£1,174
4	Miscellaneous			£25	Business	£25
	**Total**					£12,160
**A.9.**	**Other costs**					
No.	Cost item	Number of	Cost per item	Summary costs	Source of	**Total**
1	Provisions			£6	Business	£6
2	Uniforms			£334	Business	£334
	**Total**					£340
**A.10.**	**Additional costs**					
No.	Cost item	Number of	Cost per item	Summary costs	Source of	**Total**
1	Other Operating Income**			-£92,377		-£92,377
	**Total**					-£92,377

**TOTAL**	**£4,527,65**
**Unit cost of HAH admission**	**£2,926.73**
**Unit cost of HAH bed day**	**£398.01**

Site three

		from:	01/01/2015 (dd/mm/yyyy)	PERIOD Until: (dd/mm/yyyy)	01/01/2016	12 Months
				Source of		
	Number of HAH admissions (in period)	598		ISD IPD data		
		598		business case		
	Length of HAH stay per episode (in days)	7.35	Mean	ISD IPD data		
		0.14	Standard error			
	HAH bed days (period)	4397				

A.1.	Staff costs					
No.	Profession	WTEs	Gross annual	Summary salary cost	Source of	Total
a)	Medical staff					
1	Consultant	1		£114,776	Business	£114,77
2	Specialty doctor	1		£79,224	Business	£79,224
3					Business	£0
4						£0
5						£0
b)	Nursing and pharmacy services					
1	Nurse (Band 6)	3		£125,484	Business	£125,48
2	Nurse (Band 5)	1.6		£53,256	Business	£53,256
c)	Allied health professions					
1	Occupational therapist	1		£45,156	Business	£45,156
2	Physiotherapist	1		£45,156	Business	£45,156
d)	Administration, ICT and management					
1	Admin Clerical	1		£23,664	Business	£23,664
e)	Support services staff					
1						£0
	Total					£486,71
A.2.	Trainning costs<br>Note: the time to attend a course should be included in					
No.	Profession	Number of	Cost per	Summary costs	Source of	Total
1	Trainning costs			£1,000		£1,000
	Total					£1,000
A.3.	Transport costs					
No.	Cost item	Number of	Cost per item	Summary costs	Source of	Total
1	Transport/travel			£20,000	Business	£20,000
	Total					£20,000
A.4.	Information and communication costs<br>(e.g. brochures and leaflets for patients and their family)					
No.	Cost item	Number of	Cost per item	Summary costs	Source of	Total
1						£0
	Total					£0
A.5.	Clinical materials/equipment and drugs					
No.	Cost item	Number of	Cost per item	Summary costs	Source of	Total
1	Drugs			£4,840	Business	£4,840
2	Medical supplies			£2,393	Business	£2,393
	Total					£7,233
A.6.	Support services supplies					
No.	Cost item	Number of	Cost per item	Summary costs	Source of	Total
1						£0
	Total					£0
A.7.	Labs and diagnostics					
No.	Cost item	Number of	Cost per item	Summary costs	Source of	Total
1						£0
						£0
A.8.	Overhead costs					
No.	Cost item	Number of	Cost per item	Summary costs	Source of	Total

No.	Cost item	Number of	Cost per item	Summary costs	Source of	Total
1	Phones, stationary etc.			£1,796	Business	£1,796
	Total					£1,796
A.9.	Other costs					
No.	Cost item	Number of	Cost per item	Summary costs	Source of	Total
1	Mischellaneous			£250		£250
	Total					£250
A.10	Additional costs					
No.	Cost item	Number of	Cost per item	Summary costs	Source of	Total
1						£0
	Total					£0

TOTAL	£516,99
Unit cost of HAH admission	£864.54
Unit cost of HAH bed day	£117.57

Appendix 2 Results of selecting PSM technique and plots of covariance balance before and after propensity score matching

	**Site one**		**Site two**		**Site three**	
Variable	*Costs* mean/median bias;Rubin's B/R	*Survival* mean/median bias;Rubin's B/R	*Costs* mean/median bias;Rubin's B/R	*Survival* mean/median bias;Rubin's B/R	*Costs* mean/median bias;Rubin's B/R	*Survival* mean/median bias;Rubin's B/R
Mahalanobis	7.5/4.2;51.4/1.56	7.2/3.7;48.6/1.54	7.6/6.7;46.1/1.54	7.3/6.7;43.9/1.53	6.3/4.7/38.4/1.69	6.3/3.5/38.4/1.52
1-to-1	2.9/2.8;14.1/0.90	1.9/1.6;12.1/0.84	1.4/1.4;9.4/0.97	2.2/2.2;14.6/1.14	2.7/2.7/14.6/1.02	2.3/2.6/14.9/0.73
K-to-1	1.9/1.6;11.3/0.76	1.9/1.5;12.0/0.81	1.8/1.5;11.0/0.83	2.4/2.4;13.6/0.76	3.6/2.9/16.5/0.99	2.8/2.0/16.5/0.94
Kernel	1.6/1.1;9.8/0.97	1.5/1.2;8.9/0.92	1.1/0.9;6.9/1.02	0.9/0.7;6.5/1.01	2.2/1.6/12.3/1.22	1.9/1.2/11.2/1.21
Local linear regression	1.5/1.2;9.4/0.89	1.6/1.4;9.4/0.89	1.7/1.0;11.0/0.32	2.3/1.4;12.8/0.43	1.8/1.6/9.6/1.27	1.6/1.2/8.5/1.35
Spline	2.9/2.6;15.7/0.94	2.4/2.0;14.9/0.91	3.2/2.6;17.5/0.46	3.2/2.3;21.0/1.07	3.9/3.1/21.6/0.47	3.9/2.3/25.7/1.02
IPW	11.5/5.8;83.2/0.76	11.5/5.6;83.1/0.75	11.6/8.3;61.3/0.92	11.2/7.8;60.2/0.89	10.5/8.5/52.2/0.77	10.2/8.5/50.9/0.77

Rubin's B: the absolute standardized difference of the means of the linear index of the propensity score in the treated and (matched) non-treated group; Rubin's R: the ratio of treated to (matched) non-treated variances of the propensity score index; Samples sufficiently balanced if B less than 25 and that R between 0.5 and 2.

Standardised percentage bias before and after local linear regression propensity score matching for costs in site one

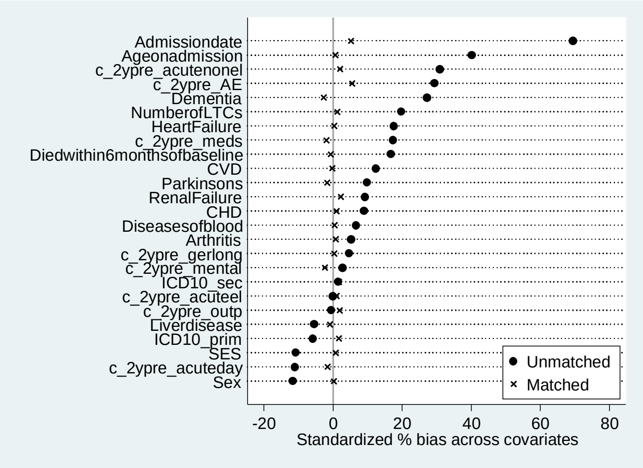

Standardised percentage bias before and after local linear regression propensity score matching for survival in site one

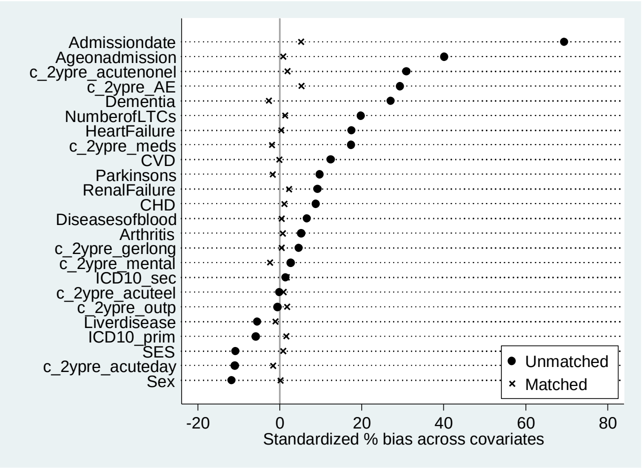

Standardised percentage bias before and after Kernel propensity score matching for costs in site two

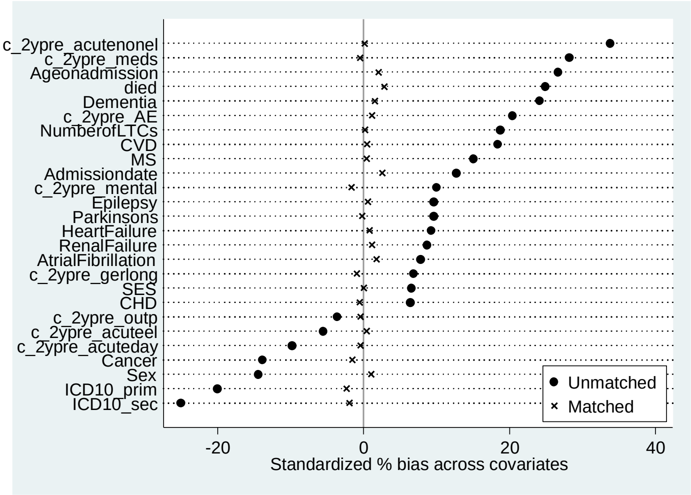

Standardised percentage bias before and after Kernel propensity score matching for survival in site two

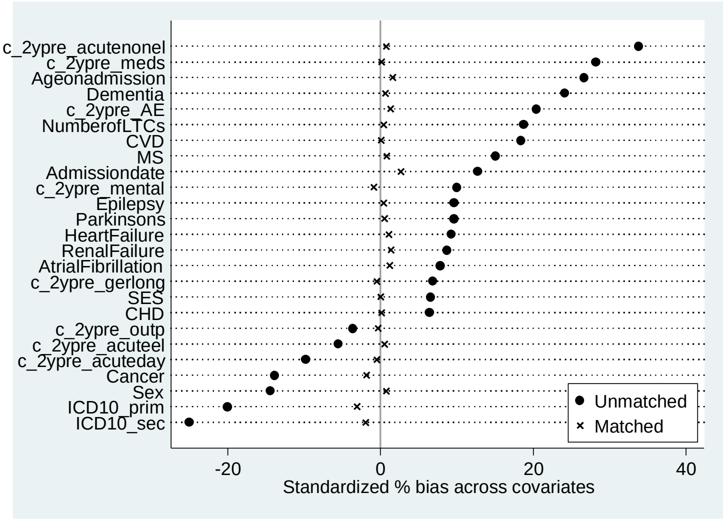

Standardised percentage bias before and after local linear regression propensity score matching for costs in site three

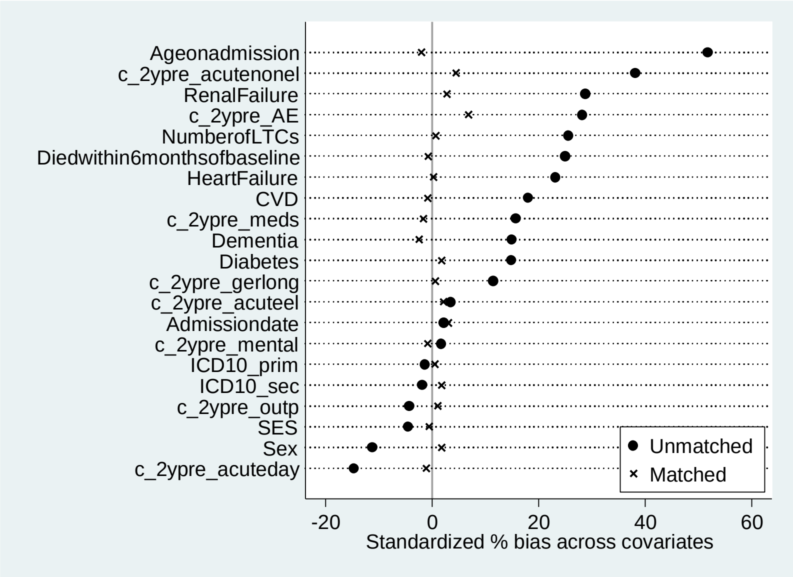

Standardised percentage bias before and after local linear regression propensity score matching for survival in site three

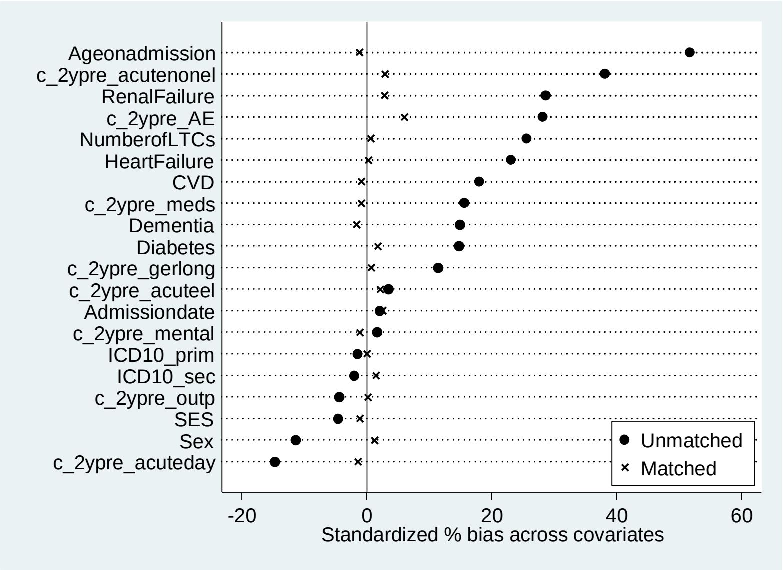

Propensity score distributions by cohort in each site

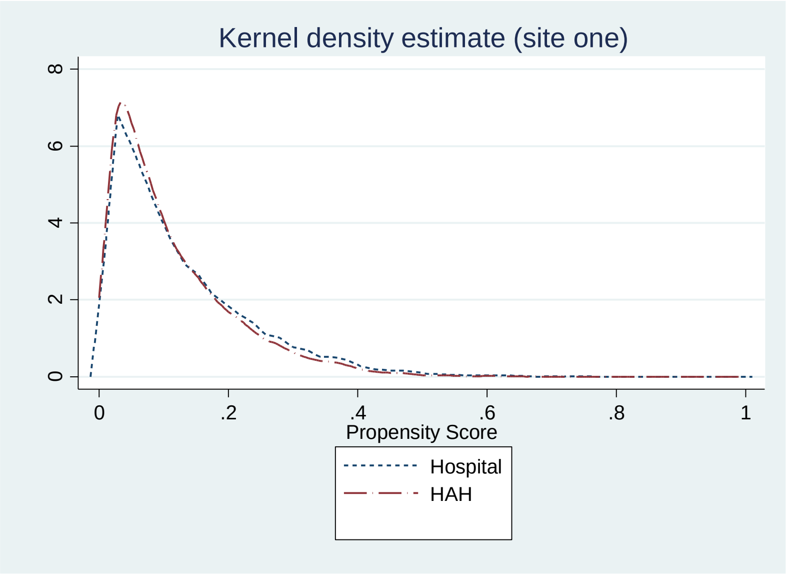

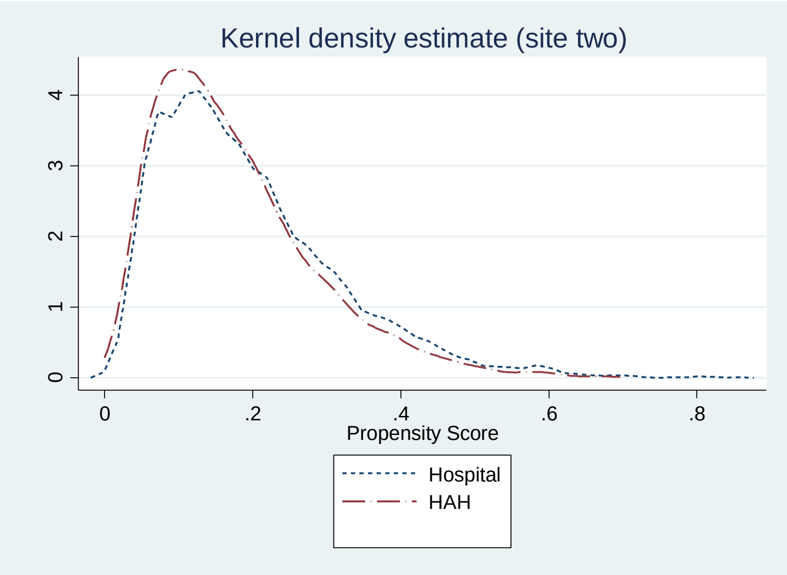

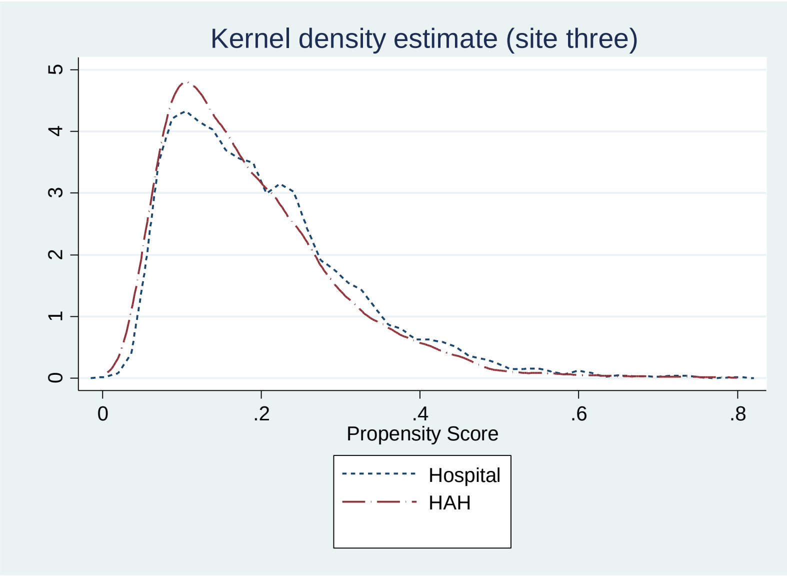

Patient characteristics at index admission after propensity score matching

	**Site one**		**Site two**		**Site three**	
Variable	Control	HAH	Control	HAH	Control	HAH
Mean age on admission (sd)	81.2 (7.95)	81.2 (7.20)	82.2 (8.03)	82.4 (7.68)	81.6 (7.96)	81.4 (7.10)
Female	63%	63%	62%	62%	62%	61%
Higher than 4 on the SIMD	35%	35%	53%	52%	44%	44%
More than 4 chronic conditions	44%	45%	48%	50%	43%	43%
Arthritis	29%	29%	38%	38%	33%	36%
Asthma	10%	11%	13%	14%	9%	11%
Atrial fibrillation	29%	28%	32%	33%	30%	29%
Cancer	28%	28%	28%	27%	30%	28%
CVD	27%	27%	26%	26%	27%	26%
Liver disease	3%	3%	4%	4%	4%	5%
COPD	27%	29%	26%	28%	26%	30%
Dementia	26%	25%	26%	26%	18%	17%
Diabetes	23%	23%	23%	24%	26%	26%
Epilepsy	4%	4%	5%	5%	2%	2%
CHD	42%	42%	40%	40%	37%	32%
Heart failure	23%	23%	22%	23%	25%	25%
MS	0%	0%	1%	1%	1%	1%
Parkinson’s	4%	4%	3%	3%	3%	5%
Renal failure	22%	23%	24%	24%	25%	25%
Congenital problems	2%	2%	4%	4%	2%	2%
Diseases of blood	32%	32%	30%	27%	29%	29%
Endocrine metabolic disease	36%	36%	46%	45%	39%	35%
Disease of digestive system	70%	72%	70%	70%	64%	66%

HAH: hospital-at-home; SIMD ranges from 1 (most deprived) to 10 (most affluent); Note: a patient could be registered with more than one ICD-10 codes

Appendix 3. Full results of the regression analyses

Association of hospital at home with total costs (after propensity score matching)

	**site one (n=13,267)**		**site two (n=4,769)**		**site three (n=2110)**	
	Follow-up period coefficient (se) [95%CI] p value	6 months after discharge coefficient (se) [95%CI] p value	Follow-up period coefficient (se) [95%CI] p value	6 months after discharge coefficient (se) [95%CI] p value	Follow-up period coefficient (se) [95%CI] p value	6 months after discharge coefficient (se) [95%CI] p value
HAH	0.82 (0.03) [0.76;0.89] <0.001	1.27 (0.07) [1.14;1.41] <0.001	1.00 (0.05) [0.92;1.09] 0.982	1.09 (0.07) [0.95;1.24] 0.219	1.15 (0.09) [0.99;1.33] 0.073	1.70 (0.17) [1.4;2.07] <0.001
Admission date	1.00 (0.00) [1.00;1.00] 0.058	1.00 (0.00) [1.00;1.00] 0.009	1.00 (0.00) [1.00;1.00] 0.386	1.00 (0.00) [1.00;1.00] 0.824	1.00 (0.00) [1.00;1.00] 0.009	1.00 (0.00) [1.00;1.00] 0.056
ICD10 primary	1.00 (0.00) [1.00;1.00] 0.660	1.00 (0.00) [1.00;1.00] 0.230	1.00 (0.00) [1.00;1.00] 0.001	1.00 (0.00) [1.00;1.00] <0.001	1.00 (0.00) [1.00;1.00] 0.162	1.00 (0.00) [1.00;1.00] 0.101
ICD10 secondary	1.00 (0.00) [1.00;1.00] 0.641	1.00 (0.00) [1.00;1.00] 0.988	1.00 (0.00) [1.00;1.00] 0.146	1.00 (0.00) [1.00;1.00] 0.238	1.00 (0.00) [1.00;1.00] 0.897	1.00 (0.00) [1.00;1.00] 0.971
2yrs pre AE costs	1.00 (0.00) [1.00;1.00] 0.240	1.00 (0.00) [1.00;1.00] 0.018	1.00 (0.00) [1.00;1.00] 0.624	1.00 (0.00) [1.00;1.00] 0.309	1.00 (0.00) [1.00;1.00] 0.284	1.00 (0.00) [1.00;1.00] 0.42
2yrs pre elective costs	1.00 (0.00) [1.00;1.00] 0.906	1.00 (0.00) [1.00;1.00] 0.919	1.00 (0.00) [1.00;1.00] 0.588	1.00 (0.00) [1.00;1.00] 0.435	1.00 (0.00) [1.00;1.00] 0.865	1.00 (0.00) [1.00;1.00] 0.931
2yrs pre non-elective costs	1.00 (0.00) [1.00;1.00] <0.001	1.00 (0.00) [1.00;1.00] 0.001	1.00 (0.00) [1.00;1.00] 0.694	1.00 (0.00) [1.00;1.00] 0.697	1.00 (0.00) [1.00;1.00] 0.018	1.00 (0.00) [1.00;1.00] 0.015
2yrs pre day case costs	1.00 (0.00) [1.00;1.00] 0.098	1.00 (0.00) [1.00;1.00] 0.020	1.00 (0.00) [1.00;1.00] 0.005	1.00 (0.00) [1.00;1.00] <0.001	1.00 (0.00) [1.00;1.00] 0.14	1.00 (0.00) [1.00;1.00] 0.100
2yrs pre geriatric ward costs	1.00 (0.00) [1.00;1.00] 0.005	1.00 (0.00) [1.00;1.00] 0.054	1.00 (0.00) [1.00;1.00] 0.001	1.00 (0.00) [1.00;1.00] 0.003	1.00 (0.00) [1.00;1.00] 0.634	1.00 (0.00) [1.00;1.00] 0.342
2yrs pre mental ward costs	1.00 (0.00) [1.00;1.00] 0.880	1.00 (0.00) [1.00;1.00] 0.911	1.00 (0.00) [1.00;1.00] 0.009	1.00 (0.00) [1.00;1.00] 0.014	1.00 (0.00) [1.00;1.00] 0.111	1.00 (0.00) [1.00;1.00] 0.382
2yrs pre outpatient costs	1.00 (0.00) [1.00;1.00] 0.087	1.00 (0.00) [1.00;1.00] 0.056	1.00 (0.00) [1.00;1.00] 0.026	1.00 (0.00) [1.00;1.00] 0.043	1.00 (0.00) [1.00;1.00] 0.683	1.00 (0.00) [1.00;1.00] 0.656
2yrs pre medication costs	1.00 (0.00) [1.00;1.00] 0.798	1.00 (0.00) [1.00;1.00] 0.750	1.00 (0.00) [1.00;1.00] 0.172	1.00 (0.00) [1.00;1.00] 0.369	1.00 (0.00) [1.00;1.00] 0.687	1.00 (0.00) [1.00;1.00] 0.935
Died during follow-up	1.03 (0.04) [0.95;1.11] 0.530	0.91 (0.05) [0.82;1.01] 0.089	1.05 (0.05) [0.96;1.15] 0.302	0.90 (0.06) [0.78;1.05] 0.143	1.06 (0.09) [0.90;1.24] 0.498	0.97 (0.11) [0.78;1.21] 0.784
Number of LTCs	1.09 (0.02) [1.05;1.12] <0.001	1.12 (0.02) [1.07;1.16] <0.001	1.04 (0.02) [1.00;1.07] 0.054	1.06 (0.03) [1.00;1.11] 0.035	1.06 (0.03) [1.01;1.11] 0.017	1.10 (0.03) [1.03;1.17] 0.003
Age on admission	1.00 (0.00) [0.99;1.01] 0.383	1.00 (0.00) [0.99;1.01] 0.981	1.00 (0.00) [0.99;1.01] 0.984	1.00 (0.00) [1.00;1.00] 0.349	1.01 (0.01) [1.00;1.02] 0.045	1.01 (0.01) [0.99;1.02] 0.41
Male	1.09 (0.05) [1.01;1.19] 0.034	1.08 (0.06) [0.97;1.19] 0.136	0.95 (0.05) [0.86;1.05] 0.340	0.99 (0.08) [0.85;1.15] 0.859	0.97 (0.08) [0.83;1.13] 0.709	0.98 (0.10) [0.81;1.2] 0.875
SES	1.00 (0.01) [0.98;1.02] 0.988	1.00 (0.01) [0.98;1.03] 0.741	1.01 (0.01) [1.00;1.03] 0.182	1.03 (0.01) [1.00;1.05] 0.033	1.00 (0.02) [0.97;1.03] 0.899	1.01 (0.02) [0.97;1.05] 0.779
Arthritis	0.96 (0.04) [0.88;1.05] 0.398	0.95 (0.05) [0.85;1.06] 0.346			------------	------------
Atrial Fibrillation	------------	------------	1.09 (0.06) [0.98;1.2] 0.098	1.13 (0.08) [0.97;1.30] 0.113	------------	------------
Cancer	------------	------------	1.04 (0.05) [0.94;1.15] 0.485	1.07 (0.08) [0.92;1.24] 0.403	------------	------------
CVD	1.01 (0.06) [0.91;1.13] 0.767	0.99 (0.07) [0.86;1.13] 0.903	1.08 (0.06) [0.97;1.2] 0.168	1.11 (0.09) [0.95;1.29] 0.199	1.10 (0.11) [0.90;1.34] 0.339	1.07 (0.13) [0.84;1.37] 0.585
Liver disease	1.21 (0.13) [0.98;1.50] 0.074	1.20 (0.14) [0.95;1.51] 0.130	------------	------------	------------	------------
Dementia	1.06 (0.05) [0.97;1.17] 0.179	1.07 (0.07) [0.95;1.21] 0.236	1.00 (0.05) [0.91;1.11] 0.942	1.03 (0.08) [0.89;1.19] 0.683	1.14 (0.11) [0.95;1.38] 0.166	1.17 (0.15) [0.91;1.5] 0.211
Epilepsy	------------	------------	1.04 (0.11) [0.85;1.27] 0.734	1.04 (0.15) [0.78;1.38] 0.803	------------	------------
CHD	0.85 (0.05) [0.77;0.95] 0.004	0.83 (0.06) [0.73;0.95] 0.008	1.01 (0.06) [0.9;1.13] 0.871	1.02 (0.08) [0.88;1.20] 0.766	------------	------------
Heart Failure	1.09 (0.06) [0.98;1.20] 0.102	1.10 (0.07) [0.97;1.24] 0.154	1.08 (0.06) [0.96;1.21] 0.186	1.08 (0.09) [0.92;1.28] 0.363	1.01 (0.10) [0.83;1.23] 0.919	0.98 (0.13) [0.76;1.26] 0.879
Multiple sclerosis	------------	------------	0.74 (0.10) [0.57;0.98] 0.033	0.59 (0.15) [0.36;0.97] 0.035	------------	------------
Parkinson's	1.24 (0.11) [1.03;1.48] 0.019	1.20 (0.14) [0.95;1.51] 0.120	1.09 (0.15) [0.83;1.42] 0.554	1.09 (0.20) [0.75;1.57] 0.664	------------	------------
Renal Failure	1.03 (0.05) [0.94;1.13] 0.513	1.06 (0.06) [0.94;1.19] 0.362	1.05 (0.06) [0.94;1.17] 0.420	1.08 (0.09) [0.92;1.26] 0.348	1.12 (0.12) [0.9;1.38] 0.306	1.14 (0.16) [0.87;1.49] 0.346
Diseases of blood	1.05 (0.05) [0.96;1.15] 0.275	1.05 (0.06) [0.94;1.18] 0.363	------------	------------	------------	------------
Diabetes	------------	------------	------------	------------	1.21 (0.11) [1.01;1.45] 0.043	1.24 (0.14) [0.99;1.55] 0.061
Constant	15.93 (46.90) [0.05;5098.92] 0.347	0.19 (0.68) [0.00;224.04] 0.644	285486.5 (1267507) [47.47; 1.72E+09] 0.005	899.53 (5743.23) [0.00;0.00] 0.287	20700000000000 (186000000000000) [500612.1;8.6E+20] 0.001	2230000000000 (25100000000000) [559.85;8.85E+21] 0.012

driven mainly by non-elective hospital care; Note the HAH unit costs in site one were £628.34 per admission to HAH and have been added to the costs during the episode.

Association of hospital-at-home with mortality risk during study period (after propensity score matching)

	**site one (n=13,267)**	**site two (n=4,771)**	**site three (n=2110)**
	coefficient (se) [95%CI] p value	coefficient (se) [95%CI] p value	coefficient (se) [95%CI] p value
HAH	1.09 (0.05) [1.00;1.19] 0.059	1.29 (0.07) [1.15;1.44] <0.0010	1.27 (0.12) [1.06;1.54] 0.011
Admission date	1.00 (0.00) [1.00;1.00] 0.842	1.00 (0.00) [1.00;1.00] 0.100	1 (0) [1;1] 0.687
ICD10 primary	1.00 (0.00) [1.00;1.00] <0.001	1.00 (0.00) [1.00;1.00] 0.001	1 (0) [1;1] 0.006
ICD10 secondary	1.00 (0.00) [1.00;1.00] <0.001	1.00 (0.00) [1.00;1.00] 0.023	1 (0) [1;1] 0.359
2yrs pre AE costs	1.00 (0.00) [1.00;1.00] 0.640	1.00 (0.00) [1.00;1.00] 0.153	1 (0) [1;1] 0.027
2yrs pre elective costs	1.00 (0.00) [1.00;1.00] 0.487	1.00 (0.00) [1.00;1.00] 0.462	1 (0) [1;1] 0.079
2yrs pre non-elective costs	1.00 (0.00) [1.00;1.00] 0.001	1.00 (0.00) [1.00;1.00] 0.007	1 (0) [1;1] 0.052
2yrs pre day case costs	1.00 (0.00) [1.00;1.00] <0.001	1.00 (0.00) [1.00;1.00] 0.001	1 (0) [1;1] 0.903
2yrs pre geriatric ward costs	1.00 (0.00) [1.00;1.00] 0.022	1.00 (0.00) [1.00;1.00] <0.001	1 (0) [1;1] 0.338
2yrs pre mental ward costs	1.00 (0.00) [1.00;1.00] 0.419	1.00 (0.00) [1.00;1.00] 0.943	1 (0) [1;1] 0
2yrs pre outpatient costs	1.00 (0.00) [1.00;1.00] 0.091	1.00 (0.00) [1.00;1.00] 0.882	1 (0) [1;1] 0.001
2yrs pre medication costs	1.00 (0.00) [1.00;1.00] 0.044	1.00 (0.00) [1.00;1.00] 0.037	1 (0) [1;1] 0
Number of LTCs	1.03 (0.02) [0.99;1.07] 0.120	0.96 (0.02) [0.92;1.01] 0.107	1.07 (0.04) [1;1.14] 0.048
Age on admission	1.04 (0) [1.03;1.04] <0.001	1.03 (0.00) [1.02;1.04] <0.001	1.04 (0.01) [1.02;1.05] 0
Male	1.12 (0.05) [1.01;1.22] 0.017	1.23 (0.08) [1.09;1.39] 0.001	1.37 (0.14) [1.12;1.67] 0.002
SES	0.97 (0.01) [0.95;0.99] 0.001	0.98 (0.01) [0.96;1.00] 0.088	1.01 (0.02) [0.98;1.05] 0.483
Arthritis	0.86 (0.05) [0.77;0.97] 0.008	- - - - - - - - - - - -	- - - - - - - - - - - -
Atrial Fibrillation	- - - - - - - - - - - -	1.11 (0.08) [0.97;1.28] 0.133	- - - - - - - - - - - -
Cancer	- - - - - - - - - - - -	1.86 (0.12) [1.64;2.11] <0.001	- - - - - - - - - - - -
CVD	0.94 (0.06) [0.83;1.05] 0.276	1.06 (0.08) [0.92;1.22] 0.438	0.95 (0.12) [0.74;1.21] 0.673
Liver disease	1.33 (0.16) [1.04;1.67] 0.015	- - - - - - - - - - - -	- - - - - - - - - - - -
Dementia	1.11 (0.06) [1.00;1.25] 0.058	1.59 (0.11) [1.39;1.82] <0.001	1.31 (0.16) [1.03;1.67] 0.025
Epilepsy		1.19 (0.17) [0.91;1.57] 0.207	- - - - - - - - - - - -
CHD	0.91 (0.05) [0.82;1.03] 0.114	0.93 (0.07) [0.80;1.08] 0.345	- - - - - - - - - - - -
Heart Failure	1.13 (0.07) [1.00;1.28] 0.052	1.35 (0.11) [1.15;1.57] <0.001	1.16 (0.15) [0.9;1.5] 0.256
Multiple sclerosis	- - - - - - - - - - - -	1.54 (0.39) [0.94;2.52] 0.086	- - - - - - - - - - - -
Parkinson’s	1.11 (0.13) [0.86;1.39] 0.374	0.93 (0.17) [0.65;1.33] 0.678	- - - - - - - - - - - -
Renal Failure	1.07 (0.07) [0.95;1.21] 0.292	1.35 (0.10) [1.16;1.56] <0.001	0.93 (0.12) [0.72;1.2] 0.571
Diseases of blood	0.93 (0.05) [0.85;1.06] 0.201	- - - - - - - - - - - -	- - - - - - - - - - - -
Diabetes	- - - - - - - - - - - -	- - - - - - - - - - - -	0.74 (0.1) [0.57;0.97] 0.026
Constant	0.01 (0.04) [0.00;7.06] 0.174	0.00 (0.00) [0.00;0.18] 0.025	0 (0) [0;319640.8] 0.405

Results of the subgroup analysis including only patients with dementia (costs)

	**site one (n=2,321)**		**site two (n=1,053)**		**site three (n=280)**	
	Follow-up period	Total costs in 6 months after discharge	Follow-up period	Total costs in 6 months after discharge	Follow-up period	Total costs in 6 months after discharge
	coefficient (se) [95%CI] p value	coefficient (se) [95%CI] p value	coefficient (se) [95%CI] p value	coefficient (se) [95%CI] p value	coefficient (se) [95%CI] p value	coefficient (se) [95%CI] p value
HAH (hospital)	0.76 (0.05) [0.66;0.87] 0	1.18 (0.11) [0.99;1.41] 0.071	0.76 (0.06) [0.66;0.88] 0	0.75 (0.09) [0.59;0.96] 0.021	0.87 (0.15) [0.63;1.21] 0.409	1.58 (0.41) [0.95;2.63] 0.078
Admission date	1 (0) [1;1] 0.528	1.00 (0.00) [1.00;1.00] 0.329	1 (0) [1;1] 0.513	1.00 (0.00) [1.00;1.00] 0.532	1 (0) [1;1] 0.002	1 (0) [0.99;1] 0.003
ICD10 primary	1 (0) [1;1] 0.025	1.00 (0.00) [1.00;1.00] 0.003	1 (0) [1;1] 0.079	1.00 (0.00) [1.00;1.00] 0.008	1 (0) [1;1] 0.666	1 (0) [1;1] 0.123
ICD10 secondary	1 (0) [1;1] 0.027	1.00 (0.00) [1.00;1.00] 0.086	- - - - - - - - - - - -	- - - - - - - - - - - -	1 (0) [1;1] 0.946	1 (0) [1;1] 0.594
2yrs pre AE costs	1 (0) [1;1] 0.063	1.00 (0.00) [1.00;1.00] 0.021	1 (0) [1;1] 0.979	1.00 (0.00) [1.00;1.00] 0.93	1 (0) [1;1] 0.57	1 (0) [1;1] 0.331
2yrs pre elective costs	1 (0) [1;1] 0.913	1.00 (0.00) [1.00;1.00] 0.708	1 (0) [1;1] 0.979	1.00 (0.00) [1.00;1.00] 0.889	1 (0) [1;1] 0.115	1 (0) [1;1] 0.208
2yrs pre non-elective costs	1 (0) [1;1] 0.564	1.00 (0.00) [1.00;1.00] 0.605	1 (0) [1;1] 0.031	1.00 (0.00) [1.00;1.00] 0.008	1 (0) [1;1] 0.888	1 (0) [1;1] 0.639
2yrs pre day case costs	1 (0) [1;1] 0.455	1.00 (0.00) [1.00;1.00] 0.632	1 (0) [1;1] 0.725	1.00 (0.00) [1.00;1.00] 0.307	1 (0) [1;1] 0.1	1 (0) [1;1] 0.279
2yrs pre geriatric ward costs	1 (0) [1;1] 0.233	1.00 (0.00) [1.00;1.00] 0.566	1 (0) [1;1] 0.012	1.00 (0.00) [1.00;1.00] 0.003	1 (0) [1;1] 0.907	1 (0) [1;1] 0.952
2yrs pre mental ward costs	1 (0) [1;1] 0.343	1.00 (0.00) [1.00;1.00] 0.335	1 (0) [1;1] 0.084	1.00 (0.00) [1.00;1.00] 0.042	1 (0) [1;1] 0.01	1 (0) [1;1] 0.021
2yrs pre outpatient costs	1 (0) [1;1] 0.066	1.00 (0.00) [1.00;1.00] 0.082	1 (0) [1;1] 0.001	1.00 (0.00) [1.00;1.00] 0.001	1 (0) [1;1] 0.685	1 (0) [1;1] 0.403
2yrs pre medication costs	1 (0) [1;1] 0.306	1.00 (0.00) [1.00;1.00] 0.316	1 (0) [1;1] 0.13	1.00 (0.00) [1.00;1.00] 0.265	1 (0) [1;1] 0.042	1 (0) [1;1] 0.044
Died within 6months	0.81 (0.06) [0.7;0.94] 0.005	0.70 (0.07) [0.58;0.85] <0.001	0.89 (0.07) [0.76;1.03] 0.118	0.73 (0.09) [0.58;0.93] 0.011	0.66 (0.13) [0.45;0.96] 0.031	0.44 (0.13) [0.25;0.77] 0.004
Number of LTCs	1.06 (0.03) [1;1.12] 0.069	1.07 (0.04) [1.00;1.16] 0.063	1.08 (0.03) [1.02;1.14] 0.006	1.15 (0.05) [1.05;1.26] 0.003	1.04 (0.06) [0.94;1.16] 0.443	1.01 (0.08) [0.86;1.18] 0.935
Age on admission	0.99 (0.01) [0.98;1] 0.094	0.98 (0.01) [0.97;1.00] 0.015	0.98 (0.01) [0.97;1] 0.007	0.97 (0.01) [0.95;0.99] 0.003	1 (0.01) [0.98;1.03] 0.933	1 (0.02) [0.97;1.03] 0.946
Male	1.13 (0.08) [0.99;1.31] 0.076	1.14 (0.11) [0.95;1.37] 0.151	0.95 (0.07) [0.82;1.11] 0.511	0.95 (0.12) [0.74;1.22] 0.679	1.05 (0.17) [0.76;1.43] 0.78	1.07 (0.26) [0.67;1.71] 0.774
SES	1.01 (0.01) [0.98;1.04] 0.693	1.01 (0.02) [0.97;1.04] 0.77	1.03 (0.01) [1;1.05] 0.053	1.06 (0.02) [1.01;1.10] 0.010	1.03 (0.03) [0.97;1.09] 0.3	1.04 (0.04) [0.96;1.12] 0.3
Atrial Fibrillation	- - - - - - - - - - - -	- - - - - - - - - - - -	1.03 (0.09) [0.87;1.23] 0.722	1.00 (0.14) [0.77;1.31] 0.986	- - - - - - - - - - - -	- - - - - - - - - - - -
Arthritis	1.02 (0.09) [0.86;1.2] 0.833	1.02 (0.11) [0.83;1.25] 0.862	- - - - - - - - - - - -	- - - - - - - - - - - -	- - - - - - - - - - - -	- - - - - - - - - - - -
Cancer	- - - - - - - - - - - -	- - - - - - - - - - - -	1.04 (0.1) [0.87;1.24] 0.679	1.06 (0.16) [0.79;1.43] 0.688	- - - - - - - - - - - -	- - - - - - - - - - - -
CVD	0.92 (0.07) [0.78;1.08] 0.3	0.91 (0.1) [0.74;1.12] 0.374	0.98 (0.08) [0.83;1.16] 0.845	0.95 (0.14) [0.72;1.26] 0.741	1.39 (0.28) [0.94;2.06] 0.103	1.65 (0.48) [0.93;2.91] 0.085
Liver disease	0.8 (0.12) [0.59;1.08] 0.138	0.8 (0.16) [0.54;1.20] 0.286	- - - - - - - - - - - -	- - - - - - - - - - - -	- - - - - - - - - - - -	- - - - - - - - - - - -
CHD	1.01 (0.09) [0.85;1.2] 0.917	1.05 (0.12) [0.84;1.30] 0.688	0.94 (0.09) [0.78;1.12] 0.482	0.98 (0.14) [0.74;1.30] 0.891	- - - - - - - - - - - -	- - - - - - - - - - - -
Epilepsy	- - - - - - - - - - - -	- - - - - - - - - - - -	0.97 (0.15) [0.72;1.3] 0.842	0.78 (0.16) [0.53;1.16] 0.221	- - - - - - - - - - - -	- - - - - - - - - - - -
Heart Failure	1.03 (0.11) [0.83;1.27] 0.818	1.02 (0.14) [0.79;1.33] 0.878	0.92 (0.11) [0.73;1.15] 0.452	0.90 (0.17) [0.62;1.29] 0.558	0.83 (0.19) [0.53;1.3] 0.409	1.16 (0.42) [0.57;2.37] 0.687
Multiple sclerosis	- - - - - - - - - - - -	- - - - - - - - - - - -	0.4 (0.06) [0.29;0.54] 0	0.18 (0.07) [0.09;0.37] <0.001	- - - - - - - - - - - -	- - - - - - - - - - - -
Parkinson’s	1.13 (0.15) [0.88;1.46] 0.333	1.00 (0.17) [0.72;1.39] 0.992	0.87 (0.14) [0.63;1.18] 0.365	0.68 (0.20) [0.39;1.20] 0.188	- - - - - - - - - - - -	- - - - - - - - - - - -
Renal Failure	1.03 (0.1) [0.85;1.24] 0.769	1.12 (0.14) [0.88;1.42] 0.354	0.9 (0.09) [0.75;1.09] 0.296	0.82 (0.13) [0.60;1.12] 0.203	1.2 (0.24) [0.81;1.78] 0.354	1.25 (0.35) [0.72;2.17] 0.435
Diseases of blood	0.93 (0.08) [0.79;1.11] 0.437	0.90 (0.1) [0.73;1.11] 0.337	- - - - - - - - - - - -	- - - - - - - - - - - -	- - - - - - - - - - - -	- - - - - - - - - - - -
Diabetes	- - - - - - - - - - - -	- - - - - - - - - - - -	- - - - - - - - - - - -	- - - - - - - - - - - -	0.85 (0.18) [0.55;1.3] 0.449	0.92 (0.26) [0.52;1.6] 0.756
Constant	469.5 (2319.98) [0.03;7547051] 0.213	22.71 (140.52) [0;4194325] 0.614	2796754 (19900000) [2.38;3290000000000] 0.037	40500000 (472000000) [0;329000000000000000] 0.132	2.82E+29 (5.36E+30) [18000000000000;4.43E+45] 0	3.34E+38 (9.1E+39) [2100000000000000;5.29E+61] 0.001

Results of the subgroup analysis including only patients with dementia (mortality risk)

	**site one (n=2,321)**	**site two (n=1,053)**	**site three (n=280)**
	Mortality rate during follow-up coefficient (se) [95%CI] p value	Mortality rate during follow-up coefficient (se) [95%CI] p value	Mortality rate during follow-up coefficient (se) [95%CI] p value
HAH (hospital)	1.05 (0.09) [0.89;1.24] 0.594	1.41 (0.12) [1.19;1.67] <0.001	1.65 (0.32) [1.12;2.41] 0.011
Admission date	1.00 (0.00) [1.00;1.00] 0.19	1.00 (0.00) [1.00;1.00] 0.001	1 (0) [1;1] 0.788
ICD10 primary	1.00 (0.00) [1.00;1.00] <0.001	1.00 (0.00) [1.00;1.00] 0.001	1 (0) [1;1] 0.14
ICD10 secondary	1.00 (0.00) [1.00;1.00] 0.207	- - - - - - - - - - - -	1 (0) [1;1] 0.979
2yrs pre AE costs	1.00 (0.00) [1.00;1.00] 0.251	1.00 (0.00) [1.00;1.00] 0.609	1 (0) [1;1] 0.029
2yrs pre elective costs	1.00 (0.00) [1.00;1.00] 0.735	1.00 (0.00) [1.00;1.00] 0.129	1 (0) [1;1] 0.554
2yrs pre non-elective costs	1.00 (0.00) [1.00;1.00] 0.173	1.00 (0.00) [1.00;1.00] 0.484	1 (0) [1;1] 0.814
2yrs pre day case costs	1.00 (0.00) [1.00;1.00] 0.088	1.00 (0.00) [1.00;1.00] 0.004	1 (0) [1;1] 0.896
2yrs pre geriatric ward costs	1.00 (0.00) [1.00;1.00] 0.644	1.00 (0.00) [1.00;1.00] <0.001	1 (0) [1;1] 0.783
2yrs pre mental ward costs	1.00 (0.00) [1.00;1.00] 0.569	1.00 (0.00) [1.00;1.00] 0.112	1 (0) [1;1] 0
2yrs pre outpatient costs	1.00 (0.00) [1.00;1.00] 0.070	1.00 (0.00) [1.00;1.00] 0.167	1 (0) [1;1] 0
2yrs pre medication costs	1.00 (0.00) [1.00;1.00] 0.004	1.00 (0.00) [1.00;1.00] 0.156	1 (0) [1;1] 0.011
Died within 6months	- - - - - - - - - - - -	- - - - - - - - - - - -	- - - - - - - - - - - -
Number of LTCs	0.94 (0.03) [0.88;1.01] 0.113	0.95 (0.03) [0.89;1.01] 0.115	0.98 (0.07) [0.86;1.13] 0.827
Age on admission	1.04 (0.01) [1.02;1.05] <0.001	1.03 (0.01) [1.01;1.04] <0.001	1.04 (0.02) [1;1.07] 0.024
Male	1.19 (0.11) [0.99;1.42] 0.063	1.17 (0.10) [0.99;1.38] 0.070	1.18 (0.25) [0.78;1.79] 0.43
SES	0.97 (0.02) [0.94;1.01] 0.134	1.00 (0.02) [0.97;1.03] 0.991	0.96 (0.04) [0.88;1.04] 0.3
Atrial Fibrillation	- - - - - - - - - - - -	1.03 (0.11) [0.85;1.26] 0.75	- - - - - - - - - - - -
Arthritis	1.06 (0.11) [0.86;1.30] 0.600	- - - - - - - - - - - -	- - - - - - - - - - - -
Cancer	- - - - - - - - - - - -	1.40 (0.13) [1.16;1.68] <0.001	- - - - - - - - - - - -
CVD	1.55 (0.41) [0.92;2.61] 0.099	1.14 (0.11) [0.94;1.39] 0.176	1.02 (0.25) [0.63;1.65] 0.925
Liver disease	0.98 (0.11) [0.79;1.21] 0.845	- - - - - - - - - - - -	- - - - - - - - - - - -
CHD	1.21 (0.16) [0.94;1.56] 0.135	0.99 (0.10) [0.81;1.20] 0.885	- - - - - - - - - - - -
Epilepsy	- - - - - - - - - - - -	1.26 (0.19) [0.94;1.70] 0.120	- - - - - - - - - - - -
Heart Failure	1.21 (0.16) [0.94;1.56] 0.135	1.33 (0.17) [1.04;1.70] 0.023	1.88 (0.49) [1.12;3.14] 0.017
Multiple sclerosis	- - - - - - - - - - - -	0.96 (0.51) [0.34;2.72] 0.932	- - - - - - - - - - - -
Parkinson's	1.26 (0.22) [0.9;1.78] 0.180	1.04 (0.20) [0.71;1.51] 0.848	- - - - - - - - - - - -
Renal Failure	1.06 (0.12) [0.84;1.32] 0.637	1.15 (0.12) [0.93;1.41] 0.192	0.56 (0.16) [0.32;0.97] 0.037
Diseases of blood	0.96 (0.11) [0.77;1.19] 0.709	- - - - - - - - - - - -	- - - - - - - - - - - -
Diabetes	- - - - - - - - - - - -	- - - - - - - - - - - -	0.6 (0.2) [0.32;1.15] 0.123
Constant	0.00 (0.00) [0.00;1.37] 0.057	0.00 (0.00) [0.00;0.00] <0.001	0 (0) [0;1810000000000000] 0.652

Results of the subgroup analysis excluding those who had died

	site one (n=10,132)		site two (n=3,584)		site three (n=1691)	
	Follow-up period	Total costs in 6 months after discharge	Follow-up period	Total costs in 6 months after discharge	Follow-up period	Total costs in 6 months after discharge
	coefficient (se) [95%CI] p value	coefficient (se) [95%CI] p value	coefficient (se) [95%CI] p value	coefficient (se) [95%CI] p value	coefficient (se) [95%CI] p value	coefficient (se) [95%CI] p value
HAH (hospital)	0.85 (0.04) [0.77;0.94] 0.002	1.23 (0.08) [1.08;1.4] 0.002	1.11 (0.06) [1;1.25] 0.058	1.17 (0.10) [0.99;1.38] 0.070	1.20 (0.11) [1;1.43] 0.046	1.71 (0.20) [1.36;2.15] <0.001
Admission date	1 (0) [1;1] 0.076	1.00 (0.00) [1.00;1.00] 0.032	1 (0) [1;1] 0.833	1.00 (0.00) [1.00;1.00] 0.337	1 (0) [1;1] 0.075	1 (0) [1;1] 0.282
ICD10 primary	1 (0) [1;1] 0.692	1.00 (0.00) [1.00;1.00] 0.993	1 (0) [1;1] 0.126	1.00 (0.00) [1.00;1.00] 0.038	1 (0) [1;1] 0.282	1 (0) [1;1] 0.279
ICD10 secondary	1 (0) [1;1] 0.817	1.00 (0.00) [1.00;1.00] 0.473	1 (0) [1;1] 0.014	1.00 (0.00) [1.00;1.00] 0.024	1 (0) [1;1] 0.724	1 (0) [1;1] 0.801
2yrs pre AE costs	1 (0) [1;1] 0.08	1.00 (0.00) [1.00;1.00] 0.012	1 (0) [1;1] 0.461	1.00 (0.00) [1.00;1.00] 0.135	1 (0) [1;1] 0.435	1 (0) [1;1] 0.761
2yrs pre elective costs	1 (0) [1;1] 0.015	1.00 (0.00) [1.00;1.00] 0.046	1 (0) [1;1] 0.576	1.00 (0.00) [1.00;1.00] 0.429	1 (0) [1;1] 0.63	1 (0) [1;1] 0.725
2yrs pre non-elective costs	1 (0) [1;1] 0	1.00 (0.00) [1.00;1.00] <0.001	1 (0) [1;1] 0.651	1.00 (0.00) [1.00;1.00] 0.700	1 (0) [1;1] 0.199	1 (0) [1;1] 0.01
2yrs pre day case costs	1 (0) [1;1] 0.416	1.00 (0.00) [1.00;1.00] 0.158	1 (0) [1;1] 0.057	1.00 (0.00) [1.00;1.00] 0.023	1 (0) [1;1] 0.068	1 (0) [1;1] 0.064
2yrs pre geriatric ward costs	1 (0) [1;1] 0.031	1.00 (0.00) [1.00;1.00] 0.029	1 (0) [1;1] 0.625	1.00 (0.00) [1.00;1.00] 0.806	1 (0) [1;1] 0.484	1 (0) [1;1] 0.103
2yrs pre mental ward costs	1 (0) [1;1] 0.206	1.00 (0.00) [1.00;1.00] 0.166	1 (0) [1;1] 0.009	1.00 (0.00) [1.00;1.00] 0.020	1 (0) [1;1] 0.01	1 (0) [1;1] 0.004
2yrs pre outpatient costs	1 (0) [1;1] 0.236	1.00 (0.00) [1.00;1.00] 0.187	1 (0) [1;1] 0.748	1.00 (0.00) [1.00;1.00] 0.802	1 (0) [1;1] 0.798	1 (0) [1;1] 0.908
2yrs pre medication costs	1 (0) [1;1] 0.399	1.00 (0.00) [1.00;1.00] 0.383	1 (0) [1;1] 0.011	1.00 (0.00) [1.00;1.00] 0.016	1 (0) [1;1] 0.37	1 (0) [1;1] 0.77
Number of LTCs	1.08 (0.02) [1.04;1.12] 0	1.12 (0.03) [1.07;1.18] <0.001	1.03 (0.02) [0.99;1.08] 0.169	1.06 (0.04) [0.99;1.13] 0.076	1.06 (0.03) [1.01;1.13] 0.032	1.09 (0.04) [1.01;1.17] 0.026
Age on admission	1.01 (0) [1;1.01] 0.025	1.01 (0.00) [1.00;1.02] 0.048	1.01 (0) [1;1.01] 0.054	1.01 (0.01) [1.00;1.02] 0.254	1.02 (0.01) [1;1.03] 0.019	1.01 (0.01) [0.99;1.03] 0.171
Male	1.11 (0.06) [1;1.22] 0.051	1.12 (0.07) [0.99;1.26] 0.085	0.94 (0.06) [0.83;1.07] 0.353	0.97 (0.09) [0.80;1.17] 0.752	0.97 (0.09) [0.8;1.16] 0.716	1 (0.12) [0.79;1.26] 0.974
SES	1 (0.01) [0.98;1.02] 0.965	1 (0.01) [0.98;1.03] 0.778	1.02 (0.01) [1;1.04] 0.081	1.03 (0.01) [1.00;1.06] 0.023	1 (0.02) [0.96;1.03] 0.822	1 (0.02) [0.95;1.05] 0.951
Atrial Fibrillation	------------	------------	1.07 (0.07) [0.94;1.21] 0.305	1.09 (0.10) [0.92;1.29] 0.335	------------	------------
Arthritis	0.99 (0.05) [0.89;1.1] 0.889	0.96 (0.06) [0.85;1.1] 0.584	------------	------------	------------	------------
Cancer	------------	------------	1 (0.07) [0.88;1.15] 0.961	1.01 (0.10) [0.84;1.23] 0.899	------------	------------
CVD	1.04 (0.07) [0.91;1.2] 0.552	1.00 (0.09) [0.85;1.19] 0.956	1.14 (0.08) [1;1.3] 0.058	1.14 (0.11) [0.95;1.36] 0.174	1.12 (0.14) [0.88;1.43] 0.367	1.1 (0.17) [0.81;1.5] 0.531
Liver disease	1.35 (0.2) [1.01;1.8] 0.045	1.31 (0.21) [0.95;1.81] 0.097	------------	------------	------------	------------
Dementia	1.16 (0.07) [1.04;1.3] 0.009	1.17 (0.08) [1.01;1.35] 0.033	1.08 (0.07) [0.96;1.22] 0.195	1.11 (0.10) [0.93;1.31] 0.244	1.37 (0.16) [1.09;1.73] 0.008	1.49 (0.23) [1.09;2.02] 0.011
CHD	0.82 (0.06) [0.72;0.94] 0.004	0.79 (0.07) [0.67;0.93] 0.004	1.01 (0.07) [0.87;1.16] 0.941	1.03 (0.10) [0.85;1.24] 0.799	------------	------------
Epilepsy	------------	------------	1.08 (0.12) [0.86;1.35] 0.518	1.09 (0.17) [0.80;1.48] 0.581	------------	------------
Heart Failure	1.1 (0.07) [0.97;1.25] 0.131	1.08 (0.08) [0.93;1.26] 0.293	1.08 (0.08) [0.94;1.24] 0.287	1.07 (0.11) [0.88;1.31] 0.491	1.05 (0.13) [0.82;1.34] 0.719	1.01 (0.16) [0.74;1.39] 0.932
Multiple sclerosis	------------	------------	0.72 (0.14) [0.49;1.06] 0.095	0.66 (0.21) [0.35;1.25] 0.202	------------	------------
Parkinson’s	1.19 (0.1) [1;1.41] 0.05	1.15 (0.13) [0.93;1.43] 0.19	1.22 (0.18) [0.91;1.64] 0.193	1.34 (0.27) [0.91;1.98] 0.139	------------	------------
Renal Failure	1.01 (0.06) [0.89;1.14] 0.911	1.00 (0.07) [0.87;1.16] 0.949	1.06 (0.08) [0.92;1.22] 0.443	1.06 (0.11) [0.86;1.29] 0.602	1.12 (0.15) [0.86;1.46] 0.411	1.19 (0.2) [0.85;1.66] 0.317
Diseases of blood	1.04 (0.06) [0.94;1.16] 0.414	1.04 (0.07) [0.92;1.19] 0.516	------------	------------	------------	------------
Diabetes	------------	------------	------------	------------	1.33 (0.15) [1.07;1.65] 0.01	1.37 (0.19) [1.04;1.81] 0.026
Constant	3.67 (13.85) [0;5959] 0.73	0.07 (0.31) [0;592.13] 0.558	1064.79 (5943.4) [0.02;60000000] 0.212	0.89 (6.96) [0;4301665] 0.988	101000000000 (1050000000000) [149.57;681000000000000000000] 0.015	1320000000 (18000000000) [0;5.67E+20] 0.124

Results of the sensitivity analysis

	site one (n=13,267)		site two (n=4,769)		site three (n=2110)	
	Total costs in follow-up (50% higher HAH unit costs) coefficient (se) [95%CI] p value	Total costs in follow-up (50% lower HAH unit costs) coefficient (se) [95%CI] p value	Total costs in follow-up (50% higher HAH unit costs) coefficient (se) [95%CI] p value	Total costs in follow-up (50% lower HAH unit costs) coefficient (se) [95%CI] p value	Total costs in follow-up (50% higher HAH unit costs) coefficient (se) [95%CI] p value	Total costs in follow-up (50% lower HAH unit costs) coefficient (se) [95%CI] p value
HAH (hospital)	0.87 (0.03) [0.81;0.94] 0.001	0.77 (0.03) [0.71;0.84] 0	1.18 (0.05) [1.09;1.28] 0	0.81 (0.04) [0.74;0.9] 0	1.23 (0.09) [1.07;1.42] 0.004	1.07 (0.09) [0.91;1.25] 0.399
Admission date	1 (0) [1;1] 0.071	1 (0) [1;1] 0.048	1 (0) [1;1] 0.489	1 (0) [1;1] 0.3	1 (0) [1;1] 0.007	1 (0) [1;1] 0.012
ICD10 primary	1 (0) [1;1] 0.649	1 (0) [1;1] 0.671	1 (0) [1;1] 0.001	1 (0) [1;1] 0.001	1 (0) [1;1] 0.167	1 (0) [1;1] 0.16
ICD10 secondary	1 (0) [1;1] 0.588	1 (0) [1;1] 0.701	1 (0) [1;1] 0.148	1 (0) [1;1] 0.145	1 (0) [1;1] 0.875	1 (0) [1;1] 0.909
2yrs pre AE costs	1 (0) [1;1] 0.223	1 (0) [1;1] 0.261	1 (0) [1;1] 0.687	1 (0) [1;1] 0.561	1 (0) [1;1] 0.307	1 (0) [1;1] 0.267
2yrs pre elective costs	1 (0) [1;1] 0.909	1 (0) [1;1] 0.904	1 (0) [1;1] 0.537	1 (0) [1;1] 0.657	1 (0) [1;1] 0.896	1 (0) [1;1] 0.813
2yrs pre non-elective costs	1 (0) [1;1] 0	1 (0) [1;1] 0	1 (0) [1;1] 0.919	1 (0) [1;1] 0.458	1 (0) [1;1] 0.015	1 (0) [1;1] 0.021
2yrs pre day case costs	1 (0) [1;1] 0.099	1 (0) [1;1] 0.097	1 (0) [1;1] 0.006	1 (0) [1;1] 0.004	1 (0) [1;1] 0.131	1 (0) [1;1] 0.148
2yrs pre geriatric ward costs	1 (0) [1;1] 0.006	1 (0) [1;1] 0.005	1 (0) [1;1] 0.002	1 (0) [1;1] 0	1 (0) [1;1] 0.562	1 (0) [1;1] 0.713
2yrs pre mental ward costs	1 (0) [1;1] 0.905	1 (0) [1;1] 0.854	1 (0) [1;1] 0.005	1 (0) [1;1] 0.02	1 (0) [1;1] 0.09	1 (0) [1;1] 0.132
2yrs pre outpatient costs	1 (0) [1;1] 0.086	1 (0) [1;1] 0.088	1 (0) [1;1] 0.027	1 (0) [1;1] 0.026	1 (0) [1;1] 0.699	1 (0) [1;1] 0.675
2yrs pre medication costs	1 (0) [1;1] 0.713	1 (0) [1;1] 0.892	1 (0) [1;1] 0.136	1 (0) [1;1] 0.236	1 (0) [1;1] 0.713	1 (0) [1;1] 0.663
Died within 6months	1.03 (0.04) [0.95;1.11] 0.492	1.02 (0.04) [0.94;1.12] 0.572	1.05 (0.04) [0.97;1.14] 0.252	1.05 (0.05) [0.95;1.16] 0.38	1.06 (0.08) [0.91;1.23] 0.474	1.06 (0.09) [0.89;1.25] 0.517
Number of LTCs	1.08 (0.02) [1.05;1.11] 0	1.09 (0.02) [1.05;1.13] 0	1.04 (0.02) [1;1.07] 0.033	1.04 (0.02) [0.99;1.08] 0.093	1.06 (0.02) [1.01;1.1] 0.016	1.06 (0.03) [1.01;1.11] 0.019
Age on admission	1 (0) [1;1.01] 0.323	1 (0) [1;1.01] 0.452	1 (0) [1;1.01] 0.788	1 (0) [0.99;1.01] 0.789	1.01 (0.01) [1;1.02] 0.037	1.01 (0.01) [1;1.02] 0.055
Male	1.09 (0.04) [1.01;1.18] 0.035	1.1 (0.05) [1.01;1.2] 0.034	0.96 (0.04) [0.88;1.04] 0.311	0.95 (0.06) [0.85;1.07] 0.382	0.97 (0.07) [0.84;1.12] 0.686	0.97 (0.08) [0.82;1.14] 0.704
SES	1 (0.01) [0.98;1.02] 0.979	1 (0.01) [0.98;1.02] 0.954	1.01 (0.01) [1;1.02] 0.17	1.01 (0.01) [0.99;1.03] 0.205	1 (0.01) [0.97;1.03] 0.887	1 (0.02) [0.97;1.03] 0.917
Atrial Fibrillation	- - - - - - - - - - - -	- - - - - - - - - - - -	1.08 (0.05) [0.98;1.18] 0.104	1.1 (0.06) [0.98;1.23] 0.094	- - - - - - - - - - - -	- - - - - - - - - - - -
Arthritis	0.96 (0.04) [0.89;1.05] 0.392	0.96 (0.05) [0.88;1.05] 0.403	- - - - - - - - - - - -	- - - - - - - - - - - -	- - - - - - - - - - - -	- - - - - - - - - - - -
Cancer	- - - - - - - - - - - -	- - - - - - - - - - - -	1.04 (0.05) [0.95;1.14] 0.426	1.03 (0.06) [0.92;1.16] 0.566	- - - - - - - - - - - -	- - - - - - - - - - - -
CVD	1.02 (0.05) [0.92;1.13] 0.743	1.02 (0.06) [0.91;1.14] 0.794	1.07 (0.05) [0.98;1.18] 0.146	1.08 (0.07) [0.96;1.22] 0.199	1.09 (0.11) [0.91;1.32] 0.352	1.11 (0.12) [0.9;1.37] 0.324
Liver disease	1.21 (0.13) [0.98;1.48] 0.073	1.23 (0.14) [0.98;1.53] 0.074	- - - - - - - - - - - -	- - - - - - - - - - - -	- - - - - - - - - - - -	- - - - - - - - - - - -
Dementia	1.07 (0.05) [0.97;1.17] 0.16	1.07 (0.05) [0.97;1.18] 0.2	1.02 (0.05) [0.93;1.11] 0.738	0.99 (0.06) [0.88;1.1] 0.795	1.14 (0.11) [0.95;1.37] 0.153	1.14 (0.12) [0.94;1.4] 0.18
CHD	0.86 (0.05) [0.77;0.95] 0.004	0.85 (0.05) [0.76;0.95] 0.005	1.07 (0.05) [0.97;1.18] 0.174	1.02 (0.06) [0.9;1.15] 0.785	- - - - - - - - - - - -	- - - - - - - - - - - -
Epilepsy	- - - - - - - - - - - -	- - - - - - - - - - - -	1.04 (0.1) [0.86;1.26] 0.664	1.02 (0.12) [0.82;1.28] 0.841	- - - - - - - - - - - -	- - - - - - - - - - - -
Heart Failure	1.09 (0.05) [0.99;1.2] 0.095	1.09 (0.06) [0.98;1.21] 0.11	1.07 (0.06) [0.96;1.19] 0.201	1.09 (0.07) [0.96;1.24] 0.177	1.01 (0.1) [0.83;1.22] 0.947	1.02 (0.11) [0.82;1.25] 0.885
Multiple sclerosis	- - - - - - - - - - - -	- - - - - - - - - - - -	0.76 (0.1) [0.59;0.98] 0.033	0.73 (0.11) [0.54;0.99] 0.046	- - - - - - - - - - - -	- - - - - - - - - - - -
Parkinson's	1.23 (0.11) [1.04;1.45] 0.018	1.24 (0.12) [1.03;1.49] 0.021	1.07 (0.14) [0.84;1.37] 0.582	1.11 (0.18) [0.81;1.52] 0.512	- - - - - - - - - - - -	- - - - - - - - - - - -
Renal Failure	1.04 (0.05) [0.95;1.13] 0.436	1.03 (0.05) [0.93;1.13] 0.601	1.04 (0.05) [0.94;1.15] 0.408	1.06 (0.07) [0.94;1.2] 0.366	1.11 (0.11) [0.91;1.36] 0.3	1.12 (0.13) [0.9;1.39] 0.317
Diseases of blood	1.05 (0.05) [0.97;1.14] 0.246	1.05 (0.05) [0.96;1.15] 0.308	- - - - - - - - - - - -	- - - - - - - - - - - -	- - - - - - - - - - - -	- - - - - - - - - - - -
Diabetes	- - - - - - - - - - - -	- - - - - - - - - - - -	- - - - - - - - - - - -	- - - - - - - - - - - -	1.2 (0.11) [1;1.42] 0.044	1.22 (0.12) [1.01;1.48] 0.042
Constant	26.62 (74.48) [0.11;6410.63] 0.241	8.84 (27.52) [0.02;3945.99] 0.484	295178.8 (1199605) [102.52;850000000] 0.002	1223534 (6192074) [60.23;24900000000] 0.006	14800000000000 (127000000000000) [776224.7;2.84E+20] 0	31000000000(2920000000) [292677.5;3.28E+21] 0.001

Appendix 4 Estimated E-values

Site one

Total costs during follow-up period E-value for point estimate: 1.73 and for confidence interval: 1.49;

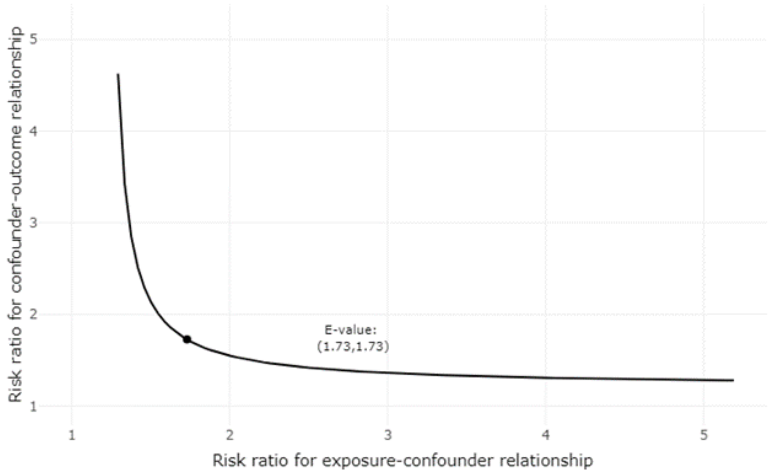

Total costs in 6 months after discharge E-value for point estimate: 1.86 and for confidence interval: 1.55;

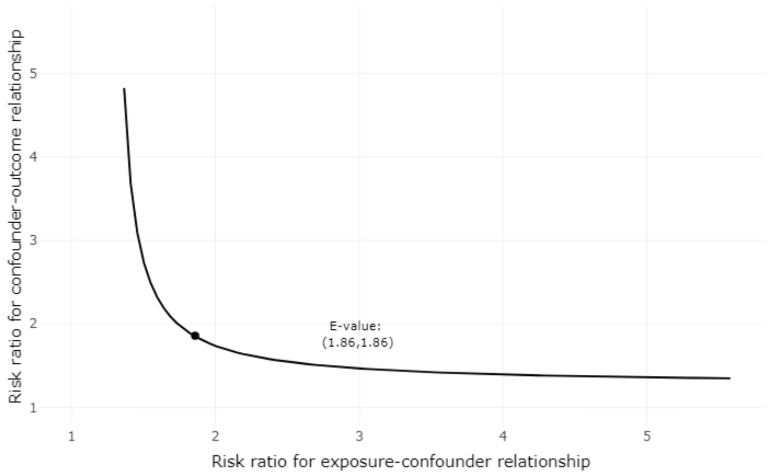

Mortality rate during follow-up: E-value for point estimate: 1.42 and for confidence interval: 1.04;

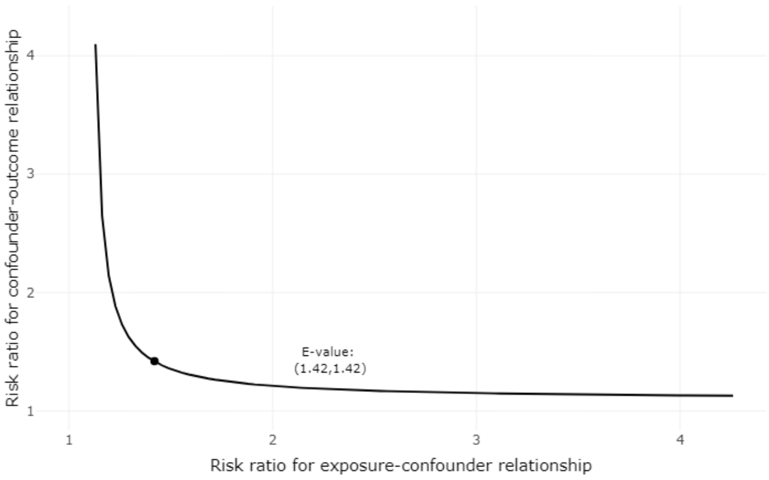

Site two

Total costs during follow-up period: E-value for point estimate: 1.03 and for confidence interval: 1

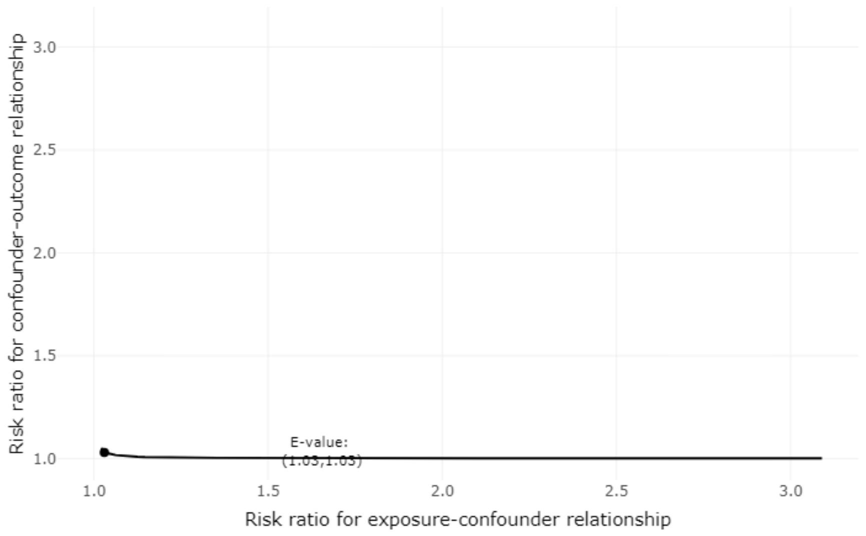

Total costs in 6 months after discharge: E-value for point estimate: 1.4 and for confidence interval: 1

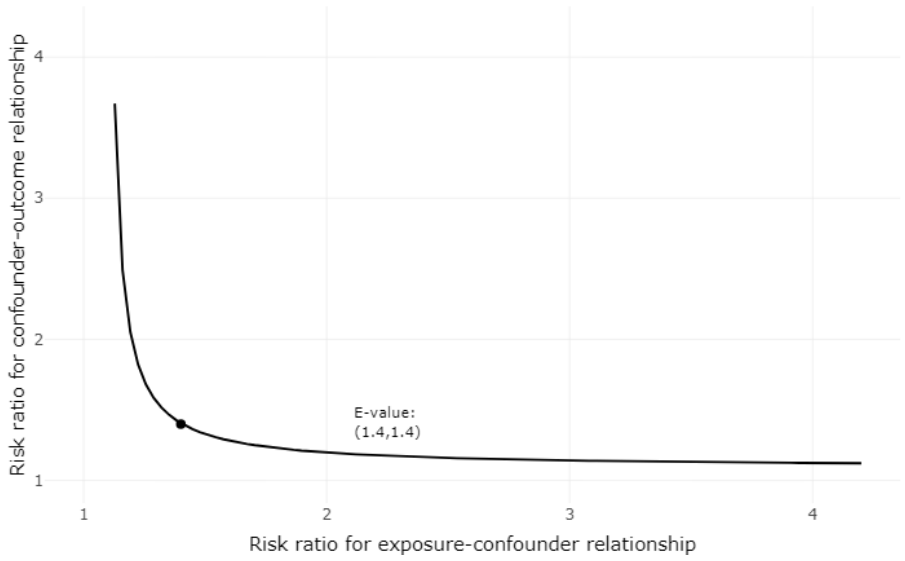

Mortality rate during follow-up: E-value for point estimate: 1.9 and for confidence interval: 1.57

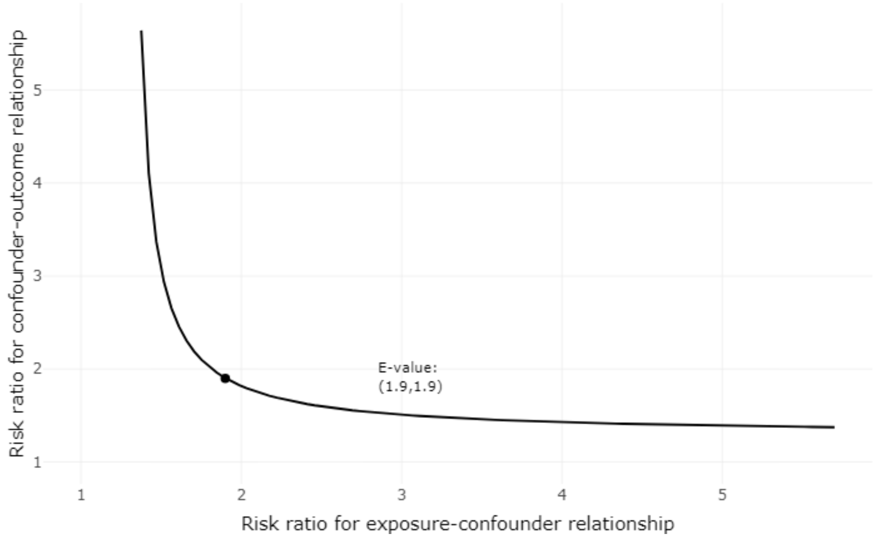

Site three

Total costs during follow-up period: E-value for point estimate: 1.57 and for confidence interval: 1

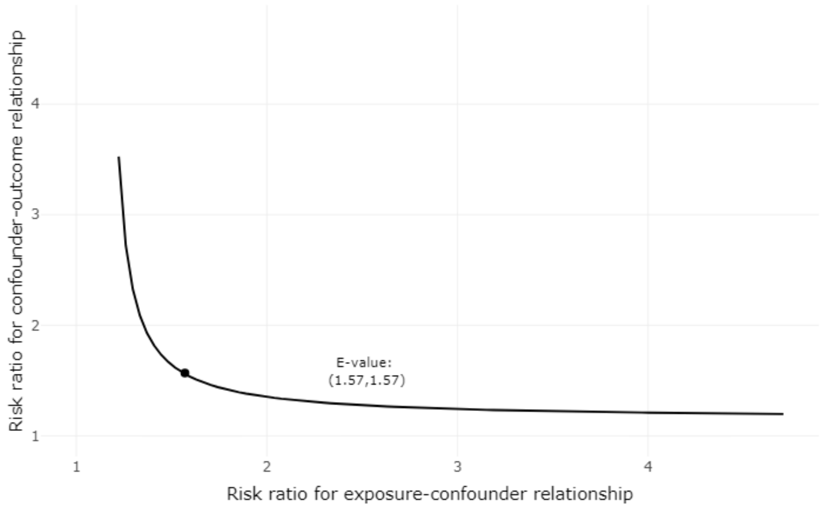

Total costs in 6 months after discharge: E-value for point estimate: 2.79 and for confidence interval: 2.15

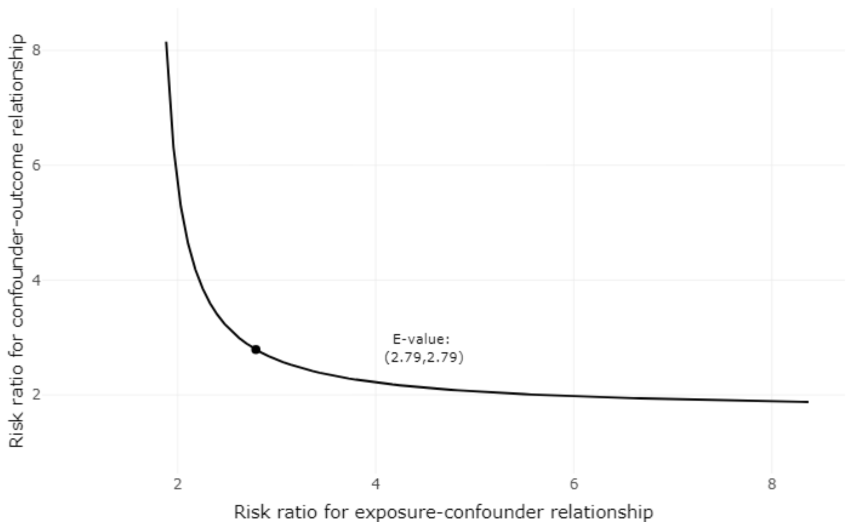

Mortality rate during follow-up: E-value for point estimate: 1.86 and for confidence interval: 1.31

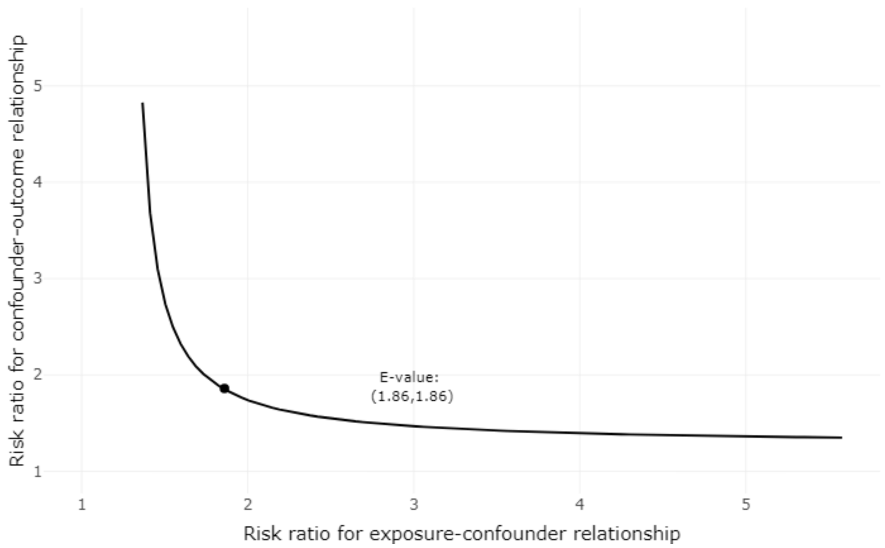

Supplement: Supplementary data [file bmjopen-2018-023350supp001.pdf]
