# Supplementary material for: Should I stay or should I go? A retrospective propensity score-matched analysis using administrative data of hospital-at-home for older people in Scotland
Source: BMJ Open. 2019 May 9;9(5):e023350. doi: 10.1136/bmjopen-2018-023350 (PMC6527981; doi:10.1136/bmjopen-2018-023350)
Supplement: Author's manuscript [file bmjopen-2018-023350.draft_revisions.pdf]

BMJ Open is committed to open peer review. As part of this commitment we make the peer review history of every article we publish publicly available.

When an article is published we post the peer reviewers' comments and the authors' responses online. We also post the versions of the paper that were used during peer review. These are the versions that the peer review comments apply to.

The versions of the paper that follow are the versions that were submitted during the peer review process. They are not the versions of record or the final published versions. They should not be cited or distributed as the published version of this manuscript.

BMJ Open is an open access journal and the full, final, typeset and author-corrected version of record of the manuscript is available on our site with no access controls, subscription charges or pay-per-view fees (http://bmjopen.bmj.com).

If you have any questions on BMJ Open's open peer review process please email info.bmjopen@bmj.com

BMJ Open

# Should I stay or should I go? A retrospective propensity score matched analysis using administrative data of hospital-at-home for older people

| | |
|---|---|
| Journal: | *BMJ Open* |
| Manuscript ID | bmjopen-2018-023350 |
| Article Type: | Research |
| Date Submitted by the Author: | 04-Apr-2018 |
| Complete List of Authors: | Tsiachristas, Apostolos; University of Oxford, Health Economics Research Centre, Nuffield Department of Population Health<br>Ellis, Graham; Monklands Hospital, NHS Lanarkshire<br>Buchanan, Scott; Information Services Division, National Services Scotland<br>Langhorne, Peter; University of Glasgow, Institute of Cardiovascular and Medical Sciences<br>Stott, David; Institute of Cardiovascular and Medical Sciences, University of Glasgow, Geriatric Medicine<br>Shepperd, S; University of Oxford, Nuffield Department of Population Health |
| Keywords: | hospital-at-home, admission avoidance, intermediate care, costs, mortality, UK |
| | |

SCHOLARONE™
Manuscripts

# Should I stay or should I go? A retrospective propensity score matched analysis using administrative data of hospital-at-home for older people

Apostolos Tsiachristas[1], Graham Ellis[2], Scott Buchanan[3], Peter Langhorne[4], David Stott[4], Sasha Shepperd[1].

[1] Nuffield Department of Population Health, University of Oxford, Oxford, UK

[2] Monklands Hospital, NHS Lanarkshire, Glasgow, UK

[3] Information Services Division, National Services Scotland, Edinburgh, UK

[4] Institute of Cardiovascular and Medical Sciences, University of Glasgow, Glasgow, UK

Word Count: 4783

Correspondence to: Apostolos Tsiachristas, Health Economics Research Centre, Nuffield Department of Population Health, Richard Doll building, Old Road Campus, OX3 7LF, Oxford, UK; Tel: +44(0)1865 289470; email: apostolos.tsiachristas@dph.ox.ac.uk



For peer review only - http://bmjopen.bmj.com/site/about/guidelines.xhtml

# Abstract

Objectives: To compare the characteristics of populations admitted to hospital-at-home services with the population admitted to hospital and assess the association of these services with healthcare costs and mortality.

Design: In a retrospective observational cohort study of patient level data we used propensity score matching in combination with regression analysis.

Participants: Patients aged 65 years and older who were admitted with similar diagnoses to either hospital-at-home or hospital at the same period.

Interventions: Three geriatrician-led admission avoidance hospital-at-home services.

Main outcome measures: Healthcare costs and mortality.

Results: Patients in hospital-at-home were older and more socioeconomically disadvantaged, had higher rates of previous hospitalization, and there was a greater proportion of women and people with several chronic conditions compared with the population admitted to hospital. The cost of providing hospital-at-home varied between the three sites from £628 to £2928 per admission. Hospital-at-home was associated to 18% lower costs during the follow-up period in site one (ratio of means 0.82; 95%CI: 0.76 to 0.89). Limiting the analysis to costs during the 6 months following index discharge, patients in the hospital-at-home cohorts had 27% higher costs (ratio of means 1.27; 95%CI: 1.14 to 1.41) in site one, 9% (ratio of means 1.09; 95%CI: 0.95 to 1.24) and 70% in site three (ratio of means 1.70; 95%CI: 1.40 to 2.07) compared with patients in the control cohorts. Admission to hospital-at-home was associated with an increased risk of death during the follow-up period in all three sites (1.09, 95%CI: 1.00 to1.19 site one; 1.29, 95%CI 1.15 to 1.44 site two; 1.27, 95%CI 1.06 to1.54 site three).

Conclusions: It is important to identify robust measures for admission to hospital-at-home and collect data on subsequent use of health, social, and informal care following admission to hospital-at-home or hospital to be used in a clinical trial.




For peer review only - http://bmjopen.bmj.com/site/about/guidelines.xhtml

**Strengths and limitations of the study**

- The study used a large dataset from three of the largest Health Boards in Scotland.
- The quasi-experimental study design has allowed inferences from real world evidence.
- Various sensitivity analyses helped to address uncertainty in the results.
- The major limitation of this type of non-randomised comparison is residual confounding.
- The data lacked of quality of life measurements as well as use of community and informal care.

For peer review only



For peer review only - http://bmjopen.bmj.com/site/about/guidelines.xhtml

# Introduction

Organising health systems to optimise the health outcomes of older people and contain costs is a priority as populations around the world age, and the demand for healthcare continues to rise. Despite a global policy emphasis on 'care closer to home'[1] and initiatives that seek to ease demand for hospital based healthcare, efforts to innovate and deliver healthcare services that provide an alternative to hospital admission for older people have been piecemeal and often lack a health system perspective. A lack of evidence to support decision-making has contributed to this. Avoiding admission to hospital by providing acute healthcare in people's homes, often as a hospital outreach service, is one of the more popular service innovations and yet there is uncertainty around the effectiveness and cost-effectiveness of this form of care.[2]

The use of administrative data to evaluate service delivery interventions has the potential to provide a simple and efficient mechanism to provide real-world evidence about policy relevant service innovations, and embed evaluation into local decision-making. However, previous experience of using routine data in this area of research has been of mixed success due to a limited set of variables, missing data and the complexity of policy relevant questions that often require a broad and longer term perspective.[3] Administrative healthcare data collected in Scotland is unique in that it is population based, with little missing data. The aim of this study was to use these data to compare the characteristics of populations from three Health Boards who used a geriatrician-led hospital-at-home service with the population who received hospital care, and to assess the impact of these services on healthcare costs and mortality.

**Box 1 Description of each service**

Hospital-at-home

The three hospital-at-home services are broadly similar, capacity ranged between 24 to 60 beds for the period of the analysis. Each is a geriatrician-led service that is supported by nurses (sometimes nurse practitioners) and therapy practitioners for the initial assessment; geriatricians and the multi-disciplinary team review patients in their homes and meet daily (a virtual ward round) to discuss patient cases and agree actions. Rehabilitation is available within the existing team with onward referral to community rehabilitation as required, and in one site rehabilitation is accessed through a parallel community rehabilitation services. Out of hours emergency cover is provided by primary care out-of-hours. Patients are referred to the service from GPs, sometimes through a central referral number or via step down from the acute hospital. The service offers access to diagnostics such as radiology, and intravenous fluids, antibiotics and oxygen. Cases are discussed daily with the multidisciplinary team at the virtual ward round and daily management plans agreed. In one site there is close working with the day hospital where patients can be referred for follow up or for investigations. Patients access investigations and treatment with the same speed as inpatients. The services support intravenous therapies in the home.

Hospital

The provision of hospital based acute health services varied among the sites; in one site there were three district general hospitals (1,653 beds) that provide acute health services to a mainly urban population of 652,230, with a total of 1,653 beds; in site two a hospital (550 beds) provides acute healthcare to a population of 180,130; and in site three there are two district general hospitals (825 beds) that provide healthcare to a population of 358,900, and acute admissions are via one of the hospitals.



For peer review only - http://bmjopen.bmj.com/site/about/guidelines.xhtml

# Methods

### *Setting*

We used patient level data collected by three of the fourteen Scottish Health Boards of all patients aged 64 years and older, and who were admitted (referred to as the index admission) to either geriatrician-led admission avoidance hospital-at-home or inpatient hospital between August 2014 and December 2015 (17 months) in site one and site two, and between January 2015 and December 2016 (24 months) in site three. These services are commissioned by integrated health and social care boards that cover a population of almost 1.5 million in urban and rural areas. The Information Service Division (ISD), part of NHS Scotland, de-identified, cleaned and linked individual patient records to derive activity and costs related to periods before and after the index admissions. We obtained signed release forms from each Board's Caldicott guardian, and followed the ISD data sharing agreement.

### *Intervention*

The three service models of hospital-at-home provided an admission avoidance function that provided an alternative to inpatient hospital care, and had similar structures and functions; the main differences were in the capacity of the services and the organisation of services for rehabilitation. (Box 1)

### *Data sources*

Data were available for each person for two years prior to their index admission, and from the point of their index admission to six months after index discharge from hospital-at-home or hospital. Box 2 presents a full list of all variables included in the dataset. Figure 1 provides schematic examples of the differing calendar time periods studied before and after index admission for people

Box 2. List of variables included in the dataset

Costs of accidents and emergency attendances,
Costs of acute day cases,
Costs of acute elective hospitalisation,
Costs of acute non-elective hospitalisation,
Costs of geriatric wards,
Costs of mental health wards,
Costs of outpatient visits,
Costs of prescribed medication,
Costs of (re)admission to hospital-at-home.
Primary ICD-10 codes on index discharge,
Secondary ICD-10 codes on index discharge,
Length of stay of the index admission,
Age on index admission,
Gender,
Scottish Index of Multiple Deprivation (SIMD), 1 (most deprived) to 10 (most affluent)
Long-term conditions,
Date of death (if applicable),
Based on ICD-10 codes:
Cardiovascular disease (CVD) (I60-I69, G45)
Chronic obstructive pulmonary disorder (COPD) (J41-J44, J47),
Dementia (F00-F03, F05.1),
Diabetes (E10-E14),
Coronary heart disease (CHD, ICD10: I20-I25),
Heart failure (I500, I501, I509),
Renal failure (N03, N18, N19, I12, I13),
Epilepsy (G40, G41),
Asthma (J45, J46),
Atrial fibrillation (I48, MS, G35),
Cancer (C00-C97),
Arthritis (M05, M19, M45, M47, M460-M462, M464, M468, M469),
Parkinson's (G20-G22),
Chronic liver disease (K711, K713, K714, K717, K754),
Congenital problems (Q00-Q99),
Diseases of blood and blood forming organs (D50-D89),
Other diseases of the digestive system (K00-K122, K130-K839, K85X, K860-K93),
Other endocrine metabolic diseases (E00-E07, E15-E35, E70-E90)
Admitted to HAH or hospital.



For peer review only - http://bmjopen.bmj.com/site/about/guidelines.xhtml

admitted between August 2014 and December 2015 to hospital-at-home (Patients A and B) or hospital (Patients C and D) in site one. As this illustrates, the maximum follow-up period for each patient consisted of the period between index admission and index discharge and 6 months after index discharge. The data were collected via the data systems used in hospitals to collect patient data. Hospital-at-home activity data is submitted to ISD from the local systems of the three sites. The following data sets are included acute inpatient, geriatric long stay and day case, mental health admissions, outpatient appointments accident and emergency attendances, community prescribing and NRS death registrations.

Figure 1. Illustration of obtained data from site one

*Selection of patients in the hospital-at-home and control cohorts*

We included patients aged 65 years and older, and who were classified as an unscheduled admission to general or geriatric medicine. In the control cohort, we excluded those with a diagnosis that would not be eligible for management through hospital-at-home; these exclusions included acute intracerebral crisis (intracerebral infections, trauma or haemorrhage), stroke and related codes, acute coronary syndromes and myocardial infarction, surgical emergencies including vascular, urological, gynaecological and general surgical presentations, orthopaedic diagnosis of fractures and trauma, cardiothoracic diagnoses, poisoning and complications of surgery. We also excluded from the control group those who had a diagnosis (i.e. primary and secondary ICD-10 code) that was not observed in any of the hospital-at-home admissions in each site (1081 patients in site one, 1405 in site two and in 451 in site three) (Figure 2). Each patient was counted as a single episode of healthcare.

*Intervention costs*

We collected data on the costs of hospital-at-home using a template derived from the Cost-It tool of the World Health Organisation.[4] The cost categories included staff, training, transport, information and communication, clinical materials/equipment, support services, laboratory services, diagnostics, overheads and other costs. Clinician managers supported by finance staff in the three Health Boards completed this template based on the actual spending for the hospital-at-home service for the time periods covered by the ISD data. The cost per hospital-at-home admission was calculated by dividing the total costs of the hospital-at-home service by the total number of hospital-at-home admissions during the same period.



For peer review only - http://bmjopen.bmj.com/site/about/guidelines.xhtml

*Statistical analysis*

We used an iterative approach to the analysis, starting with a description of the two cohorts (i.e. those admitted to hospital-at-home and those admitted to hospital) for each Health Board. We calculated means, standard errors, and frequencies to describe differences in patient characteristics at index admission and tested differences using Mann-Whitney test for continues variables and Chi-square test for categorical variables. We also estimated the mean differences in resource utilisation costs (with bootstrapped standard errors) and the unadjusted relative risk of mortality between the two cohorts for each Health Board.

Further, we investigated the association of being admitted to hospital-at-home or hospital with mortality and cost over a minimum follow-up period of six months. To do this, we followed the Medical Research Council guidelines on performing natural experiments and scientific literature to adopt a step-wise strategy to select the propensity score matching (PSM) technique that most reduced observed confounding between the two cohorts in each Health Board.[5-8] First, we included all possible confounding variables available in the dataset (see Box 2 and Figure 2), and considered that the inclusion of covariates not associated with the treatment assignment would have little influence in the propensity score model.[5] Second, we matched the two cohorts in each site using a range of the most commonly used PSM techniques; these included Mahalanobis, 1-to-1, K-to-1, kernel, local linear regression, spline, and inverse probability weighting techniques. Second, the performance of each PSM technique on covariate balancing was assessed based on the mean and median percentage standardised bias as well as Rubin's B (the absolute standardized difference of the means of the linear index of the propensity score in the treated and (matched) non-treated group) and Rubin's R (the ratio of treated to (matched) non-treated variances of the propensity score index). Following Rubin's (2001) recommendation, we considered B less than 25 and R between 0.5 and 2 to indicate sufficient balance.[9] Third, we chose the PSM technique that had the lowest values on these performance indicators in each of the three Health Boards. We matched the two cohorts in each Health Board by socio-demographic characteristics (i.e. age, gender, socio-economic status), diagnosis code (i.e. primary and secondary ICD-10 code) of index admission, morbidity (i.e. type of long-term condition, mortality during follow-up (for the analysis of cost), 2-year costs prior to the index admission (by cost category as listed in Box 1), and date of index admission (to account for seasonal trends).

We performed a doubly robust estimation to further reduce confounding by using a regression analysis after performing the most suitable PSM technique and including the confounding variables listed above as covariates.[10] In the regression, we used generalised linear regression models (GLMs) with gamma distribution and log link to investigate the association of hospital-at-home with total



For peer review only - http://bmjopen.bmj.com/site/about/guidelines.xhtml

costs during the follow-up period, and total costs in 6 months following index discharge. We also used GLMs with Poisson distribution and log link to estimate the relative risk of mortality. Robust standard errors were specified in all regression models. We calculated Kaplan-Meier survival curves, with and without using the weights from the PSM, and used log-rank tests to test the equality of the survival functions. There were few missing observations in the dataset and thus, complete case analysis was performed.

*Subgroup analysis*

We conducted a sub-group analysis, running the same regression models used in the main analysis, to investigate the association of hospital-at-home services with costs and mortality for the population who had a diagnosis of dementia. We considered this population to be important due to their complex healthcare needs, and the increasing prevalence of dementia.[11 12] In a second subgroup analysis, we excluded patients who died during the follow-up period and investigated the association of hospital-at-home with costs. In both subgroup analyses, propensity score matching was performed to match sub-cohorts in each site.

*Sensitivity analysis*

In a univariate sensitivity analysis, we reduced and increased the intervention cost of admission avoidance hospital-at-home by 50%, as there are no standard unit costs to benchmark these types of services and we were concerned that costs for these services may vary due to economies of scale, size, experience, setting, human resource capacity, and error. This sensitivity analysis was expected to impact the costs during index admission and the costs of admission to hospital-at-home in the six months after discharge.

*Patient involvement*

Patients were not involved in this retrospective analysis of administrative data.

## Results

*Characteristics of the population cohorts*

Between August 2014 and December 2015 (17 months) 1771 patients were admitted to hospital-at-home in site one, between January 2015 and December 2016 (24 months) 1547 patient were admitted to hospital-at-home in site two, and between August 2014 and December 2015 (17 months) 443 patients were admitted to hospital-at-home in site three (Figure 2). In the same period, there were 14220 patients admitted to 3 hospitals in site one, 5399 patients admitted to 1 hospital in site two, and 2295 patients admitted to 1 hospital in site three.



For peer review only - http://bmjopen.bmj.com/site/about/guidelines.xhtml

There were few differences between the hospital-at-home cohorts in site one, site two, and site three, the main difference being that a larger proportion of the population in site two lived in a more affluent area (i.e. scored five or higher on the SIMD). Patients admitted to hospital-at-home were on average three to four years older than those admitted to hospital, were more likely to be female (ranging from 5 percentage points in site three to 9 percentage points in site two), and a higher proportion had more than four long-term conditions (approximately 7 percentage points) compared with patients admitted to hospital (Table 1). The largest difference between those admitted to hospital-at-home and to hospital in site one and site two was in the proportion of patients with dementia (10 percentage points higher in the hospital-at-home cohorts), while in site three it was the proportion of patients with renal failure (also 10 percentage points higher in the hospital-at-home cohort).

We compared the two cohorts in each site, from index admission to six months post discharge from hospital-at-home or hospital (Table 2). In all sites there was on average a higher percentage of deaths while receiving healthcare in hospital compared with those receiving healthcare in hospital-at-home (6% vs., 1% site one; 6% vs., 3% site two; 4% vs., 1% site three), and a higher percentage of deaths (21% vs., 28% site one; 22% vs., 32% site two; 17% vs., 27% site three) during the whole follow-up period (i.e. during admission and six months after discharge) in the group that had received hospital-at-home. Patients in the hospital-at-home cohort lived on average eight (site one), ten (site two), and twelve (site three) fewer days during the whole follow-up, and their index admission was on average fewer days in site one (mean unadjusted difference -2.64, 95%CI -2.97 to -2.31) and site three (mean unadjusted difference -2.02, 95%CI -2.66 to -1.37) and longer in site two (mean unadjusted difference 1.25, 95% CI 0.86 to 1.64).

The cost during a hospital-at-home admission was on average lower than hospital admission in site one (mean difference -£2318; 95%CI: £-2420 to £-2217) and site three (mean difference -£1096; 95%CI: £-1398 to £-793), and slightly lower (mean difference £-153; 95%CI: £-277; to £-29) in site two. In the hospital-at-home cohort, these costs included the intervention costs of delivering the service at home, which were £628 per admission and £113 per day in site one, £2928 per admission and £398 per day in site two, and £864.54 per admission and £117.57 per day in site three. In each Health Board, staff were the major driver of intervention (i.e. hospital-at-home) cost (site one 95%, site two 87%, site three 94%). Detailed information on the interventions costs in site one, site two, and site three are presented in Appendix 1.

In site one, in the two years prior to the index admission, the hospital-at-home cohort had on average 40% (mean difference £3219; 95%CI: £2513 to £3925) more healthcare costs, driven primarily by higher costs of non-elective hospitalisation. We observed a similar pattern in site two



For peer review only - http://bmjopen.bmj.com/site/about/guidelines.xhtml

and site three where the mean costs in the hospital-at-home-cohort were on average 56% higher (mean difference £5064; 95%CI: £3984 to £6143) and 57% (mean difference £4115; 95%CI: £2467 to £5764) respectively and again were due to non-elective hospitalisation. In the six months following discharge, and excluding the costs of the index admission, costs were on average 43% higher (mean difference £1839; 95%CI: £1423 to £2255) in site one for those who had been admitted to hospital-at-home, in site two they were 16% higher (mean difference £875, 95%CI: £156 to £1595), and in site three they were 92% higher (mean difference £3068, 95%CI: £2178 to £3958). The larger increase in costs in all sites was due to higher non-elective hospitalisation costs in the group who had received hospital-at-home care (mean difference £1517, 95%CI £1134 to 1899 site one; mean difference £529, 95%CI £-77 to 1135 site two; mean difference £2618, 95%CI £1779 to 3458 site three) during the six months follow-up.

When the cost of the index admission was included in the analysis, the cost during follow-up (i.e. including the index admission and 6-months healthcare resource use after index discharge) was 6% lower (mean difference -£480, 95%CI: £-996 to £36) in the hospital-at-home cohort, compared with the control cohort in site one; while these costs were 8% higher in site two (mean difference £722, 95%CI: £32 to £1413) and 35% higher in site three (mean difference £1973, 95%CI: £1019 to £2927).

Compared with the control cohort, the mean costs per lived day were 13% (mean difference £-12; 95%CI: -17 to -6) lower in the hospital-at-home cohort in site one, while these costs were 34% higher (mean difference £37; 95%CI: 18 to 56) and 66% higher (mean difference £36; 95%CI: 18 to 53) in site two and site three respectively.

Figure 2 Flowchart of study population



For peer review only - http://bmjopen.bmj.com/site/about/guidelines.xhtml

Table 1 Patient characteristics at index admission

| | Site one | | Site two | | Site three | |
|---|---|---|---|---|---|---|
| *Variable* | *Control (n=13139)* | *HAH (n=1737)* | *Control (n=3994)* | *HAH (n=1463)* | *Control (n=1844)* | *HAH (n=433)* |
| Mean age on admission (se) | 77.8 (0.07) | 81.2 (0.17)** | 78.5 (0.13) | 82.2 (0.21)** | 77.3 (0.18) | 81.4 (0.34)** |
| Female | 7,468 (57%) | 1,096 (63%)** | 2,102 (53%) | 909 (62%)** | 1037 (56%) | 266 (61%)* |
| Higher than 4 on the SIMD | 5,005 (38%) | 609 (35%)** | 1,960 (49%) | 775 (53%)* | 837 (45%) | 192 (44%) |
| More than 4 chronic conditions | 4,974 (38%) | 777 (45%)** | 1,664 (42%) | 725 (50%)** | 659 (36%) | 185 (43%)** |
| Arthritis | 3,431 (26%) | 497 (29%)* | 1,455 (37%) | 572 (39%) | 606 (33%) | 155 (36%) |
| Asthma | 1,370 (10%) | 183 (11%) | 497 (13%) | 207 (14%) | 177 (10%) | 49 (11%) |
| Atrial fibrillation | 3,659 (28%) | 488 (28%) | 1,555 (29%) | 468 (32%)* | 498 (27%) | 126 (29%) |
| Cancer | 3,749 (29%) | 485 (28%) | 1,261 (32%) | 371 (25%)** | 580 (31%) | 124 (29%) |
| CVD | 2,922 (22%) | 467 (27%)** | 763 (19%) | 392 (27%)** | 373 (20%) | 114 (26%)** |
| Liver disease | 499 (4%) | 50 (3%) | 183 (5%) | 52 (4%) | 72 (4%) | 20 (5%) |
| COPD | 3,641 (28%) | 505 (29%) | 1,083 (27%) | 428 (29%) | 510 (28%) | 132 (31%) |
| Dementia | 1,999 (15%) | 439 (25%)** | 665 (17%) | 390 (27%)** | 223 (12%) | 74 (17%)** |
| Diabetes | 2,985 (23%) | 403 (23%) | 948 (24%) | 350 (24%) | 410 (22%) | 115 (27%)* |
| Epilepsy | 459 (4%) | 75 (4%) | 146 (4%) | 78 (5%)** | 53 (3%) | 10 (2%) |
| CHD | 5,034 (38%) | 733 (42%)** | 1,425 (36%) | 575 (39%)* | 624 (34%) | 141 (33%) |
| Heart failure | 2,197 (17%) | 404 (23%)** | 744 (19%) | 32 (23%)** | 328 (18%) | 109 (25%)** |
| MS | 73 (1%) | 6 (0%) | 21 (1%) | 17 (1%)* | 14 (1%) | 2 (1%) |
| Parkinson’s | 293 (2%) | 66 (4%)** | 82 (2%) | 53 (4%)** | 53 (3%) | 20 (5%) |
| Renal failure | 2,501 (19%) | 394 (23%)** | 780 (20%) | 339 (23%)** | 284 (15%) | 110 (25%)** |
| Congenital problems | 277 (2%) | 38 (2%) | 159 (4%) | 51 (4%) | 51 (3%) | 9 (2%) |
| Diseases of blood | 3,784 (29%) | 553 (32%)** | 1,143 (29%) | 426 (29%) | 485 (26%) | 125 (29%) |
| Endocrine metabolic disease | 4,505 (34%) | 624 (36%) | 1,737 (44%) | 652 (45%) | 642 (35%) | 151 (35%) |
| Disease of digestive system | 9,341 (71%) | 1,249 (72%) | 2,710 (68%) | 1,006 (69%) | 1145 (62%) | 286 (66%) |

* p<0.05 ** p<0.01 in chi-square test for categorical and Mann-Whitney for continuous variables to test differences between HAH and control; HAH: hospital-at-home; SIMD ranges from 1 (most deprived) to 10 (most affluent); Note: a patient could be registered with more than one ICD-10 codes;

For peer review only



For peer review only - http://bmjopen.bmj.com/site/about/guidelines.xhtml

Table 2. Mortality, resource utilisation and costs

| | Site one | | | Site two | | | Site three | | |
|---|---|---|---|---|---|---|---|---|---|
| *Variable* | *Control (n=13139)* | *HAH (n=1737)* | *Mean difference or risk ratio (95%CI)* | *Control (n=3994)* | *HAH (n=1463)* | *Mean difference or risk ratio (95%CI)* | *Control (n=1844)* | *HAH (n=433)* | *Mean difference or risk ratio (95%CI)* |
| Died during index admission | 844 (6%) | 20 (1%) | 0.18 (0.12;0.28)[##] | 256 (6%) | 47 (3%) | 0.50 (0.37;0.68)[##] | 78 (4%) | 2 (1%) | 0.11 (0.03;0.44)[##] |
| Died during follow-up including index admission | 2,787 (21%) | 483 (28%) | 1.31 (1.21;1.42)[##] | 867 (22%) | 471 (32%) | 1.48 (1.35;1.63)[##] | 319 (17%) | 116 (27%) | 1.55 (1.29;1.86)[##] |
| Means days alive during follow-up (se) | 159 (0.50) | 151 (1.45) | -8.32 (-11.32;-5.32) | 156 (0.91) | 146 (1.72) | -10.10 (-14;-7) | 163 (1.22) | 151 (2.88) | -12 (-18;-6) |
| Mean length of index admission in days (se) | 8.18 (0.12) | 5.54 (0.13) | -2.64 (-2.97;-2.31) | 6.10 (0.14) | 7.35 (0.14) | 1.25 (0.86;1.64) | 6.36 (0.26) | 4.34 (0.20) | -2.02 (-2.66;-1.37) |
| Mean 2 year historical costs (se) | | | | | | | | | |
| A&E | 173 (2) | 253 (7) | 80 (65;94) | 136 (4) | 180 (6) | 44 (28;60) | 143 (5) | 202 (12) | 59 (31;87) |
| Elective hospital care | 985 (37) | 956 (134) | -28 (-352;295) | 1,027 (64) | 705 (86) | -321 (-519;-123) | 981 (87) | 1036 (372) | 55 (-723;833) |
| Non-elective hospital care | 4,037 (79) | 6,945 (266) | 2908 (2452;3364) | 5,101 (185) | 9,593 (394) | 4492 (3804;5179) | 3978 (211) | 7832 (614) | 3854 (2591;5118) |
| Hospital day case | 707 (25) | 439 (32) | -269 (-340;-197) | 625 (66) | 290 (44) | -336 (-479;-193) | 544 (49) | 358 (55) | -186 (-334;-38) |
| Geriatric long stay | 360 (27) | 504 (82) | 143 (-66;354) | 117 (29) | 252 (72) | 135 (-13;283) | 105 (31) | 229 (59) | 125 (14;235) |
| Mental ward | 247 (32) | 367 (117) | 119 (-177;411) | 347 (79) | 1,053 (205) | 706 (265;1147) | 220 (75) | 252 (139) | 32 (-329;393) |
| Outpatient | 173 (2) | 173 (5) | 0 (-11;11) | 222 (4) | 206 (6) | -15 (-30;0) | 212 (6) | 201 (12) | -11 (-38;15) |
| Medication (GP prescriptions) | 1,468 (15) | 1,733 (43) | 256 (187;341) | 1,524 (28) | 1,883 (52) | 360 (253;466) | 1034 (39) | 1221 (78) | 188 (30;346) |
| Total | 8,149 (109) | 11,369 (359) | 3219 (2513;3925) | 9,098 (239) | 14,162 (477) | 5064 (3984;6143) | 7217 (267) | 11333 (772) | 4115 (2467;5764) |
| Mean costs during index admission (se) | 3,195 (41) | 877[#] (32) | -2318 (-2420;-2217) | 3,426 (71) | 3,273[#] (32) | -153 (-277;-29) | 2383 (90) | 1287 (132) | -1096 (-1398;-793) |
| Mean costs 6 months after index discharge (se) | | | | | | | | | |
| A&E | 72 (1) | 88 (3) | 17 (11;22) | 55 (2) | 53 (3) | -2 (-9;4) | 59 (2) | 71 (5) | 12 (-1;25) |
| Elective hospital care | 305 (20) | 157 (40) | -148 (-236;-60) | 272 (28) | 204 (50) | -68 (-190;53) | 169 (33) | 313 (117) | 144 (-92;380) |
| Non-elective hospital care | 2,444 (51) | 3,961 (171) | 1517 (1134;1899) | 3,942 (130) | 4,471 (251) | 529 (-77;1135) | 2029 (123) | 4648 (421) | 2618 (1779;3458) |
| Hospital day case | 237 (11) | 73 (11) | -164 (-191;-138) | 234 (24) | 96 (21) | -139 (-198;-79) | 168 (23) | 63 (15) | -105 (-162;-48) |
| Geriatric long stay | 643 (45) | 1,014 (131) | 371 (79;663) | 218 (34) | 150 (46) | -68 (-178;41) | 320 (56) | 700 (186) | 381 (-73;834) |
| Mental ward | 165 (22) | 206 (51) | 41 (-58;140) | 299 (56) | 259 (77) | -40 (-224;143) | 211 (65) | 120 (62) | -91 (-245;64) |
| Outpatient | 54 (1) | 45 (2) | -9 (-13;-5) | 61 (2) | 54 (3) | -8 (-14;-2) | 65 (3) | 67 (6) | 2 (-12;16) |
| Medication (GP prescriptions) | 392 (5) | 415 (13) | 23 (-5;52) | 402 (9) | 482 (16) | 80 (45;115) | 314 (12) | 338 (27) | 24 (-28;76) |
| Hospital-at-home | 4 (1) | 196 (11) | 193 (170;216) | 50 (7) | 642 (45) | 592 (506;679) | 7 (1) | 90 (12) | 83 (59;108) |
| Total | 4,316 (78) | 6,155 (240) | 1839 (1423;2255) | 5,535 (154) | 6,410 (286) | 875 (156;1595) | 3342 (163) | 6410 (510) | 3068 (2178;3958) |
| Mean costs in follow-up (se) including index admission | 7,513 (92) | 7,031 (243) | -480 (-996;36) | 8,961 (180) | 9,683 (290) | 722 (32;1413) | 5724 (199) | 7697 (521) | 1973 (1019;2927) |
| Mean costs per lived day in follow-up (se) | 83 (1) | 72 (3) | -12 (-17;-6) | 109 (3) | 146 (8) | 37 (18;56) | 55 (2) | 91 (8) | 36 (18;53) |

# it includes the interventions costs (i.e. £628 in site one, £2,928 in site two, and £865.54 in site three) and other costs occurred during the episode; ## Unadjusted Risk Ratio;



For peer review only - http://bmjopen.bmj.com/site/about/guidelines.xhtml

For peer review only

*Selection of propensity score matching technique*

In the propensity score matched analysis, there were 1696, 925, and 427 patients in the hospital-at-home cohort and 11571, 3849, and 1683 patients in the hospital cohort in site one, site two, and site three respectively (Figure 2). Local linear regression matching was the best PSM technique to match the cohorts in site one and site three for costs and mortality, as it resulted in a lower mean (i.e. 1.5 and 1.8 respectively) and median (i.e. 1.2 and 1.6 respectively) percentage standardised bias, as well as the lowest Rubin's B (i.e. 9.4 and 9.6 respectively). Based on the same criteria, Kernell matching was selected to match the cohorts in site two. Rubin's R was within the suggested range (i.e. from 0.5 to 2) in the selected techniques. These results are presented in Appendix 2.

*Main propensity score matched analysis*

The results of the main analysis are presented in Panel A in Table 3. After propensity score matching and regression analysis, the healthcare cost during index admission in hospital-at-home and over six months after index discharge was on average 18% lower (ratio of means: 0.82; 95%CI: 0.76 to 0.89) than admission to hospital in site one. Excluding the cost of the index admission (hospital-at-home or hospital) the costs during the six months following discharge for those who had been admitted to hospital-at-home were on average 27% higher (ratio of means: 1.27; 95%CI: 1.14 to 1.41) compared with patients who had been admitted to hospital. In site two, the difference in costs between the cohorts was close to zero (ratio of means: 1.00; 95%CI 0.92 to 1.09) during the index admission and six month follow-up period; and 9% (ratio of means: 1.09; 95%CI: 0.95 to1.24) more costly in the six months after index discharge (i.e. excluding the index admission). In site three, patients admitted to hospital-at-home had on average 15% higher cost during the entire follow-up period (ratio of means: 1.15; 95%CI 0.99 to 1.33) and 70% higher cost during the six months after discharge (ratio of means: 1.70; 95%CI 1.40 to 1.07) compared with patients admitted to hospital.

There may be an increased risk of mortality in all three hospital-at-home cohorts (site one: relative risk 1.09; 95%CI 1.00 to 1.19) (site two: relative risk 1.29; 95%CI: 1.15 to 1.44) (site three: relative risk 1.27; 95%CI: 1.06 to 1.54) compared with the hospital cohort after PSM and regression to adjust for confounding. The Kaplan-Meier survival curves presented in Figure 3 show higher survival rates in the inpatient control cohorts in all three sites, and after weighting with the propensity score the control cohort in site two still had a higher survival rate than the hospital-at-home cohort. The difference in survival in site three between the results reported in Table 3 and the survival curve after weighting is explained by the fact that Kaplan-Meier curves are only weighted with the propensity score without performing an additional regression analysis.



For peer review only - http://bmjopen.bmj.com/site/about/guidelines.xhtml

Table 3. Results of the propensity score matched regression analyses

| | | | |
|---|---|---|---|
| **Panel A: main analysis** | | | |
| *Outcome variable* | *Site one (n=13267)* | *Site two (n=4769)* | *Site three (n=2110)* |
| Total costs during follow-up period# | 0.82 (0.03) [0.76;0.89] <0.001 | 1.00 (0.05) [0.92;1.09] 0.982 | 1.15 (0.09) [0.99;1.33] 0.073 |
| Total costs in 6 months after discharge | 1.27 (0.07) [1.14;1.41] <0.001 | 1.09 (0.07) [0.95;1.24] 0.219 | 1.70 (0.17) [1.40;2.07] <0.001 |
| Mortality rate during follow-up | 1.09 (0.05) [1.00;1.19] 0.059 | 1.29 (0.07) [1.15;1.44] <0.0010 | 1.27 (0.12) [1.06;1.54] 0.011 |
| **Panel B: subgroup analysis including only patients with dementia** | | | |
| *Outcome variable* | *Site one (n=2321)* | *Site two (n=1053)* | *Site three (n=280)* |
| Total costs during follow-up period# | 0.76 (0.05) [0.66;0.87] <0.001 | 0.76 (0.06) [0.66;0.88] <0.001 | 0.87 (0.15) [0.63;1.21] 0.409 |
| Total costs in 6 months after discharge | 1.18 (0.11) [0.99;1.41] 0.071 | 0.75 (0.09) [0.59;0.96] 0.021 | 1.58 (0.41) [0.95;2.63] 0.078 |
| Mortality rate during follow-up | 1.05 (0.09) [0.89;1.24] 0.594 | 1.41 (0.12) [1.19;1.67] <0.001 | 1.65 (0.32) [1.12;2.41] 0.011 |
| **Panel C: subgroup analysis including only survivors** | | | |
| *Outcome variable* | *Site one (n=10132)* | *Site two (n=3584)* | *Site three (n=1691)* |
| Total costs during follow-up period# | 0.85 (0.04) [0.77;0.94] 0.002 | 1.11 (0.03) [1.00;1.25] 0.058 | 1.20 (0.11) [1.00;1.43] 0.046 |
| Total costs in 6 months after discharge | 1.23 (0.08) [1.08;1.40] 0.002 | 1.17 (0.10) [0.99;1.38] 0.070 | 1.71 (0.20) [1.36;2.15] <0.001 |
| ***Panel D: sensitivity analysis*** | | | |
| *Outcome variable* | *Site one (n=13267)* | *Site two (n=4769)* | *Site three (n=2110)* |
| Total costs during follow-up period# (assuming 50% lower intervention costs) | 0.77 (0.03) [0.71;0.84] <0.001 | 0.81 (0.04) [0.74;0.9] 0.001 | 1.07 (0.09) [0.91;1.25] 0.399 |
| Total costs during follow-up period# (assuming 50% higher intervention costs) | 0.87 (0.03) [0.81;0.94] 0.001 | 1.18 (0.05) [1.09;1.28] <0.001 | 1.23 (0.09) [1.07;1.42] 0.004 |

# It includes the index admission period and 6 months post-discharge; Note: The results are presents as coefficient (se) [95%CI] p value; The results are after matching and adjusting for age, gender, socio-economic status, primary and secondary ICD-10 codes of index admission, type of long-term condition, mortality (for the analysis of costs), 2-year costs prior to the index admission (by cost category as listed in Box 1).

Figure 3. Survival curves before and after propensity score matching

## *Results of the subgroup analysis*

Patients with dementia (Panel B in Table 3) admitted to hospital-at-home services in site one and site two had about 25% lower costs (site one: ratio of means 0.75; 95%CI 0.65 to 0.87; site two: ratio of means 0.76 95%CI: 0.66 to 0.88) during the index admission and six months post-discharge. After excluding the index admission period, the same difference in mean costs remained in site two. We found that the population who were admitted to hospital-at-home, and had a diagnosis of dementia, may have an increased risk of death in two sites (site two: relative risk 1.41, 95%CI 1.19 to 1.67; site three: relative risk 1.65, 95%CI 1.12 to 2.41) compared with those who had a diagnosis of dementia and who were admitted to hospital.

When we excluded people who died during follow-up (i.e. during index admission and 6 months after discharge), patients admitted to hospital-at-home in site one had lower costs (ratio of means 0.85, 95%CI: 0.77 to 0.94), while there was 11% increase in costs in site two (ratio of means 1.11, 95%CI: 1.00 to 1.25) and 20% increase in site three (ratio of means 1.20, 95%CI: 1.00 to 1.43); the mean costs were higher in the hospital-at-home cohort when the costs during the index admission were excluded (site one: ratio of means 1.23, 95%CI: 1.08 to 1.40; site two: ratio of means 1.17, 95% CI 0.99 to 1.38; site three: ratio of means 1.71, 95% CI 1.36 to 2.15) compared with patients admitted to hospital (Panel C in Table 3).


For peer review only - http://bmjopen.bmj.com/site/about/guidelines.xhtml

*Results of the sensitivity analyses*

The results from the sensitivity analysis (Panel D in Table 3) showed that patients in the hospital-at-home cohort in site one had 13% lower costs (ratio of means 0.87; 95%CI: 0.81 to 0.94) during the follow-up period (i.e. during index admission and 6 months after index discharge) when the hospital-at-home service costs were assumed to be 50% higher than in the main analysis. In site two, the results from the sensitivity analysis showed that the uncertainty in hospital-at-home service costs lead to increased costs or cost savings by about 18% during the whole follow-up period. In site three, the sensitivity analysis showed a 23% cost increase (ratio of means 1.23; 95%CI: 1.07 to 1.42), if the intervention costs of hospital-at-home were 50% higher.

# Discussion

*Main findings*

Patients who received healthcare from the hospital-at-home services were older, were more socioeconomically disadvantaged, had higher morbidity (measured by the number of long term conditions), higher rates of previous hospitalisation, and there was a greater proportion of women compared with the group admitted to hospital. The two groups also differed in terms of their clinical diagnosis, with the most marked difference across the three services being a greater percentage (five to ten percent difference) of people with dementia. The higher healthcare costs over the two years prior to index admission in those admitted to hospital-at-home were mainly driven by the costs of non-elective hospital care. The cost of providing hospital-at-home varied between the three sites from £628 to £2928 per admission, and costs were driven primarily by staff costs. Our findings indicate that hospital-at-home might be associated with an increase in healthcare costs in the six months after index discharge. However, this increase in costs might be offset by likely cost-savings during the index admission. The higher healthcare cost at six months after index discharge, was driven primarily by acute non-elective admissions. Interpreting this is not straightforward; it might indicate a lack of resources during the index admission to hospital-at-home, or an increased risk of hospital admission in the population who receive their healthcare through hospital-at-home. The suggestion of an increased risk of mortality at six months after the index admission might be genuine, or could indicate that propensity score matching did not control for all differences between the groups and thus, the estimates are subject to residual confounding.[13 14]

*Comparison with previous studies*

A meta-analysis of six small randomised controlled trials concluded that admission avoidance hospital-at-home probably makes little or no difference to the risk of death or transfer to hospital at


For peer review only - http://bmjopen.bmj.com/site/about/guidelines.xhtml

six months' follow-up, and might increase the likelihood of living at home (albeit with low-certainty evidence); and highlighted the lack of evidence on cost.[2] Studies that have used 'real life data' offer the potential to address criticisms of limited external validity from randomised trials; and propensity score matching is one technique that has been used to balance co-variates when analysing routinely collected health data to assess these type of service delivery interventions. Findings have been consistent, and previous studies have reported higher rates of mortality and unplanned admission for those who received an intermediate care intervention, compared with matched controls.[6 14 15] However, it is possible that these findings are subject to residual confounding.

*Potential mechanisms and interpretation*

Healthcare services that cross the interface of primary and secondary care can bridge and strengthen the integration of acute and community services, and social care. However, by definition this can lead to a complex arrangement of services that reflect availability of local resources,[16] and a willingness to innovate. The hospital-at-home services evaluated in this analysis were established to reduce the demand for acute hospital beds by providing an alternative to admission to hospital, and to lower the risk of functional decline from the limited mobility that older people might experience when in hospital. However, it is possible that the services have several functions, for example by providing both rapid response and reablement, and this is reflected in the diverse population included in this analysis.

*Implications for clinicians and policy makers*

The variation in intervention costs of the three hospital-at-home services is primarily driven by staff costs, and the findings of the sensitivity analysis confirms that staff costs are likely to determine whether a hospital-at-home service leads to higher costs or cost savings. The skill-mix of healthcare professionals who provide hospital-at-home should be guided by national standards, the type of patients the service targets, and the function of the service in terms of whether or not the service supplements existing community based healthcare, substitutes for hospital level care, augments palliative care services or a combination of these. The integration of these types of service with existing primary and secondary care services, for example the provision of out-of-hours care by primary care services, might also determine the costs of these services. Managerial capacity of these services is expected to be of crucial importance in setting-up and managing the team of professionals able to provide high quality care.

The absence of evidence based guidelines about who and under which conditions a patient may be admitted to admission avoidance hospital-at-home might explain the variation in the set-up of services, and the relatively small size of the services. This is confirmed by the National Audit of


For peer review only - http://bmjopen.bmj.com/site/about/guidelines.xhtml

Intermediate Care , [17] that was established in response to concerns about governance structures in intermediate care services, and reported a complex pattern of service provision.

Data on the role and capability of informal care givers is largely absent. In many cases, people admitted to hospital-at-home services receive care from their partners who if old might have health issues themselves. In the context of our findings, that patients admitted to hospital-at-home services are older and more fragile than patients admitted to hospital, there might be a risk that carers are overburdened by being involved in the provision of healthcare.

*Strengths and limitations*

The strengths of this study include the dataset from three of the largest Health Boards in Scotland, the quasi-experimental study design that has allowed inferences from real world evidence, and the sensitivity analyses that helped to address uncertainty in the results. The major limitation of this type of non-randomised comparison is residual confounding. While matching individuals and performing regression analysis can reduce this risk, it is possible that the two populations differed in frailty because we did not match and adjust for differences in the use of community and social services prior to index admission. If unobserved confounders were part of the clinical-decision making by GPs and geriatricians to admit patients to hospital-at-home or hospital, our findings might be biased due to confounding by clinical indication. This type of confounding is often not measured directly because standardised criteria are not available to guide clinical decision-making.[18 19] Therefore, the magnitude of this bias in our results depends on the clinical-decision making process to admit patients to hospital-at-home in the three sites. If clinicians did not consider hospital-at-home as a substitute service to hospitalisation then confounding by indication would increase the residual confounding in our analysis. GPs and geriatricians who refer patients to hospital-at-home are likely to have a clinical bias in preferring to keep older, frailer and terminally ill patients in their own home. Using hospital-at-home admission criteria to define the control cohort accepts that such open criteria will include general medical patients who are likely to have fewer comorbidities, be younger and with a longer life expectancy. However, as the results of the survivors' subgroup analysis were very similar with the results of the main cost analysis we expect that the magnitude of the residual confounding to be small. Furthermore, the use of routine data has been used to reliably identify older people with fraility,[20] and approaches using clinical codes to define this population are being tested.[21]

*Future research*

Guidance on the use of real life data to evaluate service delivery interventions is largely absent, and could provide healthcare decision-makers with a relatively inexpensive way of evaluating local service innovations and how to avoid pitfalls in analysis and interpretations. Similar to all


For peer review only - http://bmjopen.bmj.com/site/about/guidelines.xhtml

observational studies, the findings of this study may be used to identify important questions to be tested in randomised trials.[18] A multi-centre randomised trial that measures outcomes that are key to decision-makers (including informal care giving), and is accompanied by a process evaluation to help explain the findings, is necessary to provide clinicians and policy makers with further evidence about the effectiveness and cost-effectiveness of admission avoidance hospital-at-home services across UK. The authors are involved in such a trial the results of which are expected to be available in 2019.[22]

## Conclusions

We found differences in the populations admitted to hospital-at-home and hospital. The likely higher cost in all three hospital-at-home cohorts compared with the hospital cohorts during the six months following discharge, highlight the importance of characterising populations eligible to receive these types of healthcare services and of assessing subsequent use of health, social, and informal care following admission to hospital-at-home or hospital.

## Competing interests

All authors have completed the ICMJE uniform disclosure form. GE is employed at the Monklands hospital and leading the hospital-at-home service in site one. All other authors declare no support from any organisation for the submitted work; no financial relationships with any organisations that might have an interest in the submitted work in the previous three years; no other relationships or activities that could appear to have influenced the submitted work.

## Details of contributors

AT, GE, and SS were responsible for study concept, GE facilitated the acquisition of data; AF and SS led the writing of the protocol, study design and drafting of the manuscript; AT performed the statistical analysis. All authors interpreted the data, critically revised the manuscript for important intellectual content and approved the final version for submission. AT and SS are guarantors.

## Acknowledgement

We would like to thank Charmaine Walker, Jenny Boyd, Alistair Smith and Josh Matthews from ISD Scotland for providing us with the data as well as Christine McGregor (economist in the Scottish Government) for her insightful views and expertise. We are also indebted to Dr Mike Gardner and Prof Alastair Gray (both University of Oxford), Prof Stavros Petrou (University of Warwick), and Dr



For peer review only - http://bmjopen.bmj.com/site/about/guidelines.xhtml

Matthew Sperrin (University of Manchester) for commenting on previous drafts of the manuscript. Our thanks also to Prof Gillian Parker (University of York), Dr Angela Coulter (University of Oxford) and Prof Stuart Parker (University of Newcastle) for their useful reflection on the study findings. Finally, we would like to thank all healthcare staff in all three sites who made this study happen.

## Ethical approval

We obtained signed release forms from each Health Board's Caldicott guardian.

## Funding

NIHR, UK. (12/5003//01; "How to Implement Cost-Effective Comprehensive Geriatric Assessment")

## Data sharing agreement

No additional data are available.

## References

1. World Health Organization. Noncommunicable diseases progress monitor 2015. Geneva: World Health Organization, 2015.
2. Shepperd S, Iliffe S, Doll HA, et al. Admission avoidance hospital at home. *Cochrane Database Syst Rev* 2016;9:CD007491. doi: 10.1002/14651858.CD007491.pub2
3. Sherman RE, Anderson SA, Dal Pan GJ, et al. Real-World Evidence - What Is It and What Can It Tell Us? *N Engl J Med* 2016;375(23):2293-97. doi: 10.1056/NEJMsb1609216
4. Johns B, Baltussen R, Hutubessy R. Programme costs in the economic evaluation of health interventions. *Cost effectiveness and resource allocation : C/E* 2003;1(1):1.
5. Stuart EA. Matching methods for causal inference: A review and a look forward. *Statistical science : a review journal of the Institute of Mathematical Statistics* 2010;25(1):1-21. doi: 10.1214/09-STS313
6. Garrido MM, Kelley AS, Paris J, et al. Methods for constructing and assessing propensity scores. *Health Serv Res* 2014;49(5):1701-20. doi: 10.1111/1475-6773.12182
7. Baser O. Too much ado about propensity score models? Comparing methods of propensity score matching. *Value Health* 2006;9(6):377-85. doi: 10.1111/j.1524-4733.2006.00130.x
8. Craig P, Cooper C, Gunnell D, et al. Using natural experiments to evaluate population health interventions: new Medical Research Council guidance. *J Epidemiol Community Health* 2012;66(12):1182-6. doi: 10.1136/jech-2011-200375
9. Rubin DB. Using propensity scores to help design observational studies: application to the tobacco litigation. *Health Services & Outcomes Research Methodology* 2001;2:169-88.
10. Funk MJ, Westreich D, Wiesen C, et al. Doubly robust estimation of causal effects. *Am J Epidemiol* 2011;173(7):761-7. doi: 10.1093/aje/kwq439
11. Leist AK. Social Inequalities in Dementia Care, Cure, and Research. *J Am Geriatr Soc* 2017;65(5):1100-01. doi: 10.1111/jgs.14893
12. World Health Organization. Draft global action plan on the public health response to dementia, 2016.


For peer review only - http://bmjopen.bmj.com/site/about/guidelines.xhtml

13. Iliffe S. Hospital at home: from red to amber?. Data that will reassure advocates-but without satisfying the sceptics. *Bmj* 1998;316(7147):1761-2.
14. Steventon A, Bardsley M, Billings J, et al. The role of matched controls in building an evidence base for hospital-avoidance schemes: a retrospective evaluation. *Health Serv Res* 2012;47(4):1679-98. doi: 10.1111/j.1475-6773.2011.01367.x
15. Lewis G, Vaithianathan R, Wright L, et al. Integrating care for high-risk patients in England using the virtual ward model: lessons in the process of care integration from three case sites. *Int J Integr Care* 2013;13:e046.
16. Young J, Gladman JR, Forsyth DR, et al. The second national audit of intermediate care. *Age Ageing* 2015;44(2):182-4. doi: 10.1093/ageing/afu174
17. NHS Benchmarking Network. Summary report- England. National audit of intermediate care: NHS Benchmarking Network, 2017.
18. Freemantle N, Marston L, Walters K, et al. Making inferences on treatment effects from real world data: propensity scores, confounding by indication, and other perils for the unwary in observational research. *Bmj* 2013;347:f6409. doi: 10.1136/bmj.f6409
19. Wong AY, Root A, Douglas IJ, et al. Cardiovascular outcomes associated with use of clarithromycin: population based study. *Bmj* 2016;352:h6926. doi: 10.1136/bmj.h6926
20. Clegg A, Bates C, Young J, et al. Development and validation of an electronic frailty index using routine primary care electronic health record data. *Age Ageing* 2017 doi: 10.1093/ageing/afx001
21. Ham C, York N, Sutch S, et al. Hospital bed utilisation in the NHS, Kaiser Permanente, and the US Medicare programme: analysis of routine data. *Bmj* 2003;327(7426):1257. doi: 10.1136/bmj.327.7426.1257
22. Shepperd S, Cradduck-Bamford A, Butler C, et al. A multi-centre randomised trial to compare the effectiveness of geriatrician-led admission avoidance hospital at home versus inpatient admission. *Trials* 2017;18(1):491. doi: 10.1186/s13063-017-2214-y

For peer review only


For peer review only - http://bmjopen.bmj.com/site/about/guidelines.xhtml

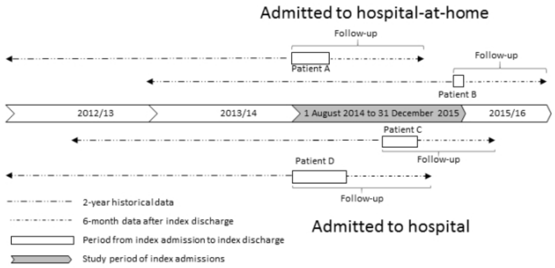


Figure 1. Illustration of obtained data from site one

338x190mm (96 x 96 DPI)

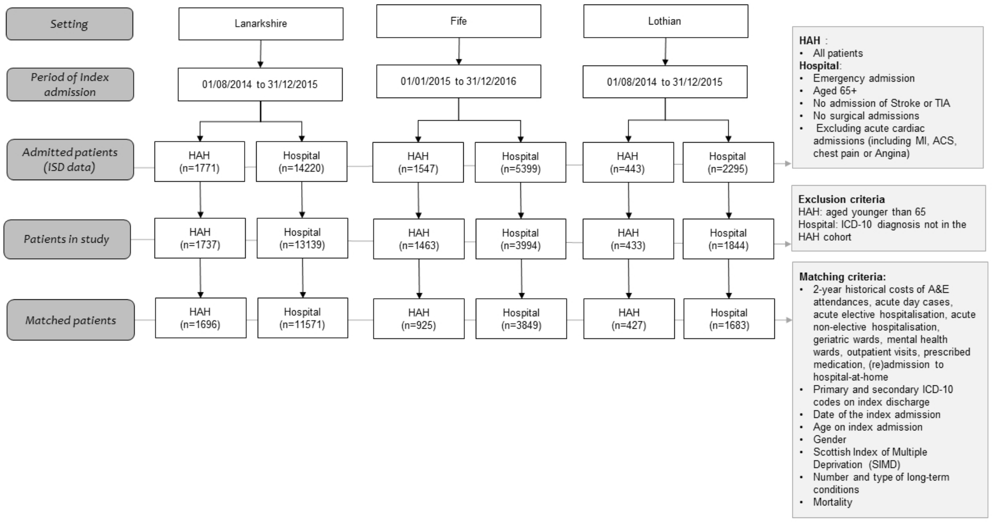


Figure 2 Flowchart of study population

338x190mm (96 x 96 DPI)

Figure 3. Survival curves before and after propensity score matching

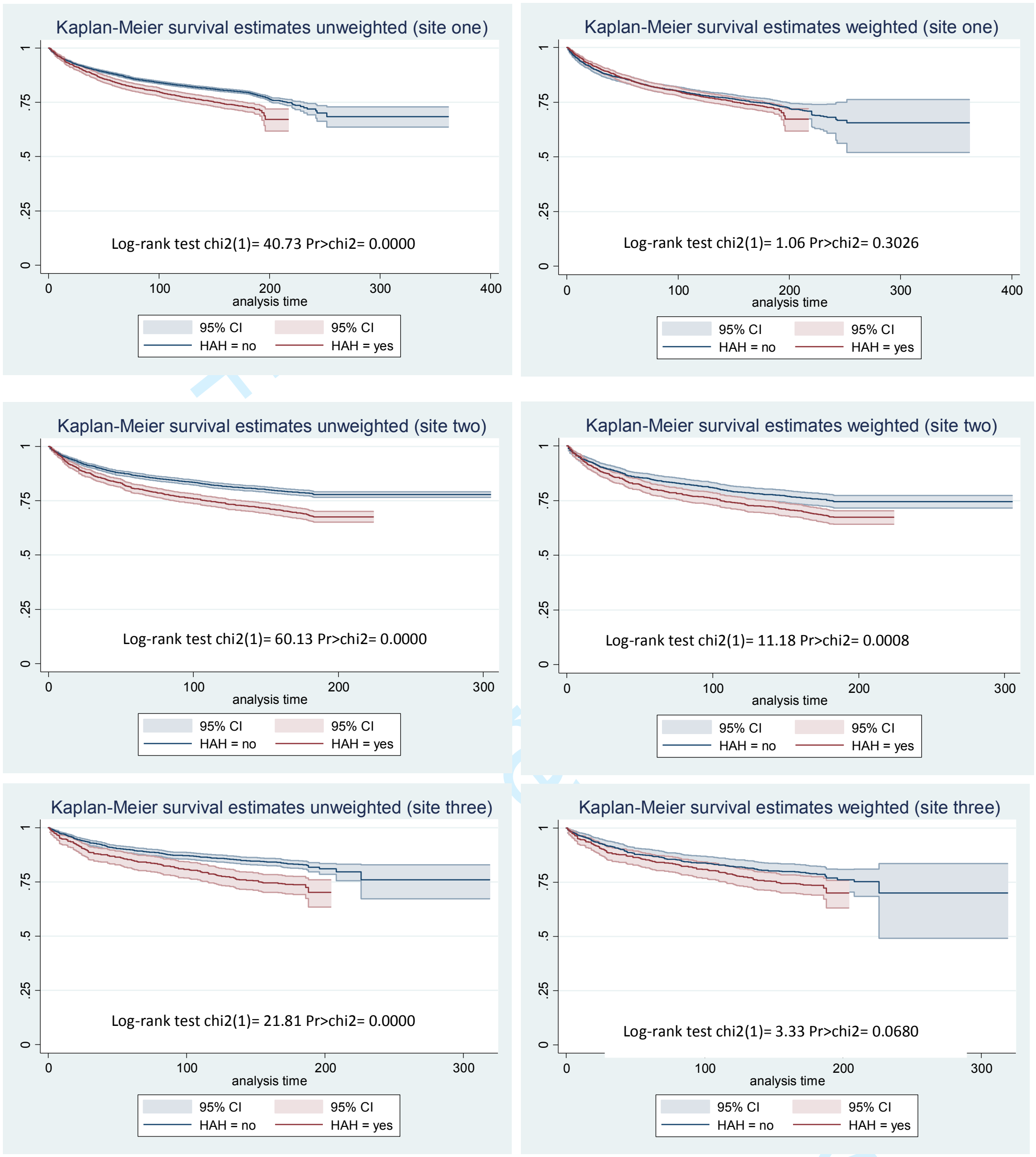


Note: The cohorts in each site were matched on age, gender, socio-economic status, primary and secondary ICD-10 codes of index admission, type of long-term condition, 2-year costs prior to the index admission (by cost category as listed in Box 1); Weighted refers to weighting the observation of each patient based on the propensity score to be in the hospital-at-home cohort as described in the propensity score matching section.

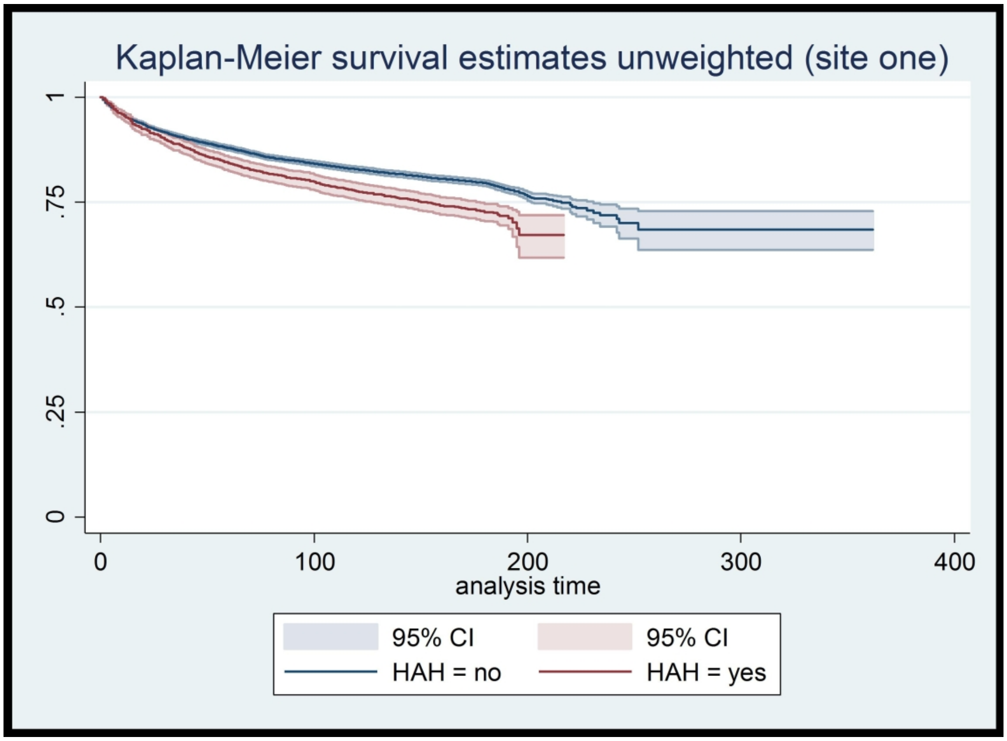


886x649mm (96 x 96 DPI)

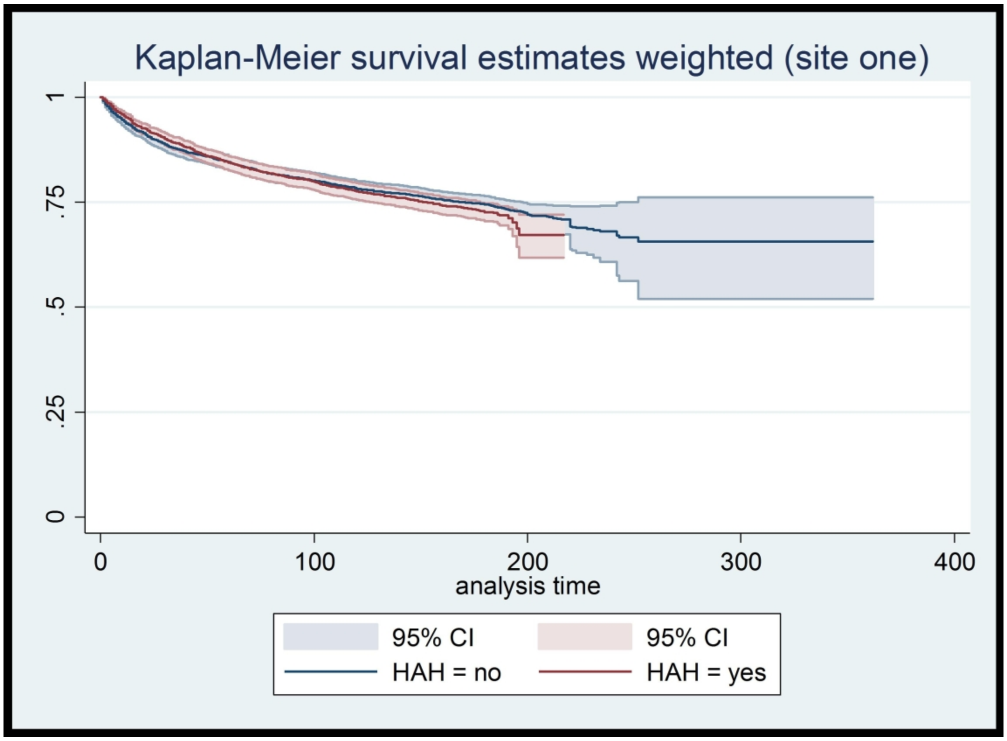


886x649mm (96 x 96 DPI)

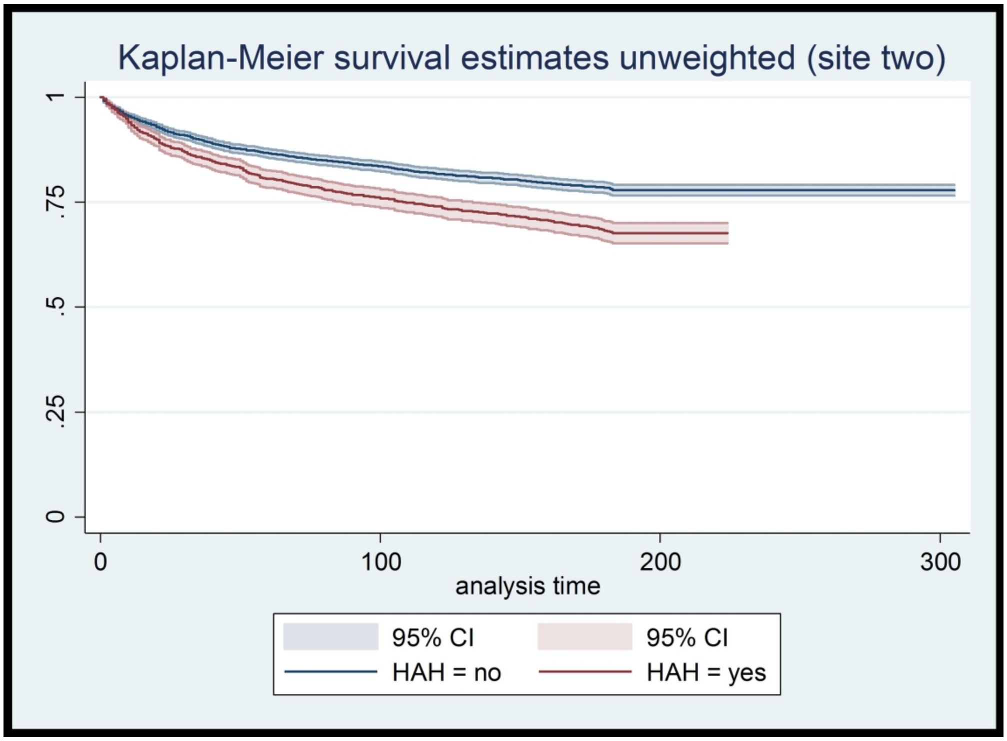


886x649mm (96 x 96 DPI)

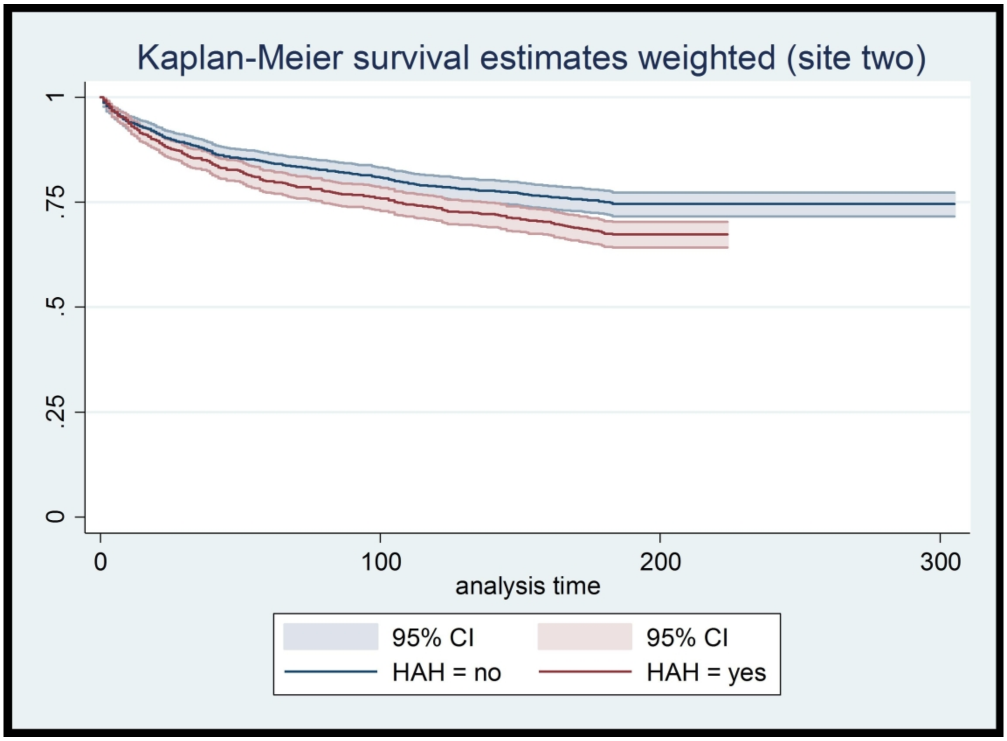


886x649mm (96 x 96 DPI)

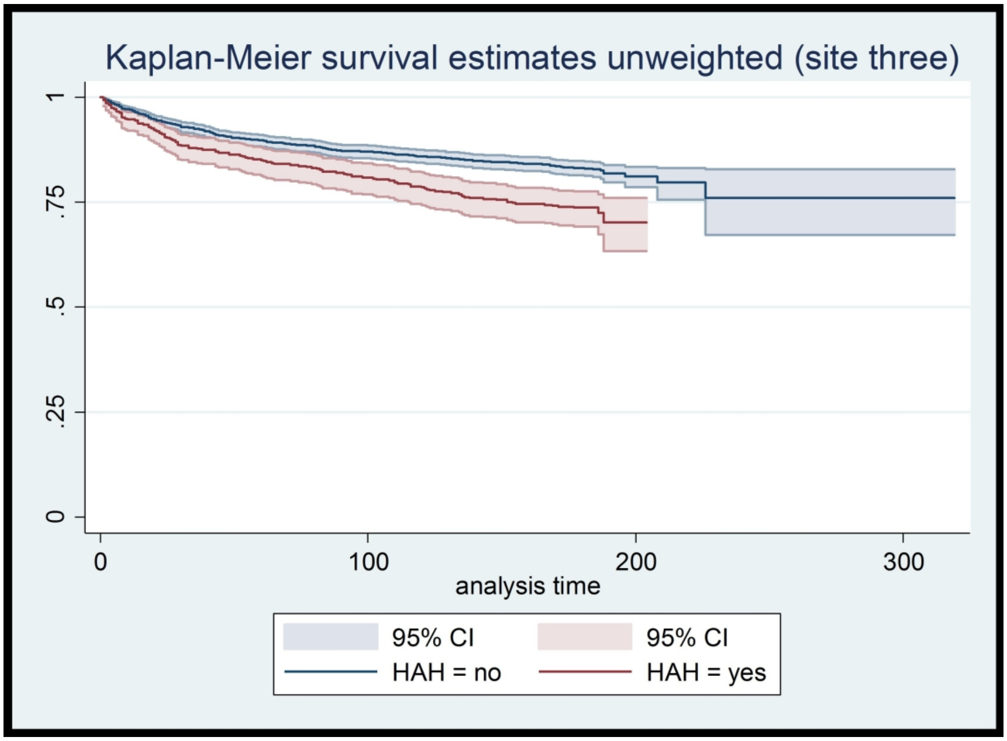


886x649mm (96 x 96 DPI)

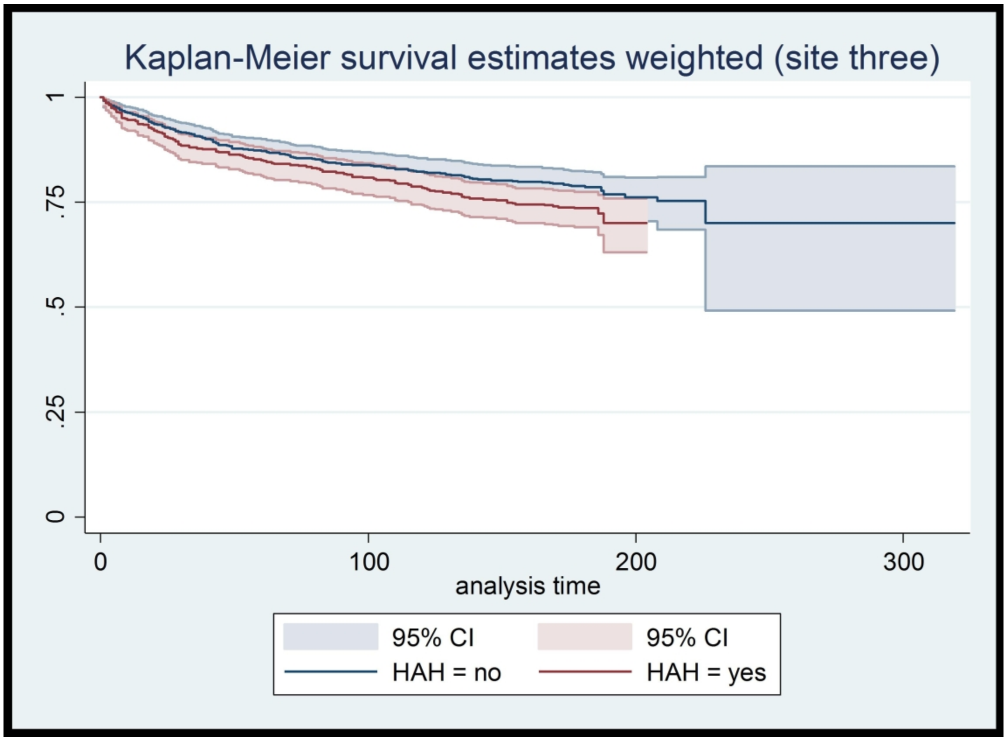


886x649mm (96 x 96 DPI)

Appendix 1. Calculation of admission avoidance hospital-at-home in each site

**Site one**

| | | | | |
|---|---|---|---|---|
| | | | **PERIOD** | |
| **from:** | 01/08/2014 | | **Until:** 01/01/2016 | **17** |
| | (dd/mm/yyyy) | | (dd/mm/yyyy) | Months |

| | | | |
|---|---|---|---|
| | | | Source of |
| **Number of HAH admissions (in period)** | 1771 | | ISD IPD data (1/8/14-31/12/15) |
| **Length of HAH stay per episode (in** | 5.53886 | Mean | ISD IPD data (1/8/14-31/12/15) |
| | 0.125605 | Standard error | |
| **HAH bed days (period)** | 9809 | | |

| | | | | | | |
|---|---|---|---|---|---|---|
| **A.1.** | **Staff costs** | | | | | |
| N | Profession | WTEs | Gross | Summary salary | Source of | **Total** |
| a) | Medical staff | | | | | |
| 1 | Consultant | 1.50 | £151,596 | | Business | £227,394 |
| 2 | Agency consultant | 0.16 | £156,926 | | Business | £25,651 |
| 3 | Consultant | 1.07 | £119,710 | | Business | £127,767 |
| b) | Nursing and pharmacy services | | | | | |
| 1 | Band 3 nurse | 3.00 | £24,790 | | Business | £74,369 |
| 2 | Band 6 nurse | 1.49 | £41,425 | | Business | £61,740 |
| 3 | Band 5 Bank nurse | 0.71 | £32,885 | | Business | £23,399 |
| 4 | Band 6 Bank nurse | 0.36 | £38,471 | | Business | £13,687 |
| 5 | Band 7 pharmacist | 0.71 | £55,491 | | Business | £39,484 |
| 6 | Band 5 nurse | 0.16 | £37,036 | | Business | £6,054 |
| 7 | Band 6 nurse | 1.42 | £42,342 | | Business | £60,303 |
| 8 | Band 7 nurse | 1.00 | £42,444 | | Business | £42,444 |
| 9 | Band 8a nurse | 0.71 | £53,126 | | Business | £37,801 |
| c) | Allied health professions | | | | | |
| 1 | Band 6 occupational therapist | 2.59 | £35,489 | | Business | £91,793 |
| 2 | Band 6 physiotherapist | 1.16 | £46,585 | | Business | £54,200 |
| 3 | Band 4 assistant practitioners for rehab | 3.59 | £24,660 | | Business | £88,444 |
| 4 | Band 6 physiotherapy | 0.71 | £46,848 | | Business | £33,334 |
| d) | Administration, ICT and management | | | | | |
| 1 | Band 2 admin/clerical | 0.30 | £19,346 | | Business | £5,804 |
| 2 | Band 3 admin/clerical | 1.00 | £23,948 | | Business | £23,948 |
| 3 | Band 3 admin/clerical | 0.71 | £21,353 | | Business | £15,193 |
| e) | Support services staff | | | | | |
| 1 | | | | | | £0 |
| | **Total** | | | | | £1,052,80 |
| **A.2.** | **Trainning costs**<br>Note: the time to attend a course should be included in | | | | | |
| No. | Profession | Number of | Cost per | Summary costs | Source of | **Total** |
| 1 | Acute urgent care course | 20 | £250 | | | £5,000 |
| 2 | Prescribing course | 3 | £310 | | | £930 |
| | **Total** | | | | | £5,930 |
| **A.3.** | **Transport costs** | | | | | |
| No. | Cost item | Number of | Cost per item | Summary costs | Source of | **Total** |
| 1 | Travel and subsistence | | | £37,918 | Business | £37,918 |
| | **Total** | | | | | £37,918 |
| **A.4.** | **Information and communication costs**<br>**(e.g. brochures and leaflets for patients and their** | | | | | |
| No. | Cost item | Number of | Cost per item | Summary costs | Source of | **Total** |
| 1 | | | | | | £0 |
| | **Total** | | | | | £0 |
| **A.5.** | **Clinical materials/equipment and drugs** | | | | | |
| No. | Cost item | Number of | Cost per item | Summary costs | Source of | **Total** |
| 1 | Instruments and sundries | | | £2,867 | Business | £2,867 |

For peer review only - http://bmjopen.bmj.com/site/about/guidelines.xhtml

| No. | Cost item | Number of | Cost per item | Summary costs | Source of | Total |
|---|---|---|---|---|---|---|
| 2 | Equipment repairs clinical | | | £585 | Business | £585 |
| 3 | Surgical appliances | | | £104 | Business | £104 |
| 4 | Drugs | | | £1,693 | Business | £1,693 |
| 5 | Equipment purchase clinical | | | £298 | Business | £298 |
| | **Total** | | | | | £5,546 |
| A.6. | **Support services supplies** | | | | | |
| No. | Cost item | Number of | Cost per item | Summary costs | Source of | **Total** |
| 1 | Catering | | | £177 | Business | £177 |
| 2 | Uniforms | | | £552 | Business | £552 |
| 3 | Printing and stationery | | | £737 | Business | £737 |
| 4 | Dressings | | | £473 | Business | £473 |
| 5 | general services | | | £16 | Business | £16 |
| | **Total** | | | | | £1,955 |
| A.7. | **Labs and diagnostics** | | | | | |
| No. | Cost item | Number of | Cost per item | Summary costs | Source of | **Total** |
| 1 | Diagnostic supplies | | | £559 | Business | £559 |
| | | | | | | £559 |
| A.8. | **Overhead costs** | | | | | |
| No. | Cost item | Number of | Cost per item | Summary costs | Source of | **Total** |
| 1 | Telephone | | | £3,794 | Business | £3,794 |
| 2 | Building | | | £119 | Business | £119 |
| 3 | Miscellaneous | | | £34 | Business | £34 |
| | **Total** | | | | | £3,947 |
| A.9. | **Other costs** | | | | | |
| No. | Cost item | Number of | Cost per item | Summary costs | Source of | **Total** |
| 1 | Equipment purchase non medical | | | £3,354 | Business | £3,354 |
| 2 | postage | | | £772 | Business | £772 |
| | **Total** | | | | | £4,126 |
| A.10 | **Additional costs** | | | | | |
| No. | Cost item | Number of | Cost per item | Summary costs | Source of | **Total** |
| 1 | | | | | | £0 |
| | **Total** | | | | | £0 |
| | | | | | **TOTAL** | **£1,112,79** |
| | | | | **Unit cost of HAH admission** | | **£628.34** |
| | | | | **Unit cost of HAH bed day** | | **£113.44** |

For peer review only

**Site two**

| | | | | | | |
|---|---|---|---|---|---|---|
| | | | | **PERIOD** | | |
| | **from:** | 01/01/2015 | | **Until:** | 01/01/2017 | **24** |
| | | (dd/mm/yyyy) | | (dd/mm/yyyy) | | Months |
| | | | | Source of | | |
| **Number of HAH admissions (in period)** | 1547 | | ISD IPD data | | | |
| **Length of HAH stay per episode (in** | 7.35 | Mean | ISD IPD data | | | |
| | 0.14 | Standard error | | | | |
| **HAH bed days (period)** | 11376 | | | | | |

| | | | | | | |
|---|---|---|---|---|---|---|
| **A.1.** | **Staff costs** | | | | | |
| N | Profession | WTEs | Gross | Summary salary | Source of | **Total** |
| a) | Medical staff | | | | | |
| 1 | Senior medical | | | £82,099 | Business | £82,099 |
| 2 | Professional fees and charges | | | £124,391 | Business | £124,391 |
| b) | Nursing and pharmacy services | | | | | |
| 1 | Nursing & Midwifery-trained | | | £2,904,576 | Business | £2,904,57 |
| 2 | Nursing & Midwifery-untrained | | | £627,532 | Business | £627,532 |
| 3 | Pharmacists | | | £43,715 | Business | £43,715 |
| 4 | Pharmacy Technicians | | | £14,471 | Business | £14,471 |
| c) | Allied health professions | | | | | |
| 1 | | | | | Business | £0 |
| d) | Administration, ICT and management | | | | | |
| 1 | Admin Clerical | | | £126,018 | Business | £126,018 |
| e) | Support services staff | | | | | |
| 1 | | | | | | £0 |
| | **Total** | | | | | £3,922,80 |

| | | | | | | |
|---|---|---|---|---|---|---|
| **A.2.** | **Trainning costs**<br>Note: the time to attend a course should be included in | | | | | |
| No. | Profession | Number of | Cost per | Summary costs | Source of | **Total** |
| 1 | Trainning costs | | | £1,512 | | £1,512 |
| | **Total** | | | | | £1,512 |

| | | | | | | |
|---|---|---|---|---|---|---|
| **A.3.** | **Transport costs** | | | | | |
| No. | Cost item | Number of | Cost per item | Summary costs | Source of | **Total** |
| 1 | Transport | | | £25,711 | Business | £25,711 |
| 2 | Travel And Subsistence | | | £340,388 | | £340,388 |
| | **Total** | | | | | £366,099 |

| | | | | | | |
|---|---|---|---|---|---|---|
| **A.4.** | **Information and communication costs**<br>**(e.g. brochures and leaflets for patients and their** | | | | | |
| No. | Cost item | Number of | Cost per item | Summary costs | Source of | **Total** |
| 1 | | | | | | £0 |
| | **Total** | | | | | £0 |

| | | | | | | |
|---|---|---|---|---|---|---|
| **A.5.** | **Clinical materials/equipment and drugs** | | | | | |
| No. | Cost item | Number of | Cost per item | Summary costs | Source of | **Total** |
| 1 | Drugs | | | £203,900 | Business | £203,900 |
| 2 | Equipment | | | £14,589 | Business | £14,589 |
| 3 | Paramedical Supplies | | | £3,015 | Business | £3,015 |
| 4 | Surgical Appliances | | | £18 | Business | £18 |
| 5 | Surgical Sundries | | | £80,855 | Business | £80,855 |
| | **Total** | | | | | £302,377 |

| | | | | | | |
|---|---|---|---|---|---|---|
| **A.6.** | **Support services supplies** | | | | | |
| No. | Cost item | Number of | Cost per item | Summary costs | Source of | **Total** |
| 1 | Bedding And Linen | | | £112 | Business | £112 |
| 2 | Cleaning | | | £8,251 | Business | £8,251 |
| 3 | General Services | | | £2,595 | | £2,595 |
| | **Total** | | | | | £10,958 |

| | | | | | | |
|---|---|---|---|---|---|---|
| **A.7.** | **Labs and diagnostics** | | | | | |
| No. | Cost item | Number of | Cost per item | Summary costs | Source of | **Total** |
| 1 | Cssd/diagnostic Supplies | | | £3,783 | | £3,783 |

For peer review only

| | | | | | | |
|---|---|---|---|---|---|---|
| | | | | | | £3,783 |
| **A.8.** | **Overhead costs** | | | | | |
| No. | Cost item | Number of | Cost per item | Summary costs | Source of | **Total** |
| 1 | Post Carriage And Telephones | | | £5,224 | | £5,224 |
| 2 | Printing And Stationery | | | £5,737 | Business | £5,737 |
| 3 | Property Maintenance | | | £1,174 | | £1,174 |
| 4 | Miscellaneous | | | £25 | Business | £25 |
| | **Total** | | | | | £12,160 |
| **A.9.** | **Other costs** | | | | | |
| No. | Cost item | Number of | Cost per item | Summary costs | Source of | **Total** |
| 1 | Provisions | | | £6 | Business | £6 |
| 2 | Uniforms | | | £334 | Business | £334 |
| | **Total** | | | | | £340 |
| **A.10** | **Additional costs** | | | | | |
| No. | Cost item | Number of | Cost per item | Summary costs | Source of | **Total** |
| 1 | Other Operating Income** | | | -£92,377 | | -£92,377 |
| | **Total** | | | | | -£92,377 |

| | |
|---|---|
| **TOTAL** | **£4,527,65** |
| **Unit cost of HAH admission** | **£2,926.73** |
| **Unit cost of HAH bed day** | **£398.01** |

For peer review only

| | | | | | | |
|---|---|---|---|---|---|---|
| Site three | | | | | | |
| | | from: | 01/01/2015 (dd/mm/yyyy) | PERIOD Until: (dd/mm/yyyy) | 01/01/2016 | 12 Months |
| | | | | Source of | | |
| | Number of HAH admissions (in period) | 598 | | ISD IPD data | | |
| | | 598 | | business case | | |
| | Length of HAH stay per episode (in days) | 7.35 | Mean | ISD IPD data | | |
| | | 0.14 | Standard error | | | |
| | HAH bed days (period) | 4397 | | | | |
| A.1. | Staff costs | | | | | |
| No. | Profession | WTEs | Gross annual | Summary salary cost | Source of | Total |
| a) | Medical staff | | | | | |
| 1 | Consultant | 1 | | £114,776 | Business | £114,77 |
| 2 | Specialty doctor | 1 | | £79,224 | Business | £79,224 |
| 3 | | | | | Business | £0 |
| 4 | | | | | | £0 |
| 5 | | | | | | £0 |
| b) | Nursing and pharmacy services | | | | | |
| 1 | Nurse (Band 6) | 3 | | £125,484 | Business | £125,48 |
| 2 | Nurse (Band 5) | 1.6 | | £53,256 | Business | £53,256 |
| c) | Allied health professions | | | | | |
| 1 | Occupational therapist | 1 | | £45,156 | Business | £45,156 |
| 2 | Physiotherapist | 1 | | £45,156 | Business | £45,156 |
| d) | Administration, ICT and management | | | | | |
| 1 | Admin Clerical | 1 | | £23,664 | Business | £23,664 |
| e) | Support services staff | | | | | |
| 1 | | | | | | £0 |
| | Total | | | | | £486,71 |
| A.2. | Trainning costs<br>Note: the time to attend a course should be included in | | | | | |
| No. | Profession | Number of | Cost per | Summary costs | Source of | Total |
| 1 | Trainning costs | | | £1,000 | | £1,000 |
| | Total | | | | | £1,000 |
| A.3. | Transport costs | | | | | |
| No. | Cost item | Number of | Cost per item | Summary costs | Source of | Total |
| 1 | Transport/travel | | | £20,000 | Business | £20,000 |
| | Total | | | | | £20,000 |
| A.4. | Information and communication costs<br>(e.g. brochures and leaflets for patients and their family) | | | | | |
| No. | Cost item | Number of | Cost per item | Summary costs | Source of | Total |
| 1 | | | | | | £0 |
| | Total | | | | | £0 |
| A.5. | Clinical materials/equipment and drugs | | | | | |
| No. | Cost item | Number of | Cost per item | Summary costs | Source of | Total |
| 1 | Drugs | | | £4,840 | Business | £4,840 |
| 2 | Medical supplies | | | £2,393 | Business | £2,393 |
| | Total | | | | | £7,233 |
| A.6. | Support services supplies | | | | | |
| No. | Cost item | Number of | Cost per item | Summary costs | Source of | Total |
| 1 | | | | | | £0 |
| | Total | | | | | £0 |
| A.7. | Labs and diagnostics | | | | | |
| No. | Cost item | Number of | Cost per item | Summary costs | Source of | Total |
| 1 | | | | | | £0 |
| | | | | | | £0 |
| A.8. | Overhead costs | | | | | |
| No. | Cost item | Number of | Cost per item | Summary costs | Source of | Total |

| | | | | | | |
|---|---|---|---|---|---|---|
| 1 | Phones, stationary etc. | | | £1,796 | Business | £1,796 |
| | Total | | | | | £1,796 |
| A.9. | Other costs | | | | | |
| No. | Cost item | Number of | Cost per item | Summary costs | Source of | Total |
| 1 | Mischellaneous | | | £250 | | £250 |
| | Total | | | | | £250 |
| A.10 | Additional costs | | | | | |
| No. | Cost item | Number of | Cost per item | Summary costs | Source of | Total |
| 1 | | | | | | £0 |
| | Total | | | | | £0 |

| | |
|---|---|
| TOTAL | £516,99 |
| Unit cost of HAH admission | £864.54 |
| Unit cost of HAH bed day | £117.57 |

For peer review only

Appendix 2 Results of selecting PSM technique and plots of covariance balance before and after propensity score matching

| | Site one | | Site two | | Site three | |
|---|---|---|---|---|---|---|
| *Variable* | *Costs* mean/median bias;Rubin's B/R | *Survival* mean/median bias;Rubin's B/R | *Costs* mean/median bias;Rubin's B/R | *Survival* mean/median bias;Rubin's B/R | *Costs* mean/median bias;Rubin's B/R | *Survival* mean/median bias;Rubin's B/R |
| Mahalanobis | 7.5/4.2;51.4/1.56 | 7.2/3.7;48.6/1.54 | 7.6/6.7;46.1/1.54 | 7.3/6.7;43.9/1.53 | 6.3/4.7/38.4/1.69 | 6.3/3.5/38.4/1.52 |
| 1-to-1 | 2.9/2.8;14.1/0.90 | 1.9/1.6;12.1/0.84 | 1.4/1.4;9.4/0.97 | 2.2/2.2;14.6/1.14 | 2.7/2.7/14.6/1.02 | 2.3/2.6/14.9/0.73 |
| K-to-1 | 1.9/1.6;11.3/0.76 | 1.9/1.5;12.0/0.81 | 1.8/1.5;11.0/0.83 | 2.4/2.4;13.6/0.76 | 3.6/2.9/16.5/0.99 | 2.8/2.0/16.5/0.94 |
| Kernel | 1.6/1.1;9.8/0.97 | 1.5/1.2;8.9/0.92 | 1.1/0.9;6.9/1.02 | 0.9/0.7;6.5/1.01 | 2.2/1.6/12.3/1.22 | 1.9/1.2/11.2/1.21 |
| Local linear regression | 1.5/1.2;9.4/0.89 | 1.6/1.4;9.4/0.89 | 1.7/1.0;11.0/0.32 | 2.3/1.4;12.8/0.43 | 1.8/1.6/9.6/1.27 | 1.6/1.2/8.5/1.35 |
| Spline | 2.9/2.6;15.7/0.94 | 2.4/2.0;14.9/0.91 | 3.2/2.6;17.5/0.46 | 3.2/2.3;21.0/1.07 | 3.9/3.1/21.6/0.47 | 3.9/2.3/25.7/1.02 |
| IPW | 11.5/5.8;83.2/0.76 | 11.5/5.6;83.1/0.75 | 11.6/8.3;61.3/0.92 | 11.2/7.8;60.2/0.89 | 10.5/8.5/52.2/0.77 | 10.2/8.5/50.9/0.77 |

Rubin's B: the absolute standardized difference of the means of the linear index of the propensity score in the treated and (matched) non-treated group; Rubin's R: the ratio of treated to (matched) non-treated variances of the propensity score index; Samples sufficiently balanced if B less than 25 and that R between 0.5 and 2.

Standardised percentage bias before and after local linear regression propensity score matching for costs in site one

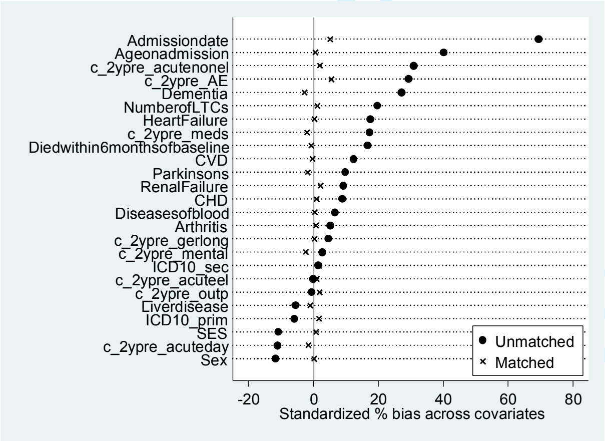


Standardised percentage bias before and after local linear regression propensity score matching for survival in site one

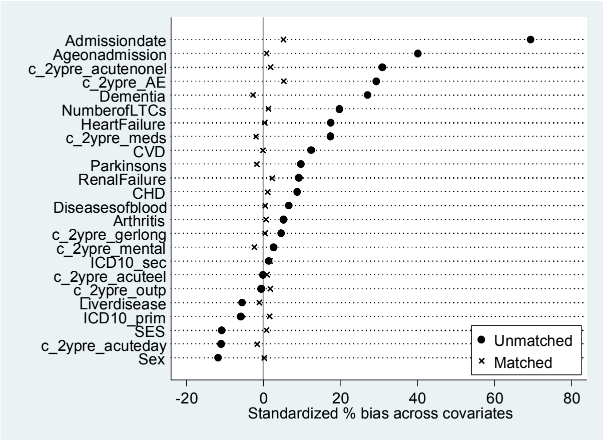


For peer review only

Standardised percentage bias before and after Kernel propensity score matching for costs in site two

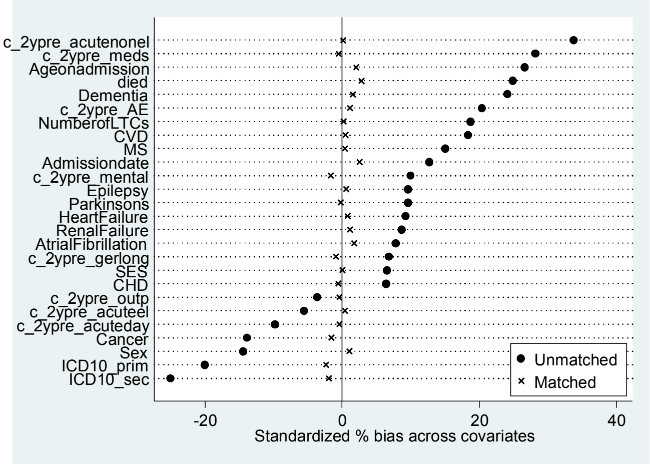


Standardised percentage bias before and after Kernel propensity score matching for survival in site two

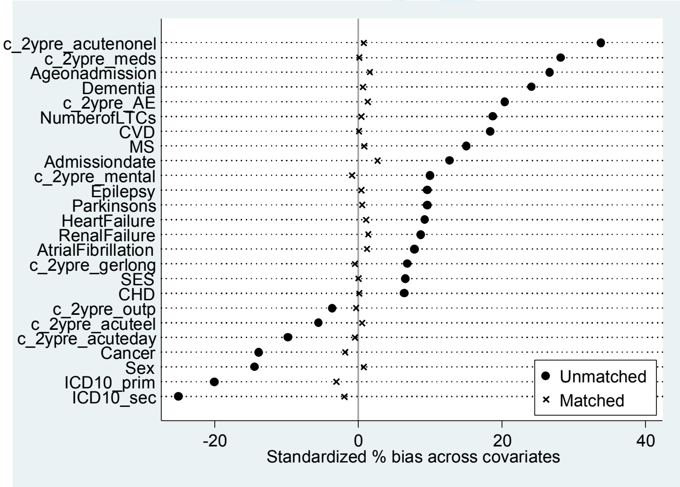

Standardised percentage bias before and after local linear regression propensity score matching for costs in site three

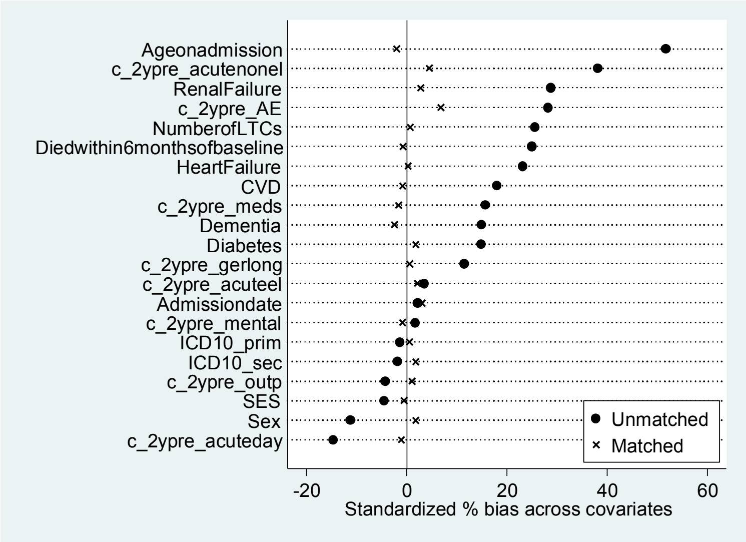


Standardised percentage bias before and after local linear regression propensity score matching for survival in site three

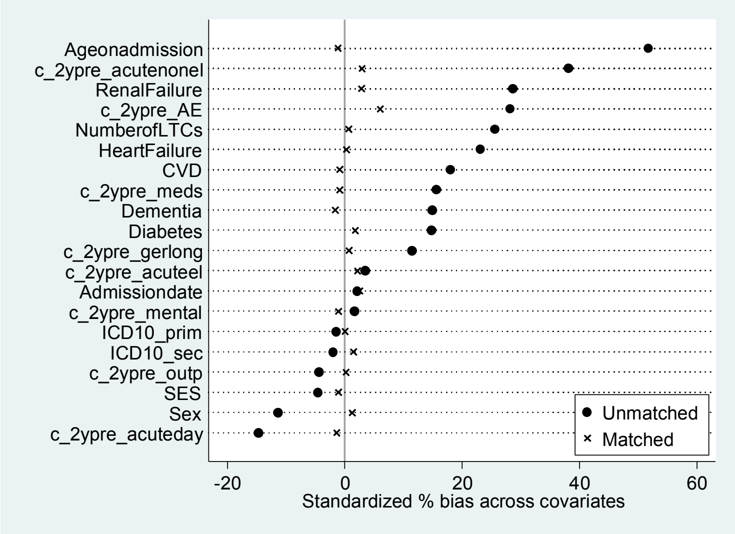

Propensity score distributions by cohort in each site

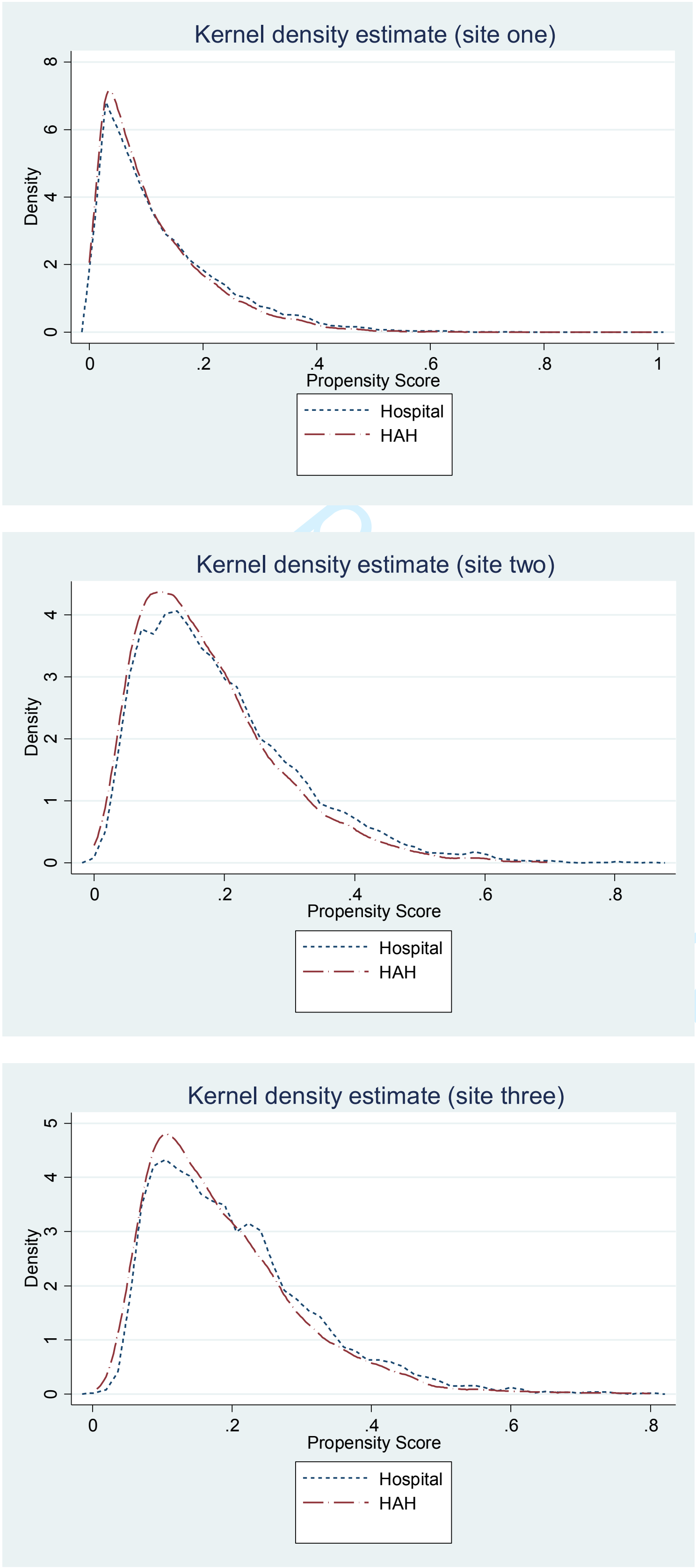

Appendix 3. Full results of the regression analyses

Association of hospital at home with total costs (after propensity score matching)

| | site one (n=13,267) | | site two (n=4,769) | | site three (n=2110) | |
|---|---|---|---|---|---|---|
| | Follow-up period coefficient (se) [95%CI] p value | 6 months after discharge coefficient (se) [95%CI] p value | Follow-up period coefficient (se) [95%CI] p value | 6 months after discharge coefficient (se) [95%CI] p value | Follow-up period coefficient (se) [95%CI] p value | 6 months after discharge coefficient (se) [95%CI] p value |
| HAH | 0.82 (0.03) [0.76;0.89] <0.001 | 1.27 (0.07) [1.14;1.41] <0.001 | 1.00 (0.05) [0.92;1.09] 0.982 | 1.09 (0.07) [0.95;1.24] 0.219 | 1.15 (0.09) [0.99;1.33] 0.073 | 1.70 (0.17) [1.4;2.07] <0.001 |
| Admission date | 1.00 (0.00) [1.00;1.00] 0.058 | 1.00 (0.00) [1.00;1.00] 0.009 | 1.00 (0.00) [1.00;1.00] 0.386 | 1.00 (0.00) [1.00;1.00] 0.824 | 1.00 (0.00) [1.00;1.00] 0.009 | 1.00 (0.00) [1.00;1.00] 0.056 |
| ICD10 primary | 1.00 (0.00) [1.00;1.00] 0.660 | 1.00 (0.00) [1.00;1.00] 0.230 | 1.00 (0.00) [1.00;1.00] 0.001 | 1.00 (0.00) [1.00;1.00] <0.001 | 1.00 (0.00) [1.00;1.00] 0.162 | 1.00 (0.00) [1.00;1.00] 0.101 |
| ICD10 secondary | 1.00 (0.00) [1.00;1.00] 0.641 | 1.00 (0.00) [1.00;1.00] 0.988 | 1.00 (0.00) [1.00;1.00] 0.146 | 1.00 (0.00) [1.00;1.00] 0.238 | 1.00 (0.00) [1.00;1.00] 0.897 | 1.00 (0.00) [1.00;1.00] 0.971 |
| 2yrs pre AE costs | 1.00 (0.00) [1.00;1.00] 0.240 | 1.00 (0.00) [1.00;1.00] 0.018 | 1.00 (0.00) [1.00;1.00] 0.624 | 1.00 (0.00) [1.00;1.00] 0.309 | 1.00 (0.00) [1.00;1.00] 0.284 | 1.00 (0.00) [1.00;1.00] 0.42 |
| 2yrs pre elective costs | 1.00 (0.00) [1.00;1.00] 0.906 | 1.00 (0.00) [1.00;1.00] 0.919 | 1.00 (0.00) [1.00;1.00] 0.588 | 1.00 (0.00) [1.00;1.00] 0.435 | 1.00 (0.00) [1.00;1.00] 0.865 | 1.00 (0.00) [1.00;1.00] 0.931 |
| 2yrs pre non-elective costs | 1.00 (0.00) [1.00;1.00] <0.001 | 1.00 (0.00) [1.00;1.00] 0.001 | 1.00 (0.00) [1.00;1.00] 0.694 | 1.00 (0.00) [1.00;1.00] 0.697 | 1.00 (0.00) [1.00;1.00] 0.018 | 1.00 (0.00) [1.00;1.00] 0.015 |
| 2yrs pre day case costs | 1.00 (0.00) [1.00;1.00] 0.098 | 1.00 (0.00) [1.00;1.00] 0.020 | 1.00 (0.00) [1.00;1.00] 0.005 | 1.00 (0.00) [1.00;1.00] <0.001 | 1.00 (0.00) [1.00;1.00] 0.14 | 1.00 (0.00) [1.00;1.00] 0.100 |
| 2yrs pre geriatric ward costs | 1.00 (0.00) [1.00;1.00] 0.005 | 1.00 (0.00) [1.00;1.00] 0.054 | 1.00 (0.00) [1.00;1.00] 0.001 | 1.00 (0.00) [1.00;1.00] 0.003 | 1.00 (0.00) [1.00;1.00] 0.634 | 1.00 (0.00) [1.00;1.00] 0.342 |
| 2yrs pre mental ward costs | 1.00 (0.00) [1.00;1.00] 0.880 | 1.00 (0.00) [1.00;1.00] 0.911 | 1.00 (0.00) [1.00;1.00] 0.009 | 1.00 (0.00) [1.00;1.00] 0.014 | 1.00 (0.00) [1.00;1.00] 0.111 | 1.00 (0.00) [1.00;1.00] 0.382 |
| 2yrs pre outpatient costs | 1.00 (0.00) [1.00;1.00] 0.087 | 1.00 (0.00) [1.00;1.00] 0.056 | 1.00 (0.00) [1.00;1.00] 0.026 | 1.00 (0.00) [1.00;1.00] 0.043 | 1.00 (0.00) [1.00;1.00] 0.683 | 1.00 (0.00) [1.00;1.00] 0.656 |
| 2yrs pre medication costs | 1.00 (0.00) [1.00;1.00] 0.798 | 1.00 (0.00) [1.00;1.00] 0.750 | 1.00 (0.00) [1.00;1.00] 0.172 | 1.00 (0.00) [1.00;1.00] 0.369 | 1.00 (0.00) [1.00;1.00] 0.687 | 1.00 (0.00) [1.00;1.00] 0.935 |
| Died during follow-up | 1.03 (0.04) [0.95;1.11] 0.530 | 0.91 (0.05) [0.82;1.01] 0.089 | 1.05 (0.05) [0.96;1.15] 0.302 | 0.90 (0.06) [0.78;1.05] 0.143 | 1.06 (0.09) [0.90;1.24] 0.498 | 0.97 (0.11) [0.78;1.21] 0.784 |
| Number of LTCs | 1.09 (0.02) [1.05;1.12] <0.001 | 1.12 (0.02) [1.07;1.16] <0.001 | 1.04 (0.02) [1.00;1.07] 0.054 | 1.06 (0.03) [1.00;1.11] 0.035 | 1.06 (0.03) [1.01;1.11] 0.017 | 1.10 (0.03) [1.03;1.17] 0.003 |
| Age on admission | 1.00 (0.00) [0.99;1.01] 0.383 | 1.00 (0.00) [0.99;1.01] 0.981 | 1.00 (0.00) [0.99;1.01] 0.984 | 1.00 (0.00) [1.00;1.00] 0.349 | 1.01 (0.01) [1.00;1.02] 0.045 | 1.01 (0.01) [0.99;1.02] 0.41 |
| Male | 1.09 (0.05) [1.01;1.19] 0.034 | 1.08 (0.06) [0.97;1.19] 0.136 | 0.95 (0.05) [0.86;1.05] 0.340 | 0.99 (0.08) [0.85;1.15] 0.859 | 0.97 (0.08) [0.83;1.13] 0.709 | 0.98 (0.10) [0.81;1.2] 0.875 |
| SES | 1.00 (0.01) [0.98;1.02] 0.988 | 1.00 (0.01) [0.98;1.03] 0.741 | 1.01 (0.01) [1.00;1.03] 0.182 | 1.03 (0.01) [1.00;1.05] 0.033 | 1.00 (0.02) [0.97;1.03] 0.899 | 1.01 (0.02) [0.97;1.05] 0.779 |
| Arthritis | 0.96 (0.04) [0.88;1.05] 0.398 | 0.95 (0.05) [0.85;1.06] 0.346 | ------------ | ------------ | ------------ | ------------ |
| Atrial Fibrillation | ------------ | ------------ | 1.09 (0.06) [0.98;1.2] 0.098 | 1.13 (0.08) [0.97;1.30] 0.113 | ------------ | ------------ |
| Cancer | ------------ | ------------ | 1.04 (0.05) [0.94;1.15] 0.485 | 1.07 (0.08) [0.92;1.24] 0.403 | ------------ | ------------ |
| CVD | 1.01 (0.06) [0.91;1.13] 0.767 | 0.99 (0.07) [0.86;1.13] 0.903 | 1.08 (0.06) [0.97;1.2] 0.168 | 1.11 (0.09) [0.95;1.29] 0.199 | 1.10 (0.11) [0.90;1.34] 0.339 | 1.07 (0.13) [0.84;1.37] 0.585 |
| Liver disease | 1.21 (0.13) [0.98;1.50] 0.074 | 1.20 (0.14) [0.95;1.51] 0.130 | ------------ | ------------ | ------------ | ------------ |
| Dementia | 1.06 (0.05) [0.97;1.17] 0.179 | 1.07 (0.07) [0.95;1.21] 0.236 | 1.00 (0.05) [0.91;1.11] 0.942 | 1.03 (0.08) [0.89;1.19] 0.683 | 1.14 (0.11) [0.95;1.38] 0.166 | 1.17 (0.15) [0.91;1.5] 0.211 |
| Epilepsy | ------------ | ------------ | 1.04 (0.11) [0.85;1.27] 0.734 | 1.04 (0.15) [0.78;1.38] 0.803 | ------------ | ------------ |
| CHD | 0.85 (0.05) [0.77;0.95] 0.004 | 0.83 (0.06) [0.73;0.95] 0.008 | 1.01 (0.06) [0.9;1.13] 0.871 | 1.02 (0.08) [0.88;1.20] 0.766 | ------------ | ------------ |
| Heart Failure | 1.09 (0.06) [0.98;1.20] 0.102 | 1.10 (0.07) [0.97;1.24] 0.154 | 1.08 (0.06) [0.96;1.21] 0.186 | 1.08 (0.09) [0.92;1.28] 0.363 | 1.01 (0.10) [0.83;1.23] 0.919 | 0.98 (0.13) [0.76;1.26] 0.879 |
| Multiple sclerosis | ------------ | ------------ | 0.74 (0.10) [0.57;0.98] 0.033 | 0.59 (0.15) [0.36;0.97] 0.035 | ------------ | ------------ |
| Parkinson’s | 1.24 (0.11) [1.03;1.48] 0.019 | 1.20 (0.14) [0.95;1.51] 0.120 | 1.09 (0.15) [0.83;1.42] 0.554 | 1.09 (0.20) [0.75;1.57] 0.664 | ------------ | ------------ |
| Renal Failure | 1.03 (0.05) [0.94;1.13] 0.513 | 1.06 (0.06) [0.94;1.19] 0.362 | 1.05 (0.06) [0.94;1.17] 0.420 | 1.08 (0.09) [0.92;1.26] 0.348 | 1.12 (0.12) [0.9;1.38] 0.306 | 1.14 (0.16) [0.87;1.49] 0.346 |
| Diseases of blood | 1.05 (0.05) [0.96;1.15] 0.275 | 1.05 (0.06) [0.94;1.18] 0.363 | ------------ | ------------ | ------------ | ------------ |
| Diabetes | ------------ | ------------ | ------------ | ------------ | 1.21 (0.11) [1.01;1.45] 0.043 | 1.24 (0.14) [0.99;1.55] 0.061 |
| Constant | 15.93 (46.90) [0.05;5098.92] 0.347 | 0.19 (0.68) [0.00;224.04] 0.644 | 285486.5 (1267507) [47.47; 1.72E+09] 0.005 | 899.53 (5743.23) [0.00;0.00] 0.287 | 20700000000000 (186000000000000) [500612.1;8.6E+20] 0.001 | 2230000000000 (25100000000000) [559.85;8.85E+21] 0.012 |

# driven mainly by non-elective hospital care; Note the HAH unit costs in site one were £628.34 per admission to HAH and have been added to the costs during the episode.

For peer review only

Association of hospital-at-home with mortality risk during study period (after propensity score matching)

| | site one (n=13,267) | site two (n=4,771) | site three (n=2110) |
|---|---|---|---|
| | coefficient (se) [95%CI] p value | coefficient (se) [95%CI] p value | coefficient (se) [95%CI] p value |
| HAH | 1.09 (0.05) [1.00;1.19] 0.059 | 1.29 (0.07) [1.15;1.44] <0.0010 | 1.27 (0.12) [1.06;1.54] 0.011 |
| Admission date | 1.00 (0.00) [1.00;1.00] 0.842 | 1.00 (0.00) [1.00;1.00] 0.100 | 1 (0) [1;1] 0.687 |
| ICD10 primary | 1.00 (0.00) [1.00;1.00] <0.001 | 1.00 (0.00) [1.00;1.00] 0.001 | 1 (0) [1;1] 0.006 |
| ICD10 secondary | 1.00 (0.00) [1.00;1.00] <0.001 | 1.00 (0.00) [1.00;1.00] 0.023 | 1 (0) [1;1] 0.359 |
| 2yrs pre AE costs | 1.00 (0.00) [1.00;1.00] 0.640 | 1.00 (0.00) [1.00;1.00] 0.153 | 1 (0) [1;1] 0.027 |
| 2yrs pre elective costs | 1.00 (0.00) [1.00;1.00] 0.487 | 1.00 (0.00) [1.00;1.00] 0.462 | 1 (0) [1;1] 0.079 |
| 2yrs pre non-elective costs | 1.00 (0.00) [1.00;1.00] 0.001 | 1.00 (0.00) [1.00;1.00] 0.007 | 1 (0) [1;1] 0.052 |
| 2yrs pre day case costs | 1.00 (0.00) [1.00;1.00] <0.001 | 1.00 (0.00) [1.00;1.00] 0.001 | 1 (0) [1;1] 0.903 |
| 2yrs pre geriatric ward costs | 1.00 (0.00) [1.00;1.00] 0.022 | 1.00 (0.00) [1.00;1.00] <0.001 | 1 (0) [1;1] 0.338 |
| 2yrs pre mental ward costs | 1.00 (0.00) [1.00;1.00] 0.419 | 1.00 (0.00) [1.00;1.00] 0.943 | 1 (0) [1;1] 0 |
| 2yrs pre outpatient costs | 1.00 (0.00) [1.00;1.00] 0.091 | 1.00 (0.00) [1.00;1.00] 0.882 | 1 (0) [1;1] 0.001 |
| 2yrs pre medication costs | 1.00 (0.00) [1.00;1.00] 0.044 | 1.00 (0.00) [1.00;1.00] 0.037 | 1 (0) [1;1] 0 |
| Number of LTCs | 1.03 (0.02) [0.99;1.07] 0.120 | 0.96 (0.02) [0.92;1.01] 0.107 | 1.07 (0.04) [1;1.14] 0.048 |
| Age on admission | 1.04 (0) [1.03;1.04] <0.001 | 1.03 (0.00) [1.02;1.04] <0.001 | 1.04 (0.01) [1.02;1.05] 0 |
| Male | 1.12 (0.05) [1.01;1.22] 0.017 | 1.23 (0.08) [1.09;1.39] 0.001 | 1.37 (0.14) [1.12;1.67] 0.002 |
| SES | 0.97 (0.01) [0.95;0.99] 0.001 | 0.98 (0.01) [0.96;1.00] 0.088 | 1.01 (0.02) [0.98;1.05] 0.483 |
| Arthritis | 0.86 (0.05) [0.77;0.97] 0.008 | ------------ | ------------ |
| Atrial Fibrillation | ------------ | 1.11 (0.08) [0.97;1.28] 0.133 | ------------ |
| Cancer | ------------ | 1.86 (0.12) [1.64;2.11] <0.001 | ------------ |
| CVD | 0.94 (0.06) [0.83;1.05] 0.276 | 1.06 (0.08) [0.92;1.22] 0.438 | 0.95 (0.12) [0.74;1.21] 0.673 |
| Liver disease | 1.33 (0.16) [1.04;1.67] 0.015 | ------------ | ------------ |
| Dementia | 1.11 (0.06) [1.00;1.25] 0.058 | 1.59 (0.11) [1.39;1.82] <0.001 | 1.31 (0.16) [1.03;1.67] 0.025 |
| Epilepsy | | 1.19 (0.17) [0.91;1.57] 0.207 | ------------ |
| CHD | 0.91 (0.05) [0.82;1.03] 0.114 | 0.93 (0.07) [0.80;1.08] 0.345 | ------------ |
| Heart Failure | 1.13 (0.07) [1.00;1.28] 0.052 | 1.35 (0.11) [1.15;1.57] <0.001 | 1.16 (0.15) [0.9;1.5] 0.256 |
| Multiple sclerosis | ------------ | 1.54 (0.39) [0.94;2.52] 0.086 | ------------ |
| Parkinson's | 1.11 (0.13) [0.86;1.39] 0.374 | 0.93 (0.17) [0.65;1.33] 0.678 | ------------ |
| Renal Failure | 1.07 (0.07) [0.95;1.21] 0.292 | 1.35 (0.10) [1.16;1.56] <0.001 | 0.93 (0.12) [0.72;1.2] 0.571 |
| Diseases of blood | 0.93 (0.05) [0.85;1.06] 0.201 | ------------ | ------------ |
| Diabetes | ------------ | ------------ | 0.74 (0.1) [0.57;0.97] 0.026 |
| Constant | 0.01 (0.04) [0.00;7.06] 0.174 | 0.00 (0.00) [0.00;0.18] 0.025 | 0 (0) [0;319640.8] 0.405 |

For peer review only - http://bmjopen.bmj.com/site/about/guidelines.xhtml

Results of the subgroup analysis including only patients with dementia (costs)

| | site one (n=2,321) | | site two (n=1,053) | | site three (n=280) | |
|---|---|---|---|---|---|---|
| | Follow-up period<br>coefficient (se) [95%CI] p value | Total costs in 6 months after discharge<br>coefficient (se) [95%CI] p value | Follow-up period<br>coefficient (se) [95%CI] p value | Total costs in 6 months after discharge<br>coefficient (se) [95%CI] p value | Follow-up period<br>coefficient (se) [95%CI] p value | Total costs in 6 months after discharge<br>coefficient (se) [95%CI] p value |
| HAH (hospital) | 0.76 (0.05) [0.66;0.87] 0 | 1.18 (0.11) [0.99;1.41] 0.071 | 0.76 (0.06) [0.66;0.88] 0 | 0.75 (0.09) [0.59;0.96] 0.021 | 0.87 (0.15) [0.63;1.21] 0.409 | 1.58 (0.41) [0.95;2.63] 0.078 |
| Admission date | 1 (0) [1;1] 0.528 | 1.00 (0.00) [1.00;1.00] 0.329 | 1 (0) [1;1] 0.513 | 1.00 (0.00) [1.00;1.00] 0.532 | 1 (0) [1;1] 0.002 | 1 (0) [0.99;1] 0.003 |
| ICD10 primary | 1 (0) [1;1] 0.025 | 1.00 (0.00) [1.00;1.00] 0.003 | 1 (0) [1;1] 0.079 | 1.00 (0.00) [1.00;1.00] 0.008 | 1 (0) [1;1] 0.666 | 1 (0) [1;1] 0.123 |
| ICD10 secondary | 1 (0) [1;1] 0.027 | 1.00 (0.00) [1.00;1.00] 0.086 | ------------ | ------------ | 1 (0) [1;1] 0.946 | 1 (0) [1;1] 0.594 |
| 2yrs pre AE costs | 1 (0) [1;1] 0.063 | 1.00 (0.00) [1.00;1.00] 0.021 | 1 (0) [1;1] 0.979 | 1.00 (0.00) [1.00;1.00] 0.93 | 1 (0) [1;1] 0.57 | 1 (0) [1;1] 0.331 |
| 2yrs pre elective costs | 1 (0) [1;1] 0.913 | 1.00 (0.00) [1.00;1.00] 0.708 | 1 (0) [1;1] 0.979 | 1.00 (0.00) [1.00;1.00] 0.889 | 1 (0) [1;1] 0.115 | 1 (0) [1;1] 0.208 |
| 2yrs pre non-elective costs | 1 (0) [1;1] 0.564 | 1.00 (0.00) [1.00;1.00] 0.605 | 1 (0) [1;1] 0.031 | 1.00 (0.00) [1.00;1.00] 0.008 | 1 (0) [1;1] 0.888 | 1 (0) [1;1] 0.639 |
| 2yrs pre day case costs | 1 (0) [1;1] 0.455 | 1.00 (0.00) [1.00;1.00] 0.632 | 1 (0) [1;1] 0.725 | 1.00 (0.00) [1.00;1.00] 0.307 | 1 (0) [1;1] 0.1 | 1 (0) [1;1] 0.279 |
| 2yrs pre geriatric ward costs | 1 (0) [1;1] 0.233 | 1.00 (0.00) [1.00;1.00] 0.566 | 1 (0) [1;1] 0.012 | 1.00 (0.00) [1.00;1.00] 0.003 | 1 (0) [1;1] 0.907 | 1 (0) [1;1] 0.952 |
| 2yrs pre mental ward costs | 1 (0) [1;1] 0.343 | 1.00 (0.00) [1.00;1.00] 0.335 | 1 (0) [1;1] 0.084 | 1.00 (0.00) [1.00;1.00] 0.042 | 1 (0) [1;1] 0.01 | 1 (0) [1;1] 0.021 |
| 2yrs pre outpatient costs | 1 (0) [1;1] 0.066 | 1.00 (0.00) [1.00;1.00] 0.082 | 1 (0) [1;1] 0.001 | 1.00 (0.00) [1.00;1.00] 0.001 | 1 (0) [1;1] 0.685 | 1 (0) [1;1] 0.403 |
| 2yrs pre medication costs | 1 (0) [1;1] 0.306 | 1.00 (0.00) [1.00;1.00] 0.316 | 1 (0) [1;1] 0.13 | 1.00 (0.00) [1.00;1.00] 0.265 | 1 (0) [1;1] 0.042 | 1 (0) [1;1] 0.044 |
| Died within 6months | 0.81 (0.06) [0.7;0.94] 0.005 | 0.70 (0.07) [0.58;0.85] <0.001 | 0.89 (0.07) [0.76;1.03] 0.118 | 0.73 (0.09) [0.58;0.93] 0.011 | 0.66 (0.13) [0.45;0.96] 0.031 | 0.44 (0.13) [0.25;0.77] 0.004 |
| Number of LTCs | 1.06 (0.03) [1;1.12] 0.069 | 1.07 (0.04) [1.00;1.16] 0.063 | 1.08 (0.03) [1.02;1.14] 0.006 | 1.15 (0.05) [1.05;1.26] 0.003 | 1.04 (0.06) [0.94;1.16] 0.443 | 1.01 (0.08) [0.86;1.18] 0.935 |
| Age on admission | 0.99 (0.01) [0.98;1] 0.094 | 0.98 (0.01) [0.97;1.00] 0.015 | 0.98 (0.01) [0.97;1] 0.007 | 0.97 (0.01) [0.95;0.99] 0.003 | 1 (0.01) [0.98;1.03] 0.933 | 1 (0.02) [0.97;1.03] 0.946 |
| Male | 1.13 (0.08) [0.99;1.31] 0.076 | 1.14 (0.11) [0.95;1.37] 0.151 | 0.95 (0.07) [0.82;1.11] 0.511 | 0.95 (0.12) [0.74;1.22] 0.679 | 1.05 (0.17) [0.76;1.43] 0.78 | 1.07 (0.26) [0.67;1.71] 0.774 |
| SES | 1.01 (0.01) [0.98;1.04] 0.693 | 1.01 (0.02) [0.97;1.04] 0.77 | 1.03 (0.01) [1;1.05] 0.053 | 1.06 (0.02) [1.01;1.10] 0.010 | 1.03 (0.03) [0.97;1.09] 0.3 | 1.04 (0.04) [0.96;1.12] 0.3 |
| Atrial Fibrillation | ------------ | ------------ | 1.03 (0.09) [0.87;1.23] 0.722 | 1.00 (0.14) [0.77;1.31] 0.986 | ------------ | ------------ |
| Arthritis | 1.02 (0.09) [0.86;1.2] 0.833 | 1.02 (0.11) [0.83;1.25] 0.862 | ------------ | ------------ | ------------ | ------------ |
| Cancer | ------------ | ------------ | 1.04 (0.1) [0.87;1.24] 0.679 | 1.06 (0.16) [0.79;1.43] 0.688 | ------------ | ------------ |
| CVD | 0.92 (0.07) [0.78;1.08] 0.3 | 0.91 (0.1) [0.74;1.12] 0.374 | 0.98 (0.08) [0.83;1.16] 0.845 | 0.95 (0.14) [0.72;1.26] 0.741 | 1.39 (0.28) [0.94;2.06] 0.103 | 1.65 (0.48) [0.93;2.91] 0.085 |
| Liver disease | 0.8 (0.12) [0.59;1.08] 0.138 | 0.8 (0.16) [0.54;1.20] 0.286 | ------------ | ------------ | ------------ | ------------ |
| CHD | 1.01 (0.09) [0.85;1.2] 0.917 | 1.05 (0.12) [0.84;1.30] 0.688 | 0.94 (0.09) [0.78;1.12] 0.482 | 0.98 (0.14) [0.74;1.30] 0.891 | ------------ | ------------ |
| Epilepsy | ------------ | ------------ | 0.97 (0.15) [0.72;1.3] 0.842 | 0.78 (0.16) [0.53;1.16] 0.221 | ------------ | ------------ |
| Heart Failure | 1.03 (0.11) [0.83;1.27] 0.818 | 1.02 (0.14) [0.79;1.33] 0.878 | 0.92 (0.11) [0.73;1.15] 0.452 | 0.90 (0.17) [0.62;1.29] 0.558 | 0.83 (0.19) [0.53;1.3] 0.409 | 1.16 (0.42) [0.57;2.37] 0.687 |
| Multiple sclerosis | ------------ | ------------ | 0.4 (0.06) [0.29;0.54] 0 | 0.18 (0.07) [0.09;0.37] <0.001 | ------------ | ------------ |
| Parkinson’s | 1.13 (0.15) [0.88;1.46] 0.333 | 1.00 (0.17) [0.72;1.39] 0.992 | 0.87 (0.14) [0.63;1.18] 0.365 | 0.68 (0.20) [0.39;1.20] 0.188 | ------------ | ------------ |
| Renal Failure | 1.03 (0.1) [0.85;1.24] 0.769 | 1.12 (0.14) [0.88;1.42] 0.354 | 0.9 (0.09) [0.75;1.09] 0.296 | 0.82 (0.13) [0.60;1.12] 0.203 | 1.2 (0.24) [0.81;1.78] 0.354 | 1.25 (0.35) [0.72;2.17] 0.435 |
| Diseases of blood | 0.93 (0.08) [0.79;1.11] 0.437 | 0.90 (0.1) [0.73;1.11] 0.337 | ------------ | ------------ | ------------ | ------------ |
| Diabetes | ------------ | ------------ | ------------ | ------------ | 0.85 (0.18) [0.55;1.3] 0.449 | 0.92 (0.26) [0.52;1.6] 0.756 |
| Constant | 469.5 (2319.98) [0.03;7547051] 0.213 | 22.71 (140.52) [0;4194325] 0.614 | 2796754 (19900000) [2.38;3290000000000] 0.037 | 40500000 (472000000) [0;3290000000000000000] 0.132 | 2.82E+29 (5.36E+30) [18000000000000;4.43E+45] 0 | 3.34E+38 (9.1E+39) [2100000000000000;5.29E+61] 0.001 |



For peer review only - http://bmjopen.bmj.com/site/about/guidelines.xhtml

Results of the subgroup analysis including only patients with dementia (mortality risk)

| | site one (n=2,321) | site two (n=1,053) | site three (n=280) |
|---|---|---|---|
| | Mortality rate during follow-up coefficient (se) [95%CI] p value | Mortality rate during follow-up coefficient (se) [95%CI] p value | Mortality rate during follow-up coefficient (se) [95%CI] p value |
| HAH (hospital) | 1.05 (0.09) [0.89;1.24] 0.594 | 1.41 (0.12) [1.19;1.67] <0.001 | 1.65 (0.32) [1.12;2.41] 0.011 |
| Admission date | 1.00 (0.00) [1.00;1.00] 0.19 | 1.00 (0.00) [1.00;1.00] 0.001 | 1 (0) [1;1] 0.788 |
| ICD10 primary | 1.00 (0.00) [1.00;1.00] <0.001 | 1.00 (0.00) [1.00;1.00] 0.001 | 1 (0) [1;1] 0.14 |
| ICD10 secondary | 1.00 (0.00) [1.00;1.00] 0.207 | ------------ | 1 (0) [1;1] 0.979 |
| 2yrs pre AE costs | 1.00 (0.00) [1.00;1.00] 0.251 | 1.00 (0.00) [1.00;1.00] 0.609 | 1 (0) [1;1] 0.029 |
| 2yrs pre elective costs | 1.00 (0.00) [1.00;1.00] 0.735 | 1.00 (0.00) [1.00;1.00] 0.129 | 1 (0) [1;1] 0.554 |
| 2yrs pre non-elective costs | 1.00 (0.00) [1.00;1.00] 0.173 | 1.00 (0.00) [1.00;1.00] 0.484 | 1 (0) [1;1] 0.814 |
| 2yrs pre day case costs | 1.00 (0.00) [1.00;1.00] 0.088 | 1.00 (0.00) [1.00;1.00] 0.004 | 1 (0) [1;1] 0.896 |
| 2yrs pre geriatric ward costs | 1.00 (0.00) [1.00;1.00] 0.644 | 1.00 (0.00) [1.00;1.00] <0.001 | 1 (0) [1;1] 0.783 |
| 2yrs pre mental ward costs | 1.00 (0.00) [1.00;1.00] 0.569 | 1.00 (0.00) [1.00;1.00] 0.112 | 1 (0) [1;1] 0 |
| 2yrs pre outpatient costs | 1.00 (0.00) [1.00;1.00] 0.070 | 1.00 (0.00) [1.00;1.00] 0.167 | 1 (0) [1;1] 0 |
| 2yrs pre medication costs | 1.00 (0.00) [1.00;1.00] 0.004 | 1.00 (0.00) [1.00;1.00] 0.156 | 1 (0) [1;1] 0.011 |
| Died within 6months | ------------ | ------------ | ------------ |
| Number of LTCs | 0.94 (0.03) [0.88;1.01] 0.113 | 0.95 (0.03) [0.89;1.01] 0.115 | 0.98 (0.07) [0.86;1.13] 0.827 |
| Age on admission | 1.04 (0.01) [1.02;1.05] <0.001 | 1.03 (0.01) [1.01;1.04] <0.001 | 1.04 (0.02) [1;1.07] 0.024 |
| Male | 1.19 (0.11) [0.99;1.42] 0.063 | 1.17 (0.10) [0.99;1.38] 0.070 | 1.18 (0.25) [0.78;1.79] 0.43 |
| SES | 0.97 (0.02) [0.94;1.01] 0.134 | 1.00 (0.02) [0.97;1.03] 0.991 | 0.96 (0.04) [0.88;1.04] 0.3 |
| Atrial Fibrillation | ------------ | 1.03 (0.11) [0.85;1.26] 0.75 | ------------ |
| Arthritis | 1.06 (0.11) [0.86;1.30] 0.600 | ------------ | ------------ |
| Cancer | ------------ | 1.40 (0.13) [1.16;1.68] <0.001 | ------------ |
| CVD | 1.55 (0.41) [0.92;2.61] 0.099 | 1.14 (0.11) [0.94;1.39] 0.176 | 1.02 (0.25) [0.63;1.65] 0.925 |
| Liver disease | 0.98 (0.11) [0.79;1.21] 0.845 | ------------ | ------------ |
| CHD | 1.21 (0.16) [0.94;1.56] 0.135 | 0.99 (0.10) [0.81;1.20] 0.885 | ------------ |
| Epilepsy | ------------ | 1.26 (0.19) [0.94;1.70] 0.120 | ------------ |
| Heart Failure | 1.21 (0.16) [0.94;1.56] 0.135 | 1.33 (0.17) [1.04;1.70] 0.023 | 1.88 (0.49) [1.12;3.14] 0.017 |
| Multiple sclerosis | ------------ | 0.96 (0.51) [0.34;2.72] 0.932 | ------------ |
| Parkinson's | 1.26 (0.22) [0.9;1.78] 0.180 | 1.04 (0.20) [0.71;1.51] 0.848 | ------------ |
| Renal Failure | 1.06 (0.12) [0.84;1.32] 0.637 | 1.15 (0.12) [0.93;1.41] 0.192 | 0.56 (0.16) [0.32;0.97] 0.037 |
| Diseases of blood | 0.96 (0.11) [0.77;1.19] 0.709 | ------------ | ------------ |
| Diabetes | ------------ | ------------ | 0.6 (0.2) [0.32;1.15] 0.123 |
| Constant | 0.00 (0.00) [0.00;1.37] 0.057 | 0.00 (0.00) [0.00;0.00] <0.001 | 0 (0) [0;1810000000000000] 0.652 |



For peer review only - http://bmjopen.bmj.com/site/about/guidelines.xhtml

Results of the subgroup analysis excluding those who had died

| | site one (n=10,132) | | site two (n=3,584) | | site three (n=1691) | |
|---|---|---|---|---|---|---|
| | Follow-up period | Total costs in 6 months after discharge | Follow-up period | Total costs in 6 months after discharge | Follow-up period | Total costs in 6 months after discharge |
| | coefficient (se) [95%CI] p value | coefficient (se) [95%CI] p value | coefficient (se) [95%CI] p value | coefficient (se) [95%CI] p value | coefficient (se) [95%CI] p value | coefficient (se) [95%CI] p value |
| HAH (hospital) | 0.85 (0.04) [0.77;0.94] 0.002 | 1.23 (0.08) [1.08;1.4] 0.002 | 1.11 (0.06) [1;1.25] 0.058 | 1.17 (0.10) [0.99;1.38] 0.070 | 1.20 (0.11) [1;1.43] 0.046 | 1.71 (0.20) [1.36;2.15] <0.001 |
| Admission date | 1 (0) [1;1] 0.076 | 1.00 (0.00) [1.00;1.00] 0.032 | 1 (0) [1;1] 0.833 | 1.00 (0.00) [1.00;1.00] 0.337 | 1 (0) [1;1] 0.075 | 1 (0) [1;1] 0.282 |
| ICD10 primary | 1 (0) [1;1] 0.692 | 1.00 (0.00) [1.00;1.00] 0.993 | 1 (0) [1;1] 0.126 | 1.00 (0.00) [1.00;1.00] 0.038 | 1 (0) [1;1] 0.282 | 1 (0) [1;1] 0.279 |
| ICD10 secondary | 1 (0) [1;1] 0.817 | 1.00 (0.00) [1.00;1.00] 0.473 | 1 (0) [1;1] 0.014 | 1.00 (0.00) [1.00;1.00] 0.024 | 1 (0) [1;1] 0.724 | 1 (0) [1;1] 0.801 |
| 2yrs pre AE costs | 1 (0) [1;1] 0.08 | 1.00 (0.00) [1.00;1.00] 0.012 | 1 (0) [1;1] 0.461 | 1.00 (0.00) [1.00;1.00] 0.135 | 1 (0) [1;1] 0.435 | 1 (0) [1;1] 0.761 |
| 2yrs pre elective costs | 1 (0) [1;1] 0.015 | 1.00 (0.00) [1.00;1.00] 0.046 | 1 (0) [1;1] 0.576 | 1.00 (0.00) [1.00;1.00] 0.429 | 1 (0) [1;1] 0.63 | 1 (0) [1;1] 0.725 |
| 2yrs pre non-elective costs | 1 (0) [1;1] 0 | 1.00 (0.00) [1.00;1.00] <0.001 | 1 (0) [1;1] 0.651 | 1.00 (0.00) [1.00;1.00] 0.700 | 1 (0) [1;1] 0.199 | 1 (0) [1;1] 0.01 |
| 2yrs pre day case costs | 1 (0) [1;1] 0.416 | 1.00 (0.00) [1.00;1.00] 0.158 | 1 (0) [1;1] 0.057 | 1.00 (0.00) [1.00;1.00] 0.023 | 1 (0) [1;1] 0.068 | 1 (0) [1;1] 0.064 |
| 2yrs pre geriatric ward costs | 1 (0) [1;1] 0.031 | 1.00 (0.00) [1.00;1.00] 0.029 | 1 (0) [1;1] 0.625 | 1.00 (0.00) [1.00;1.00] 0.806 | 1 (0) [1;1] 0.484 | 1 (0) [1;1] 0.103 |
| 2yrs pre mental ward costs | 1 (0) [1;1] 0.206 | 1.00 (0.00) [1.00;1.00] 0.166 | 1 (0) [1;1] 0.009 | 1.00 (0.00) [1.00;1.00] 0.020 | 1 (0) [1;1] 0.01 | 1 (0) [1;1] 0.004 |
| 2yrs pre outpatient costs | 1 (0) [1;1] 0.236 | 1.00 (0.00) [1.00;1.00] 0.187 | 1 (0) [1;1] 0.748 | 1.00 (0.00) [1.00;1.00] 0.802 | 1 (0) [1;1] 0.798 | 1 (0) [1;1] 0.908 |
| 2yrs pre medication costs | 1 (0) [1;1] 0.399 | 1.00 (0.00) [1.00;1.00] 0.383 | 1 (0) [1;1] 0.011 | 1.00 (0.00) [1.00;1.00] 0.016 | 1 (0) [1;1] 0.37 | 1 (0) [1;1] 0.77 |
| Number of LTCs | 1.08 (0.02) [1.04;1.12] 0 | 1.12 (0.03) [1.07;1.18] <0.001 | 1.03 (0.02) [0.99;1.08] 0.169 | 1.06 (0.04) [0.99;1.13] 0.076 | 1.06 (0.03) [1.01;1.13] 0.032 | 1.09 (0.04) [1.01;1.17] 0.026 |
| Age on admission | 1.01 (0) [1;1.01] 0.025 | 1.01 (0.00) [1.00;1.02] 0.048 | 1.01 (0) [1;1.01] 0.054 | 1.01 (0.01) [1.00;1.02] 0.254 | 1.02 (0.01) [1;1.03] 0.019 | 1.01 (0.01) [0.99;1.03] 0.171 |
| Male | 1.11 (0.06) [1;1.22] 0.051 | 1.12 (0.07) [0.99;1.26] 0.085 | 0.94 (0.06) [0.83;1.07] 0.353 | 0.97 (0.09) [0.80;1.17] 0.752 | 0.97 (0.09) [0.8;1.16] 0.716 | 1 (0.12) [0.79;1.26] 0.974 |
| SES | 1 (0.01) [0.98;1.02] 0.965 | 1 (0.01) [0.98;1.03] 0.778 | 1.02 (0.01) [1;1.04] 0.081 | 1.03 (0.01) [1.00;1.06] 0.023 | 1 (0.02) [0.96;1.03] 0.822 | 1 (0.02) [0.95;1.05] 0.951 |
| Atrial Fibrillation | ------------ | ------------ | 1.07 (0.07) [0.94;1.21] 0.305 | 1.09 (0.10) [0.92;1.29] 0.335 | ------------ | ------------ |
| Arthritis | 0.99 (0.05) [0.89;1.1] 0.889 | 0.96 (0.06) [0.85;1.1] 0.584 | ------------ | ------------ | ------------ | ------------ |
| Cancer | ------------ | ------------ | 1 (0.07) [0.88;1.15] 0.961 | 1.01 (0.10) [0.84;1.23] 0.899 | ------------ | ------------ |
| CVD | 1.04 (0.07) [0.91;1.2] 0.552 | 1.00 (0.09) [0.85;1.19] 0.956 | 1.14 (0.08) [1;1.3] 0.058 | 1.14 (0.11) [0.95;1.36] 0.174 | 1.12 (0.14) [0.88;1.43] 0.367 | 1.1 (0.17) [0.81;1.5] 0.531 |
| Liver disease | 1.35 (0.2) [1.01;1.8] 0.045 | 1.31 (0.21) [0.95;1.81] 0.097 | ------------ | ------------ | ------------ | ------------ |
| Dementia | 1.16 (0.07) [1.04;1.3] 0.009 | 1.17 (0.08) [1.01;1.35] 0.033 | 1.08 (0.07) [0.96;1.22] 0.195 | 1.11 (0.10) [0.93;1.31] 0.244 | 1.37 (0.16) [1.09;1.73] 0.008 | 1.49 (0.23) [1.09;2.02] 0.011 |
| CHD | 0.82 (0.06) [0.72;0.94] 0.004 | 0.79 (0.07) [0.67;0.93] 0.004 | 1.01 (0.07) [0.87;1.16] 0.941 | 1.03 (0.10) [0.85;1.24] 0.799 | ------------ | ------------ |
| Epilepsy | ------------ | ------------ | 1.08 (0.12) [0.86;1.35] 0.518 | 1.09 (0.17) [0.80;1.48] 0.581 | ------------ | ------------ |
| Heart Failure | 1.1 (0.07) [0.97;1.25] 0.131 | 1.08 (0.08) [0.93;1.26] 0.293 | 1.08 (0.08) [0.94;1.24] 0.287 | 1.07 (0.11) [0.88;1.31] 0.491 | 1.05 (0.13) [0.82;1.34] 0.719 | 1.01 (0.16) [0.74;1.39] 0.932 |
| Multiple sclerosis | ------------ | ------------ | 0.72 (0.14) [0.49;1.06] 0.095 | 0.66 (0.21) [0.35;1.25] 0.202 | ------------ | ------------ |
| Parkinson's | 1.19 (0.1) [1;1.41] 0.05 | 1.15 (0.13) [0.93;1.43] 0.19 | 1.22 (0.18) [0.91;1.64] 0.193 | 1.34 (0.27) [0.91;1.98] 0.139 | ------------ | ------------ |
| Renal Failure | 1.01 (0.06) [0.89;1.14] 0.911 | 1.00 (0.07) [0.87;1.16] 0.949 | 1.06 (0.08) [0.92;1.22] 0.443 | 1.06 (0.11) [0.86;1.29] 0.602 | 1.12 (0.15) [0.86;1.46] 0.411 | 1.19 (0.2) [0.85;1.66] 0.317 |
| Diseases of blood | 1.04 (0.06) [0.94;1.16] 0.414 | 1.04 (0.07) [0.92;1.19] 0.516 | ------------ | ------------ | ------------ | ------------ |
| Diabetes | ------------ | ------------ | ------------ | ------------ | 1.33 (0.15) [1.07;1.65] 0.01 | 1.37 (0.19) [1.04;1.81] 0.026 |
| Constant | 3.67 (13.85) [0;5959] 0.73 | 0.07 (0.31) [0;592.13] 0.558 | 1064.79 (5943.4) [0.02;60000000] 0.212 | 0.89 (6.96) [0;4301665] 0.988 | 101000000000 (1050000000000) [149.57;68100000000000000000] 0.015 | 1320000000 (18000000000) [0;5.67E+20] 0.124 |

For peer review only



For peer review only - http://bmjopen.bmj.com/site/about/guidelines.xhtml

Results of the sensitivity analysis

| | site one (n=13,267) | | site two (n=4,769) | | site three (n=2110) | |
|---|---|---|---|---|---|---|
| | Total costs in follow-up (50% higher HAH unit costs) coefficient (se) [95%CI] p value | Total costs in follow-up (50% lower HAH unit costs) coefficient (se) [95%CI] p value | Total costs in follow-up (50% higher HAH unit costs) coefficient (se) [95%CI] p value | Total costs in follow-up (50% lower HAH unit costs) coefficient (se) [95%CI] p value | Total costs in follow-up (50% higher HAH unit costs) coefficient (se) [95%CI] p value | Total costs in follow-up (50% lower HAH unit costs) coefficient (se) [95%CI] p value |
| HAH (hospital) | 0.87 (0.03) [0.81;0.94] 0.001 | 0.77 (0.03) [0.71;0.84] 0 | 1.18 (0.05) [1.09;1.28] 0 | 0.81 (0.04) [0.74;0.9] 0 | 1.23 (0.09) [1.07;1.42] 0.004 | 1.07 (0.09) [0.91;1.25] 0.399 |
| Admission date | 1 (0) [1;1] 0.071 | 1 (0) [1;1] 0.048 | 1 (0) [1;1] 0.489 | 1 (0) [1;1] 0.3 | 1 (0) [1;1] 0.007 | 1 (0) [1;1] 0.012 |
| ICD10 primary | 1 (0) [1;1] 0.649 | 1 (0) [1;1] 0.671 | 1 (0) [1;1] 0.001 | 1 (0) [1;1] 0.001 | 1 (0) [1;1] 0.167 | 1 (0) [1;1] 0.16 |
| ICD10 secondary | 1 (0) [1;1] 0.588 | 1 (0) [1;1] 0.701 | 1 (0) [1;1] 0.148 | 1 (0) [1;1] 0.145 | 1 (0) [1;1] 0.875 | 1 (0) [1;1] 0.909 |
| 2yrs pre AE costs | 1 (0) [1;1] 0.223 | 1 (0) [1;1] 0.261 | 1 (0) [1;1] 0.687 | 1 (0) [1;1] 0.561 | 1 (0) [1;1] 0.307 | 1 (0) [1;1] 0.267 |
| 2yrs pre elective costs | 1 (0) [1;1] 0.909 | 1 (0) [1;1] 0.904 | 1 (0) [1;1] 0.537 | 1 (0) [1;1] 0.657 | 1 (0) [1;1] 0.896 | 1 (0) [1;1] 0.813 |
| 2yrs pre non-elective costs | 1 (0) [1;1] 0 | 1 (0) [1;1] 0 | 1 (0) [1;1] 0.919 | 1 (0) [1;1] 0.458 | 1 (0) [1;1] 0.015 | 1 (0) [1;1] 0.021 |
| 2yrs pre day case costs | 1 (0) [1;1] 0.099 | 1 (0) [1;1] 0.097 | 1 (0) [1;1] 0.006 | 1 (0) [1;1] 0.004 | 1 (0) [1;1] 0.131 | 1 (0) [1;1] 0.148 |
| 2yrs pre geriatric ward costs | 1 (0) [1;1] 0.006 | 1 (0) [1;1] 0.005 | 1 (0) [1;1] 0.002 | 1 (0) [1;1] 0 | 1 (0) [1;1] 0.562 | 1 (0) [1;1] 0.713 |
| 2yrs pre mental ward costs | 1 (0) [1;1] 0.905 | 1 (0) [1;1] 0.854 | 1 (0) [1;1] 0.005 | 1 (0) [1;1] 0.02 | 1 (0) [1;1] 0.09 | 1 (0) [1;1] 0.132 |
| 2yrs pre outpatient costs | 1 (0) [1;1] 0.086 | 1 (0) [1;1] 0.088 | 1 (0) [1;1] 0.027 | 1 (0) [1;1] 0.026 | 1 (0) [1;1] 0.699 | 1 (0) [1;1] 0.675 |
| 2yrs pre medication costs | 1 (0) [1;1] 0.713 | 1 (0) [1;1] 0.892 | 1 (0) [1;1] 0.136 | 1 (0) [1;1] 0.236 | 1 (0) [1;1] 0.713 | 1 (0) [1;1] 0.663 |
| Died within 6months | 1.03 (0.04) [0.95;1.11] 0.492 | 1.02 (0.04) [0.94;1.12] 0.572 | 1.05 (0.04) [0.97;1.14] 0.252 | 1.05 (0.05) [0.95;1.16] 0.38 | 1.06 (0.08) [0.91;1.23] 0.474 | 1.06 (0.09) [0.89;1.25] 0.517 |
| Number of LTCs | 1.08 (0.02) [1.05;1.11] 0 | 1.09 (0.02) [1.05;1.13] 0 | 1.04 (0.02) [1;1.07] 0.033 | 1.04 (0.02) [0.99;1.08] 0.093 | 1.06 (0.02) [1.01;1.1] 0.016 | 1.06 (0.03) [1.01;1.11] 0.019 |
| Age on admission | 1 (0) [1;1.01] 0.323 | 1 (0) [1;1.01] 0.452 | 1 (0) [1;1.01] 0.788 | 1 (0) [0.99;1.01] 0.789 | 1.01 (0.01) [1;1.02] 0.037 | 1.01 (0.01) [1;1.02] 0.055 |
| Male | 1.09 (0.04) [1.01;1.18] 0.035 | 1.1 (0.05) [1.01;1.2] 0.034 | 0.96 (0.04) [0.88;1.04] 0.311 | 0.95 (0.06) [0.85;1.07] 0.382 | 0.97 (0.07) [0.84;1.12] 0.686 | 0.97 (0.08) [0.82;1.14] 0.704 |
| SES | 1 (0.01) [0.98;1.02] 0.979 | 1 (0.01) [0.98;1.02] 0.954 | 1.01 (0.01) [1;1.02] 0.17 | 1.01 (0.01) [0.99;1.03] 0.205 | 1 (0.01) [0.97;1.03] 0.887 | 1 (0.02) [0.97;1.03] 0.917 |
| Atrial Fibrillation | ------------ | ------------ | 1.08 (0.05) [0.98;1.18] 0.104 | 1.1 (0.06) [0.98;1.23] 0.094 | ------------ | ------------ |
| Arthritis | 0.96 (0.04) [0.89;1.05] 0.392 | 0.96 (0.05) [0.88;1.05] 0.403 | ------------ | ------------ | ------------ | ------------ |
| Cancer | ------------ | ------------ | 1.04 (0.05) [0.95;1.14] 0.426 | 1.03 (0.06) [0.92;1.16] 0.566 | ------------ | ------------ |
| CVD | 1.02 (0.05) [0.92;1.13] 0.743 | 1.02 (0.06) [0.91;1.14] 0.794 | 1.07 (0.05) [0.98;1.18] 0.146 | 1.08 (0.07) [0.96;1.22] 0.199 | 1.09 (0.11) [0.91;1.32] 0.352 | 1.11 (0.12) [0.9;1.37] 0.324 |
| Liver disease | 1.21 (0.13) [0.98;1.48] 0.073 | 1.23 (0.14) [0.98;1.53] 0.074 | ------------ | ------------ | ------------ | ------------ |
| Dementia | 1.07 (0.05) [0.97;1.17] 0.16 | 1.07 (0.05) [0.97;1.18] 0.2 | 1.02 (0.05) [0.93;1.11] 0.738 | 0.99 (0.06) [0.88;1.1] 0.795 | 1.14 (0.11) [0.95;1.37] 0.153 | 1.14 (0.12) [0.94;1.4] 0.18 |
| CHD | 0.86 (0.05) [0.77;0.95] 0.004 | 0.85 (0.05) [0.76;0.95] 0.005 | 1.07 (0.05) [0.97;1.18] 0.174 | 1.02 (0.06) [0.9;1.15] 0.785 | ------------ | ------------ |
| Epilepsy | ------------ | ------------ | 1.04 (0.1) [0.86;1.26] 0.664 | 1.02 (0.12) [0.82;1.28] 0.841 | ------------ | ------------ |
| Heart Failure | 1.09 (0.05) [0.99;1.2] 0.095 | 1.09 (0.06) [0.98;1.21] 0.11 | 1.07 (0.06) [0.96;1.19] 0.201 | 1.09 (0.07) [0.96;1.24] 0.177 | 1.01 (0.1) [0.83;1.22] 0.947 | 1.02 (0.11) [0.82;1.25] 0.885 |
| Multiple sclerosis | ------------ | ------------ | 0.76 (0.1) [0.59;0.98] 0.033 | 0.73 (0.11) [0.54;0.99] 0.046 | ------------ | ------------ |
| Parkinson's | 1.23 (0.11) [1.04;1.45] 0.018 | 1.24 (0.12) [1.03;1.49] 0.021 | 1.07 (0.14) [0.84;1.37] 0.582 | 1.11 (0.18) [0.81;1.52] 0.512 | ------------ | ------------ |
| Renal Failure | 1.04 (0.05) [0.95;1.13] 0.436 | 1.03 (0.05) [0.93;1.13] 0.601 | 1.04 (0.05) [0.94;1.15] 0.408 | 1.06 (0.07) [0.94;1.2] 0.366 | 1.11 (0.11) [0.91;1.36] 0.3 | 1.12 (0.13) [0.9;1.39] 0.317 |
| Diseases of blood | 1.05 (0.05) [0.97;1.14] 0.246 | 1.05 (0.05) [0.96;1.15] 0.308 | ------------ | ------------ | ------------ | ------------ |
| Diabetes | ------------ | ------------ | ------------ | ------------ | 1.2 (0.11) [1;1.42] 0.044 | 1.22 (0.12) [1.01;1.48] 0.042 |
| Constant | 26.62 (74.48) [0.11;6410.63] 0.241 | 8.84 (27.52) [0.02;3945.99] 0.484 | 295178.8 (1199605) [102.52;850000000] 0.002 | 1223534 (6192074) [60.23;24900000000] 0.006 | 14800000000000 (127000000000000) [776224.7;2.84E+20] 0 | 31000000000(2920000000) [292677.5;3.28E+21] 0.001 |

For peer review only



For peer review only - http://bmjopen.bmj.com/site/about/guidelines.xhtml

For peer review only



For peer review only - http://bmjopen.bmj.com/site/about/guidelines.xhtml

Appendix 4 STROBE Statement—checklist of items that should be included in reports of observational studies

| | Item No | Recommendation | Page |
|---|---|---|---|
| **Title and abstract** | 1 | (*a*) Indicate the study's design with a commonly used term in the title or the abstract | 1 |
| | | (*b*) Provide in the abstract an informative and balanced summary of what was done and what was found | 2 |
| **Introduction** | | | |
| Background/rationale | 2 | Explain the scientific background and rationale for the investigation being reported | 4 |
| Objectives | 3 | State specific objectives, including any prespecified hypotheses | 4 |
| **Methods** | | | |
| Study design | 4 | Present key elements of study design early in the paper | 5 |
| Setting | 5 | Describe the setting, locations, and relevant dates, including periods of recruitment, exposure, follow-up, and data collection | 5-6 |
| Participants | 6 | (*a*) *Cohort study*—Give the eligibility criteria, and the sources and methods of selection of participants. Describe methods of follow-up<br>*Case-control study*—Give the eligibility criteria, and the sources and methods of case ascertainment and control selection. Give the rationale for the choice of cases and controls<br>*Cross-sectional study*—Give the eligibility criteria, and the sources and methods of selection of participants | |
| | | (*b*) *Cohort study*—For matched studies, give matching criteria and number of exposed and unexposed<br>*Case-control study*—For matched studies, give matching criteria and the number of controls per case | 6-8 |
| Variables | 7 | Clearly define all outcomes, exposures, predictors, potential confounders, and effect modifiers. Give diagnostic criteria, if applicable | 7-8 |
| Data sources/ measurement | 8* | For each variable of interest, give sources of data and details of methods of assessment (measurement). Describe comparability of assessment methods if there is more than one group | |
| Bias | 9 | Describe any efforts to address potential sources of bias | 6-8 |
| Study size | 10 | Explain how the study size was arrived at | 6-8 |
| Quantitative variables | 11 | Explain how quantitative variables were handled in the analyses. If applicable, describe which groupings were chosen and why | 7-8 |
| Statistical methods | 12 | (*a*) Describe all statistical methods, including those used to control for confounding | 7-8 |
| | | (*b*) Describe any methods used to examine subgroups and interactions | 8 |
| | | (*c*) Explain how missing data were addressed | 8 |
| | | (*d*) *Cohort study*—If applicable, explain how loss to follow-up was addressed<br>*Case-control study*—If applicable, explain how matching of cases and controls was addressed<br>*Cross-sectional study*—If applicable, describe analytical methods taking account of sampling strategy | 6-8 |
| | | (*e*) Describe any sensitivity analyses | 8 |

Continued on next page

For peer review only

| Results | | | Page |
|---|---|---|---|
| Participants | 13* | (a) Report numbers of individuals at each stage of study—eg numbers potentially eligible, examined for eligibility, confirmed eligible, included in the study, completing follow-up, and analysed | 9-10 |
| | | (b) Give reasons for non-participation at each stage | 9-10 |
| | | (c) Consider use of a flow diagram | 10 |
| Descriptive data | 14* | (a) Give characteristics of study participants (eg demographic, clinical, social) and information on exposures and potential confounders | 9,12 |
| | | (b) Indicate number of participants with missing data for each variable of interest | NA |
| | | (c) *Cohort study*—Summarise follow-up time (eg, average and total amount) | 6, 13 |
| Outcome data | 15* | *Cohort study*—Report numbers of outcome events or summary measures over time | |
| | | *Case-control study*—Report numbers in each exposure category, or summary measures of exposure | *13* |
| | | *Cross-sectional study*—Report numbers of outcome events or summary measures | |
| Main results | 16 | (*a*) Give unadjusted estimates and, if applicable, confounder-adjusted estimates and their precision (eg, 95% confidence interval). Make clear which confounders were adjusted for and why they were included | 13, 15 |
| | | (*b*) Report category boundaries when continuous variables were categorized | NA |
| | | (*c*) If relevant, consider translating estimates of relative risk into absolute risk for a meaningful time period | NA |
| Other analyses | 17 | Report other analyses done—eg analyses of subgroups and interactions, and sensitivity analyses | 15 |
| **Discussion** | | | |
| Key results | 18 | Summarise key results with reference to study objectives | 17-18 |
| Limitations | 19 | Discuss limitations of the study, taking into account sources of potential bias or imprecision. Discuss both direction and magnitude of any potential bias | 19-20 |
| Interpretation | 20 | Give a cautious overall interpretation of results considering objectives, limitations, multiplicity of analyses, results from similar studies, and other relevant evidence | 18-19 |
| Generalisability | 21 | Discuss the generalisability (external validity) of the study results | 18 |
| **Other information** | | | |
| Funding | 22 | Give the source of funding and the role of the funders for the present study and, if applicable, for the original study on which the present article is based | 21 |

*Give information separately for cases and controls in case-control studies and, if applicable, for exposed and unexposed groups in cohort and cross-sectional studies.

**Note:** An Explanation and Elaboration article discusses each checklist item and gives methodological background and published examples of transparent reporting. The STROBE checklist is best used in conjunction with this article (freely available on the Web sites of PLoS Medicine at http://www.plosmedicine.org/, Annals of Internal Medicine at http://www.annals.org/, and Epidemiology at http://www.epidem.com/). Information on the STROBE Initiative is available at www.strobe-statement.org.

For peer review only

BMJ Open

# Should I stay or should I go? A retrospective propensity score matched analysis using administrative data of hospital-at-home for older people

| | |
|---|---|
| Journal: | *BMJ Open* |
| Manuscript ID | bmjopen-2018-023350.R1 |
| Article Type: | Research |
| Date Submitted by the Author: | 18-Dec-2018 |
| Complete List of Authors: | Tsiachristas, Apostolos; University of Oxford, Health Economics Research Centre, Nuffield Department of Population Health<br>Ellis, Graham; Monklands Hospital, NHS Lanarkshire<br>Buchanan, Scott; Information Services Division, National Services Scotland<br>Langhorne, Peter; University of Glasgow, Institute of Cardiovascular and Medical Sciences<br>Stott, David; Institute of Cardiovascular and Medical Sciences, University of Glasgow, Geriatric Medicine<br>Shepperd, S; University of Oxford, Nuffield Department of Population Health |
| <b>Primary Subject Heading</b>: | Geriatric medicine |
| Secondary Subject Heading: | Health economics, Health services research |
| Keywords: | hospital-at-home, admission avoidance, intermediate care, costs, mortality, UK |
| | |

SCHOLARONE™ Manuscripts

# Should I stay or should I go? A retrospective propensity score matched analysis using administrative data of hospital-at-home for older people

Apostolos Tsiachristas[1], Graham Ellis[2], Scott Buchanan[3], Peter Langhorne[4], David J Stott[4], Sasha Shepperd[1].

[1] Nuffield Department of Population Health, University of Oxford, Oxford, UK

[2] Monklands Hospital, NHS Lanarkshire, Glasgow, UK

[3] Information Services Division, National Services Scotland, Edinburgh, UK

[4] Institute of Cardiovascular and Medical Sciences, University of Glasgow, Glasgow, UK

Word Count: 4783

Correspondence to: Apostolos Tsiachristas, Health Economics Research Centre, Nuffield Department of Population Health, Richard Doll building, Old Road Campus, OX3 7LF, Oxford, UK; Tel: +44(0)1865 289470; email: apostolos.tsiachristas@dph.ox.ac.uk

For peer review only

# Abstract

Objectives: To compare the characteristics of populations admitted to hospital-at-home services with the population admitted to hospital and assess the association of these services with healthcare costs and mortality.

Design: In a retrospective observational cohort study of linked patient level data, we used propensity score matching in combination with regression analysis.

Participants: Patients aged 65 years and older admitted to hospital-at-home or hospital.

Interventions: Three geriatrician-led admission avoidance hospital-at-home services in Scotland.

Outcome measures: Healthcare costs and mortality.

Results: Patients in hospital-at-home were older and more socioeconomically disadvantaged, had higher rates of previous hospitalization, and there was a greater proportion of women and people with several chronic conditions compared with the population admitted to hospital. The cost of providing hospital-at-home varied between the three sites from £628 to £2928 per admission. Hospital-at-home was associated with 18% lower costs during the follow-up period in site one (ratio of means 0.82; 95%CI: 0.76-0.89). Limiting the analysis to costs during the 6 months following index discharge, patients in the hospital-at-home cohorts had 27% higher costs (ratio of means 1.27; 95%CI: 1.14-1.41) in site one, 9% (ratio of means 1.09; 95%CI: 0.95-1.24) in site two and 70% in site three (ratio of means 1.70; 95%CI: 1.40-2.07) compared with patients in the control cohorts. Admission to hospital-at-home was associated with an increased risk of death during the follow-up period in all three sites (1.09, 95%CI: 1.00-1.19 site one; 1.29, 95%CI: 1.15-1.44 site two; 1.27, 95%CI: 1.06-1.54 site three).

Conclusions: Our findings indicate that in these three cohorts, the populations admitted to hospital-at-home and hospital differ. We cannot rule out the risk of residual confounding, as our analysis relied on an administrative data set and we lacked data on disease severity and type of hospitalised care received in the control cohorts.


**Strengths and limitations of the study**

- The study used a large dataset from three of the largest Health Boards in Scotland.
- The retrospective cohort study has allowed inferences from real world evidence.
- Various sensitivity analyses helped to address uncertainty in the results.
- The major limitation of this type of non-randomised comparison is residual confounding.
- The lack of data on quality of life, as well as use of subsequent health, social, community and informal care is a limitation.



For peer review only - http://bmjopen.bmj.com/site/about/guidelines.xhtml

For peer review only



For peer review only - http://bmjopen.bmj.com/site/about/guidelines.xhtml

# Introduction

Organising health systems to optimise the health outcomes of older people and contain costs is a priority as populations around the world age, and the demand for healthcare continues to rise. Despite a global policy emphasis on 'care closer to home'[1] and initiatives that seek to ease demand for hospital based healthcare, efforts to innovate and deliver healthcare services that provide an alternative to hospital admission for older people have been piecemeal and often lack a health system perspective. A lack of evidence to support decision-making has contributed to this. Avoiding admission to hospital by providing acute healthcare in people's homes, often as a hospital outreach service, is one of the more popular service innovations and yet there is uncertainty around the effectiveness and cost-effectiveness of this form of care.[2]

Box 1 Description of each service

Hospital-at-home

The three hospital-at-home services are broadly similar, capacity ranged between 24 to 60 beds for the period of the analysis. Each is a geriatrician-led service that is supported by nurses (sometimes nurse practitioners) and therapy practitioners for the initial assessment; geriatricians and the multi-disciplinary team review patients in their homes and meet daily (a virtual ward round) to discuss patient cases and agree actions. Rehabilitation is available within the existing team with onward referral to community rehabilitation as required, and in one site rehabilitation is accessed through a parallel community rehabilitation services. Out of hours emergency cover is provided by primary care out-of-hours. Patients are referred to the service from GPs, sometimes through a central referral number or via step down from the acute hospital. The service offers access to diagnostics such as radiology, and intravenous fluids, antibiotics and oxygen. Cases are discussed daily with the multidisciplinary team at the virtual ward round and daily management plans agreed. In one site there is close working with the day hospital where patients can be referred for follow up or for investigations. Patients access investigations and treatment with the same speed as inpatients. The services support intravenous therapies in the home.

Hospital

The provision of hospital based acute health services varied among the sites; in one site there were three district general hospitals (1,653 beds) that provide acute health services to a mainly urban population of 652,230, with a total of 1,653 beds; in site two a hospital (550 beds) provides acute healthcare to a population of 180,130; and in site three there are two district general hospitals (825 beds) that provide healthcare to a population of 358,900, and acute admissions are via one of the hospitals.

The use of administrative data to evaluate service delivery interventions has the potential to provide a simple and efficient mechanism to provide real-world evidence about policy relevant service innovations, and embed evaluation into local decision-making. However, previous experience of using routine data in this area of research has been of mixed success due to a limited set of variables, missing data and the complexity of policy relevant questions that often require a broad and longer term perspective.[3] Administrative healthcare data collected in Scotland is unique in that it is population based, with little missing data. The aim of this study was to use these data to compare the characteristics of populations from three Health Boards who used a geriatrician-led hospital-at-home service with the population who received hospital care, and to assess the impact of these services on healthcare costs and mortality.

For peer review only

# Methods

## *Setting*

We used patient level data collected by three of the fourteen Scottish Health Boards of all patients aged 64 years and older, and who were admitted (referred to as the index admission) to either geriatrician-led admission avoidance hospital-at-home or inpatient hospital between August 2014 and December 2015 (17 months) in site one and site two, and between January 2015 and December 2016 (24 months) in site three. These services are commissioned by integrated health and social care boards that cover a population of almost 1.5 million in urban and rural areas. The Information Service Division (ISD), part of NHS Scotland, de-identified, cleaned and linked individual patient records to derive activity and costs related to periods before and after the index admissions. We obtained signed release forms from each Board's Caldicott guardian, and followed the ISD data sharing agreement.

## *Intervention*

The three service models of hospital-at-home provided an admission avoidance function that provided an alternative to inpatient hospital care, and had similar structures and functions; the main differences were in the capacity of the services and the organisation of services for rehabilitation. (Box 1)

## *Data sources*

Data were available for each person for two years prior to their index admission, and from the point of their index admission to six months after index discharge from hospital-at-home or hospital. Box 2 presents a full list of all variables included in the dataset. Figure 1 provides schematic examples of the differing calendar time periods studied before and after index admission for people admitted between August 2014 and December 2015 to hospital-at-home (Patients A and B) or hospital (Patients C and D) in site one. As this illustrates, the maximum follow-up period for each patient consisted of the period between index admission and index discharge and 6 months after index discharge. The data were collected via the data systems used in hospitals to collect patient data. Hospital-at-home activity data was submitted to ISD from the local systems of the three sites. The linked data set included acute inpatient, geriatric long stay and day case, mental health admissions, outpatient appointments accident and emergency attendances, community prescribing and death registrations.



For peer review only - http://bmjopen.bmj.com/site/about/guidelines.xhtml

Figure 1. Illustration of obtained data from site one

Box 2. List of variables included in the dataset
Costs of accidents and emergency attendances,
Costs of acute day cases,
Costs of acute elective hospitalisation,
Costs of acute non-elective hospitalisation,
Costs of geriatric wards,
Costs of mental health wards,
Costs of outpatient visits,
Costs of prescribed medication,
Costs of (re)admission to hospital-at-home.
Primary ICD-10 codes on index discharge,
Secondary ICD-10 codes on index discharge,
Length of stay of the index admission,
Age on index admission,
Gender,
Scottish Index of Multiple Deprivation (SIMD), 1 (most deprived) to 10 (most affluent)
Long-term conditions,
Date of death (if applicable),
Based on ICD-10 codes:
Cardiovascular disease (CVD) (I60-I69, G45)
Chronic obstructive pulmonary disorder (COPD) (J41-J44, J47),
Dementia (F00-F03, F05.1),
Diabetes (E10-E14),
Coronary heart disease (CHD, ICD10: I20-I25),
Heart failure (I500, I501, I509),
Renal failure (N03, N18, N19, I12, I13),
Epilepsy (G40, G41),
Asthma (J45, J46),
Atrial fibrillation (I48, MS, G35),
Cancer (C00-C97),
Arthritis (M05, M19, M45, M47, M460-M462, M464, M468, M469),
Parkinson's (G20-G22),
Chronic liver disease (K711, K713, K714, K717, K754),
Congenital problems (Q00-Q99),
Diseases of blood and blood forming organs (D50-D89),
Other diseases of the digestive system (K00-K122, K130-K839, K85X, K860-K93),
Other endocrine metabolic diseases (E00-E07, E15-E35, E70-E90)
Admitted to HAH or hospital.

*Selection of patients in the hospital-at-home and control cohorts*

We included patients aged 65 years and older, and who were classified as an unscheduled admission to general or geriatric medicine. In the control cohort, we excluded those with a diagnosis that would not be eligible for management through hospital-at-home; these exclusions included acute intracerebral crisis (intracerebral infections, trauma or haemorrhage), stroke and related codes, acute coronary syndromes and myocardial infarction, surgical emergencies including vascular, urological, gynaecological and general surgical presentations, orthopaedic diagnosis of fractures and trauma, cardiothoracic diagnoses, poisoning and complications of surgery. We also excluded from the control group those who had a diagnosis (i.e. primary and secondary ICD-10 code) that was not observed in any of the hospital-at-home admissions in each site (1081 patients in site one, 1405 in site two and in 451 in site three) (Figure 2). Each patient was counted as a single episode of healthcare.



For peer review only - http://bmjopen.bmj.com/site/about/guidelines.xhtml

*Intervention costs*

We collected data on the costs of hospital-at-home using a template derived from the Cost-It tool of the World Health Organisation.[4] The cost categories included staff, training, transport, information and communication, clinical materials/equipment, support services, laboratory services, diagnostics, overheads and other costs. Clinician managers supported by finance staff in the three Health Boards completed this template based on the actual spending for the hospital-at-home service for the time periods covered by the ISD data. The cost per hospital-at-home admission was calculated by dividing the total costs of the hospital-at-home service by the total number of hospital-at-home admissions during the same period.

*Statistical analysis*

We used an iterative approach to the analysis, starting with a description of the two cohorts (i.e. those admitted to hospital-at-home and those admitted to hospital) for each Health Board. We calculated means, standard errors, and frequencies to describe differences in patient characteristics at index admission and tested differences using Mann-Whitney test for continues variables and Chi-square test for categorical variables. We also estimated the mean differences in resource utilisation costs (with bootstrapped standard errors) and the unadjusted relative risk of mortality between the two cohorts for each Health Board.

Further, we investigated the association of being admitted to hospital-at-home or hospital with mortality and cost over a minimum follow-up period of six months. To do this, we followed the Medical Research Council guidelines on performing natural experiments and scientific literature to adopt a step-wise strategy to select the propensity score matching (PSM) technique that most reduced observed confounding between the two cohorts in each Health Board.[5-8] First, we included all possible confounding variables available in the dataset (see Box 2 and Figure 2), and considered that the inclusion of covariates not associated with the treatment assignment would have little influence in the propensity score model.[5] Second, we matched the two cohorts in each site using a range of the most commonly used PSM techniques; these included Mahalanobis, 1-to-1, K-to-1, kernel, local linear regression, spline, and inverse probability weighting techniques. Second, the performance of each PSM technique on covariate balancing was assessed based on the mean and median percentage standardised bias as well as Rubin's B (the absolute standardized difference of the means of the linear index of the propensity score in the treated and (matched) non-treated group) and Rubin's R (the ratio of treated to (matched) non-treated variances of the propensity score index). Following Rubin's (2001) recommendation, we considered B less than 25 and R between 0.5 and 2 to indicate sufficient balance.[9] Third, we chose the PSM technique that had the lowest values on these performance indicators in each of the three Health Boards. We matched the two cohorts in each Health Board by



For peer review only - http://bmjopen.bmj.com/site/about/guidelines.xhtml

socio-demographic characteristics (i.e. age, gender, socio-economic status), diagnosis code (i.e. primary and secondary ICD-10 code) of index admission, morbidity (i.e. type of long-term condition, mortality during follow-up (for the analysis of cost), 2-year costs prior to the index admission (by cost category as listed in Box 1), and date of index admission (to account for seasonal trends).

We performed a doubly robust estimation to further reduce confounding by using a regression analysis after performing the most suitable PSM technique and including the confounding variables listed above as covariates.[10] In the regression, we used generalised linear regression models (GLMs) with gamma distribution and log link to investigate the association of hospital-at-home with total costs during the follow-up period, and total costs in 6 months following index discharge. We also used GLMs with Poisson distribution and log link to estimate the relative risk of mortality. Robust standard errors were specified in all regression models. We calculated Kaplan-Meier survival curves, with and without using the weights from the PSM, and used log-rank tests to test the equality of the survival functions. There were few missing observations in the dataset and thus, complete case analysis was performed.

*Subgroup analysis*

We conducted a sub-group analysis, running the same regression models used in the main analysis, to investigate the association of hospital-at-home services with costs and mortality for the population who had a diagnosis of dementia. We considered this population to be important due to their complex healthcare needs, and the increasing prevalence of dementia.[11 12] In a second subgroup analysis, we excluded patients who died during the follow-up period and investigated the association of hospital-at-home with costs. In both subgroup analyses, propensity score matching was performed to match sub-cohorts in each site.

*Sensitivity analysis*

In a univariate sensitivity analysis, we reduced and increased the intervention cost of admission avoidance hospital-at-home by 50%, as there are no standard unit costs to benchmark these types of services and we were concerned that costs for these services may vary due to economies of scale, size, experience, setting, human resource capacity, and error. This sensitivity analysis was expected to impact the costs during index admission and the costs of admission to hospital-at-home in the six months after discharge.

*Patient involvement*

Patients were not involved in this retrospective analysis of administrative data.



For peer review only - http://bmjopen.bmj.com/site/about/guidelines.xhtml

# Results

*Characteristics of the population cohorts*

After applying the exclusion criteria, 1737 patients were admitted to hospital-at-home in site one between August 2014 and December 2015 (17 months), 1463 patients were admitted to hospital-at-home in site two between January 2015 and December 2016 (24 months), and 433 patients were admitted to hospital-at-home in site three between August 2014 and December 2015 (17 months) (Figure 2). In the same period, there were 13139 patients admitted to 3 hospitals in site one, 3994 patients admitted to 1 hospital in site two, and 1844 patients admitted to 1 hospital in site three.

There were few differences between the hospital-at-home cohorts in the three sites, the main difference being that a larger proportion of the population in site two lived in a more affluent area (i.e. scored five or higher on the Scottish Index of Multiple Deprivation) . Patients admitted to hospital-at-home were on average three to four years older than those admitted to hospital, were more likely to be female (range from 5 percentage points to 9 percentage points), and a higher proportion had more than four long-term conditions (approximately 7 percentage points) compared with patients admitted to hospital (Table 1). The largest difference between those admitted to hospital-at-home and to hospital in site one and site two was in the proportion of patients with dementia (10 percentage points higher in the hospital-at-home cohorts), while in site three it was the proportion of patients with renal failure (also 10 percentage points higher in the hospital-at-home cohort).

We compared the two cohorts in each site, from index admission to six months post discharge from hospital-at-home or hospital (Table 2). There was on average a higher percentage of deaths while receiving healthcare in hospital compared with those receiving healthcare in hospital-at-home (6% vs., 1% site one; 6% vs., 3% site two; 4% vs., 1% site three); and a higher percentage of deaths in the follow-up period, from admission to six months after discharge, in the groups that had received hospital-at-home (21% vs., 28% site one; 22% vs., 32% site two; 17% vs., 27% site three). Patients in the hospital-at-home cohort lived on average eight (site one), ten (site two), and twelve (site three) fewer days during the whole follow-up, and their index admission was on average fewer days in site one (mean unadjusted difference -2.64, 95%CI -2.97 to -2.31) and site three (mean unadjusted difference -2.02, 95%CI -2.66 to -1.37) and longer in site two (mean unadjusted difference 1.25, 95% CI 0.86 to 1.64).

The cost during a hospital-at-home admission was on average lower than hospital admission in site one (mean difference -£2318; 95%CI: £-2420 to £-2217) and site three (mean difference -£1096; 95%CI: £-1398 to £-793), and slightly lower (mean difference £-153; 95%CI: £-277; to £-29) in site two (Table 2). In the hospital-at-home cohort, these costs included the intervention costs of delivering the



For peer review only - http://bmjopen.bmj.com/site/about/guidelines.xhtml

service at home, which were £628 per admission and £113 per day in site one, £2928 per admission and £398 per day in site two, and £864.54 per admission and £117.57 per day in site three. In each Health Board, staff were the major driver of the cost of delivering hospital-at-home (site one 95%, site two 87%, site three 94%). Detailed information on the costs of delivering hospital at home are in Appendix 1.

Each of the three hospital-at-home cohorts incurred higher healthcare costs, driven by non-elective hospitalisation, prior to their index admission compared with the respective control cohort. Site one had on average 40% higher costs (mean difference £3219; 95%CI: £2513 to £3925), site two 56% higher costs (mean difference £5064; 95%CI: £3984 to £6143) and site three 57% higher costs (mean difference £4115; 95%CI: £2467 to £5764). In the six months following discharge from the index admission, costs were higher for each of the three hospital-at-home cohorts; in site one costs were on average 43% higher (mean difference £1839; 95%CI: £1423 to £2255), in site two they were 16% higher (mean difference £875, 95%CI: £156 to £1595), and in site three they were 92% higher (mean difference £3068, 95%CI: £2178 to £3958). The larger increase in costs in all sites was due to higher non-elective hospitalisation costs in the group who had received hospital-at-home care (mean difference £1517, 95%CI £1134 to 1899 site one; mean difference £529, 95%CI £-77 to 1135 site two; mean difference £2618, 95%CI £1779 to 3458 site three) during the six months follow-up.

When the cost of the index admission was included in the analysis, the cost during follow-up (i.e. including the index admission and 6-months healthcare resource use after index discharge) was 6% lower (mean difference -£480, 95%CI: £-996 to £36) in the hospital-at-home cohort, compared with the control cohort in site one; while these costs were 8% higher in site two (mean difference £722, 95%CI: £32 to £1413) and 35% higher in site three (mean difference £1973, 95%CI: £1019 to £2927).

Compared with the control cohort, the mean costs per day of being alive during the follow-up period were 13% (mean difference £-12; 95%CI: -17 to -6) lower in the hospital-at-home cohort in site one, while these costs were 34% higher (mean difference £37; 95%CI: 18 to 56) and 66% higher (mean difference £36; 95%CI: 18 to 53) in site two and site three respectively.

Figure 2 Flowchart of study population



For peer review only - http://bmjopen.bmj.com/site/about/guidelines.xhtml

Table 1 Patient characteristics at index admission

| | Site one | | Site two | | Site three | |
|---|---|---|---|---|---|---|
| *Variable* | *Control (n=13139)* | *HAH (n=1737)* | *Control (n=3994)* | *HAH (n=1463)* | *Control (n=1844)* | *HAH (n=433)* |
| Mean age on admission (se) | 77.8 (0.07) | 81.2 (0.17)** | 78.5 (0.13) | 82.2 (0.21)** | 77.3 (0.18) | 81.4 (0.34)** |
| Female | 7,468 (57%) | 1,096 (63%)** | 2,102 (53%) | 909 (62%)** | 1037 (56%) | 266 (61%)* |
| Higher than 4 on the SIMD | 5,005 (38%) | 609 (35%)** | 1,960 (49%) | 775 (53%)* | 837 (45%) | 192 (44%) |
| More than 4 chronic conditions | 4,974 (38%) | 777 (45%)** | 1,664 (42%) | 725 (50%)** | 659 (36%) | 185 (43%)** |
| Arthritis | 3,431 (26%) | 497 (29%)* | 1,455 (37%) | 572 (39%) | 606 (33%) | 155 (36%) |
| Asthma | 1,370 (10%) | 183 (11%) | 497 (13%) | 207 (14%) | 177 (10%) | 49 (11%) |
| Atrial fibrillation | 3,659 (28%) | 488 (28%) | 1,555 (29%) | 468 (32%)* | 498 (27%) | 126 (29%) |
| Cancer | 3,749 (29%) | 485 (28%) | 1,261 (32%) | 371 (25%)** | 580 (31%) | 124 (29%) |
| CVD | 2,922 (22%) | 467 (27%)** | 763 (19%) | 392 (27%)** | 373 (20%) | 114 (26%)** |
| Liver disease | 499 (4%) | 50 (3%) | 183 (5%) | 52 (4%) | 72 (4%) | 20 (5%) |
| COPD | 3,641 (28%) | 505 (29%) | 1,083 (27%) | 428 (29%) | 510 (28%) | 132 (31%) |
| Dementia | 1,999 (15%) | 439 (25%)** | 665 (17%) | 390 (27%)** | 223 (12%) | 74 (17%)** |
| Diabetes | 2,985 (23%) | 403 (23%) | 948 (24%) | 350 (24%) | 410 (22%) | 115 (27%)* |
| Epilepsy | 459 (4%) | 75 (4%) | 146 (4%) | 78 (5%)** | 53 (3%) | 10 (2%) |
| CHD | 5,034 (38%) | 733 (42%)** | 1,425 (36%) | 575 (39%)* | 624 (34%) | 141 (33%) |
| Heart failure | 2,197 (17%) | 404 (23%)** | 744 (19%) | 32 (23%)** | 328 (18%) | 109 (25%)** |
| MS | 73 (1%) | 6 (0%) | 21 (1%) | 17 (1%)* | 14 (1%) | 2 (1%) |
| Parkinson's | 293 (2%) | 66 (4%)** | 82 (2%) | 53 (4%)** | 53 (3%) | 20 (5%) |
| Renal failure | 2,501 (19%) | 394 (23%)** | 780 (20%) | 339 (23%)** | 284 (15%) | 110 (25%)** |
| Congenital problems | 277 (2%) | 38 (2%) | 159 (4%) | 51 (4%) | 51 (3%) | 9 (2%) |
| Diseases of blood | 3,784 (29%) | 553 (32%)** | 1,143 (29%) | 426 (29%) | 485 (26%) | 125 (29%) |
| Endocrine metabolic disease | 4,505 (34%) | 624 (36%) | 1,737 (44%) | 652 (45%) | 642 (35%) | 151 (35%) |
| Disease of digestive system | 9,341 (71%) | 1,249 (72%) | 2,710 (68%) | 1,006 (69%) | 1145 (62%) | 286 (66%) |

* p<0.05 ** p<0.01 in chi-square test for categorical and Mann-Whitney for continuous variables to test differences between HAH and control; HAH: hospital-at-home; SIMD ranges from 1 (most deprived) to 10 (most affluent); Note: a patient could be registered with more than one ICD-10 codes;

For peer review only

Table 2. Mortality, resource utilisation and costs

| | Site one | | | Site two | | | Site three | | |
|---|---|---|---|---|---|---|---|---|---|
| *Variable* | *Control (n=13139)* | *HAH (n=1737)* | *Mean difference or risk ratio (95%CI)* | *Control (n=3994)* | *HAH (n=1463)* | *Mean difference or risk ratio (95%CI)* | *Control (n=1844)* | *HAH (n=433)* | *Mean difference or risk ratio (95%CI)* |
| Died during index admission | 844 (6%) | 20 (1%) | 0.18 (0.12;0.28)## | 256 (6%) | 47 (3%) | 0.50 (0.37;0.68)## | 78 (4%) | 2 (1%) | 0.11 (0.03;0.44)## |
| Died during follow-up including index admission | 2,787 (21%) | 483 (28%) | 1.31 (1.21;1.42)## | 867 (22%) | 471 (32%) | 1.48 (1.35;1.63)## | 319 (17%) | 116 (27%) | 1.55 (1.29;1.86)## |
| Means days alive during follow-up (se) | 159 (0.50) | 151 (1.45) | -8.32 (-11.32;-5.32) | 156 (0.91) | 146 (1.72) | -10.10 (-14;-7) | 163 (1.22) | 151 (2.88) | -12 (-18;-6) |
| Mean length of index admission in days (se) | 8.18 (0.12) | 5.54 (0.13) | -2.64 (-2.97;-2.31) | 6.10 (0.14) | 7.35 (0.14) | 1.25 (0.86;1.64) | 6.36 (0.26) | 4.34 (0.20) | -2.02 (-2.66;-1.37) |
| Mean 2 year historical costs (se) | | | | | | | | | |
| A&E | 173 (2) | 253 (7) | 80 (65;94) | 136 (4) | 180 (6) | 44 (28;60) | 143 (5) | 202 (12) | 59 (31;87) |
| Elective hospital care | 985 (37) | 956 (134) | -28 (-352;295) | 1,027 (64) | 705 (86) | -321 (-519;-123) | 981 (87) | 1036 (372) | 55 (-723;833) |
| Non-elective hospital care | 4,037 (79) | 6,945 (266) | 2908 (2452;3364) | 5,101 (185) | 9,593 (394) | 4492 (3804;5179) | 3978 (211) | 7832 (614) | 3854 (2591;5118) |
| Hospital day case | 707 (25) | 439 (32) | -269 (-340;-197) | 625 (66) | 290 (44) | -336 (-479;-193) | 544 (49) | 358 (55) | -186 (-334;-38) |
| Geriatric long stay | 360 (27) | 504 (82) | 143 (-66;354) | 117 (29) | 252 (72) | 135 (-13;283) | 105 (31) | 229 (59) | 125 (14;235) |
| Mental ward | 247 (32) | 367 (117) | 119 (-177;411) | 347 (79) | 1,053 (205) | 706 (265;1147) | 220 (75) | 252 (139) | 32 (-329;393) |
| Outpatient | 173 (2) | 173 (5) | 0 (-11;11) | 222 (4) | 206 (6) | -15 (-30;0) | 212 (6) | 201 (12) | -11 (-38;15) |
| Medication (GP prescriptions) | 1,468 (15) | 1,733 (43) | 256 (187;341) | 1,524 (28) | 1,883 (52) | 360 (253;466) | 1034 (39) | 1221 (78) | 188 (30;346) |
| Total | 8,149 (109) | 11,369 (359) | 3219 (2513;3925) | 9,098 (239) | 14,162 (477) | 5064 (3984;6143) | 7217 (267) | 11333 (772) | 4115 (2467;5764) |
| Mean costs during index admission (se) | 3,195 (41) | 877# (32) | -2318 (-2420;-2217) | 3,426 (71) | 3,273# (32) | -153 (-277;-29) | 2383 (90) | 1287 (132) | -1096 (-1398;-793) |
| Mean costs 6 months after index discharge (se) | | | | | | | | | |
| A&E | 72 (1) | 88 (3) | 17 (11;22) | 55 (2) | 53 (3) | -2 (-9;4) | 59 (2) | 71 (5) | 12 (-1;25) |
| Elective hospital care | 305 (20) | 157 (40) | -148 (-236;-60) | 272 (28) | 204 (50) | -68 (-190;53) | 169 (33) | 313 (117) | 144 (-92;380) |
| Non-elective hospital care | 2,444 (51) | 3,961 (171) | 1517 (1134;1899) | 3,942 (130) | 4,471 (251) | 529 (-77;1135) | 2029 (123) | 4648 (421) | 2618 (1779;3458) |
| Hospital day case | 237 (11) | 73 (11) | -164 (-191;-138) | 234 (24) | 96 (21) | -139 (-198;-79) | 168 (23) | 63 (15) | -105 (-162;-48) |
| Geriatric long stay | 643 (45) | 1,014 (131) | 371 (79;663) | 218 (34) | 150 (46) | -68 (-178;41) | 320 (56) | 700 (186) | 381 (-73;834) |
| Mental ward | 165 (22) | 206 (51) | 41 (-58;140) | 299 (56) | 259 (77) | -40 (-224;143) | 211 (65) | 120 (62) | -91 (-245;64) |
| Outpatient | 54 (1) | 45 (2) | -9 (-13;-5) | 61 (2) | 54 (3) | -8 (-14;-2) | 65 (3) | 67 (6) | 2 (-12;16) |
| Medication (GP prescriptions) | 392 (5) | 415 (13) | 23 (-5;52) | 402 (9) | 482 (16) | 80 (45;115) | 314 (12) | 338 (27) | 24 (-28;76) |
| Hospital-at-home | 4 (1) | 196 (11) | 193 (170;216) | 50 (7) | 642 (45) | 592 (506;679) | 7 (1) | 90 (12) | 83 (59;108) |
| Total | 4,316 (78) | 6,155 (240) | 1839 (1423;2255) | 5,535 (154) | 6,410 (286) | 875 (156;1595) | 3342 (163) | 6410 (510) | 3068 (2178;3958) |
| Mean costs in follow-up (se) including index admission | 7,513 (92) | 7,031 (243) | -480 (-996;36) | 8,961 (180) | 9,683 (290) | 722 (32;1413) | 5724 (199) | 7697 (521) | 1973 (1019;2927) |
| Mean costs per lived day in follow-up (se) | 83 (1) | 72 (3) | -12 (-17;-6) | 109 (3) | 146 (8) | 37 (18;56) | 55 (2) | 91 (8) | 36 (18;53) |

# it includes the interventions costs (i.e. £628 in site one, £2,928 in site two, and £865.54 in site three) and other costs occurred during the episode; ## Unadjusted Risk Ratio;

For peer review only

*Selection of propensity score matching technique*

In the propensity score matched analysis, there were 1696, 925, and 427 patients in the hospital-at-home cohort and 11571, 3849, and 1683 patients in the hospital cohort in site one, site two, and site three respectively (Figure 2). Local linear regression matching was the best PSM technique to match the cohorts in site one and site three for costs and mortality, as it resulted in a lower mean (i.e. 1.5 and 1.8 respectively) and median (i.e. 1.2 and 1.6 respectively) percentage standardised bias, as well as the lowest Rubin's B (i.e. 9.4 and 9.6 respectively). Based on the same criteria, Kernell matching was selected to match the cohorts in site two. Rubin's R was within the suggested range (i.e. from 0.5 to 2) in the selected techniques. These results as well as the patient characteristics at index admission after propensity score matching are presented in Appendix 2. As this Appendix shows, the differences in patient characteristics between the compared cohorts were almost eliminated after propensity score matching.

*Main propensity score matched analysis*

The results of the main analysis are presented in Panel A in Table 3. After propensity score matching and regression analysis, the healthcare cost for site one in hospital-at-home during the whole follow-up period (i.e. during index admission and over six months after discharge from the index admission) was on average 18% lower (ratio of means: 0.82; 95%CI: 0.76 to 0.89) than admission to hospital. When the cost of the index admission was excluded from the hospital-at-home and hospital cohorts, costs were on average 27% higher (ratio of means: 1.27; 95%CI: 1.14 to 1.41) for hospital-at-home compared with hospital in site one. In site two, the difference in costs between the hospital-at-home and hospital was close to zero (ratio of means: 1.00; 95%CI 0.92 to 1.09) during the whole follow-up period and 9% higher (ratio of means: 1.09; 95%CI: 0.95 to1.24) when the cost of the index admission was excluded. In site three, patients admitted to hospital-at-home had on average 15% higher cost during the whole follow-up period (ratio of means: 1.15; 95%CI 0.99 to 1.33) and 70% higher cost when the cost of the index admission was excluded (ratio of means: 1.70; 95%CI 1.40 to 2.07) compared with patients admitted to hospital. The full results of the regression analyses are presented in Appendix 3.

There may be an increased risk of mortality in all three hospital-at-home cohorts (site one: relative risk 1.09; 95%CI 1.00 to 1.19) (site two: relative risk 1.29; 95%CI: 1.15 to 1.44) (site three: relative risk 1.27; 95%CI: 1.06 to 1.54) compared with the hospital cohort after PSM and regression were performed to adjust for confounding. The Kaplan-Meier survival curves presented in Figure 3 show higher survival rates in the inpatient control cohorts in all three sites, and after weighting with the propensity score the control cohort in site two continued to have a higher survival rate than the hospital-at-home cohort. The difference in survival in site three between the results reported in Table


For peer review only - http://bmjopen.bmj.com/site/about/guidelines.xhtml

3 and the survival curve after weighting is explained by the fact that Kaplan-Meier curves are only weighted with the propensity score without performing an additional regression analysis.

Table 3. Results of the propensity score matched regression analyses

| Outcome variable | Site one (n=13267) | Site two (n=4769) | Site three (n=2110) |
|---|---|---|---|
| **Panel A: main analysis** | | | |
| Total costs during follow-up period[#] | 0.82 (0.03) [0.76;0.89] <0.001 | 1.00 (0.05) [0.92;1.09] 0.982 | 1.15 (0.09) [0.99;1.33] 0.073 |
| Total costs in 6 months after discharge | 1.27 (0.07) [1.14;1.41] <0.001 | 1.09 (0.07) [0.95;1.24] 0.219 | 1.70 (0.17) [1.40;2.07] <0.001 |
| Mortality rate during follow-up | 1.09 (0.05) [1.00;1.19] 0.059 | 1.29 (0.07) [1.15;1.44] <0.0010 | 1.27 (0.12) [1.06;1.54] 0.011 |
| **Panel B: subgroup analysis including only patients with dementia** | | | |
| *Outcome variable* | *Site one (n=2321)* | *Site two (n=1053)* | *Site three (n=280)* |
| Total costs during follow-up period[#] | 0.76 (0.05) [0.66;0.87] <0.001 | 0.76 (0.06) [0.66;0.88] <0.001 | 0.87 (0.15) [0.63;1.21] 0.409 |
| Total costs in 6 months after discharge | 1.18 (0.11) [0.99;1.41] 0.071 | 0.75 (0.09) [0.59;0.96] 0.021 | 1.58 (0.41) [0.95;2.63] 0.078 |
| Mortality rate during follow-up | 1.05 (0.09) [0.89;1.24] 0.594 | 1.41 (0.12) [1.19;1.67] <0.001 | 1.65 (0.32) [1.12;2.41] 0.011 |
| **Panel C: subgroup analysis including only survivors** | | | |
| *Outcome variable* | *Site one (n=10132)* | *Site two (n=3584)* | *Site three (n=1691)* |
| Total costs during follow-up period[#] | 0.85 (0.04) [0.77;0.94] 0.002 | 1.11 (0.03) [1.00;1.25] 0.058 | 1.20 (0.11) [1.00;1.43] 0.046 |
| Total costs in 6 months after discharge | 1.23 (0.08) [1.08;1.40] 0.002 | 1.17 (0.10) [0.99;1.38] 0.070 | 1.71 (0.20) [1.36;2.15] <0.001 |
| ***Panel D: sensitivity analysis*** | | | |
| *Outcome variable* | *Site one (n=13267)* | *Site two (n=4769)* | *Site three (n=2110)* |
| Total costs during follow-up period[#] (assuming 50% lower intervention costs) | 0.77 (0.03) [0.71;0.84] <0.001 | 0.81 (0.04) [0.74;0.9] 0.001 | 1.07 (0.09) [0.91;1.25] 0.399 |
| Total costs during follow-up period[#] (assuming 50% higher intervention costs) | 0.87 (0.03) [0.81;0.94] 0.001 | 1.18 (0.05) [1.09;1.28] <0.001 | 1.23 (0.09) [1.07;1.42] 0.004 |

# It includes the index admission period and 6 months post-discharge; Note: The results are presents as coefficient (se) [95%CI] p value; The results are after matching and adjusting for age, gender, socio-economic status, primary and secondary ICD-10 codes of index admission, type of long-term condition, mortality (for the analysis of costs), 2-year costs prior to the index admission (by cost category as listed in Box 1).

Figure 3. Survival curves before and after propensity score matching

### *Results of the subgroup analysis*

Patients with dementia (Panel B in Table 3) admitted to hospital-at-home services in site one and site two had an average of 24% lower costs (site one: ratio of means 0.76; 95%CI 0.66 to 0.87; site two: ratio of means 0.76 95%CI: 0.66 to 0.88) from the index admission to six months post-discharge. We found that the population who were admitted to hospital-at-home, and had a diagnosis of dementia, may have an increased risk of death (site one: 1.05, 95% CI 0.89 to 1.24; site two: relative risk 1.41, 95%CI 1.19 to 1.67; site three: relative risk 1.65, 95%CI 1.12 to 2.41) compared with those who had a diagnosis of dementia and who were admitted to hospital.

When we excluded people who died during follow-up (i.e. during index admission and 6 months after discharge), patients admitted to hospital-at-home in site one had lower costs (ratio of means 0.85, 95%CI: 0.77 to 0.94), while there was 11% increase in costs in site two (ratio of means 1.11, 95%CI: 1.00 to 1.25) and 20% increase in site three (ratio of means 1.20, 95%CI: 1.00 to 1.43); the mean costs were higher in the hospital-at-home cohort when the costs during the index admission were excluded (site one: ratio of means 1.23, 95%CI: 1.08 to 1.40; site two: ratio of means 1.17, 95% CI 0.99 to 1.38; site three: ratio of means 1.71, 95% CI 1.36 to 2.15) compared with patients admitted to hospital (Panel C in Table 3).


For peer review only - http://bmjopen.bmj.com/site/about/guidelines.xhtml

*Results of the sensitivity analyses*

The results from the sensitivity analysis (Panel D in Table 3) showed that patients in the hospital-at-home cohort in site one had 13% lower costs (ratio of means 0.87; 95%CI: 0.81 to 0.94) during the follow-up period (i.e. during index admission and 6 months after index discharge) when the hospital-at-home service costs were assumed to be 50% higher than in the main analysis. In site two, the results from the sensitivity analysis showed that the uncertainty in hospital-at-home service costs lead to increased costs or cost savings by about 18% (ratio of means 1.18; 95%CI: 1.09 to 1.28) during the whole follow-up period. In site three, the sensitivity analysis showed a 23% cost increase (ratio of means 1.23; 95%CI: 1.07 to 1.42), if the intervention costs of hospital-at-home were 50% higher.

## Discussion

*Main findings*

Patients who received healthcare from the hospital-at-home services were older, were more socioeconomically disadvantaged, had higher morbidity (measured by the number of long term conditions), higher rates of previous hospitalisation, and there was a greater proportion of women compared with the group admitted to hospital. The two groups also differed in terms of their clinical diagnosis, with the most marked difference across the three services being a greater percentage (five to ten percent difference) of people with dementia. The higher healthcare costs over the two years prior to index admission in those admitted to hospital-at-home were mainly driven by the costs of non-elective hospitalisation. However, the differences in patient characteristics were almost eliminated after propensity score matching. The cost of providing hospital-at-home varied between the three sites from £628 to £2928 per admission, and costs were driven primarily by staff costs. Our findings indicate that hospital-at-home might be associated with an increase in healthcare costs in the six months after index discharge. However, this increase in costs might be offset by likely cost-savings during the index admission. The higher healthcare cost at six months after index discharge, was driven primarily by acute non-elective hospitalisation. Interpreting this is not straightforward; it might indicate a lack of resources during the index admission to hospital-at-home, or an increased risk of hospital admission in the population who receive their healthcare through hospital-at-home. The suggestion of an increased risk of mortality at six months after the index admission might be genuine, or could indicate that propensity score matching did not control for all differences between the groups and thus, the estimates are subject to residual confounding.[13 14]


For peer review only - http://bmjopen.bmj.com/site/about/guidelines.xhtml

*Comparison with previous studies*

A meta-analysis of six small randomised controlled trials concluded that admission avoidance hospital-at-home probably makes little or no difference to the risk of death or transfer to hospital at six months' follow-up, and might increase the likelihood of living at home (albeit with low-certainty evidence); and highlighted the lack of evidence on cost.[2] Studies that have used 'real life data' offer the potential to address criticisms of limited external validity from randomised trials; and propensity score matching is one technique that has been used to balance co-variates when analysing routinely collected health data to assess these type of service delivery interventions. Findings have been consistent, and previous studies have reported higher rates of mortality and unplanned admission for those who received an intermediate care intervention, compared with matched controls.[6 14 15] However, it is possible that these findings are subject to residual confounding.

*Potential mechanisms and interpretation*

Healthcare services that cross the interface of primary and secondary care can bridge and strengthen the integration of acute and community services, and social care. However, by definition this can lead to a complex arrangement of services that reflect availability of local resources,[16] and a willingness to innovate. The hospital-at-home services evaluated in this analysis were established to reduce the demand for acute hospital beds by providing an alternative to admission to hospital, and to lower the risk of functional decline from the limited mobility that older people might experience when in hospital. However, it is possible that the services have several functions, for example by providing both rapid response and reablement, and this is reflected in the diverse population included in this analysis. Existing services and the overall structure of the healthcare care system in Scotland may also have influenced the shape and scope of hospital-at-home functions. Regarding the control cohorts, older people admitted to acute hospital in Scotland receive quite variable care and access to comprehensive geriatric assessment depending on whether they are placed in a geriatric medical unit or other environments such as general adult medicine. This variation may also have influenced the results of this study.

*Implications for clinicians and policy makers*

The variation in intervention costs of the three hospital-at-home services is primarily driven by staff costs, and the findings of the sensitivity analysis confirms that staff costs are likely to determine whether a hospital-at-home service leads to higher costs or cost savings. The skill-mix of healthcare professionals who provide hospital-at-home should be guided by national standards, the type of patients the service targets, and the function of the service in terms of whether or not the service supplements existing community based healthcare, substitutes for hospital level care, augments palliative care services or a combination of these. The integration of these types of service with



For peer review only - http://bmjopen.bmj.com/site/about/guidelines.xhtml

existing primary and secondary care services, for example the provision of out-of-hours care by primary care services, might also determine the costs of these services. Managerial capacity of these services is expected to be of crucial importance in setting-up and managing the team of professionals able to provide high quality care.

The absence of evidence based guidelines about who and under which conditions a patient may be admitted to admission avoidance hospital-at-home might explain the variation in the set-up of services, the difference in patient characteristics between patients admitted to hospital-at-home and hospital, and the relatively small size of the services. This is confirmed by the National Audit of Intermediate Care, [17] that was established in response to concerns about governance structures in intermediate care services, and reported a complex pattern of service provision.

Data on the role and capability of informal care givers is largely absent. In many cases, people admitted to hospital-at-home services receive care from their partners who if old might have health issues themselves.

*Strengths and limitations*

The strengths of this study include the dataset from three of the largest Health Boards in Scotland, the quasi-experimental study design that has allowed inferences from real world evidence, and the sensitivity analyses that helped to address uncertainty in the results. The major limitation of this type of non-randomised comparison is residual confounding. While matching individuals and performing regression analysis can reduce this risk, it is possible that the two populations differed in frailty because we did not match and adjust for differences in the use of community and social services prior to index admission. If unobserved confounders were part of the clinical-decision making by GPs and geriatricians to admit patients to hospital-at-home or hospital, our findings might be biased due to confounding by clinical indication. This type of confounding is often not measured directly because standardised criteria are not available to guide clinical decision-making.[18 19] Therefore, the magnitude of this bias in our results depends on the clinical-decision making process to admit patients to hospital-at-home in the three sites. If clinicians did not consider hospital-at-home as a substitute service to hospitalisation then confounding by indication would increase the residual confounding in our analysis. GPs and geriatricians who refer patients to hospital-at-home are likely to have a clinical bias in preferring to keep older, frailer and terminally ill patients in their own home. Using hospital-at-home admission criteria to define the control cohort accepts that such open criteria will include general medical patients who are likely to have fewer comorbidities, be younger and with a longer life expectancy. However, as the results of the survivors' subgroup analysis were very similar with the results of the main cost analysis we expect that the magnitude of the residual confounding to be small.


For peer review only - http://bmjopen.bmj.com/site/about/guidelines.xhtml

Furthermore, the use of routine data has been used to reliably identify older people with frailty,[20] and approaches using clinical codes to define this population are being tested.[21]

*Future research*

Guidance on the use of real life data to evaluate service delivery interventions is largely absent, and could provide healthcare decision-makers with a relatively inexpensive way of evaluating local service innovations and how to avoid pitfalls in analysis and interpretations. Similar to all observational studies, the findings of this study may be used to identify important questions to be tested in randomised trials.[18] A multi-centre randomised trial that measures outcomes that are key to decision-makers (including informal care giving), and is accompanied by a process evaluation to help explain the findings, is necessary to provide clinicians and policy makers with further evidence about the effectiveness and cost-effectiveness of admission avoidance hospital-at-home services across UK. The authors are involved in such a trial the results of which are expected to be available in 2019.[22]

## Conclusions

We found differences in the populations admitted to hospital-at-home and hospital. The likely higher cost in all three hospital-at-home cohorts, compared with the hospital cohorts during the six months following discharge, highlights the importance of characterising populations eligible to receive these types of healthcare services and of assessing subsequent use of health, social, and informal care following admission to hospital-at-home or hospital. The lack of data on the severity of the observed acute and chronic conditions as well as on type of hospitalised care received in the control cohorts means that we cannot rule out the risk of residual confounding, and the findings should be interpreted with caution.

## Competing interests

All authors have completed the ICMJE uniform disclosure form. GE is leading one of the hospital-at-home services in this study. All other authors declare no support from any organisation for the submitted work; no financial relationships with any organisations that might have an interest in the submitted work in the previous three years; no other relationships or activities that could appear to have influenced the submitted work.

## Details of contributors

AT, GE, and SS were responsible for study concept, GE and SB facilitated the acquisition of data; AT and SS led the writing of the protocol, study design and drafting of the manuscript; AT performed the



For peer review only - http://bmjopen.bmj.com/site/about/guidelines.xhtml

statistical analysis. PL and DS provided clinical expertise and commented on previous versions of the manuscript. All authors interpreted the data, critically revised the manuscript for important intellectual content and approved the final version for submission. AT and SS are guarantors.

## Acknowledgement

We would like to thank Charmaine Walker, Jenny Boyd, Alistair Smith and Josh Matthews from ISD Scotland for providing us with the data as well as Christine McGregor (economist in the Scottish Government) for her insightful views and expertise. We are also indebted to Dr Mike Gardner and Prof Alastair Gray (both University of Oxford), Prof Stavros Petrou (University of Warwick), and Dr Matthew Sperrin (University of Manchester) for commenting on previous drafts of the manuscript. Our thanks also to Prof Gillian Parker (University of York), Dr Angela Coulter (University of Oxford) and Prof Stuart Parker (University of Newcastle) for their useful reflection on the study findings. Finally, we would like to thank all healthcare staff in all three sites who made this study happen.

## Ethical approval

We obtained local data transfer agreements and signed release forms from each Health Board's Caldicott guardian. Further approval from an ethics committee was not required because the study was part of a service audit and the data provided to the researchers was de-identified.

## Funding

NIHR, UK. (12/5003//01; "How to Implement Cost-Effective Comprehensive Geriatric Assessment")

## Data sharing agreement

No additional data are available.

## References

1. World Health Organization. Noncommunicable diseases progress monitor 2015. Geneva: World Health Organization, 2015.
2. Shepperd S, Iliffe S, Doll HA, et al. Admission avoidance hospital at home. *Cochrane Database Syst Rev* 2016;9:CD007491. doi: 10.1002/14651858.CD007491.pub2
3. Sherman RE, Anderson SA, Dal Pan GJ, et al. Real-World Evidence - What Is It and What Can It Tell Us? *N Engl J Med* 2016;375(23):2293-97. doi: 10.1056/NEJMsb1609216

For peer review only



For peer review only - http://bmjopen.bmj.com/site/about/guidelines.xhtml

4. Johns B, Baltussen R, Hutubessy R. Programme costs in the economic evaluation of health interventions. *Cost effectiveness and resource allocation : C/E* 2003;1(1):1.
5. Stuart EA. Matching methods for causal inference: A review and a look forward. *Statistical science : a review journal of the Institute of Mathematical Statistics* 2010;25(1):1-21. doi: 10.1214/09-STS313
6. Garrido MM, Kelley AS, Paris J, et al. Methods for constructing and assessing propensity scores. *Health Serv Res* 2014;49(5):1701-20. doi: 10.1111/1475-6773.12182
7. Baser O. Too much ado about propensity score models? Comparing methods of propensity score matching. *Value Health* 2006;9(6):377-85. doi: 10.1111/j.1524-4733.2006.00130.x
8. Craig P, Cooper C, Gunnell D, et al. Using natural experiments to evaluate population health interventions: new Medical Research Council guidance. *J Epidemiol Community Health* 2012;66(12):1182-6. doi: 10.1136/jech-2011-200375
9. Rubin DB. Using propensity scores to help design observational studies: application to the tobacco litigation. *Health Services & Outcomes Research Methodology* 2001;2:169-88.
10. Funk MJ, Westreich D, Wiesen C, et al. Doubly robust estimation of causal effects. *Am J Epidemiol* 2011;173(7):761-7. doi: 10.1093/aje/kwq439
11. Leist AK. Social Inequalities in Dementia Care, Cure, and Research. *J Am Geriatr Soc* 2017;65(5):1100-01. doi: 10.1111/jgs.14893
12. World Health Organization. Draft global action plan on the public health response to dementia, 2016.
13. Iliffe S. Hospital at home: from red to amber?. Data that will reassure advocates-but without satisfying the sceptics. *Bmj* 1998;316(7147):1761-2.
14. Steventon A, Bardsley M, Billings J, et al. The role of matched controls in building an evidence base for hospital-avoidance schemes: a retrospective evaluation. *Health Serv Res* 2012;47(4):1679-98. doi: 10.1111/j.1475-6773.2011.01367.x
15. Lewis G, Vaithianathan R, Wright L, et al. Integrating care for high-risk patients in England using the virtual ward model: lessons in the process of care integration from three case sites. *Int J Integr Care* 2013;13:e046.
16. Young J, Gladman JR, Forsyth DR, et al. The second national audit of intermediate care. *Age Ageing* 2015;44(2):182-4. doi: 10.1093/ageing/afu174
17. NHS Benchmarking Network. Summary report- England. National audit of intermediate care: NHS Benchmarking Network, 2017.
18. Freemantle N, Marston L, Walters K, et al. Making inferences on treatment effects from real world data: propensity scores, confounding by indication, and other perils for the unwary in observational research. *Bmj* 2013;347:f6409. doi: 10.1136/bmj.f6409
19. Wong AY, Root A, Douglas IJ, et al. Cardiovascular outcomes associated with use of clarithromycin: population based study. *Bmj* 2016;352:h6926. doi: 10.1136/bmj.h6926
20. Clegg A, Bates C, Young J, et al. Development and validation of an electronic frailty index using routine primary care electronic health record data. *Age Ageing* 2017 doi: 10.1093/ageing/afx001
21. Ham C, York N, Sutch S, et al. Hospital bed utilisation in the NHS, Kaiser Permanente, and the US Medicare programme: analysis of routine data. *Bmj* 2003;327(7426):1257. doi: 10.1136/bmj.327.7426.1257
22. Shepperd S, Cradduck-Bamford A, Butler C, et al. A multi-centre randomised trial to compare the effectiveness of geriatrician-led admission avoidance hospital at home versus inpatient admission. *Trials* 2017;18(1):491. doi: 10.1186/s13063-017-2214-y

For peer review only

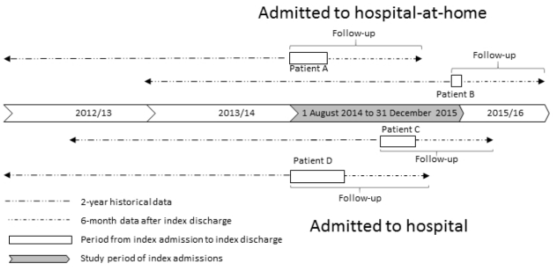


Figure 1. Illustration of obtained data from site one

338x190mm (96 x 96 DPI)

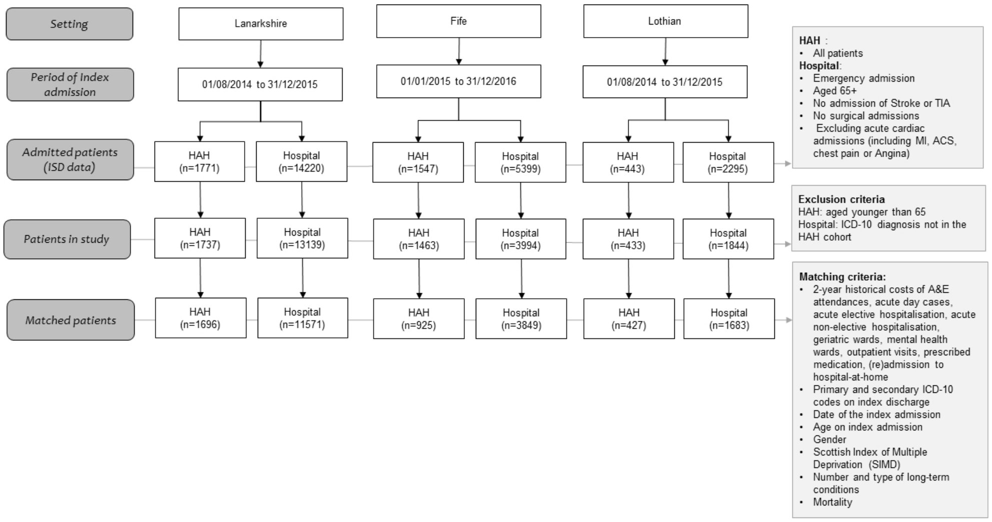


Figure 2 Flowchart of study population

338x190mm (96 x 96 DPI)

Figure 3. Survival curves before and after propensity score matching

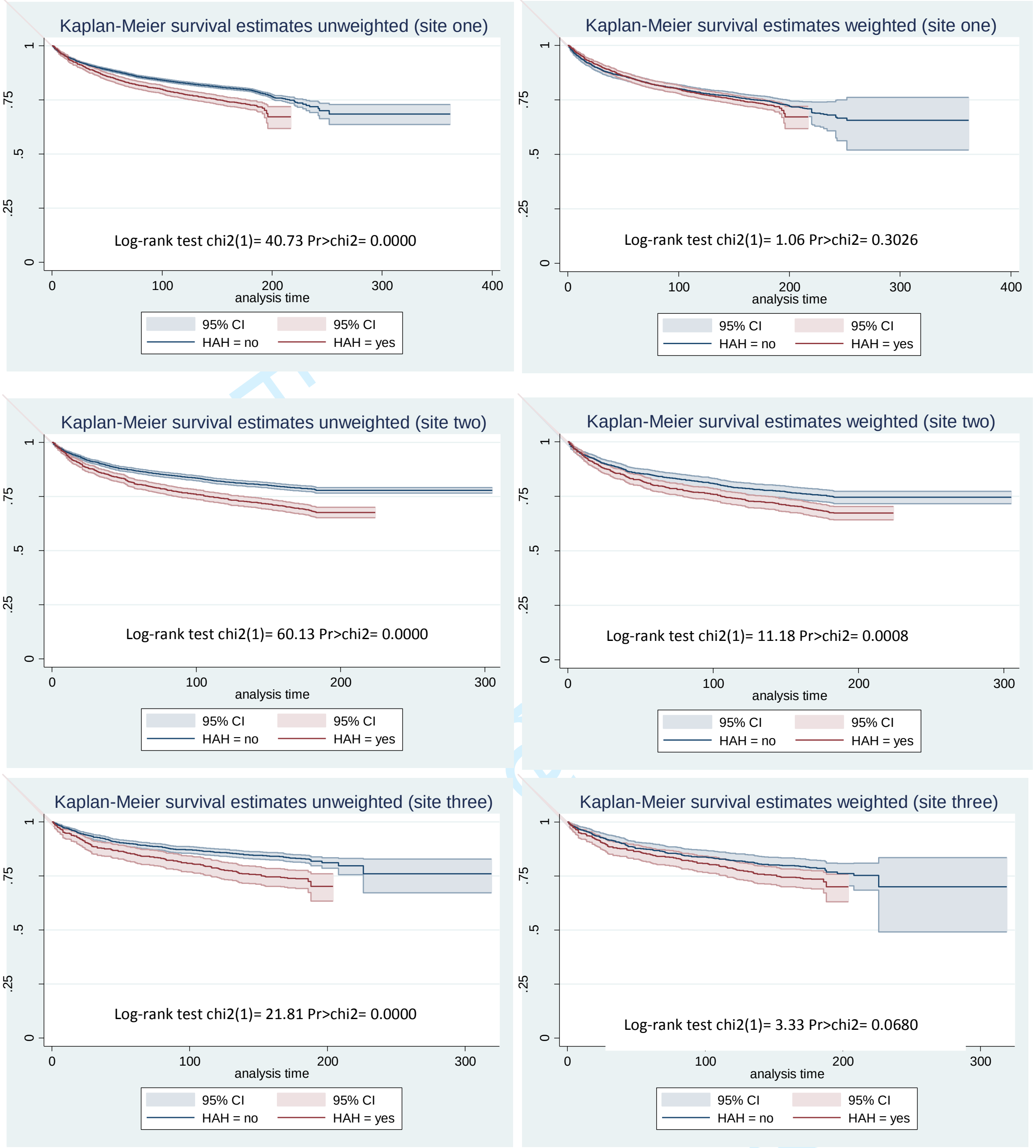


Note: The cohorts in each site were matched on age, gender, socio-economic status, primary and secondary ICD-10 codes of index admission, type of long-term condition, 2-year costs prior to the index admission (by cost category as listed in Box 1); Weighted refers to weighting the observation of each patient based on the propensity score to be in the hospital-at-home cohort as described in the propensity score matching section.

## Appendix 1. Calculation of admission avoidance hospital-at-home in each site

**Site one**

| | | | | | |
|---|---|---|---|---|---|
| | | | **PERIOD** | | |
| **from:** | 01/08/2014 | | **Until:** | 01/01/2016 | **17** |
| | (dd/mm/yyyy) | | (dd/mm/yyyy) | | Months |

| | | | Source of |
|---|---|---|---|
| **Number of HAH admissions (in period)** | 1771 | | ISD IPD data (1/8/14-31/12/15) |
| **Length of HAH stay per episode (in** | 5.53886 | Mean | ISD IPD data (1/8/14-31/12/15) |
| | 0.125605 | Standard error | |
| **HAH bed days (period)** | 9809 | | |

| No | Profession | WTEs | Gross | Summary salary | Source of | Total |
|---|---|---|---|---|---|---|
| **A.1.** | **Staff costs** | | | | | |
| a) | Medical staff | | | | | |
| 1 | Consultant | 1.50 | £151,596 | | Business | £227,394 |
| 2 | Agency consultant | 0.16 | £156,926 | | Business | £25,651 |
| 3 | Consultant | 1.07 | £119,710 | | Business | £127,767 |
| b) | Nursing and pharmacy services | | | | | |
| 1 | Band 3 nurse | 3.00 | £24,790 | | Business | £74,369 |
| 2 | Band 6 nurse | 1.49 | £41,425 | | Business | £61,740 |
| 3 | Band 5 Bank nurse | 0.71 | £32,885 | | Business | £23,399 |
| 4 | Band 6 Bank nurse | 0.36 | £38,471 | | Business | £13,687 |
| 5 | Band 7 pharmacist | 0.71 | £55,491 | | Business | £39,484 |
| 6 | Band 5 nurse | 0.16 | £37,036 | | Business | £6,054 |
| 7 | Band 6 nurse | 1.42 | £42,342 | | Business | £60,303 |
| 8 | Band 7 nurse | 1.00 | £42,444 | | Business | £42,444 |
| 9 | Band 8a nurse | 0.71 | £53,126 | | Business | £37,801 |
| c) | Allied health professions | | | | | |
| 1 | Band 6 occupational therapist | 2.59 | £35,489 | | Business | £91,793 |
| 2 | Band 6 physiotherapist | 1.16 | £46,585 | | Business | £54,200 |
| 3 | Band 4 assistant practitioners for rehab | 3.59 | £24,660 | | Business | £88,444 |
| 4 | Band 6 physiotherapy | 0.71 | £46,848 | | Business | £33,334 |
| d) | Administration, ICT and management | | | | | |
| 1 | Band 2 admin/clerical | 0.30 | £19,346 | | Business | £5,804 |
| 2 | Band 3 admin/clerical | 1.00 | £23,948 | | Business | £23,948 |
| 3 | Band 3 admin/clerical | 0.71 | £21,353 | | Business | £15,193 |
| e) | Support services staff | | | | | |
| 1 | | | | | | £0 |
| | **Total** | | | | | £1,052,80 |
| **A.2.** | **Trainning costs**<br>Note: the time to attend a course should be included in | | | | | |
| No. | Profession | Number of | Cost per | Summary costs | Source of | **Total** |
| 1 | Acute urgent care course | 20 | £250 | | | £5,000 |
| 2 | Prescribing course | 3 | £310 | | | £930 |
| | **Total** | | | | | £5,930 |
| **A.3.** | **Transport costs** | | | | | |
| No. | Cost item | Number of | Cost per item | Summary costs | Source of | **Total** |
| 1 | Travel and subsistence | | | £37,918 | Business | £37,918 |
| | **Total** | | | | | £37,918 |
| **A.4.** | **Information and communication costs**<br>**(e.g. brochures and leaflets for patients and their** | | | | | |
| No. | Cost item | Number of | Cost per item | Summary costs | Source of | **Total** |
| 1 | | | | | | £0 |
| | **Total** | | | | | £0 |
| **A.5.** | **Clinical materials/equipment and** | | | | | |
| No. | Cost item | Number of | Cost per item | Summary costs | Source of | **Total** |
| 1 | Instruments and sundries | | | £2,867 | Business | £2,867 |

| | | | | | | |
|---|---|---|---|---|---|---|
| 2 | Equipment repairs clinical | | | £585 | Business | £585 |
| 3 | Surgical appliances | | | £104 | Business | £104 |
| 4 | Drugs | | | £1,693 | Business | £1,693 |
| 5 | Equipment purchase clinical | | | £298 | Business | £298 |
| | **Total** | | | | | £5,546 |
| **A.6.** | **Support services supplies** | | | | | |
| No. | Cost item | Number of | Cost per item | Summary costs | Source of | **Total** |
| 1 | Catering | | | £177 | Business | £177 |
| 2 | Uniforms | | | £552 | Business | £552 |
| 3 | Printing and stationery | | | £737 | Business | £737 |
| 4 | Dressings | | | £473 | Business | £473 |
| 5 | general services | | | £16 | Business | £16 |
| | **Total** | | | | | £1,955 |
| **A.7.** | **Labs and diagnostics** | | | | | |
| No. | Cost item | Number of | Cost per item | Summary costs | Source of | **Total** |
| 1 | Diagnostic supplies | | | £559 | Business | £559 |
| | | | | | | £559 |
| **A.8.** | **Overhead costs** | | | | | |
| No. | Cost item | Number of | Cost per item | Summary costs | Source of | **Total** |
| 1 | Telephone | | | £3,794 | Business | £3,794 |
| 2 | Building | | | £119 | Business | £119 |
| 3 | Miscellaneous | | | £34 | Business | £34 |
| | **Total** | | | | | £3,947 |
| **A.9.** | **Other costs** | | | | | |
| No. | Cost item | Number of | Cost per item | Summary costs | Source of | **Total** |
| 1 | Equipment purchase non medical | | | £3,354 | Business | £3,354 |
| 2 | postage | | | £772 | Business | £772 |
| | **Total** | | | | | £4,126 |
| **A.10.** | **Additional costs** | | | | | |
| No. | Cost item | Number of | Cost per item | Summary costs | Source of | **Total** |
| 1 | | | | | | £0 |
| | **Total** | | | | | £0 |
| | | | | | **TOTAL** | **£1,112,79** |
| | | | | **Unit cost of HAH admission** | | **£628.34** |
| | | | | **Unit cost of HAH bed day** | | **£113.44** |

**Site two**

| | | | | | | |
|---|---|---|---|---|---|---|
| | from: | 01/01/2015 | **PERIOD** Until: | 01/01/2017 | | **24** |
| | | (dd/mm/yyyy) | (dd/mm/yyyy) | | | Months |
| | | | Source of | | | |
| **Number of HAH admissions (in period)** | 1547 | | ISD IPD data | | | |
| **Length of HAH stay per episode (in** | 7.35 | Mean | ISD IPD data | | | |
| | 0.14 | Standard error | | | | |
| **HAH bed days (period)** | 11376 | | | | | |

| No | Profession | WTEs | Gross | Summary salary | Source of | Total |
|---|---|---|---|---|---|---|
| **A.1.** | **Staff costs** | | | | | |
| a) | Medical staff | | | | | |
| 1 | Senior medical | | | £82,099 | Business | £82,099 |
| 2 | Professional fees and charges | | | £124,391 | Business | £124,391 |
| b) | Nursing and pharmacy services | | | | | |
| 1 | Nursing & Midwifery-trained | | | £2,904,576 | Business | £2,904,57 |
| 2 | Nursing & Midwifery-untrained | | | £627,532 | Business | £627,532 |
| 3 | Pharmacists | | | £43,715 | Business | £43,715 |
| 4 | Pharmacy Technicians | | | £14,471 | Business | £14,471 |
| c) | Allied health professions | | | | | |
| 1 | | | | | Business | £0 |
| d) | Administration, ICT and management | | | | | |
| 1 | Admin Clerical | | | £126,018 | Business | £126,018 |
| e) | Support services staff | | | | | |
| 1 | | | | | | £0 |
| | **Total** | | | | | £3,922,80 |

| No. | Profession | Number of | Cost per | Summary costs | Source of | Total |
|---|---|---|---|---|---|---|
| **A.2.** | **Trainning costs** Note: the time to attend a course should be included in | | | | | |
| 1 | Trainning costs | | | £1,512 | | £1,512 |
| | **Total** | | | | | £1,512 |

| No. | Cost item | Number of | Cost per item | Summary costs | Source of | Total |
|---|---|---|---|---|---|---|
| **A.3.** | **Transport costs** | | | | | |
| 1 | Transport | | | £25,711 | Business | £25,711 |
| 2 | Travel And Subsistence | | | £340,388 | | £340,388 |
| | **Total** | | | | | £366,099 |

| No. | Cost item | Number of | Cost per item | Summary costs | Source of | Total |
|---|---|---|---|---|---|---|
| **A.4.** | **Information and communication costs (e.g. brochures and leaflets for patients and their** | | | | | |
| 1 | | | | | | £0 |
| | **Total** | | | | | £0 |

| No. | Cost item | Number of | Cost per item | Summary costs | Source of | Total |
|---|---|---|---|---|---|---|
| **A.5.** | **Clinical materials/equipment and** | | | | | |
| 1 | Drugs | | | £203,900 | Business | £203,900 |
| 2 | Equipment | | | £14,589 | Business | £14,589 |
| 3 | Paramedical Supplies | | | £3,015 | Business | £3,015 |
| 4 | Surgical Appliances | | | £18 | Business | £18 |
| 5 | Surgical Sundries | | | £80,855 | Business | £80,855 |
| | **Total** | | | | | £302,377 |

| No. | Cost item | Number of | Cost per item | Summary costs | Source of | Total |
|---|---|---|---|---|---|---|
| **A.6.** | **Support services supplies** | | | | | |
| 1 | Bedding And Linen | | | £112 | Business | £112 |
| 2 | Cleaning | | | £8,251 | Business | £8,251 |
| 3 | General Services | | | £2,595 | | £2,595 |
| | **Total** | | | | | £10,958 |

| No. | Cost item | Number of | Cost per item | Summary costs | Source of | Total |
|---|---|---|---|---|---|---|
| **A.7.** | **Labs and diagnostics** | | | | | |
| 1 | Cssd/diagnostic Supplies | | | £3,783 | | £3,783 |

| No. | Cost item | Number of | Cost per item | Summary costs | Source of | Total |
|---|---|---|---|---|---|---|
| | | | | | | £3,783 |
| **A.8.** | **Overhead costs** | | | | | |
| No. | Cost item | Number of | Cost per item | Summary costs | Source of | **Total** |
| 1 | Post Carriage And Telephones | | | £5,224 | | £5,224 |
| 2 | Printing And Stationery | | | £5,737 | Business | £5,737 |
| 3 | Property Maintenance | | | £1,174 | | £1,174 |
| 4 | Miscellaneous | | | £25 | Business | £25 |
| | **Total** | | | | | £12,160 |
| **A.9.** | **Other costs** | | | | | |
| No. | Cost item | Number of | Cost per item | Summary costs | Source of | **Total** |
| 1 | Provisions | | | £6 | Business | £6 |
| 2 | Uniforms | | | £334 | Business | £334 |
| | **Total** | | | | | £340 |
| **A.10.** | **Additional costs** | | | | | |
| No. | Cost item | Number of | Cost per item | Summary costs | Source of | **Total** |
| 1 | Other Operating Income** | | | -£92,377 | | -£92,377 |
| | **Total** | | | | | -£92,377 |
| | | | | | **TOTAL** | **£4,527,65** |
| | | | | **Unit cost of HAH admission** | | **£2,926.73** |
| | | | | **Unit cost of HAH bed day** | | **£398.01** |

For peer review only

| | | | | | | |
|---|---|---|---|---|---|---|
| **Site three** | | | | | | |
| | | from: | 01/01/2015 (dd/mm/yyyy) | PERIOD Until: (dd/mm/yyyy) | 01/01/2016 | 12 Months |
| | | | | Source of | | |
| | Number of HAH admissions (in period) | 598 | | ISD IPD data | | |
| | | 598 | | business case | | |
| | Length of HAH stay per episode (in days) | 7.35 | Mean | ISD IPD data | | |
| | | 0.14 | Standard error | | | |
| | HAH bed days (period) | 4397 | | | | |
| A.1. | Staff costs | | | | | |
| No. | Profession | WTEs | Gross annual | Summary salary cost | Source of | Total |
| a) | Medical staff | | | | | |
| 1 | Consultant | 1 | | £114,776 | Business | £114,77 |
| 2 | Specialty doctor | 1 | | £79,224 | Business | £79,224 |
| 3 | | | | | Business | £0 |
| 4 | | | | | | £0 |
| 5 | | | | | | £0 |
| b) | Nursing and pharmacy services | | | | | |
| 1 | Nurse (Band 6) | 3 | | £125,484 | Business | £125,48 |
| 2 | Nurse (Band 5) | 1.6 | | £53,256 | Business | £53,256 |
| c) | Allied health professions | | | | | |
| 1 | Occupational therapist | 1 | | £45,156 | Business | £45,156 |
| 2 | Physiotherapist | 1 | | £45,156 | Business | £45,156 |
| d) | Administration, ICT and management | | | | | |
| 1 | Admin Clerical | 1 | | £23,664 | Business | £23,664 |
| e) | Support services staff | | | | | |
| 1 | | | | | | £0 |
| | Total | | | | | £486,71 |
| A.2. | Trainning costs<br>Note: the time to attend a course should be included in | | | | | |
| No. | Profession | Number of | Cost per | Summary costs | Source of | Total |
| 1 | Trainning costs | | | £1,000 | | £1,000 |
| | Total | | | | | £1,000 |
| A.3. | Transport costs | | | | | |
| No. | Cost item | Number of | Cost per item | Summary costs | Source of | Total |
| 1 | Transport/travel | | | £20,000 | Business | £20,000 |
| | Total | | | | | £20,000 |
| A.4. | Information and communication costs<br>(e.g. brochures and leaflets for patients and their family) | | | | | |
| No. | Cost item | Number of | Cost per item | Summary costs | Source of | Total |
| 1 | | | | | | £0 |
| | Total | | | | | £0 |
| A.5. | Clinical materials/equipment and drugs | | | | | |
| No. | Cost item | Number of | Cost per item | Summary costs | Source of | Total |
| 1 | Drugs | | | £4,840 | Business | £4,840 |
| 2 | Medical supplies | | | £2,393 | Business | £2,393 |
| | Total | | | | | £7,233 |
| A.6. | Support services supplies | | | | | |
| No. | Cost item | Number of | Cost per item | Summary costs | Source of | Total |
| 1 | | | | | | £0 |
| | Total | | | | | £0 |
| A.7. | Labs and diagnostics | | | | | |
| No. | Cost item | Number of | Cost per item | Summary costs | Source of | Total |
| 1 | | | | | | £0 |
| | | | | | | £0 |
| A.8. | Overhead costs | | | | | |
| No. | Cost item | Number of | Cost per item | Summary costs | Source of | Total |

| | | | | | | |
|---|---|---|---|---|---|---|
| 1 | Phones, stationary etc. | | | £1,796 | Business | £1,796 |
| | Total | | | | | £1,796 |
| A.9. | Other costs | | | | | |
| No. | Cost item | Number of | Cost per item | Summary costs | Source of | Total |
| 1 | Mischellaneous | | | £250 | | £250 |
| | Total | | | | | £250 |
| A.10 | Additional costs | | | | | |
| No. | Cost item | Number of | Cost per item | Summary costs | Source of | Total |
| 1 | | | | | | £0 |
| | Total | | | | | £0 |

| | |
|---|---|
| TOTAL | £516,99 |
| Unit cost of HAH admission | £864.54 |
| Unit cost of HAH bed day | £117.57 |

For peer review only

Appendix 2 Results of selecting PSM technique and plots of covariance balance before and after propensity score matching

| | Site one | | Site two | | Site three | |
|---|---|---|---|---|---|---|
| *Variable* | *Costs* mean/median bias;Rubin's B/R | *Survival* mean/median bias;Rubin's B/R | *Costs* mean/median bias;Rubin's B/R | *Survival* mean/median bias;Rubin's B/R | *Costs* mean/median bias;Rubin's B/R | *Survival* mean/median bias;Rubin's B/R |
| Mahalanobis | 7.5/4.2;51.4/1.56 | 7.2/3.7;48.6/1.54 | 7.6/6.7;46.1/1.54 | 7.3/6.7;43.9/1.53 | 6.3/4.7/38.4/1.69 | 6.3/3.5/38.4/1.52 |
| 1-to-1 | 2.9/2.8;14.1/0.90 | 1.9/1.6;12.1/0.84 | 1.4/1.4;9.4/0.97 | 2.2/2.2;14.6/1.14 | 2.7/2.7/14.6/1.02 | 2.3/2.6/14.9/0.73 |
| K-to-1 | 1.9/1.6;11.3/0.76 | 1.9/1.5;12.0/0.81 | 1.8/1.5;11.0/0.83 | 2.4/2.4;13.6/0.76 | 3.6/2.9/16.5/0.99 | 2.8/2.0/16.5/0.94 |
| Kernel | 1.6/1.1;9.8/0.97 | 1.5/1.2;8.9/0.92 | 1.1/0.9;6.9/1.02 | 0.9/0.7;6.5/1.01 | 2.2/1.6/12.3/1.22 | 1.9/1.2/11.2/1.21 |
| Local linear regression | 1.5/1.2;9.4/0.89 | 1.6/1.4;9.4/0.89 | 1.7/1.0;11.0/0.32 | 2.3/1.4;12.8/0.43 | 1.8/1.6/9.6/1.27 | 1.6/1.2/8.5/1.35 |
| Spline | 2.9/2.6;15.7/0.94 | 2.4/2.0;14.9/0.91 | 3.2/2.6;17.5/0.46 | 3.2/2.3;21.0/1.07 | 3.9/3.1/21.6/0.47 | 3.9/2.3/25.7/1.02 |
| IPW | 11.5/5.8;83.2/0.76 | 11.5/5.6;83.1/0.75 | 11.6/8.3;61.3/0.92 | 11.2/7.8;60.2/0.89 | 10.5/8.5/52.2/0.77 | 10.2/8.5/50.9/0.77 |

Rubin's B: the absolute standardized difference of the means of the linear index of the propensity score in the treated and (matched) non-treated group; Rubin's R: the ratio of treated to (matched) non-treated variances of the propensity score index; Samples sufficiently balanced if B less than 25 and that R between 0.5 and 2.

Standardised percentage bias before and after local linear regression propensity score matching for costs in site one

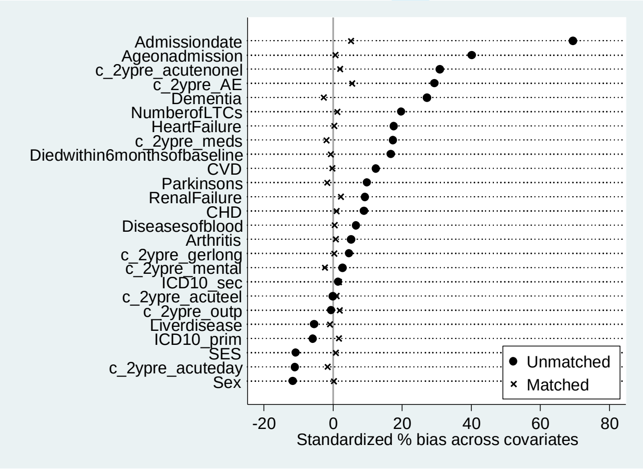


Standardised percentage bias before and after local linear regression propensity score matching for survival in site one

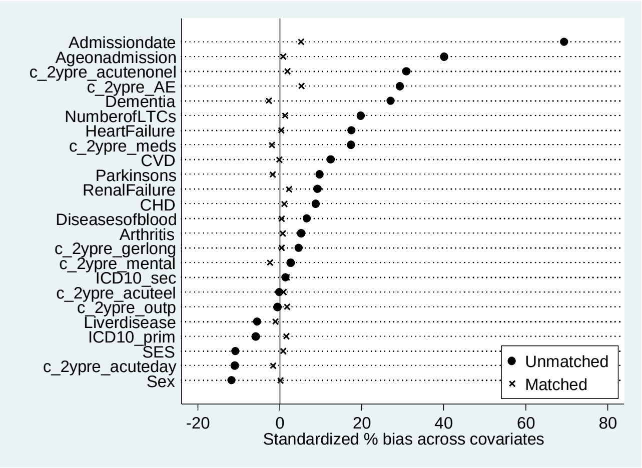

Standardised percentage bias before and after Kernel propensity score matching for costs in site two

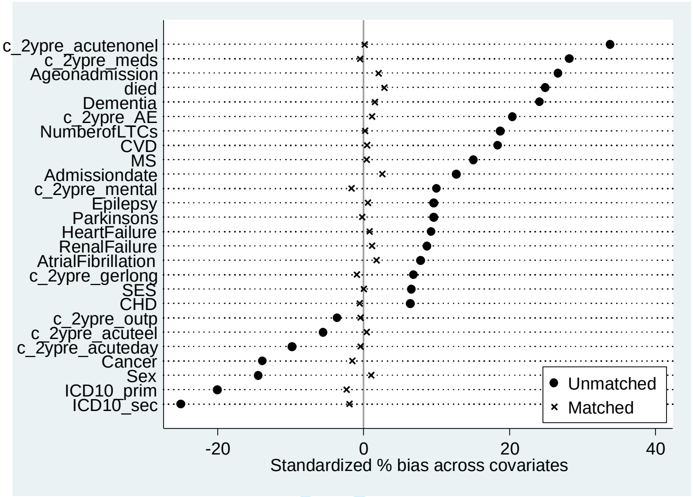


Standardised percentage bias before and after Kernel propensity score matching for survival in site two

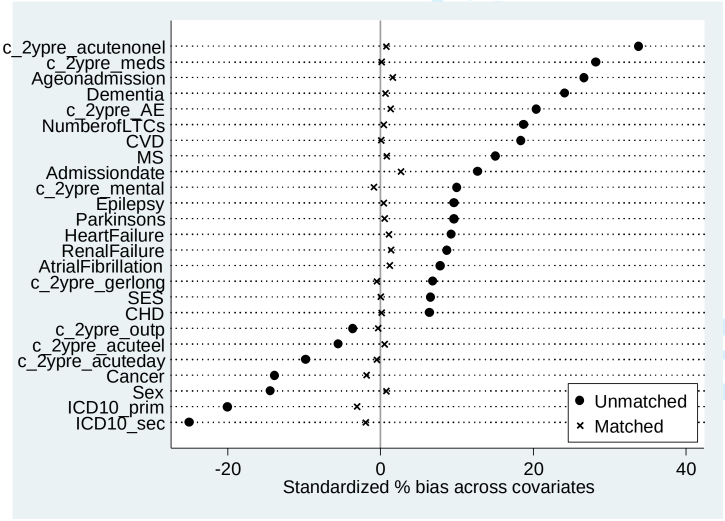


For peer review only

Standardised percentage bias before and after local linear regression propensity score matching for costs in site three

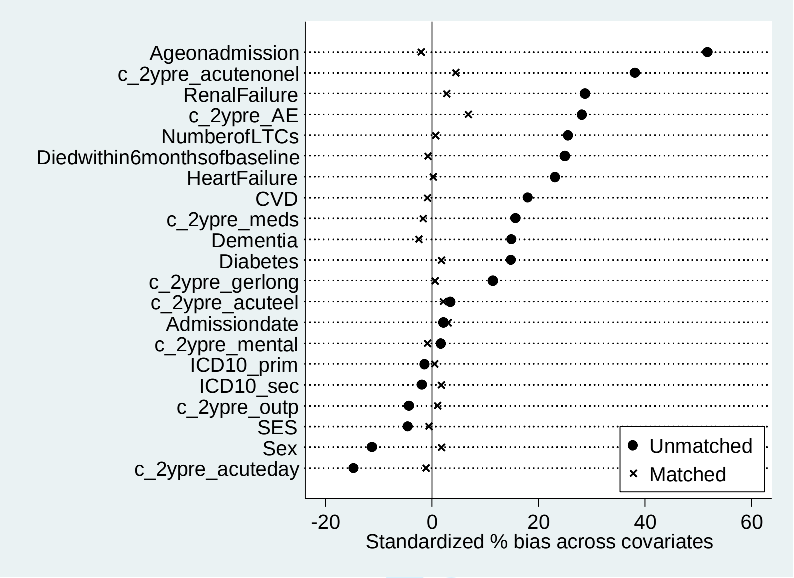


Standardised percentage bias before and after local linear regression propensity score matching for survival in site three

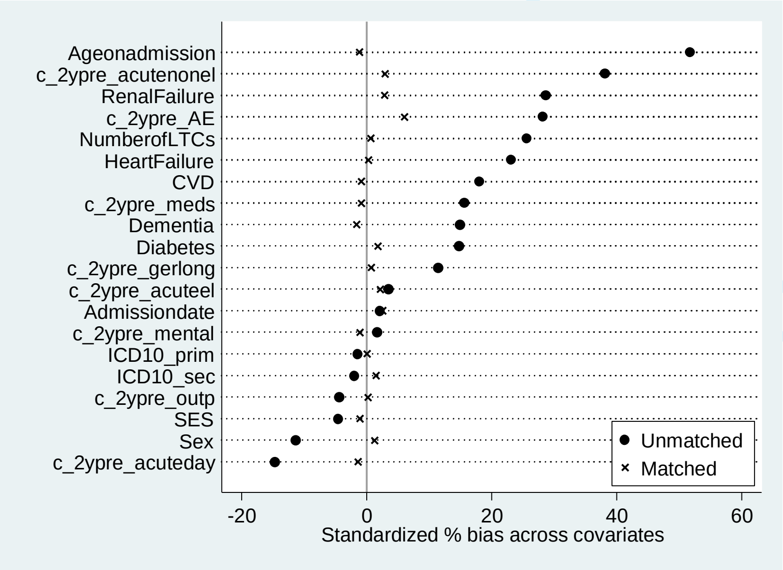

Propensity score distributions by cohort in each site

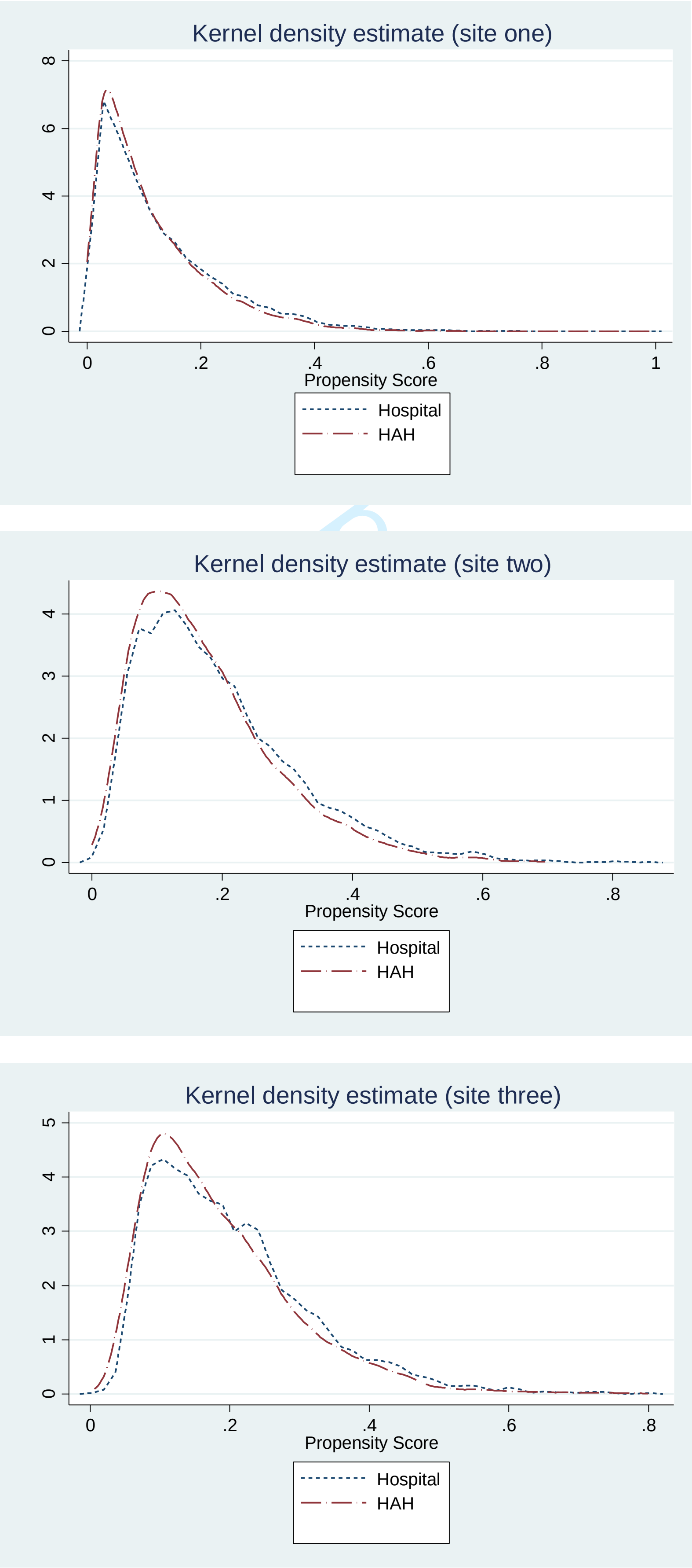

Patient characteristics at index admission after propensity score matching

| | Site one | | Site two | | Site three | |
|---|---|---|---|---|---|---|
| *Variable* | Control | HAH | Control | HAH | Control | HAH |
| Mean age on admission (sd) | 81.2 (7.95) | 81.2 (7.20) | 82.2 (8.03) | 82.4 (7.68) | 81.6 (7.96) | 81.4 (7.10) |
| Female | 63% | 63% | 62% | 62% | 62% | 61% |
| Higher than 4 on the SIMD | 35% | 35% | 53% | 52% | 44% | 44% |
| More than 4 chronic conditions | 44% | 45% | 48% | 50% | 43% | 43% |
| Arthritis | 29% | 29% | 38% | 38% | 33% | 36% |
| Asthma | 10% | 11% | 13% | 14% | 9% | 11% |
| Atrial fibrillation | 29% | 28% | 32% | 33% | 30% | 29% |
| Cancer | 28% | 28% | 28% | 27% | 30% | 28% |
| CVD | 27% | 27% | 26% | 26% | 27% | 26% |
| Liver disease | 3% | 3% | 4% | 4% | 4% | 5% |
| COPD | 27% | 29% | 26% | 28% | 26% | 30% |
| Dementia | 26% | 25% | 26% | 26% | 18% | 17% |
| Diabetes | 23% | 23% | 23% | 24% | 26% | 26% |
| Epilepsy | 4% | 4% | 5% | 5% | 2% | 2% |
| CHD | 42% | 42% | 40% | 40% | 37% | 32% |
| Heart failure | 23% | 23% | 22% | 23% | 25% | 25% |
| MS | 0% | 0% | 1% | 1% | 1% | 1% |
| Parkinson's | 4% | 4% | 3% | 3% | 3% | 5% |
| Renal failure | 22% | 23% | 24% | 24% | 25% | 25% |
| Congenital problems | 2% | 2% | 4% | 4% | 2% | 2% |
| Diseases of blood | 32% | 32% | 30% | 27% | 29% | 29% |
| Endocrine metabolic disease | 36% | 36% | 46% | 45% | 39% | 35% |
| Disease of digestive system | 70% | 72% | 70% | 70% | 64% | 66% |

HAH: hospital-at-home; SIMD ranges from 1 (most deprived) to 10 (most affluent); Note: a patient could be registered with more than one ICD-10 codes

For peer review only

## Appendix 3. Full results of the regression analyses

### Association of hospital at home with total costs (after propensity score matching)

| | **site one (n=13,267)** | | **site two (n=4,769)** | | **site three (n=2110)** | |
|---|---|---|---|---|---|---|
| | Follow-up period coefficient (se) [95%CI] p value | 6 months after discharge coefficient (se) [95%CI] p value | Follow-up period coefficient (se) [95%CI] p value | 6 months after discharge coefficient (se) [95%CI] p value | Follow-up period coefficient (se) [95%CI] p value | 6 months after discharge coefficient (se) [95%CI] p value |
| HAH | 0.82 (0.03) [0.76;0.89] <0.001 | 1.27 (0.07) [1.14;1.41] <0.001 | 1.00 (0.05) [0.92;1.09] 0.982 | 1.09 (0.07) [0.95;1.24] 0.219 | 1.15 (0.09) [0.99;1.33] 0.073 | 1.70 (0.17) [1.4;2.07] <0.001 |
| Admission date | 1.00 (0.00) [1.00;1.00] 0.058 | 1.00 (0.00) [1.00;1.00] 0.009 | 1.00 (0.00) [1.00;1.00] 0.386 | 1.00 (0.00) [1.00;1.00] 0.824 | 1.00 (0.00) [1.00;1.00] 0.009 | 1.00 (0.00) [1.00;1.00] 0.056 |
| ICD10 primary | 1.00 (0.00) [1.00;1.00] 0.660 | 1.00 (0.00) [1.00;1.00] 0.230 | 1.00 (0.00) [1.00;1.00] 0.001 | 1.00 (0.00) [1.00;1.00] <0.001 | 1.00 (0.00) [1.00;1.00] 0.162 | 1.00 (0.00) [1.00;1.00] 0.101 |
| ICD10 secondary | 1.00 (0.00) [1.00;1.00] 0.641 | 1.00 (0.00) [1.00;1.00] 0.988 | 1.00 (0.00) [1.00;1.00] 0.146 | 1.00 (0.00) [1.00;1.00] 0.238 | 1.00 (0.00) [1.00;1.00] 0.897 | 1.00 (0.00) [1.00;1.00] 0.971 |
| 2yrs pre AE costs | 1.00 (0.00) [1.00;1.00] 0.240 | 1.00 (0.00) [1.00;1.00] 0.018 | 1.00 (0.00) [1.00;1.00] 0.624 | 1.00 (0.00) [1.00;1.00] 0.309 | 1.00 (0.00) [1.00;1.00] 0.284 | 1.00 (0.00) [1.00;1.00] 0.42 |
| 2yrs pre elective costs | 1.00 (0.00) [1.00;1.00] 0.906 | 1.00 (0.00) [1.00;1.00] 0.919 | 1.00 (0.00) [1.00;1.00] 0.588 | 1.00 (0.00) [1.00;1.00] 0.435 | 1.00 (0.00) [1.00;1.00] 0.865 | 1.00 (0.00) [1.00;1.00] 0.931 |
| 2yrs pre non-elective costs | 1.00 (0.00) [1.00;1.00] <0.001 | 1.00 (0.00) [1.00;1.00] 0.001 | 1.00 (0.00) [1.00;1.00] 0.694 | 1.00 (0.00) [1.00;1.00] 0.697 | 1.00 (0.00) [1.00;1.00] 0.018 | 1.00 (0.00) [1.00;1.00] 0.015 |
| 2yrs pre day case costs | 1.00 (0.00) [1.00;1.00] 0.098 | 1.00 (0.00) [1.00;1.00] 0.020 | 1.00 (0.00) [1.00;1.00] 0.005 | 1.00 (0.00) [1.00;1.00] <0.001 | 1.00 (0.00) [1.00;1.00] 0.14 | 1.00 (0.00) [1.00;1.00] 0.100 |
| 2yrs pre geriatric ward costs | 1.00 (0.00) [1.00;1.00] 0.005 | 1.00 (0.00) [1.00;1.00] 0.054 | 1.00 (0.00) [1.00;1.00] 0.001 | 1.00 (0.00) [1.00;1.00] 0.003 | 1.00 (0.00) [1.00;1.00] 0.634 | 1.00 (0.00) [1.00;1.00] 0.342 |
| 2yrs pre mental ward costs | 1.00 (0.00) [1.00;1.00] 0.880 | 1.00 (0.00) [1.00;1.00] 0.911 | 1.00 (0.00) [1.00;1.00] 0.009 | 1.00 (0.00) [1.00;1.00] 0.014 | 1.00 (0.00) [1.00;1.00] 0.111 | 1.00 (0.00) [1.00;1.00] 0.382 |
| 2yrs pre outpatient costs | 1.00 (0.00) [1.00;1.00] 0.087 | 1.00 (0.00) [1.00;1.00] 0.056 | 1.00 (0.00) [1.00;1.00] 0.026 | 1.00 (0.00) [1.00;1.00] 0.043 | 1.00 (0.00) [1.00;1.00] 0.683 | 1.00 (0.00) [1.00;1.00] 0.656 |
| 2yrs pre medication costs | 1.00 (0.00) [1.00;1.00] 0.798 | 1.00 (0.00) [1.00;1.00] 0.750 | 1.00 (0.00) [1.00;1.00] 0.172 | 1.00 (0.00) [1.00;1.00] 0.369 | 1.00 (0.00) [1.00;1.00] 0.687 | 1.00 (0.00) [1.00;1.00] 0.935 |
| Died during follow-up | 1.03 (0.04) [0.95;1.11] 0.530 | 0.91 (0.05) [0.82;1.01] 0.089 | 1.05 (0.05) [0.96;1.15] 0.302 | 0.90 (0.06) [0.78;1.05] 0.143 | 1.06 (0.09) [0.90;1.24] 0.498 | 0.97 (0.11) [0.78;1.21] 0.784 |
| Number of LTCs | 1.09 (0.02) [1.05;1.12] <0.001 | 1.12 (0.02) [1.07;1.16] <0.001 | 1.04 (0.02) [1.00;1.07] 0.054 | 1.06 (0.03) [1.00;1.11] 0.035 | 1.06 (0.03) [1.01;1.11] 0.017 | 1.10 (0.03) [1.03;1.17] 0.003 |
| Age on admission | 1.00 (0.00) [0.99;1.01] 0.383 | 1.00 (0.00) [0.99;1.01] 0.981 | 1.00 (0.00) [0.99;1.01] 0.984 | 1.00 (0.00) [1.00;1.00] 0.349 | 1.01 (0.01) [1.00;1.02] 0.045 | 1.01 (0.01) [0.99;1.02] 0.41 |
| Male | 1.09 (0.05) [1.01;1.19] 0.034 | 1.08 (0.06) [0.97;1.19] 0.136 | 0.95 (0.05) [0.86;1.05] 0.340 | 0.99 (0.08) [0.85;1.15] 0.859 | 0.97 (0.08) [0.83;1.13] 0.709 | 0.98 (0.10) [0.81;1.2] 0.875 |
| SES | 1.00 (0.01) [0.98;1.02] 0.988 | 1.00 (0.01) [0.98;1.03] 0.741 | 1.01 (0.01) [1.00;1.03] 0.182 | 1.03 (0.01) [1.00;1.05] 0.033 | 1.00 (0.02) [0.97;1.03] 0.899 | 1.01 (0.02) [0.97;1.05] 0.779 |
| Arthritis | 0.96 (0.04) [0.88;1.05] 0.398 | 0.95 (0.05) [0.85;1.06] 0.346 | ------------ | ------------ | ------------ | ------------ |
| Atrial Fibrillation | ------------ | ------------ | 1.09 (0.06) [0.98;1.2] 0.098 | 1.13 (0.08) [0.97;1.30] 0.113 | ------------ | ------------ |
| Cancer | ------------ | ------------ | 1.04 (0.05) [0.94;1.15] 0.485 | 1.07 (0.08) [0.92;1.24] 0.403 | ------------ | ------------ |
| CVD | 1.01 (0.06) [0.91;1.13] 0.767 | 0.99 (0.07) [0.86;1.13] 0.903 | 1.08 (0.06) [0.97;1.2] 0.168 | 1.11 (0.09) [0.95;1.29] 0.199 | 1.10 (0.11) [0.90;1.34] 0.339 | 1.07 (0.13) [0.84;1.37] 0.585 |
| Liver disease | 1.21 (0.13) [0.98;1.50] 0.074 | 1.20 (0.14) [0.95;1.51] 0.130 | ------------ | ------------ | ------------ | ------------ |
| Dementia | 1.06 (0.05) [0.97;1.17] 0.179 | 1.07 (0.07) [0.95;1.21] 0.236 | 1.00 (0.05) [0.91;1.11] 0.942 | 1.03 (0.08) [0.89;1.19] 0.683 | 1.14 (0.11) [0.95;1.38] 0.166 | 1.17 (0.15) [0.91;1.5] 0.211 |
| Epilepsy | ------------ | ------------ | 1.04 (0.11) [0.85;1.27] 0.734 | 1.04 (0.15) [0.78;1.38] 0.803 | ------------ | ------------ |
| CHD | 0.85 (0.05) [0.77;0.95] 0.004 | 0.83 (0.06) [0.73;0.95] 0.008 | 1.01 (0.06) [0.9;1.13] 0.871 | 1.02 (0.08) [0.88;1.20] 0.766 | ------------ | ------------ |
| Heart Failure | 1.09 (0.06) [0.98;1.20] 0.102 | 1.10 (0.07) [0.97;1.24] 0.154 | 1.08 (0.06) [0.96;1.21] 0.186 | 1.08 (0.09) [0.92;1.28] 0.363 | 1.01 (0.10) [0.83;1.23] 0.919 | 0.98 (0.13) [0.76;1.26] 0.879 |
| Multiple sclerosis | ------------ | ------------ | 0.74 (0.10) [0.57;0.98] 0.033 | 0.59 (0.15) [0.36;0.97] 0.035 | ------------ | ------------ |
| Parkinson's | 1.24 (0.11) [1.03;1.48] 0.019 | 1.20 (0.14) [0.95;1.51] 0.120 | 1.09 (0.15) [0.83;1.42] 0.554 | 1.09 (0.20) [0.75;1.57] 0.664 | ------------ | ------------ |
| Renal Failure | 1.03 (0.05) [0.94;1.13] 0.513 | 1.06 (0.06) [0.94;1.19] 0.362 | 1.05 (0.06) [0.94;1.17] 0.420 | 1.08 (0.09) [0.92;1.26] 0.348 | 1.12 (0.12) [0.9;1.38] 0.306 | 1.14 (0.16) [0.87;1.49] 0.346 |
| Diseases of blood | 1.05 (0.05) [0.96;1.15] 0.275 | 1.05 (0.06) [0.94;1.18] 0.363 | ------------ | ------------ | ------------ | ------------ |
| Diabetes | ------------ | ------------ | ------------ | ------------ | 1.21 (0.11) [1.01;1.45] 0.043 | 1.24 (0.14) [0.99;1.55] 0.061 |
| Constant | 15.93 (46.90) [0.05;5098.92] 0.347 | 0.19 (0.68) [0.00;224.04] 0.644 | 285486.5 (1267507) [47.47; 1.72E+09] 0.005 | 899.53 (5743.23) [0.00;0.00] 0.287 | 207000000000000 (186000000000000) [500612.1;8.6E+20] 0.001 | 2230000000000 (25100000000000) [559.85;8.85E+21] 0.012 |

# driven mainly by non-elective hospital care; Note the HAH unit costs in site one were £628.34 per admission to HAH and have been added to the costs during the episode.

Association of hospital-at-home with mortality risk during study period (after propensity score matching)

| | site one (n=13,267) | site two (n=4,771) | site three (n=2110) |
|---|---|---|---|
| | coefficient (se) [95%CI] p value | coefficient (se) [95%CI] p value | coefficient (se) [95%CI] p value |
| HAH | 1.09 (0.05) [1.00;1.19] 0.059 | 1.29 (0.07) [1.15;1.44] <0.0010 | 1.27 (0.12) [1.06;1.54] 0.011 |
| Admission date | 1.00 (0.00) [1.00;1.00] 0.842 | 1.00 (0.00) [1.00;1.00] 0.100 | 1 (0) [1;1] 0.687 |
| ICD10 primary | 1.00 (0.00) [1.00;1.00] <0.001 | 1.00 (0.00) [1.00;1.00] 0.001 | 1 (0) [1;1] 0.006 |
| ICD10 secondary | 1.00 (0.00) [1.00;1.00] <0.001 | 1.00 (0.00) [1.00;1.00] 0.023 | 1 (0) [1;1] 0.359 |
| 2yrs pre AE costs | 1.00 (0.00) [1.00;1.00] 0.640 | 1.00 (0.00) [1.00;1.00] 0.153 | 1 (0) [1;1] 0.027 |
| 2yrs pre elective costs | 1.00 (0.00) [1.00;1.00] 0.487 | 1.00 (0.00) [1.00;1.00] 0.462 | 1 (0) [1;1] 0.079 |
| 2yrs pre non-elective costs | 1.00 (0.00) [1.00;1.00] 0.001 | 1.00 (0.00) [1.00;1.00] 0.007 | 1 (0) [1;1] 0.052 |
| 2yrs pre day case costs | 1.00 (0.00) [1.00;1.00] <0.001 | 1.00 (0.00) [1.00;1.00] 0.001 | 1 (0) [1;1] 0.903 |
| 2yrs pre geriatric ward costs | 1.00 (0.00) [1.00;1.00] 0.022 | 1.00 (0.00) [1.00;1.00] <0.001 | 1 (0) [1;1] 0.338 |
| 2yrs pre mental ward costs | 1.00 (0.00) [1.00;1.00] 0.419 | 1.00 (0.00) [1.00;1.00] 0.943 | 1 (0) [1;1] 0 |
| 2yrs pre outpatient costs | 1.00 (0.00) [1.00;1.00] 0.091 | 1.00 (0.00) [1.00;1.00] 0.882 | 1 (0) [1;1] 0.001 |
| 2yrs pre medication costs | 1.00 (0.00) [1.00;1.00] 0.044 | 1.00 (0.00) [1.00;1.00] 0.037 | 1 (0) [1;1] 0 |
| Number of LTCs | 1.03 (0.02) [0.99;1.07] 0.120 | 0.96 (0.02) [0.92;1.01] 0.107 | 1.07 (0.04) [1;1.14] 0.048 |
| Age on admission | 1.04 (0) [1.03;1.04] <0.001 | 1.03 (0.00) [1.02;1.04] <0.001 | 1.04 (0.01) [1.02;1.05] 0 |
| Male | 1.12 (0.05) [1.01;1.22] 0.017 | 1.23 (0.08) [1.09;1.39] 0.001 | 1.37 (0.14) [1.12;1.67] 0.002 |
| SES | 0.97 (0.01) [0.95;0.99] 0.001 | 0.98 (0.01) [0.96;1.00] 0.088 | 1.01 (0.02) [0.98;1.05] 0.483 |
| Arthritis | 0.86 (0.05) [0.77;0.97] 0.008 | ------------ | ------------ |
| Atrial Fibrillation | ------------ | 1.11 (0.08) [0.97;1.28] 0.133 | ------------ |
| Cancer | ------------ | 1.86 (0.12) [1.64;2.11] <0.001 | ------------ |
| CVD | 0.94 (0.06) [0.83;1.05] 0.276 | 1.06 (0.08) [0.92;1.22] 0.438 | 0.95 (0.12) [0.74;1.21] 0.673 |
| Liver disease | 1.33 (0.16) [1.04;1.67] 0.015 | ------------ | ------------ |
| Dementia | 1.11 (0.06) [1.00;1.25] 0.058 | 1.59 (0.11) [1.39;1.82] <0.001 | 1.31 (0.16) [1.03;1.67] 0.025 |
| Epilepsy | | 1.19 (0.17) [0.91;1.57] 0.207 | ------------ |
| CHD | 0.91 (0.05) [0.82;1.03] 0.114 | 0.93 (0.07) [0.80;1.08] 0.345 | ------------ |
| Heart Failure | 1.13 (0.07) [1.00;1.28] 0.052 | 1.35 (0.11) [1.15;1.57] <0.001 | 1.16 (0.15) [0.9;1.5] 0.256 |
| Multiple sclerosis | ------------ | 1.54 (0.39) [0.94;2.52] 0.086 | ------------ |
| Parkinson's | 1.11 (0.13) [0.86;1.39] 0.374 | 0.93 (0.17) [0.65;1.33] 0.678 | ------------ |
| Renal Failure | 1.07 (0.07) [0.95;1.21] 0.292 | 1.35 (0.10) [1.16;1.56] <0.001 | 0.93 (0.12) [0.72;1.2] 0.571 |
| Diseases of blood | 0.93 (0.05) [0.85;1.06] 0.201 | ------------ | ------------ |
| Diabetes | ------------ | ------------ | 0.74 (0.1) [0.57;0.97] 0.026 |
| Constant | 0.01 (0.04) [0.00;7.06] 0.174 | 0.00 (0.00) [0.00;0.18] 0.025 | 0 (0) [0;319640.8] 0.405 |

For peer review only

Results of the subgroup analysis including only patients with dementia (costs)

| | site one (n=2,321) | | site two (n=1,053) | | site three (n=280) | |
|---|---|---|---|---|---|---|
| | Follow-up period<br>coefficient (se) [95%CI] p value | Total costs in 6 months after discharge<br>coefficient (se) [95%CI] p value | Follow-up period<br>coefficient (se) [95%CI] p value | Total costs in 6 months after discharge<br>coefficient (se) [95%CI] p value | Follow-up period<br>coefficient (se) [95%CI] p value | Total costs in 6 months after discharge<br>coefficient (se) [95%CI] p value |
| HAH (hospital) | 0.76 (0.05) [0.66;0.87] 0 | 1.18 (0.11) [0.99;1.41] 0.071 | 0.76 (0.06) [0.66;0.88] 0 | 0.75 (0.09) [0.59;0.96] 0.021 | 0.87 (0.15) [0.63;1.21] 0.409 | 1.58 (0.41) [0.95;2.63] 0.078 |
| Admission date | 1 (0) [1;1] 0.528 | 1.00 (0.00) [1.00;1.00] 0.329 | 1 (0) [1;1] 0.513 | 1.00 (0.00) [1.00;1.00] 0.532 | 1 (0) [1;1] 0.002 | 1 (0) [0.99;1] 0.003 |
| ICD10 primary | 1 (0) [1;1] 0.025 | 1.00 (0.00) [1.00;1.00] 0.003 | 1 (0) [1;1] 0.079 | 1.00 (0.00) [1.00;1.00] 0.008 | 1 (0) [1;1] 0.666 | 1 (0) [1;1] 0.123 |
| ICD10 secondary | 1 (0) [1;1] 0.027 | 1.00 (0.00) [1.00;1.00] 0.086 | - - - - - - - - - - - - | - - - - - - - - - - - - | 1 (0) [1;1] 0.946 | 1 (0) [1;1] 0.594 |
| 2yrs pre AE costs | 1 (0) [1;1] 0.063 | 1.00 (0.00) [1.00;1.00] 0.021 | 1 (0) [1;1] 0.979 | 1.00 (0.00) [1.00;1.00] 0.93 | 1 (0) [1;1] 0.57 | 1 (0) [1;1] 0.331 |
| 2yrs pre elective costs | 1 (0) [1;1] 0.913 | 1.00 (0.00) [1.00;1.00] 0.708 | 1 (0) [1;1] 0.979 | 1.00 (0.00) [1.00;1.00] 0.889 | 1 (0) [1;1] 0.115 | 1 (0) [1;1] 0.208 |
| 2yrs pre non-elective costs | 1 (0) [1;1] 0.564 | 1.00 (0.00) [1.00;1.00] 0.605 | 1 (0) [1;1] 0.031 | 1.00 (0.00) [1.00;1.00] 0.008 | 1 (0) [1;1] 0.888 | 1 (0) [1;1] 0.639 |
| 2yrs pre day case costs | 1 (0) [1;1] 0.455 | 1.00 (0.00) [1.00;1.00] 0.632 | 1 (0) [1;1] 0.725 | 1.00 (0.00) [1.00;1.00] 0.307 | 1 (0) [1;1] 0.1 | 1 (0) [1;1] 0.279 |
| 2yrs pre geriatric ward costs | 1 (0) [1;1] 0.233 | 1.00 (0.00) [1.00;1.00] 0.566 | 1 (0) [1;1] 0.012 | 1.00 (0.00) [1.00;1.00] 0.003 | 1 (0) [1;1] 0.907 | 1 (0) [1;1] 0.952 |
| 2yrs pre mental ward costs | 1 (0) [1;1] 0.343 | 1.00 (0.00) [1.00;1.00] 0.335 | 1 (0) [1;1] 0.084 | 1.00 (0.00) [1.00;1.00] 0.042 | 1 (0) [1;1] 0.01 | 1 (0) [1;1] 0.021 |
| 2yrs pre outpatient costs | 1 (0) [1;1] 0.066 | 1.00 (0.00) [1.00;1.00] 0.082 | 1 (0) [1;1] 0.001 | 1.00 (0.00) [1.00;1.00] 0.001 | 1 (0) [1;1] 0.685 | 1 (0) [1;1] 0.403 |
| 2yrs pre medication costs | 1 (0) [1;1] 0.306 | 1.00 (0.00) [1.00;1.00] 0.316 | 1 (0) [1;1] 0.13 | 1.00 (0.00) [1.00;1.00] 0.265 | 1 (0) [1;1] 0.042 | 1 (0) [1;1] 0.044 |
| Died within 6months | 0.81 (0.06) [0.7;0.94] 0.005 | 0.70 (0.07) [0.58;0.85] <0.001 | 0.89 (0.07) [0.76;1.03] 0.118 | 0.73 (0.09) [0.58;0.93] 0.011 | 0.66 (0.13) [0.45;0.96] 0.031 | 0.44 (0.13) [0.25;0.77] 0.004 |
| Number of LTCs | 1.06 (0.03) [1;1.12] 0.069 | 1.07 (0.04) [1.00;1.16] 0.063 | 1.08 (0.03) [1.02;1.14] 0.006 | 1.15 (0.05) [1.05;1.26] 0.003 | 1.04 (0.06) [0.94;1.16] 0.443 | 1.01 (0.08) [0.86;1.18] 0.935 |
| Age on admission | 0.99 (0.01) [0.98;1] 0.094 | 0.98 (0.01) [0.97;1.00] 0.015 | 0.98 (0.01) [0.97;1] 0.007 | 0.97 (0.01) [0.95;0.99] 0.003 | 1 (0.01) [0.98;1.03] 0.933 | 1 (0.02) [0.97;1.03] 0.946 |
| Male | 1.13 (0.08) [0.99;1.31] 0.076 | 1.14 (0.11) [0.95;1.37] 0.151 | 0.95 (0.07) [0.82;1.11] 0.511 | 0.95 (0.12) [0.74;1.22] 0.679 | 1.05 (0.17) [0.76;1.43] 0.78 | 1.07 (0.26) [0.67;1.71] 0.774 |
| SES | 1.01 (0.01) [0.98;1.04] 0.693 | 1.01 (0.02) [0.97;1.04] 0.77 | 1.03 (0.01) [1;1.05] 0.053 | 1.06 (0.02) [1.01;1.10] 0.010 | 1.03 (0.03) [0.97;1.09] 0.3 | 1.04 (0.04) [0.96;1.12] 0.3 |
| Atrial Fibrillation | - - - - - - - - - - - - | - - - - - - - - - - - - | 1.03 (0.09) [0.87;1.23] 0.722 | 1.00 (0.14) [0.77;1.31] 0.986 | - - - - - - - - - - - - | - - - - - - - - - - - - |
| Arthritis | 1.02 (0.09) [0.86;1.2] 0.833 | 1.02 (0.11) [0.83;1.25] 0.862 | - - - - - - - - - - - - | - - - - - - - - - - - - | - - - - - - - - - - - - | - - - - - - - - - - - - |
| Cancer | - - - - - - - - - - - - | - - - - - - - - - - - - | 1.04 (0.1) [0.87;1.24] 0.679 | 1.06 (0.16) [0.79;1.43] 0.688 | - - - - - - - - - - - - | - - - - - - - - - - - - |
| CVD | 0.92 (0.07) [0.78;1.08] 0.3 | 0.91 (0.1) [0.74;1.12] 0.374 | 0.98 (0.08) [0.83;1.16] 0.845 | 0.95 (0.14) [0.72;1.26] 0.741 | 1.39 (0.28) [0.94;2.06] 0.103 | 1.65 (0.48) [0.93;2.91] 0.085 |
| Liver disease | 0.8 (0.12) [0.59;1.08] 0.138 | 0.8 (0.16) [0.54;1.20] 0.286 | - - - - - - - - - - - - | - - - - - - - - - - - - | - - - - - - - - - - - - | - - - - - - - - - - - - |
| CHD | 1.01 (0.09) [0.85;1.2] 0.917 | 1.05 (0.12) [0.84;1.30] 0.688 | 0.94 (0.09) [0.78;1.12] 0.482 | 0.98 (0.14) [0.74;1.30] 0.891 | - - - - - - - - - - - - | - - - - - - - - - - - - |
| Epilepsy | - - - - - - - - - - - - | - - - - - - - - - - - - | 0.97 (0.15) [0.72;1.3] 0.842 | 0.78 (0.16) [0.53;1.16] 0.221 | - - - - - - - - - - - - | - - - - - - - - - - - - |
| Heart Failure | 1.03 (0.11) [0.83;1.27] 0.818 | 1.02 (0.14) [0.79;1.33] 0.878 | 0.92 (0.11) [0.73;1.15] 0.452 | 0.90 (0.17) [0.62;1.29] 0.558 | 0.83 (0.19) [0.53;1.3] 0.409 | 1.16 (0.42) [0.57;2.37] 0.687 |
| Multiple sclerosis | - - - - - - - - - - - - | - - - - - - - - - - - - | 0.4 (0.06) [0.29;0.54] 0 | 0.18 (0.07) [0.09;0.37] <0.001 | - - - - - - - - - - - - | - - - - - - - - - - - - |
| Parkinson's | 1.13 (0.15) [0.88;1.46] 0.333 | 1.00 (0.17) [0.72;1.39] 0.992 | 0.87 (0.14) [0.63;1.18] 0.365 | 0.68 (0.20) [0.39;1.20] 0.188 | - - - - - - - - - - - - | - - - - - - - - - - - - |
| Renal Failure | 1.03 (0.1) [0.85;1.24] 0.769 | 1.12 (0.14) [0.88;1.42] 0.354 | 0.9 (0.09) [0.75;1.09] 0.296 | 0.82 (0.13) [0.60;1.12] 0.203 | 1.2 (0.24) [0.81;1.78] 0.354 | 1.25 (0.35) [0.72;2.17] 0.435 |
| Diseases of blood | 0.93 (0.08) [0.79;1.11] 0.437 | 0.90 (0.1) [0.73;1.11] 0.337 | - - - - - - - - - - - - | - - - - - - - - - - - - | - - - - - - - - - - - - | - - - - - - - - - - - - |
| Diabetes | - - - - - - - - - - - - | - - - - - - - - - - - - | - - - - - - - - - - - - | - - - - - - - - - - - - | 0.85 (0.18) [0.55;1.3] 0.449 | 0.92 (0.26) [0.52;1.6] 0.756 |
| Constant | 469.5 (2319.98) [0.03;7547051] 0.213 | 22.71 (140.52) [0;4194325] 0.614 | 2796754 (19900000) [2.38;3290000000000] 0.037 | 40500000 (472000000) [0;329000000000000000] 0.132 | 2.82E+29 (5.36E+30) [18000000000000;4.43E+45] 0 | 3.34E+38 (9.1E+39) [2100000000000000;5.29E+61] 0.001 |


For peer review only - http://bmjopen.bmj.com/site/about/guidelines.xhtml

Results of the subgroup analysis including only patients with dementia (mortality risk)

| | site one (n=2,321) | site two (n=1,053) | site three (n=280) |
|---|---|---|---|
| | Mortality rate during follow-up coefficient (se) [95%CI] p value | Mortality rate during follow-up coefficient (se) [95%CI] p value | Mortality rate during follow-up coefficient (se) [95%CI] p value |
| HAH (hospital) | 1.05 (0.09) [0.89;1.24] 0.594 | 1.41 (0.12) [1.19;1.67] <0.001 | 1.65 (0.32) [1.12;2.41] 0.011 |
| Admission date | 1.00 (0.00) [1.00;1.00] 0.19 | 1.00 (0.00) [1.00;1.00] 0.001 | 1 (0) [1;1] 0.788 |
| ICD10 primary | 1.00 (0.00) [1.00;1.00] <0.001 | 1.00 (0.00) [1.00;1.00] 0.001 | 1 (0) [1;1] 0.14 |
| ICD10 secondary | 1.00 (0.00) [1.00;1.00] 0.207 | - - - - - - - - - - - - | 1 (0) [1;1] 0.979 |
| 2yrs pre AE costs | 1.00 (0.00) [1.00;1.00] 0.251 | 1.00 (0.00) [1.00;1.00] 0.609 | 1 (0) [1;1] 0.029 |
| 2yrs pre elective costs | 1.00 (0.00) [1.00;1.00] 0.735 | 1.00 (0.00) [1.00;1.00] 0.129 | 1 (0) [1;1] 0.554 |
| 2yrs pre non-elective costs | 1.00 (0.00) [1.00;1.00] 0.173 | 1.00 (0.00) [1.00;1.00] 0.484 | 1 (0) [1;1] 0.814 |
| 2yrs pre day case costs | 1.00 (0.00) [1.00;1.00] 0.088 | 1.00 (0.00) [1.00;1.00] 0.004 | 1 (0) [1;1] 0.896 |
| 2yrs pre geriatric ward costs | 1.00 (0.00) [1.00;1.00] 0.644 | 1.00 (0.00) [1.00;1.00] <0.001 | 1 (0) [1;1] 0.783 |
| 2yrs pre mental ward costs | 1.00 (0.00) [1.00;1.00] 0.569 | 1.00 (0.00) [1.00;1.00] 0.112 | 1 (0) [1;1] 0 |
| 2yrs pre outpatient costs | 1.00 (0.00) [1.00;1.00] 0.070 | 1.00 (0.00) [1.00;1.00] 0.167 | 1 (0) [1;1] 0 |
| 2yrs pre medication costs | 1.00 (0.00) [1.00;1.00] 0.004 | 1.00 (0.00) [1.00;1.00] 0.156 | 1 (0) [1;1] 0.011 |
| Died within 6months | - - - - - - - - - - - - | - - - - - - - - - - - - | - - - - - - - - - - - - |
| Number of LTCs | 0.94 (0.03) [0.88;1.01] 0.113 | 0.95 (0.03) [0.89;1.01] 0.115 | 0.98 (0.07) [0.86;1.13] 0.827 |
| Age on admission | 1.04 (0.01) [1.02;1.05] <0.001 | 1.03 (0.01) [1.01;1.04] <0.001 | 1.04 (0.02) [1;1.07] 0.024 |
| Male | 1.19 (0.11) [0.99;1.42] 0.063 | 1.17 (0.10) [0.99;1.38] 0.070 | 1.18 (0.25) [0.78;1.79] 0.43 |
| SES | 0.97 (0.02) [0.94;1.01] 0.134 | 1.00 (0.02) [0.97;1.03] 0.991 | 0.96 (0.04) [0.88;1.04] 0.3 |
| Atrial Fibrillation | - - - - - - - - - - - - | 1.03 (0.11) [0.85;1.26] 0.75 | - - - - - - - - - - - - |
| Arthritis | 1.06 (0.11) [0.86;1.30] 0.600 | - - - - - - - - - - - - | - - - - - - - - - - - - |
| Cancer | - - - - - - - - - - - - | 1.40 (0.13) [1.16;1.68] <0.001 | - - - - - - - - - - - - |
| CVD | 1.55 (0.41) [0.92;2.61] 0.099 | 1.14 (0.11) [0.94;1.39] 0.176 | 1.02 (0.25) [0.63;1.65] 0.925 |
| Liver disease | 0.98 (0.11) [0.79;1.21] 0.845 | - - - - - - - - - - - - | - - - - - - - - - - - - |
| CHD | 1.21 (0.16) [0.94;1.56] 0.135 | 0.99 (0.10) [0.81;1.20] 0.885 | - - - - - - - - - - - - |
| Epilepsy | - - - - - - - - - - - - | 1.26 (0.19) [0.94;1.70] 0.120 | - - - - - - - - - - - - |
| Heart Failure | 1.21 (0.16) [0.94;1.56] 0.135 | 1.33 (0.17) [1.04;1.70] 0.023 | 1.88 (0.49) [1.12;3.14] 0.017 |
| Multiple sclerosis | - - - - - - - - - - - - | 0.96 (0.51) [0.34;2.72] 0.932 | - - - - - - - - - - - - |
| Parkinson's | 1.26 (0.22) [0.9;1.78] 0.180 | 1.04 (0.20) [0.71;1.51] 0.848 | - - - - - - - - - - - - |
| Renal Failure | 1.06 (0.12) [0.84;1.32] 0.637 | 1.15 (0.12) [0.93;1.41] 0.192 | 0.56 (0.16) [0.32;0.97] 0.037 |
| Diseases of blood | 0.96 (0.11) [0.77;1.19] 0.709 | - - - - - - - - - - - - | - - - - - - - - - - - - |
| Diabetes | - - - - - - - - - - - - | - - - - - - - - - - - - | 0.6 (0.2) [0.32;1.15] 0.123 |
| Constant | 0.00 (0.00) [0.00;1.37] 0.057 | 0.00 (0.00) [0.00;0.00] <0.001 | 0 (0) [0;1810000000000000] 0.652 |



For peer review only - http://bmjopen.bmj.com/site/about/guidelines.xhtml

Results of the subgroup analysis excluding those who had died

| | site one (n=10,132) | | site two (n=3,584) | | site three (n=1691) | |
|---|---|---|---|---|---|---|
| | Follow-up period | Total costs in 6 months after discharge | Follow-up period | Total costs in 6 months after discharge | Follow-up period | Total costs in 6 months after discharge |
| | coefficient (se) [95%CI] p value | coefficient (se) [95%CI] p value | coefficient (se) [95%CI] p value | coefficient (se) [95%CI] p value | coefficient (se) [95%CI] p value | coefficient (se) [95%CI] p value |
| HAH (hospital) | 0.85 (0.04) [0.77;0.94] 0.002 | 1.23 (0.08) [1.08;1.4] 0.002 | 1.11 (0.06) [1;1.25] 0.058 | 1.17 (0.10) [0.99;1.38] 0.070 | 1.20 (0.11) [1;1.43] 0.046 | 1.71 (0.20) [1.36;2.15] <0.001 |
| Admission date | 1 (0) [1;1] 0.076 | 1.00 (0.00) [1.00;1.00] 0.032 | 1 (0) [1;1] 0.833 | 1.00 (0.00) [1.00;1.00] 0.337 | 1 (0) [1;1] 0.075 | 1 (0) [1;1] 0.282 |
| ICD10 primary | 1 (0) [1;1] 0.692 | 1.00 (0.00) [1.00;1.00] 0.993 | 1 (0) [1;1] 0.126 | 1.00 (0.00) [1.00;1.00] 0.038 | 1 (0) [1;1] 0.282 | 1 (0) [1;1] 0.279 |
| ICD10 secondary | 1 (0) [1;1] 0.817 | 1.00 (0.00) [1.00;1.00] 0.473 | 1 (0) [1;1] 0.014 | 1.00 (0.00) [1.00;1.00] 0.024 | 1 (0) [1;1] 0.724 | 1 (0) [1;1] 0.801 |
| 2yrs pre AE costs | 1 (0) [1;1] 0.08 | 1.00 (0.00) [1.00;1.00] 0.012 | 1 (0) [1;1] 0.461 | 1.00 (0.00) [1.00;1.00] 0.135 | 1 (0) [1;1] 0.435 | 1 (0) [1;1] 0.761 |
| 2yrs pre elective costs | 1 (0) [1;1] 0.015 | 1.00 (0.00) [1.00;1.00] 0.046 | 1 (0) [1;1] 0.576 | 1.00 (0.00) [1.00;1.00] 0.429 | 1 (0) [1;1] 0.63 | 1 (0) [1;1] 0.725 |
| 2yrs pre non-elective costs | 1 (0) [1;1] 0 | 1.00 (0.00) [1.00;1.00] <0.001 | 1 (0) [1;1] 0.651 | 1.00 (0.00) [1.00;1.00] 0.700 | 1 (0) [1;1] 0.199 | 1 (0) [1;1] 0.01 |
| 2yrs pre day case costs | 1 (0) [1;1] 0.416 | 1.00 (0.00) [1.00;1.00] 0.158 | 1 (0) [1;1] 0.057 | 1.00 (0.00) [1.00;1.00] 0.023 | 1 (0) [1;1] 0.068 | 1 (0) [1;1] 0.064 |
| 2yrs pre geriatric ward costs | 1 (0) [1;1] 0.031 | 1.00 (0.00) [1.00;1.00] 0.029 | 1 (0) [1;1] 0.625 | 1.00 (0.00) [1.00;1.00] 0.806 | 1 (0) [1;1] 0.484 | 1 (0) [1;1] 0.103 |
| 2yrs pre mental ward costs | 1 (0) [1;1] 0.206 | 1.00 (0.00) [1.00;1.00] 0.166 | 1 (0) [1;1] 0.009 | 1.00 (0.00) [1.00;1.00] 0.020 | 1 (0) [1;1] 0.01 | 1 (0) [1;1] 0.004 |
| 2yrs pre outpatient costs | 1 (0) [1;1] 0.236 | 1.00 (0.00) [1.00;1.00] 0.187 | 1 (0) [1;1] 0.748 | 1.00 (0.00) [1.00;1.00] 0.802 | 1 (0) [1;1] 0.798 | 1 (0) [1;1] 0.908 |
| 2yrs pre medication costs | 1 (0) [1;1] 0.399 | 1.00 (0.00) [1.00;1.00] 0.383 | 1 (0) [1;1] 0.011 | 1.00 (0.00) [1.00;1.00] 0.016 | 1 (0) [1;1] 0.37 | 1 (0) [1;1] 0.77 |
| Number of LTCs | 1.08 (0.02) [1.04;1.12] 0 | 1.12 (0.03) [1.07;1.18] <0.001 | 1.03 (0.02) [0.99;1.08] 0.169 | 1.06 (0.04) [0.99;1.13] 0.076 | 1.06 (0.03) [1.01;1.13] 0.032 | 1.09 (0.04) [1.01;1.17] 0.026 |
| Age on admission | 1.01 (0) [1;1.01] 0.025 | 1.01 (0.00) [1.00;1.02] 0.048 | 1.01 (0) [1;1.01] 0.054 | 1.01 (0.01) [1.00;1.02] 0.254 | 1.02 (0.01) [1;1.03] 0.019 | 1.01 (0.01) [0.99;1.03] 0.171 |
| Male | 1.11 (0.06) [1;1.22] 0.051 | 1.12 (0.07) [0.99;1.26] 0.085 | 0.94 (0.06) [0.83;1.07] 0.353 | 0.97 (0.09) [0.80;1.17] 0.752 | 0.97 (0.09) [0.8;1.16] 0.716 | 1 (0.12) [0.79;1.26] 0.974 |
| SES | 1 (0.01) [0.98;1.02] 0.965 | 1 (0.01) [0.98;1.03] 0.778 | 1.02 (0.01) [1;1.04] 0.081 | 1.03 (0.01) [1.00;1.06] 0.023 | 1 (0.02) [0.96;1.03] 0.822 | 1 (0.02) [0.95;1.05] 0.951 |
| Atrial Fibrillation | ------------ | ------------ | 1.07 (0.07) [0.94;1.21] 0.305 | 1.09 (0.10) [0.92;1.29] 0.335 | ------------ | ------------ |
| Arthritis | 0.99 (0.05) [0.89;1.1] 0.889 | 0.96 (0.06) [0.85;1.1] 0.584 | ------------ | ------------ | ------------ | ------------ |
| Cancer | ------------ | ------------ | 1 (0.07) [0.88;1.15] 0.961 | 1.01 (0.10) [0.84;1.23] 0.899 | ------------ | ------------ |
| CVD | 1.04 (0.07) [0.91;1.2] 0.552 | 1.00 (0.09) [0.85;1.19] 0.956 | 1.14 (0.08) [1;1.3] 0.058 | 1.14 (0.11) [0.95;1.36] 0.174 | 1.12 (0.14) [0.88;1.43] 0.367 | 1.1 (0.17) [0.81;1.5] 0.531 |
| Liver disease | 1.35 (0.2) [1.01;1.8] 0.045 | 1.31 (0.21) [0.95;1.81] 0.097 | ------------ | ------------ | ------------ | ------------ |
| Dementia | 1.16 (0.07) [1.04;1.3] 0.009 | 1.17 (0.08) [1.01;1.35] 0.033 | 1.08 (0.07) [0.96;1.22] 0.195 | 1.11 (0.10) [0.93;1.31] 0.244 | 1.37 (0.16) [1.09;1.73] 0.008 | 1.49 (0.23) [1.09;2.02] 0.011 |
| CHD | 0.82 (0.06) [0.72;0.94] 0.004 | 0.79 (0.07) [0.67;0.93] 0.004 | 1.01 (0.07) [0.87;1.16] 0.941 | 1.03 (0.10) [0.85;1.24] 0.799 | ------------ | ------------ |
| Epilepsy | ------------ | ------------ | 1.08 (0.12) [0.86;1.35] 0.518 | 1.09 (0.17) [0.80;1.48] 0.581 | ------------ | ------------ |
| Heart Failure | 1.1 (0.07) [0.97;1.25] 0.131 | 1.08 (0.08) [0.93;1.26] 0.293 | 1.08 (0.08) [0.94;1.24] 0.287 | 1.07 (0.11) [0.88;1.31] 0.491 | 1.05 (0.13) [0.82;1.34] 0.719 | 1.01 (0.16) [0.74;1.39] 0.932 |
| Multiple sclerosis | ------------ | ------------ | 0.72 (0.14) [0.49;1.06] 0.095 | 0.66 (0.21) [0.35;1.25] 0.202 | ------------ | ------------ |
| Parkinson's | 1.19 (0.1) [1;1.41] 0.05 | 1.15 (0.13) [0.93;1.43] 0.19 | 1.22 (0.18) [0.91;1.64] 0.193 | 1.34 (0.27) [0.91;1.98] 0.139 | ------------ | ------------ |
| Renal Failure | 1.01 (0.06) [0.89;1.14] 0.911 | 1.00 (0.07) [0.87;1.16] 0.949 | 1.06 (0.08) [0.92;1.22] 0.443 | 1.06 (0.11) [0.86;1.29] 0.602 | 1.12 (0.15) [0.86;1.46] 0.411 | 1.19 (0.2) [0.85;1.66] 0.317 |
| Diseases of blood | 1.04 (0.06) [0.94;1.16] 0.414 | 1.04 (0.07) [0.92;1.19] 0.516 | ------------ | ------------ | ------------ | ------------ |
| Diabetes | ------------ | ------------ | ------------ | ------------ | 1.33 (0.15) [1.07;1.65] 0.01 | 1.37 (0.19) [1.04;1.81] 0.026 |
| Constant | 3.67 (13.85) [0;5959] 0.73 | 0.07 (0.31) [0;592.13] 0.558 | 1064.79 (5943.4) [0.02;60000000] 0.212 | 0.89 (6.96) [0;4301665] 0.988 | 101000000000 (1050000000000) [149.57;68100000000000000000] 0.015 | 1320000000 (18000000000) [0;5.67E+20] 0.124 |



For peer review only - http://bmjopen.bmj.com/site/about/guidelines.xhtml

For peer review only

Results of the sensitivity analysis

| | site one (n=13,267) | | site two (n=4,769) | | site three (n=2110) | |
|---|---|---|---|---|---|---|
| | Total costs in follow-up (50% higher HAH unit costs) coefficient (se) [95%CI] p value | Total costs in follow-up (50% lower HAH unit costs) coefficient (se) [95%CI] p value | Total costs in follow-up (50% higher HAH unit costs) coefficient (se) [95%CI] p value | Total costs in follow-up (50% lower HAH unit costs) coefficient (se) [95%CI] p value | Total costs in follow-up (50% higher HAH unit costs) coefficient (se) [95%CI] p value | Total costs in follow-up (50% lower HAH unit costs) coefficient (se) [95%CI] p value |
| HAH (hospital) | 0.87 (0.03) [0.81;0.94] 0.001 | 0.77 (0.03) [0.71;0.84] 0 | 1.18 (0.05) [1.09;1.28] 0 | 0.81 (0.04) [0.74;0.9] 0 | 1.23 (0.09) [1.07;1.42] 0.004 | 1.07 (0.09) [0.91;1.25] 0.399 |
| Admission date | 1 (0) [1;1] 0.071 | 1 (0) [1;1] 0.048 | 1 (0) [1;1] 0.489 | 1 (0) [1;1] 0.3 | 1 (0) [1;1] 0.007 | 1 (0) [1;1] 0.012 |
| ICD10 primary | 1 (0) [1;1] 0.649 | 1 (0) [1;1] 0.671 | 1 (0) [1;1] 0.001 | 1 (0) [1;1] 0.001 | 1 (0) [1;1] 0.167 | 1 (0) [1;1] 0.16 |
| ICD10 secondary | 1 (0) [1;1] 0.588 | 1 (0) [1;1] 0.701 | 1 (0) [1;1] 0.148 | 1 (0) [1;1] 0.145 | 1 (0) [1;1] 0.875 | 1 (0) [1;1] 0.909 |
| 2yrs pre AE costs | 1 (0) [1;1] 0.223 | 1 (0) [1;1] 0.261 | 1 (0) [1;1] 0.687 | 1 (0) [1;1] 0.561 | 1 (0) [1;1] 0.307 | 1 (0) [1;1] 0.267 |
| 2yrs pre elective costs | 1 (0) [1;1] 0.909 | 1 (0) [1;1] 0.904 | 1 (0) [1;1] 0.537 | 1 (0) [1;1] 0.657 | 1 (0) [1;1] 0.896 | 1 (0) [1;1] 0.813 |
| 2yrs pre non-elective costs | 1 (0) [1;1] 0 | 1 (0) [1;1] 0 | 1 (0) [1;1] 0.919 | 1 (0) [1;1] 0.458 | 1 (0) [1;1] 0.015 | 1 (0) [1;1] 0.021 |
| 2yrs pre day case costs | 1 (0) [1;1] 0.099 | 1 (0) [1;1] 0.097 | 1 (0) [1;1] 0.006 | 1 (0) [1;1] 0.004 | 1 (0) [1;1] 0.131 | 1 (0) [1;1] 0.148 |
| 2yrs pre geriatric ward costs | 1 (0) [1;1] 0.006 | 1 (0) [1;1] 0.005 | 1 (0) [1;1] 0.002 | 1 (0) [1;1] 0 | 1 (0) [1;1] 0.562 | 1 (0) [1;1] 0.713 |
| 2yrs pre mental ward costs | 1 (0) [1;1] 0.905 | 1 (0) [1;1] 0.854 | 1 (0) [1;1] 0.005 | 1 (0) [1;1] 0.02 | 1 (0) [1;1] 0.09 | 1 (0) [1;1] 0.132 |
| 2yrs pre outpatient costs | 1 (0) [1;1] 0.086 | 1 (0) [1;1] 0.088 | 1 (0) [1;1] 0.027 | 1 (0) [1;1] 0.026 | 1 (0) [1;1] 0.699 | 1 (0) [1;1] 0.675 |
| 2yrs pre medication costs | 1 (0) [1;1] 0.713 | 1 (0) [1;1] 0.892 | 1 (0) [1;1] 0.136 | 1 (0) [1;1] 0.236 | 1 (0) [1;1] 0.713 | 1 (0) [1;1] 0.663 |
| Died within 6months | 1.03 (0.04) [0.95;1.11] 0.492 | 1.02 (0.04) [0.94;1.12] 0.572 | 1.05 (0.04) [0.97;1.14] 0.252 | 1.05 (0.05) [0.95;1.16] 0.38 | 1.06 (0.08) [0.91;1.23] 0.474 | 1.06 (0.09) [0.89;1.25] 0.517 |
| Number of LTCs | 1.08 (0.02) [1.05;1.11] 0 | 1.09 (0.02) [1.05;1.13] 0 | 1.04 (0.02) [1;1.07] 0.033 | 1.04 (0.02) [0.99;1.08] 0.093 | 1.06 (0.02) [1.01;1.1] 0.016 | 1.06 (0.03) [1.01;1.11] 0.019 |
| Age on admission | 1 (0) [1;1.01] 0.323 | 1 (0) [1;1.01] 0.452 | 1 (0) [1;1.01] 0.788 | 1 (0) [0.99;1.01] 0.789 | 1.01 (0.01) [1;1.02] 0.037 | 1.01 (0.01) [1;1.02] 0.055 |
| Male | 1.09 (0.04) [1.01;1.18] 0.035 | 1.1 (0.05) [1.01;1.2] 0.034 | 0.96 (0.04) [0.88;1.04] 0.311 | 0.95 (0.06) [0.85;1.07] 0.382 | 0.97 (0.07) [0.84;1.12] 0.686 | 0.97 (0.08) [0.82;1.14] 0.704 |
| SES | 1 (0.01) [0.98;1.02] 0.979 | 1 (0.01) [0.98;1.02] 0.954 | 1.01 (0.01) [1;1.02] 0.17 | 1.01 (0.01) [0.99;1.03] 0.205 | 1 (0.01) [0.97;1.03] 0.887 | 1 (0.02) [0.97;1.03] 0.917 |
| Atrial Fibrillation | ------------ | ------------ | 1.08 (0.05) [0.98;1.18] 0.104 | 1.1 (0.06) [0.98;1.23] 0.094 | ------------ | ------------ |
| Arthritis | 0.96 (0.04) [0.89;1.05] 0.392 | 0.96 (0.05) [0.88;1.05] 0.403 | ------------ | ------------ | ------------ | ------------ |
| Cancer | ------------ | ------------ | 1.04 (0.05) [0.95;1.14] 0.426 | 1.03 (0.06) [0.92;1.16] 0.566 | ------------ | ------------ |
| CVD | 1.02 (0.05) [0.92;1.13] 0.743 | 1.02 (0.06) [0.91;1.14] 0.794 | 1.07 (0.05) [0.98;1.18] 0.146 | 1.08 (0.07) [0.96;1.22] 0.199 | 1.09 (0.11) [0.91;1.32] 0.352 | 1.11 (0.12) [0.9;1.37] 0.324 |
| Liver disease | 1.21 (0.13) [0.98;1.48] 0.073 | 1.23 (0.14) [0.98;1.53] 0.074 | ------------ | ------------ | ------------ | ------------ |
| Dementia | 1.07 (0.05) [0.97;1.17] 0.16 | 1.07 (0.05) [0.97;1.18] 0.2 | 1.02 (0.05) [0.93;1.11] 0.738 | 0.99 (0.06) [0.88;1.1] 0.795 | 1.14 (0.11) [0.95;1.37] 0.153 | 1.14 (0.12) [0.94;1.4] 0.18 |
| CHD | 0.86 (0.05) [0.77;0.95] 0.004 | 0.85 (0.05) [0.76;0.95] 0.005 | 1.07 (0.05) [0.97;1.18] 0.174 | 1.02 (0.06) [0.9;1.15] 0.785 | ------------ | ------------ |
| Epilepsy | ------------ | ------------ | 1.04 (0.1) [0.86;1.26] 0.664 | 1.02 (0.12) [0.82;1.28] 0.841 | ------------ | ------------ |
| Heart Failure | 1.09 (0.05) [0.99;1.2] 0.095 | 1.09 (0.06) [0.98;1.21] 0.11 | 1.07 (0.06) [0.96;1.19] 0.201 | 1.09 (0.07) [0.96;1.24] 0.177 | 1.01 (0.1) [0.83;1.22] 0.947 | 1.02 (0.11) [0.82;1.25] 0.885 |
| Multiple sclerosis | ------------ | ------------ | 0.76 (0.1) [0.59;0.98] 0.033 | 0.73 (0.11) [0.54;0.99] 0.046 | ------------ | ------------ |
| Parkinson’s | 1.23 (0.11) [1.04;1.45] 0.018 | 1.24 (0.12) [1.03;1.49] 0.021 | 1.07 (0.14) [0.84;1.37] 0.582 | 1.11 (0.18) [0.81;1.52] 0.512 | ------------ | ------------ |
| Renal Failure | 1.04 (0.05) [0.95;1.13] 0.436 | 1.03 (0.05) [0.93;1.13] 0.601 | 1.04 (0.05) [0.94;1.15] 0.408 | 1.06 (0.07) [0.94;1.2] 0.366 | 1.11 (0.11) [0.91;1.36] 0.3 | 1.12 (0.13) [0.9;1.39] 0.317 |
| Diseases of blood | 1.05 (0.05) [0.97;1.14] 0.246 | 1.05 (0.05) [0.96;1.15] 0.308 | ------------ | ------------ | ------------ | ------------ |
| Diabetes | ------------ | ------------ | ------------ | ------------ | 1.2 (0.11) [1;1.42] 0.044 | 1.22 (0.12) [1.01;1.48] 0.042 |
| Constant | 26.62 (74.48) [0.11;6410.63] 0.241 | 8.84 (27.52) [0.02;3945.99] 0.484 | 295178.8 (1199605) [102.52;850000000] 0.002 | 1223534 (6192074) [60.23;24900000000] 0.006 | 14800000000000 (127000000000000) [776224.7;2.84E+20] 0 | 31000000000(2920000000) [292677.5;3.28E+21] 0.001 |

For peer review only

For peer review only


For peer review only - http://bmjopen.bmj.com/site/about/guidelines.xhtml

**The RECORD statement – checklist of items, extended from the STROBE statement, that should be reported in observational studies using routinely collected health data.**

| | Item No. | STROBE items | Location in manuscript where items are reported | RECORD items | Location in manuscript where items are reported |
|---|---|---|---|---|---|
| **Title and abstract** | | | | | |
| | 1 | (a) Indicate the study's design with a commonly used term in the title or the abstract (b) Provide in the abstract an informative and balanced summary of what was done and what was found | 1a: page 1<br><br>1b: page 2 | RECORD 1.1: The type of data used should be specified in the title or abstract. When possible, the name of the databases used should be included.<br><br>RECORD 1.2: If applicable, the geographic region and timeframe within which the study took place should be reported in the title or abstract.<br><br>RECORD 1.3: If linkage between databases was conducted for the study, this should be clearly stated in the title or abstract. | 1.1: page 1<br><br>1.2: page 2<br><br>1.3: page 2 |
| **Introduction** | | | | | |
| Background rationale | 2 | Explain the scientific background and rationale for the investigation being reported | page 4 | | |
| Objectives | 3 | State specific objectives, including any prespecified hypotheses | page 4 | | |
| **Methods** | | | | | |
| Study Design | 4 | Present key elements of study | page 5 | | |

For peer review only

| | | | | | |
|---|---|---|---|---|---|
| | | design early in the paper | | | |
| Setting | 5 | Describe the setting, locations, and relevant dates, including periods of recruitment, exposure, follow-up, and data collection | page 5-6 | | |
| Participants | 6 | *(a) Cohort study* - Give the eligibility criteria, and the sources and methods of selection of participants. Describe methods of follow-up<br>*Case-control study* - Give the eligibility criteria, and the sources and methods of case ascertainment and control selection. Give the rationale for the choice of cases and controls<br>*Cross-sectional study* - Give the eligibility criteria, and the sources and methods of selection of participants<br><br>*(b) Cohort study* - For matched studies, give matching criteria and number of exposed and unexposed<br>*Case-control study* - For matched studies, give matching criteria and the number of controls per case | 6a: NA<br><br>6b: page 6-8 | RECORD 6.1: The methods of study population selection (such as codes or algorithms used to identify subjects) should be listed in detail. If this is not possible, an explanation should be provided.<br><br>RECORD 6.2: Any validation studies of the codes or algorithms used to select the population should be referenced. If validation was conducted for this study and not published elsewhere, detailed methods and results should be provided.<br><br>RECORD 6.3: If the study involved linkage of databases, consider use of a flow diagram or other graphical display to demonstrate the data linkage process, including the number of individuals with linked data at each stage. | 6.1: page 5-6<br><br>6.2 NA<br><br>6.3 NA |
| Variables | 7 | Clearly define all outcomes, exposures, predictors, potential confounders, and effect | page 7-8 | RECORD 7.1: A complete list of codes and algorithms used to classify exposures, outcomes, confounders, and | page 5, 7-8 |

| | | | | | |
|---|---|---|---|---|---|
| | | modifiers. Give diagnostic criteria, if applicable. | | effect modifiers should be provided. If these cannot be reported, an explanation should be provided. | |
| Data sources/ measurement | 8 | For each variable of interest, give sources of data and details of methods of assessment (measurement). Describe comparability of assessment methods if there is more than one group | page 6 | | |
| Bias | 9 | Describe any efforts to address potential sources of bias | page 6-8 | | |
| Study size | 10 | Explain how the study size was arrived at | page 6-8 | | |
| Quantitative variables | 11 | Explain how quantitative variables were handled in the analyses. If applicable, describe which groupings were chosen, and why | page 7-8 | | |
| Statistical methods | 12 | (a) Describe all statistical methods, including those used to control for confounding<br>(b) Describe any methods used to examine subgroups and interactions<br>(c) Explain how missing data were addressed<br>(d) *Cohort study* - If applicable, explain how loss to follow-up was addressed<br>*Case-control study* - If applicable, explain how | 12a: page 7-8<br>12b: page 8<br>12c: page 8<br>12d: 6-8 | | |

For peer review only

| | | | | | |
|---|---|---|---|---|---|
| | | matching of cases and controls was addressed<br>*Cross-sectional study* - If applicable, describe analytical methods taking account of sampling strategy<br>(e) Describe any sensitivity analyses | 12e: page 8 | | |
| Data access and cleaning methods | | .. | | RECORD 12.1: Authors should describe the extent to which the investigators had access to the database population used to create the study population.<br><br>RECORD 12.2: Authors should provide information on the data cleaning methods used in the study. | 12.1: page 5<br><br>12.2: page 6 |
| Linkage | | .. | | RECORD 12.3: State whether the study included person-level, institutional-level, or other data linkage across two or more databases. The methods of linkage and methods of linkage quality evaluation should be provided. | page 6 |
| **Results** | | | | | |
| Participants | 13 | (a) Report the numbers of individuals at each stage of the study (*e.g.*, numbers potentially eligible, examined for eligibility, confirmed eligible, included in the study, completing follow-up, and analysed) | 13a: page 9-10 | RECORD 13.1: Describe in detail the selection of the persons included in the study (*i.e.,* study population selection) including filtering based on data quality, data availability and linkage. The selection of included persons can be described in the text and/or by | page 6, 8, 10 |

For peer review only

| | | | | | |
|---|---|---|---|---|---|
| | | (b) Give reasons for non-participation at each stage.<br>(c) Consider use of a flow diagram | 13b: page 9-10<br><br>13c: page 10 | means of the study flow diagram. | |
| Descriptive data | 14 | (a) Give characteristics of study participants (*e.g.*, demographic, clinical, social) and information on exposures and potential confounders<br>(b) Indicate the number of participants with missing data for each variable of interest<br>(c) *Cohort study* - summarise follow-up time (*e.g.*, average and total amount) | 14a: page 9,12<br><br>14b: NA<br><br>14c: page 6,13 | | |
| Outcome data | 15 | *Cohort study* - Report numbers of outcome events or summary measures over time<br>*Case-control study* - Report numbers in each exposure category, or summary measures of exposure<br>*Cross-sectional study* - Report numbers of outcome events or summary measures | page 13 | | |
| Main results | 16 | (a) Give unadjusted estimates and, if applicable, confounder-adjusted estimates and their precision (e.g., 95% confidence interval). Make clear which confounders were adjusted for and why they were included | 16a: page 13, 15 | | |

For peer review only

| | | | | | |
|---|---|---|---|---|---|
| | | (b) Report category boundaries when continuous variables were categorized<br>(c) If relevant, consider translating estimates of relative risk into absolute risk for a meaningful time period | 16b: NA<br><br>16c: NA | | |
| Other analyses | 17 | Report other analyses done—e.g., analyses of subgroups and interactions, and sensitivity analyses | page 15 | | |
| **Discussion** | | | | | |
| Key results | 18 | Summarise key results with reference to study objectives | page 15 | | |
| Limitations | 19 | Discuss limitations of the study, taking into account sources of potential bias or imprecision. Discuss both direction and magnitude of any potential bias | page 17 | RECORD 19.1: Discuss the implications of using data that were not created or collected to answer the specific research question(s). Include discussion of misclassification bias, unmeasured confounding, missing data, and changing eligibility over time, as they pertain to the study being reported. | page 17 |
| Interpretation | 20 | Give a cautious overall interpretation of results considering objectives, limitations, multiplicity of analyses, results from similar studies, and other relevant evidence | page 18-19 | | |
| Generalisability | 21 | Discuss the generalisability (external validity) of the study | page 15-16 | | |

For peer review only

| | | | | | |
|---|---|---|---|---|---|
| | | results | | | |
| **Other Information** | | | | | |
| Funding | 22 | Give the source of funding and the role of the funders for the present study and, if applicable, for the original study on which the present article is based | page 19 | | |
| Accessibility of protocol, raw data, and programming code | | .. | | RECORD 22.1: Authors should provide information on how to access any supplemental information such as the study protocol, raw data, or programming code. | page 19 |

*Reference: Benchimol EI, Smeeth L, Guttmann A, Harron K, Moher D, Petersen I, Sørensen HT, von Elm E, Langan SM, the RECORD Working Committee. The REporting of studies Conducted using Observational Routinely-collected health Data (RECORD) Statement. *PLoS Medicine* 2015; in press.

*Checklist is protected under Creative Commons Attribution (CC BY) license.

For peer review only

BMJ Open

# Should I stay or should I go? A retrospective propensity score matched analysis using administrative data of hospital-at-home for older people in Scotland

| | |
|---|---|
| Journal: | *BMJ Open* |
| Manuscript ID | bmjopen-2018-023350.R2 |
| Article Type: | Research |
| Date Submitted by the Author: | 19-Feb-2019 |
| Complete List of Authors: | Tsiachristas, Apostolos; University of Oxford, Health Economics Research Centre, Nuffield Department of Population Health<br>Ellis, Graham; Monklands Hospital, NHS Lanarkshire<br>Buchanan, Scott; Information Services Division, National Services Scotland<br>Langhorne, Peter; University of Glasgow, Institute of Cardiovascular and Medical Sciences<br>Stott, David; Institute of Cardiovascular and Medical Sciences, University of Glasgow, Geriatric Medicine<br>Shepperd, S; University of Oxford, Nuffield Department of Population Health |
| <b>Primary Subject Heading</b>: | Geriatric medicine |
| Secondary Subject Heading: | Health economics, Health services research |
| Keywords: | hospital-at-home, admission avoidance, intermediate care, costs, mortality, UK |
| | |

SCHOLARONE™
Manuscripts

# Should I stay or should I go? A retrospective propensity score matched analysis using administrative data of hospital-at-home for older people in Scotland

Apostolos Tsiachristas[1], Graham Ellis[2], Scott Buchanan[3], Peter Langhorne[4], David J Stott[4], Sasha Shepperd[1].

[1] Nuffield Department of Population Health, University of Oxford, Oxford, UK

[2] Monklands Hospital, NHS Lanarkshire, Glasgow, UK

[3] Information Services Division, National Services Scotland, Edinburgh, UK

[4] Institute of Cardiovascular and Medical Sciences, University of Glasgow, Glasgow, UK

Word Count: 4783

Correspondence to: Apostolos Tsiachristas, Health Economics Research Centre, Nuffield Department of Population Health, Richard Doll building, Old Road Campus, OX3 7LF, Oxford, UK; Tel: +44(0)1865 289470; email: apostolos.tsiachristas@dph.ox.ac.uk



For peer review only - http://bmjopen.bmj.com/site/about/guidelines.xhtml

## Abstract

Objectives: To compare the characteristics of populations admitted to hospital-at-home services with the population admitted to hospital and assess the association of these services with healthcare costs and mortality.

Design: In a retrospective observational cohort study of linked patient level data, we used propensity score matching in combination with regression analysis.

Participants: Patients aged 65 years and older admitted to hospital-at-home or hospital.

Interventions: Three geriatrician-led admission avoidance hospital-at-home services in Scotland.

Outcome measures: Healthcare costs and mortality.

Results: Patients in hospital-at-home were older and more socioeconomically disadvantaged, had higher rates of previous hospitalization, and there was a greater proportion of women and people with several chronic conditions compared with the population admitted to hospital. The cost of providing hospital-at-home varied between the three sites from £628 to £2928 per admission. Hospital-at-home was associated with 18% lower costs during the follow-up period in site one (ratio of means 0.82; 95%CI: 0.76-0.89). Limiting the analysis to costs during the 6 months following index discharge, patients in the hospital-at-home cohorts had 27% higher costs (ratio of means 1.27; 95%CI: 1.14-1.41) in site one, 9% (ratio of means 1.09; 95%CI: 0.95-1.24) in site two and 70% in site three (ratio of means 1.70; 95%CI: 1.40-2.07) compared with patients in the control cohorts. Admission to hospital-at-home was associated with an increased risk of death during the follow-up period in all three sites (1.09, 95%CI: 1.00-1.19 site one; 1.29, 95%CI: 1.15-1.44 site two; 1.27, 95%CI: 1.06-1.54 site three).

Conclusions: Our findings indicate that in these three cohorts, the populations admitted to hospital-at-home and hospital differ. We cannot rule out the risk of residual confounding, as our analysis relied on an administrative data set and we lacked data on disease severity and type of hospitalised care received in the control cohorts.

**Strengths and limitations of the study**

- The study used a large dataset from three of the largest Health Boards in Scotland.
- The retrospective cohort study has allowed inferences from real world evidence.
- Various sensitivity analyses helped to address uncertainty in the results.
- The major limitation of this type of non-randomised comparison is residual confounding.
- The lack of data on quality of life, as well as use of subsequent health, social, community and informal care is a limitation.



For peer review only - http://bmjopen.bmj.com/site/about/guidelines.xhtml

For peer review only

For peer review only



For peer review only - http://bmjopen.bmj.com/site/about/guidelines.xhtml

# Introduction

Organising health systems to optimise the health outcomes of older people and contain costs is a priority as populations around the world age, and the demand for healthcare continues to rise. Despite a global policy emphasis on 'care closer to home'[1] and initiatives that seek to ease demand for hospital based healthcare, efforts to innovate and deliver healthcare services that provide an alternative to hospital admission for older people have been piecemeal and often lack a health system perspective. A lack of evidence to support decision-making has contributed to this. Avoiding admission to hospital by providing acute healthcare in people's homes, often as a hospital outreach service, is one of the more popular service innovations and yet there is uncertainty around the effectiveness and cost-effectiveness of this form of care.[2]

Box 1 Description of each service

Hospital-at-home

The three hospital-at-home services are broadly similar, capacity ranged between 24 to 60 beds for the period of the analysis. Each is a geriatrician-led service that is supported by nurses (sometimes nurse practitioners) and therapy practitioners for the initial assessment; geriatricians and the multi-disciplinary team review patients in their homes and meet daily (a virtual ward round) to discuss patient cases and agree actions. Rehabilitation is available within the existing team with onward referral to community rehabilitation as required, and in one site rehabilitation is accessed through a parallel community rehabilitation services. Out of hours emergency cover is provided by primary care out-of-hours. Patients are referred to the service from GPs, sometimes through a central referral number or via step down from the acute hospital. The service offers access to diagnostics such as radiology, and intravenous fluids, antibiotics and oxygen. Cases are discussed daily with the multidisciplinary team at the virtual ward round and daily management plans agreed. In one site there is close working with the day hospital where patients can be referred for follow up or for investigations. Patients access investigations and treatment with the same speed as inpatients. The services support intravenous therapies in the home.

Hospital

The provision of hospital based acute health services varied among the sites; in one site there were three district general hospitals (1,653 beds) that provide acute health services to a mainly urban population of 652,230, with a total of 1,653 beds; in site two a hospital (550 beds) provides acute healthcare to a population of 180,130; and in site three there are two district general hospitals (825 beds) that provide healthcare to a population of 358,900, and acute admissions are via one of the hospitals.

The use of administrative data to evaluate service delivery interventions has the potential to provide a simple and efficient mechanism to provide real-world evidence about policy relevant service innovations, and embed evaluation into local decision-making. However, previous experience of using routine data in this area of research has been of mixed success due to a limited set of variables, missing data and the complexity of policy relevant questions that often require a broad and longer term perspective.[3] Administrative healthcare data collected in Scotland is unique in that it is population based, with little missing data. The aim of this study was to use these data to compare the characteristics of populations from three Health Boards who used a geriatrician-led hospital-at-home service with the population who received hospital care, and to assess the impact of these services on healthcare costs and mortality.



For peer review only - http://bmjopen.bmj.com/site/about/guidelines.xhtml

# Methods

### *Setting*

We used patient level data collected by three of the fourteen Scottish Health Boards of all patients aged 64 years and older, and who were admitted (referred to as the index admission) to either geriatrician-led admission avoidance hospital-at-home or inpatient hospital between August 2014 and December 2015 (17 months) in site one and site two, and between January 2015 and December 2016 (24 months) in site three. These services are commissioned by integrated health and social care boards that cover a population of almost 1.5 million in urban and rural areas. The Information Service Division (ISD), part of NHS Scotland, de-identified, cleaned and linked individual patient records to derive activity and costs related to periods before and after the index admissions. We obtained signed release forms from each Board's Caldicott guardian, and followed the ISD data sharing agreement.

### *Intervention*

The three service models of hospital-at-home provided an admission avoidance function that provided an alternative to inpatient hospital care, and had similar structures and functions; the main differences were in the capacity of the services and the organisation of services for rehabilitation. (Box 1)

### *Data sources*

Data were available for each person for two years prior to their index admission, and from the point of their index admission to six months after index discharge from hospital-at-home or hospital. Box 2 presents a full list of all variables included in the dataset. Figure 1 provides schematic examples of the differing calendar time periods studied before and after index admission for people admitted between August 2014 and December 2015 to hospital-at-home (Patients A and B) or hospital (Patients C and D) in site one. As this illustrates, the maximum follow-up period for each patient consisted of the period between index admission and index discharge and 6 months after index discharge. The data were collected via the data systems used in hospitals to collect patient data. Hospital-at-home activity data was submitted to ISD from the local systems of the three sites. The linked data set included acute inpatient, geriatric long stay and day case, mental health admissions, outpatient appointments accident and emergency attendances, community prescribing and death registrations.

For peer review only

Figure 1. Illustration of obtained data from site one

**Box 2. List of variables included in the dataset**

Costs of accidents and emergency attendances,
Costs of acute day cases,
Costs of acute elective hospitalisation,
Costs of acute non-elective hospitalisation,
Costs of geriatric wards,
Costs of mental health wards,
Costs of outpatient visits,
Costs of prescribed medication,
Costs of (re)admission to hospital-at-home.
Primary ICD-10 codes on index discharge,
Secondary ICD-10 codes on index discharge,
Length of stay of the index admission,
Age on index admission,
Gender,
Scottish Index of Multiple Deprivation (SIMD), 1 (most deprived) to 10 (most affluent)
Long-term conditions,
Date of death (if applicable),
Based on ICD-10 codes:
Cardiovascular disease (CVD) (I60-I69, G45)
Chronic obstructive pulmonary disorder (COPD) (J41-J44, J47),
Dementia (F00-F03, F05.1),
Diabetes (E10-E14),
Coronary heart disease (CHD, ICD10: I20-I25),
Heart failure (I500, I501, I509),
Renal failure (N03, N18, N19, I12, I13),
Epilepsy (G40, G41),
Asthma (J45, J46),
Atrial fibrillation (I48, MS, G35),
Cancer (C00-C97),
Arthritis (M05, M19, M45, M47, M460-M462, M464, M468, M469),
Parkinson's (G20-G22),
Chronic liver disease (K711, K713, K714, K717, K754),
Congenital problems (Q00-Q99),
Diseases of blood and blood forming organs (D50-D89),
Other diseases of the digestive system (K00-K122, K130-K839, K85X, K860-K93),
Other endocrine metabolic diseases (E00-E07, E15-E35, E70-E90)
Admitted to HAH or hospital.

*Selection of patients in the hospital-at-home and control cohorts*

We included patients aged 65 years and older, and who were classified as an unscheduled admission to general or geriatric medicine. In the control cohort, we excluded those with a diagnosis that would not be eligible for management through hospital-at-home; these exclusions included acute intracerebral crisis (intracerebral infections, trauma or haemorrhage), stroke and related codes, acute coronary syndromes and myocardial infarction, surgical emergencies including vascular, urological, gynaecological and general surgical presentations, orthopaedic diagnosis of fractures and trauma, cardiothoracic diagnoses, poisoning and complications of surgery. We also excluded from the control group those who had a diagnosis (i.e. primary and secondary ICD-10 code) that was not observed in any of the hospital-at-home admissions in each site (1081 patients in site one, 1405 in site two and in 451 in site three) (Figure 2). Each patient was counted as a single episode of healthcare.



For peer review only - http://bmjopen.bmj.com/site/about/guidelines.xhtml

*Intervention costs*

We collected data on the costs of hospital-at-home using a template derived from the Cost-It tool of the World Health Organisation.[4] The cost categories included staff, training, transport, information and communication, clinical materials/equipment, support services, laboratory services, diagnostics, overheads and other costs. Clinician managers supported by finance staff in the three Health Boards completed this template based on the actual spending for the hospital-at-home service for the time periods covered by the ISD data. The cost per hospital-at-home admission was calculated by dividing the total costs of the hospital-at-home service by the total number of hospital-at-home admissions during the same period.

*Statistical analysis*

We used an iterative approach to the analysis, starting with a description of the two cohorts (i.e. those admitted to hospital-at-home and those admitted to hospital) for each Health Board. We calculated means, standard deviations, and frequencies to describe differences in patient characteristics at index admission and tested differences using two sample t-test and Mann-Whitney test for continues variables and Chi-square test for categorical variables. We also estimated the mean differences in resource utilisation costs (with bootstrapped standard errors) and the unadjusted relative risk of mortality between the two cohorts for each Health Board.

Further, we investigated the association of being admitted to hospital-at-home or hospital with mortality and cost over a minimum follow-up period of six months. To do this, we followed the Medical Research Council guidelines on performing natural experiments and scientific literature to adopt a step-wise strategy to select the propensity score matching (PSM) technique that most reduced observed confounding between the two cohorts in each Health Board.[5-8] First, we included all possible confounding variables available in the dataset (see Box 2 and Figure 2), and considered that the inclusion of covariates not associated with the treatment assignment would have little influence in the propensity score model.[5] Second, we matched the two cohorts in each site using a range of the most commonly used PSM techniques; these included Mahalanobis, 1-to-1, K-to-1, kernel, local linear regression, spline, and inverse probability weighting techniques. Second, the performance of each PSM technique on covariate balancing was assessed based on the mean and median percentage standardised bias as well as Rubin's B (the absolute standardized difference of the means of the linear index of the propensity score in the treated and (matched) non-treated group) and Rubin's R (the ratio of treated to (matched) non-treated variances of the propensity score index). Following Rubin's (2001) recommendation, we considered B less than 25 and R between 0.5 and 2 to indicate sufficient balance.[9] Third, we chose the PSM technique that had the lowest values on these performance indicators in each of the three Health Boards. We matched the two cohorts in each Health Board by



For peer review only - http://bmjopen.bmj.com/site/about/guidelines.xhtml

socio-demographic characteristics (i.e. age, gender, socio-economic status), diagnosis code (i.e. primary and secondary ICD-10 code) of index admission, morbidity (i.e. type of long-term condition, mortality during follow-up (for the analysis of cost), 2-year costs prior to the index admission (by cost category as listed in Box 1), and date of index admission (to account for seasonal trends).

We performed a doubly robust estimation to further reduce confounding by using a regression analysis after performing the most suitable PSM technique and including the confounding variables listed above as covariates.[10] In the regression, we used generalised linear regression models (GLMs) with gamma distribution and log link to investigate the association of hospital-at-home with total costs during the follow-up period, and total costs in 6 months following index discharge. We also used GLMs with Poisson distribution and log link to estimate the relative risk of mortality. Robust standard errors were specified in all regression models. We calculated Kaplan-Meier survival curves, with and without using the weights from the PSM, and used log-rank tests to test the equality of the survival functions. There were few missing observations in the dataset and thus, complete case analysis was performed.

*Subgroup analysis*

We conducted a sub-group analysis, running the same regression models used in the main analysis, to investigate the association of hospital-at-home services with costs and mortality for the population who had a diagnosis of dementia. We considered this population to be important due to their complex healthcare needs, and the increasing prevalence of dementia.[11 12] In a second subgroup analysis, we excluded patients who died during the follow-up period and investigated the association of hospital-at-home with costs. In both subgroup analyses, propensity score matching was performed to match sub-cohorts in each site.

*Sensitivity analysis*

In a univariate sensitivity analysis, we reduced and increased the intervention cost of admission avoidance hospital-at-home by 50%, as there are no standard unit costs to benchmark these types of services and we were concerned that costs for these services may vary due to economies of scale, size, experience, setting, human resource capacity, and error. This sensitivity analysis was expected to impact the costs during index admission and the costs of admission to hospital-at-home in the six months after discharge. In another sensitivity analysis, we estimated the E-value to assess how strong unmeasured confounding would have to be with both the treatment (i.e. admission to hospital-at-home) and outcome (i.e. costs and mortality) to fully explain away the estimated treatment effects, conditional on the measured confounders.[13 14]

*Patient involvement*

Patients were not involved in this retrospective analysis of administrative data.



For peer review only - http://bmjopen.bmj.com/site/about/guidelines.xhtml

# Results

*Characteristics of the population cohorts*

After applying the exclusion criteria, 1737 patients were admitted to hospital-at-home in site one between August 2014 and December 2015 (17 months), 1463 patients were admitted to hospital-at-home in site two between January 2015 and December 2016 (24 months), and 433 patients were admitted to hospital-at-home in site three between August 2014 and December 2015 (17 months) (Figure 2). In the same period, there were 13139 patients admitted to 3 hospitals in site one, 3994 patients admitted to 1 hospital in site two, and 1844 patients admitted to 1 hospital in site three.

There were few differences between the hospital-at-home cohorts in the three sites, the main difference being that a larger proportion of the population in site two lived in a more affluent area (i.e. scored five or higher on the Scottish Index of Multiple Deprivation) . Patients admitted to hospital-at-home were on average three to four years older than those admitted to hospital, were more likely to be female (range from 5 percentage points to 9 percentage points), and a higher proportion had more than four long-term conditions (approximately 7 percentage points) compared with patients admitted to hospital (Table 1). The largest difference between those admitted to hospital-at-home and to hospital in site one and site two was in the proportion of patients with dementia (10 percentage points higher in the hospital-at-home cohorts), while in site three it was the proportion of patients with renal failure (also 10 percentage points higher in the hospital-at-home cohort).

We compared the two cohorts in each site, from index admission to six months post discharge from hospital-at-home or hospital (Table 2). There was on average a higher percentage of deaths while receiving healthcare in hospital compared with those receiving healthcare in hospital-at-home (6% vs., 1% site one; 6% vs., 3% site two; 4% vs., 1% site three); and a higher percentage of deaths in the follow-up period, from admission to six months after discharge, in the groups that had received hospital-at-home (21% vs., 28% site one; 22% vs., 32% site two; 17% vs., 27% site three). Patients in the hospital-at-home cohort lived on average eight (site one), ten (site two), and twelve (site three) fewer days during the whole follow-up, and their index admission was on average fewer days in site one (mean unadjusted difference -2.64, 95%CI -2.97 to -2.31) and site three (mean unadjusted difference -2.02, 95%CI -2.66 to -1.37) and longer in site two (mean unadjusted difference 1.25, 95% CI 0.86 to 1.64).

The cost during a hospital-at-home admission was on average lower than hospital admission in site one (mean difference -£2318; 95%CI: £-2420 to £-2217) and site three (mean difference -£1096; 95%CI: £-1398 to £-793), and slightly lower (mean difference £-153; 95%CI: £-277; to £-29) in site two (Table 2). In the hospital-at-home cohort, these costs included the intervention costs of delivering the



For peer review only - http://bmjopen.bmj.com/site/about/guidelines.xhtml

service at home, which were £628 per admission and £113 per day in site one, £2928 per admission and £398 per day in site two, and £864.54 per admission and £117.57 per day in site three. In each Health Board, staff were the major driver of the cost of delivering hospital-at-home (site one 95%, site two 87%, site three 94%). Detailed information on the costs of delivering hospital at home are in Appendix 1.

Each of the three hospital-at-home cohorts incurred higher healthcare costs, driven by non-elective hospitalisation, prior to their index admission compared with the respective control cohort. Site one had on average 40% higher costs (mean difference £3219; 95%CI: £2513 to £3925), site two 56% higher costs (mean difference £5064; 95%CI: £3984 to £6143) and site three 57% higher costs (mean difference £4115; 95%CI: £2467 to £5764). In the six months following discharge from the index admission, costs were higher for each of the three hospital-at-home cohorts; in site one costs were on average 43% higher (mean difference £1839; 95%CI: £1423 to £2255), in site two they were 16% higher (mean difference £875, 95%CI: £156 to £1595), and in site three they were 92% higher (mean difference £3068, 95%CI: £2178 to £3958). The larger increase in costs in all sites was due to higher non-elective hospitalisation costs in the group who had received hospital-at-home care (mean difference £1517, 95%CI £1134 to 1899 site one; mean difference £529, 95%CI £-77 to 1135 site two; mean difference £2618, 95%CI £1779 to 3458 site three) during the six months follow-up.

When the cost of the index admission was included in the analysis, the cost during follow-up (i.e. including the index admission and 6-months healthcare resource use after index discharge) was 6% lower (mean difference -£480, 95%CI: £-996 to £36) in the hospital-at-home cohort, compared with the control cohort in site one; while these costs were 8% higher in site two (mean difference £722, 95%CI: £32 to £1413) and 35% higher in site three (mean difference £1973, 95%CI: £1019 to £2927).

Compared with the control cohort, the mean costs per day of being alive during the follow-up period were 13% (mean difference £-12; 95%CI: -17 to -6) lower in the hospital-at-home cohort in site one, while these costs were 34% higher (mean difference £37; 95%CI: 18 to 56) and 66% higher (mean difference £36; 95%CI: 18 to 53) in site two and site three respectively.

Figure 2 Flowchart of study population



For peer review only - http://bmjopen.bmj.com/site/about/guidelines.xhtml

Table 1 Patient characteristics at index admission

| | Site one | | Site two | | Site three | |
|---|---|---|---|---|---|---|
| *Variable* | *Control (n=13139)* | *HAH (n=1737)* | *Control (n=3994)* | *HAH (n=1463)* | *Control (n=1844)* | *HAH (n=433)* |
| Mean age on admission (SD) | 77.8 (7.78) | 81.2 (7.21)** | 78.5 (8.11) | 82.2 (7.82)** | 77.3 (7.81) | 81.4 (7.12)** |
| Female | 7,468 (57%) | 1,096 (63%)** | 2,102 (53%) | 909 (62%)** | 1037 (56%) | 266 (61%)* |
| Higher than 4 on the SIMD | 5,005 (38%) | 609 (35%)** | 1,960 (49%) | 775 (53%)* | 837 (45%) | 192 (44%) |
| More than 4 chronic conditions | 4,974 (38%) | 777 (45%)** | 1,664 (42%) | 725 (50%)** | 659 (36%) | 185 (43%)** |
| Arthritis | 3,431 (26%) | 497 (29%)* | 1,455 (37%) | 572 (39%) | 606 (33%) | 155 (36%) |
| Asthma | 1,370 (10%) | 183 (11%) | 497 (13%) | 207 (14%) | 177 (10%) | 49 (11%) |
| Atrial fibrillation | 3,659 (28%) | 488 (28%) | 1,555 (29%) | 468 (32%)* | 498 (27%) | 126 (29%) |
| Cancer | 3,749 (29%) | 485 (28%) | 1,261 (32%) | 371 (25%)** | 580 (31%) | 124 (29%) |
| CVD | 2,922 (22%) | 467 (27%)** | 763 (19%) | 392 (27%)** | 373 (20%) | 114 (26%)** |
| Liver disease | 499 (4%) | 50 (3%) | 183 (5%) | 52 (4%) | 72 (4%) | 20 (5%) |
| COPD | 3,641 (28%) | 505 (29%) | 1,083 (27%) | 428 (29%) | 510 (28%) | 132 (31%) |
| Dementia | 1,999 (15%) | 439 (25%)** | 665 (17%) | 390 (27%)** | 223 (12%) | 74 (17%)** |
| Diabetes | 2,985 (23%) | 403 (23%) | 948 (24%) | 350 (24%) | 410 (22%) | 115 (27%)* |
| Epilepsy | 459 (4%) | 75 (4%) | 146 (4%) | 78 (5%)** | 53 (3%) | 10 (2%) |
| CHD | 5,034 (38%) | 733 (42%)** | 1,425 (36%) | 575 (39%)* | 624 (34%) | 141 (33%) |
| Heart failure | 2,197 (17%) | 404 (23%)** | 744 (19%) | 32 (23%)** | 328 (18%) | 109 (25%)** |
| MS | 73 (1%) | 6 (0%) | 21 (1%) | 17 (1%)* | 14 (1%) | 2 (1%) |
| Parkinson's | 293 (2%) | 66 (4%)** | 82 (2%) | 53 (4%)** | 53 (3%) | 20 (5%) |
| Renal failure | 2,501 (19%) | 394 (23%)** | 780 (20%) | 339 (23%)** | 284 (15%) | 110 (25%)** |
| Congenital problems | 277 (2%) | 38 (2%) | 159 (4%) | 51 (4%) | 51 (3%) | 9 (2%) |
| Diseases of blood | 3,784 (29%) | 553 (32%)** | 1,143 (29%) | 426 (29%) | 485 (26%) | 125 (29%) |
| Endocrine metabolic disease | 4,505 (34%) | 624 (36%) | 1,737 (44%) | 652 (45%) | 642 (35%) | 151 (35%) |
| Disease of digestive system | 9,341 (71%) | 1,249 (72%) | 2,710 (68%) | 1,006 (69%) | 1145 (62%) | 286 (66%) |

* p<0.05 ** p<0.01 in chi-square test for categorical and two sample t-test and Mann-Whitney test for continuous variables to test differences between HAH and control; HAH: hospital-at-home; SIMD ranges from 1 (most deprived) to 10 (most affluent); Note: a patient could be registered with more than one ICD-10 codes; SD: standard deviation

For peer review only



For peer review only - http://bmjopen.bmj.com/site/about/guidelines.xhtml

Table 2. Mortality, resource utilisation and costs

| | Site one | | | Site two | | | Site three | | |
|---|---|---|---|---|---|---|---|---|---|
| *Variable* | *Control (n=13139)* | *HAH (n=1737)* | *Mean difference or risk ratio (95%CI)* | *Control (n=3994)* | *HAH (n=1463)* | *Mean difference or risk ratio (95%CI)* | *Control (n=1844)* | *HAH (n=433)* | *Mean difference or risk ratio (95%CI)* |
| Died during index admission | 844 (6%) | 20 (1%) | 0.18 (0.12;0.28)[##] | 256 (6%) | 47 (3%) | 0.50 (0.37;0.68)[##] | 78 (4%) | 2 (1%) | 0.11 (0.03;0.44)[##] |
| Died during follow-up including index admission | 2787 (21%) | 483 (28%) | 1.31 (1.21;1.42)[##] | 867 (22%) | 471 (32%) | 1.48 (1.35;1.63)[##] | 319 (17%) | 116 (27%) | 1.55 (1.29;1.86)[##] |
| Means days alive during follow-up (SD) | 159 (57) | 151 (60) | -8.32 (-11.32;-5.32) | 156 (57) | 146 (66) | -10.10 (-14;-7) | 163 (52) | 151 (60) | -12 (-18;-6) |
| Mean length of index admission in days (SD) | 8.18 (13.13) | 5.54 (5.23) | -2.64 (-2.97;-2.31) | 6.10 (8.74) | 7.35 (5.50) | 1.25 (0.86;1.64) | 6.36 (11.27) | 4.34 (4.19) | -2.02 (-2.66;-1.37) |
| Mean 2 year historical costs (SD) | | | | | | | | | |
| A&E | 173 (260) | 253 (289) | 80 (65;94) | 136 (224) | 180 (238) | 44 (28;60) | 143 (214) | 202 (248) | 59 (31;87) |
| Elective hospital care | 985 (4183) | 956 (5586) | -28 (-352;295) | 1027 (4040) | 705 (3,287) | -321 (-519;-123) | 981 (3733) | 1036 (7738) | 55 (-723;833) |
| Non-elective hospital care | 4037 (9051) | 6945 (11078) | 2908 (2452;3364) | 5101 (11716) | 9593 (15081) | 4,492 (3804;5179) | 3978 (9063) | 7832 (12784) | 3854 (2591;5118) |
| Hospital day case | 707 (2868) | 439 (1318) | -269 (-340;-197) | 625 (4186) | 290 (1676) | -336 (-479;-193) | 544 (2121) | 358 (1139) | -186 (-334;-38) |
| Geriatric long stay | 360 (3078) | 504 (3430) | 143 (-66;354) | 117 (1824) | 252 (2757) | 135 (-13;283) | 105 (1321) | 229 (1221) | 125 (14;235) |
| Mental ward | 247 (3637) | 367 (4865) | 119 (-177;411) | 347 (5019) | 1053 (7839) | 706 (265;1147) | 220 (3231) | 252 (2903) | 32 (-329;393) |
| Outpatient | 173 (204) | 173 (200) | 0 (-11;11) | 222 (244) | 206 (232) | -15 (-30;0) | 212 (270) | 201 (253) | -11 (-38;15) |
| Medication (GP prescriptions) | 1468 (1675) | 1733 (1796) | 256 (187;341) | 1524 (1738) | 1883 (1989) | 360 (253;466) | 1034 (1661) | 1221 (1621) | 188 (30;346) |
| Total | 8149 (12538) | 11369 (14951) | 3219 (2513;3925) | 9098 (239) | 14162 (477) | 5,064 (3984;6143) | 7217 (11478) | 11333 (16071) | 4115 (2467;5764) |
| Mean costs during index admission (SD) | 3195 (4683) | 877[#] (1336) | -2318 (-2420;-2217) | 3426 (4473) | 3273[#] (1217) | -153 (-277;-29) | 2383 (3872) | 1287 (2753) | -1096 (-1398;-793) |
| Mean costs 6 months after index discharge (SD) | | | | | | | | | |
| A&E | 72 (130) | 88 (117) | 17 (11;22) | 55 (124) | 53 (105) | -2 (-9;4) | 59 (101) | 71 (113) | 12 (-1;25) |
| Elective hospital care | 305 (2284) | 157 (1642) | -148 (-236;-60) | 272 (1781) | 204 (1928) | -68 (-190;53) | 169 (1433) | 313 (2440) | 144 (-92;380) |
| Non-elective hospital care | 2444 (5885) | 3961 (7124) | 1517 (1134;1899) | 3942 (8203) | 4471 (9597) | 529 (-77;1135) | 2029 (5281) | 4648 (8767) | 2618 (1779;3458) |
| Hospital day case | 237 (1230) | 73 (440) | -164 (-191;-138) | 234 (1485) | 96 (804) | -139 (-198;-79) | 168 (985) | 63 (320) | -105 (-162;-48) |
| Geriatric long stay | 643 (5191) | 1014 (5467) | 371 (79;663) | 218 (2158) | 150 (1753) | -68 (-178;41) | 320 (2400) | 700 (3873) | 381 (-73;834) |
| Mental ward | 165 (2539) | 206 (2113) | 41 (-58;140) | 299 (3508) | 259 (2928) | -40 (-224;143) | 211 (2803) | 120 (1291) | -91 (-245;64) |
| Outpatient | 54 (108) | 45 (95) | -9 (-13;-5) | 61 (116) | 54 (105) | -8 (-14;-2) | 65 (128) | 67 (131) | 2 (-12;16) |
| Medication (GP prescriptions) | 392 (515) | 415 (540) | 23 (-5;52) | 402 (546) | 482 (627) | 80 (45;115) | 314 (504) | 338 (566) | 24 (-28;76) |
| Hospital-at-home | 4 (56) | 196 (446) | 193 (170;216) | 50 (444) | 642 (1737) | 592 (506;679) | 7 (59) | 90 (257) | 83 (59;108) |
| Total | 4316 (8928) | 6155 (9990) | 1839 (1423;2255) | 5535 (9734) | 6410 (10919) | 875 (156;1595) | 3342 (6990) | 6410 (10614) | 3068 (2178;3958) |
| Mean costs in follow-up (SD) including index admission | 7513 (10510) | 7031 (10110) | -480 (-996;36) | 8961 (11394) | 9683 (11072) | 722 (32;1413) | 5724 (8523) | 7697 (10834) | 1973 (1019;2927) |
| Mean costs per lived day in follow-up (SD) | 83 (150) | 72 (114) | -12 (-17;-6) | 109 (178) | 146 (304) | 37 (18;56) | 55 (96) | 91 (165) | 36 (18;53) |

# it includes the interventions costs (i.e. £628 in site one, £2,928 in site two, and £865.54 in site three) and other costs occurred during the episode; ## Unadjusted Risk Ratio; SD: standard deviation

For peer review only



For peer review only - http://bmjopen.bmj.com/site/about/guidelines.xhtml

*Selection of propensity score matching technique*

In the propensity score matched analysis, there were 1696, 925, and 427 patients in the hospital-at-home cohort and 11571, 3849, and 1683 patients in the hospital cohort in site one, site two, and site three respectively (Figure 2). Local linear regression matching was the best PSM technique to match the cohorts in site one and site three for costs and mortality, as it resulted in a lower mean (i.e. 1.5 and 1.8 respectively) and median (i.e. 1.2 and 1.6 respectively) percentage standardised bias, as well as the lowest Rubin's B (i.e. 9.4 and 9.6 respectively). Based on the same criteria, Kernell matching was selected to match the cohorts in site two. Rubin's R was within the suggested range (i.e. from 0.5 to 2) in the selected techniques. These results as well as the patient characteristics at index admission after propensity score matching are presented in Appendix 2. As this Appendix shows, the differences in patient characteristics between the compared cohorts were almost eliminated after propensity score matching.

*Main propensity score matched analysis*

The results of the main analysis are presented in Panel A in Table 3. After propensity score matching and regression analysis, the healthcare cost for site one in hospital-at-home during the whole follow-up period (i.e. during index admission and over six months after discharge from the index admission) was on average 18% lower (ratio of means: 0.82; 95%CI: 0.76 to 0.89) than admission to hospital. When the cost of the index admission was excluded from the hospital-at-home and hospital cohorts, costs were on average 27% higher (ratio of means: 1.27; 95%CI: 1.14 to 1.41) for hospital-at-home compared with hospital in site one. In site two, the difference in costs between the hospital-at-home and hospital was close to zero (ratio of means: 1.00; 95%CI 0.92 to 1.09) during the whole follow-up period and 9% higher (although not statistically significant) (ratio of means: 1.09; 95%CI: 0.95 to1.24) when the cost of the index admission was excluded. In site three, patients admitted to hospital-at-home had on average 15% higher (although not statistically significant) cost during the whole follow-up period (ratio of means: 1.15; 95%CI 0.99 to 1.33) and 70% higher cost when the cost of the index admission was excluded (ratio of means: 1.70; 95%CI 1.40 to 2.07) compared with patients admitted to hospital. The full results of the regression analyses are presented in Appendix 3.

There may be an increased risk of mortality in all three hospital-at-home cohorts (site one: relative risk 1.09; 95%CI 1.00 to 1.19) (site two: relative risk 1.29; 95%CI: 1.15 to 1.44) (site three: relative risk 1.27; 95%CI: 1.06 to 1.54) compared with the hospital cohort after PSM and regression were performed to adjust for confounding. The Kaplan-Meier survival curves presented in Figure 3 show higher survival rates in the inpatient control cohorts in all three sites, and after weighting with the propensity score the control cohort in site two continued to have a higher survival rate than the hospital-at-home cohort. The difference in survival in site three between the results reported in Table


For peer review only - http://bmjopen.bmj.com/site/about/guidelines.xhtml

3 and the survival curve after weighting is explained by the fact that Kaplan-Meier curves are only weighted with the propensity score without performing an additional regression analysis.

Table 3. Results of the propensity score matched regression analyses

| | | | |
|---|---|---|---|
| **Panel A: main analysis** | | | |
| *Outcome variable* | *Site one (n=13267)* | *Site two (n=4769)* | *Site three (n=2110)* |
| Total costs during follow-up period# | 0.82 (0.03) [0.76;0.89] <0.001 | 1.00 (0.05) [0.92;1.09] 0.982 | 1.15 (0.09) [0.99;1.33] 0.073 |
| Total costs in 6 months after discharge | 1.27 (0.07) [1.14;1.41] <0.001 | 1.09 (0.07) [0.95;1.24] 0.219 | 1.70 (0.17) [1.40;2.07] <0.001 |
| Mortality rate during follow-up | 1.09 (0.05) [1.00;1.19] 0.059 | 1.29 (0.07) [1.15;1.44] <0.0010 | 1.27 (0.12) [1.06;1.54] 0.011 |
| **Panel B: subgroup analysis including only patients with dementia** | | | |
| *Outcome variable* | *Site one (n=2321)* | *Site two (n=1053)* | *Site three (n=280)* |
| Total costs during follow-up period# | 0.76 (0.05) [0.66;0.87] <0.001 | 0.76 (0.06) [0.66;0.88] <0.001 | 0.87 (0.15) [0.63;1.21] 0.409 |
| Total costs in 6 months after discharge | 1.18 (0.11) [0.99;1.41] 0.071 | 0.75 (0.09) [0.59;0.96] 0.021 | 1.58 (0.41) [0.95;2.63] 0.078 |
| Mortality rate during follow-up | 1.05 (0.09) [0.89;1.24] 0.594 | 1.41 (0.12) [1.19;1.67] <0.001 | 1.65 (0.32) [1.12;2.41] 0.011 |
| **Panel C: subgroup analysis including only survivors** | | | |
| *Outcome variable* | *Site one (n=10132)* | *Site two (n=3584)* | *Site three (n=1691)* |
| Total costs during follow-up period# | 0.85 (0.04) [0.77;0.94] 0.002 | 1.11 (0.03) [1.00;1.25] 0.058 | 1.20 (0.11) [1.00;1.43] 0.046 |
| Total costs in 6 months after discharge | 1.23 (0.08) [1.08;1.40] 0.002 | 1.17 (0.10) [0.99;1.38] 0.070 | 1.71 (0.20) [1.36;2.15] <0.001 |
| ***Panel D: sensitivity analysis*** | | | |
| *Outcome variable* | *Site one (n=13267)* | *Site two (n=4769)* | *Site three (n=2110)* |
| Total costs during follow-up period# (assuming 50% lower intervention costs) | 0.77 (0.03) [0.71;0.84] <0.001 | 0.81 (0.04) [0.74;0.9] 0.001 | 1.07 (0.09) [0.91;1.25] 0.399 |
| Total costs during follow-up period# (assuming 50% higher intervention costs) | 0.87 (0.03) [0.81;0.94] 0.001 | 1.18 (0.05) [1.09;1.28] <0.001 | 1.23 (0.09) [1.07;1.42] 0.004 |

# It includes the index admission period and 6 months post-discharge; Note: The results are presented as coefficient (se) [95%CI] p value; The results are after matching and adjusting for age, gender, socio-economic status, primary and secondary ICD-10 codes of index admission, type of long-term condition, mortality (for the analysis of costs), 2-year costs prior to the index admission (by cost category as listed in Box 1).

Figure 3. Survival curves before and after propensity score matching

### *Results of the subgroup analysis*

Patients with dementia (Panel B in Table 3) admitted to hospital-at-home services in site one and site two had an average of 24% lower costs (site one: ratio of means 0.76; 95%CI 0.66 to 0.87; site two: ratio of means 0.76 95%CI: 0.66 to 0.88) from the index admission to six months post-discharge. We found that the population who were admitted to hospital-at-home, and had a diagnosis of dementia, may have an increased risk of death (site one: 1.05, 95% CI 0.89 to 1.24; site two: relative risk 1.41, 95%CI 1.19 to 1.67; site three: relative risk 1.65, 95%CI 1.12 to 2.41) compared with those who had a diagnosis of dementia and who were admitted to hospital.

When we excluded people who died during follow-up (i.e. during index admission and 6 months after discharge), patients admitted to hospital-at-home in site one had lower costs (ratio of means 0.85, 95%CI: 0.77 to 0.94), while there was 11% increase in costs in site two (ratio of means 1.11, 95%CI: 1.00 to 1.25) and 20% increase in site three (ratio of means 1.20, 95%CI: 1.00 to 1.43); the mean costs were higher in the hospital-at-home cohort when the costs during the index admission were excluded (site one: ratio of means 1.23, 95%CI: 1.08 to 1.40; site two: ratio of means 1.17, 95% CI 0.99 to 1.38; site three: ratio of means 1.71, 95% CI 1.36 to 2.15) compared with patients admitted to hospital (Panel C in Table 3).


For peer review only - http://bmjopen.bmj.com/site/about/guidelines.xhtml
For peer review only

### *Results of the sensitivity analyses*

The results from the sensitivity analysis (Panel D in Table 3) showed that patients in the hospital-at-home cohort in site one had 13% lower costs (ratio of means 0.87; 95%CI: 0.81 to 0.94) during the follow-up period (i.e. during index admission and 6 months after index discharge) when the hospital-at-home service costs were assumed to be 50% higher than in the main analysis. In site two, the results from the sensitivity analysis showed that the uncertainty in hospital-at-home service costs lead to increased costs or cost savings by about 18% (ratio of means 1.18; 95%CI: 1.09 to 1.28) during the whole follow-up period. In site three, the sensitivity analysis showed a 23% cost increase (ratio of means 1.23; 95%CI: 1.07 to 1.42), if the intervention costs of hospital-at-home were 50% higher. The estimated E-Values are presented in Appendix 4 and show that unmeasured confounders should be strongly associated with admission to hospital-at-home as well as with costs and mortality after adjusting for the observed confounders in order to explain away the results of the main analysis.

## Discussion

### *Main findings*

Patients who received healthcare from the hospital-at-home services were older, were more socioeconomically disadvantaged, had higher morbidity (measured by the number of long term conditions), higher rates of previous hospitalisation, and there was a greater proportion of women compared with the group admitted to hospital. The two groups also differed in terms of their clinical diagnosis, with the most marked difference across the three services being a greater percentage (five to ten percent difference) of people with dementia. The higher healthcare costs over the two years prior to index admission in those admitted to hospital-at-home were mainly driven by the costs of non-elective hospitalisation. However, the differences in patient characteristics were almost eliminated after propensity score matching. The cost of providing hospital-at-home varied between the three sites from £628 to £2928 per admission, and costs were driven primarily by staff costs. Our findings indicate that hospital-at-home might be associated with an increase in healthcare costs in the six months after index discharge. However, this increase in costs might be offset by likely cost-savings during the index admission. The higher healthcare cost at six months after index discharge, was driven primarily by acute non-elective hospitalisation. Interpreting this is not straightforward; it might indicate a lack of resources during the index admission to hospital-at-home, or an increased risk of hospital admission in the population who receive their healthcare through hospital-at-home. The suggestion of an increased risk of mortality at six months after the index admission might be genuine, or could indicate that propensity score matching did not control for all differences between the groups and thus, the estimates are subject to residual confounding.[15 16]


For peer review only - http://bmjopen.bmj.com/site/about/guidelines.xhtml

*Comparison with previous studies*

A meta-analysis of six small randomised controlled trials concluded that admission avoidance hospital-at-home probably makes little or no difference to the risk of death or transfer to hospital at six months' follow-up, and might increase the likelihood of living at home (albeit with low-certainty evidence); and highlighted the lack of evidence on cost.[2] Studies that have used 'real life data' offer the potential to address criticisms of limited external validity from randomised trials; and propensity score matching is one technique that has been used to balance co-variates when analysing routinely collected health data to assess these type of service delivery interventions. Findings have been consistent, and previous studies have reported higher rates of mortality and unplanned admission for those who received an intermediate care intervention, compared with matched controls.[6 16 17] However, it is possible that these findings are subject to residual confounding.

*Potential mechanisms and interpretation*

Healthcare services that cross the interface of primary and secondary care can bridge and strengthen the integration of acute and community services, and social care. However, by definition this can lead to a complex arrangement of services that reflect availability of local resources,[18] and a willingness to innovate. The hospital-at-home services evaluated in this analysis were established to reduce the demand for acute hospital beds by providing an alternative to admission to hospital, and to lower the risk of functional decline from the limited mobility that older people might experience when in hospital. However, it is possible that the services have several functions, for example by providing both rapid response and reablement, and this is reflected in the diverse population included in this analysis. Existing services and the overall structure of the healthcare care system in Scotland may also have influenced the shape and scope of hospital-at-home functions. Regarding the control cohorts, older people admitted to acute hospital in Scotland receive quite variable care and access to comprehensive geriatric assessment depending on whether they are placed in a geriatric medical unit or other environments such as general adult medicine. This variation may also have influenced the results of this study.

*Implications for clinicians and policy makers*

The variation in intervention costs of the three hospital-at-home services is primarily driven by staff costs, and the findings of the sensitivity analysis confirms that staff costs are likely to determine whether a hospital-at-home service leads to higher costs or cost savings. The skill-mix of healthcare professionals who provide hospital-at-home should be guided by national standards, the type of patients the service targets, and the function of the service in terms of whether or not the service supplements existing community based healthcare, substitutes for hospital level care, augments palliative care services or a combination of these. The integration of these types of service with


For peer review only - http://bmjopen.bmj.com/site/about/guidelines.xhtml

existing primary and secondary care services, for example the provision of out-of-hours care by primary care services, might also determine the costs of these services. Managerial capacity of these services is expected to be of crucial importance in setting-up and managing the team of professionals able to provide high quality care.

The absence of evidence based guidelines about who and under which conditions a patient may be admitted to admission avoidance hospital-at-home might explain the variation in the set-up of services, the difference in patient characteristics between patients admitted to hospital-at-home and hospital, and the relatively small size of the services. This is confirmed by the National Audit of Intermediate Care, [19] that was established in response to concerns about governance structures in intermediate care services, and reported a complex pattern of service provision.

Data on the role and capability of informal care givers is largely absent. In many cases, people admitted to hospital-at-home services receive care from their partners who if old might have health issues themselves.

*Strengths and limitations*

The strengths of this study include the dataset from three of the largest Health Boards in Scotland, the quasi-experimental study design that has allowed inferences from real world evidence, and the sensitivity analyses that helped to address uncertainty in the results. The major limitation of this type of non-randomised comparison is residual confounding. While matching individuals and performing regression analysis can reduce this risk, it is possible that the two populations differed in frailty because we did not match and adjust for differences in the use of community and social services prior to index admission. If unobserved confounders were part of the clinical-decision making by GPs and geriatricians to admit patients to hospital-at-home or hospital, our findings might be biased due to confounding by clinical indication. This type of confounding is often not measured directly because standardised criteria are not available to guide clinical decision-making.[20 21] Therefore, the magnitude of this bias in our results depends on the clinical-decision making process to admit patients to hospital-at-home in the three sites. If clinicians did not consider hospital-at-home as a substitute service to hospitalisation then confounding by indication would increase the residual confounding in our analysis. GPs and geriatricians who refer patients to hospital-at-home are likely to have a clinical bias in preferring to keep older, frailer and terminally ill patients in their own home. Using hospital-at-home admission criteria to define the control cohort accepts that such open criteria will include general medical patients who are likely to have fewer comorbidities, be younger and with a longer life expectancy. However, as the results of the survivors' subgroup analysis were very similar with the results of the main cost analysis we expect that the magnitude of the residual confounding to be small.


For peer review only - http://bmjopen.bmj.com/site/about/guidelines.xhtml

Furthermore, the use of routine data has been used to reliably identify older people with fraility,[22] and approaches using clinical codes to define this population are being tested.[23]

*Future research*

Guidance on the use of real life data to evaluate service delivery interventions is largely absent, and could provide healthcare decision-makers with a relatively inexpensive way of evaluating local service innovations and how to avoid pitfalls in analysis and interpretations. Similar to all observational studies, the findings of this study may be used to identify important questions to be tested in randomised trials.[20] A multi-centre randomised trial that measures outcomes that are key to decision-makers (including informal care giving), and is accompanied by a process evaluation to help explain the findings, is necessary to provide clinicians and policy makers with further evidence about the effectiveness and cost-effectiveness of admission avoidance hospital-at-home services across UK. The authors are involved in such a trial the results of which are expected to be available in 2019.[24]

## Conclusions

We found differences in the populations admitted to hospital-at-home and hospital. The likely higher cost in all three hospital-at-home cohorts, compared with the hospital cohorts during the six months following discharge, highlights the importance of characterising populations eligible to receive these types of healthcare services and of assessing subsequent use of health, social, and informal care following admission to hospital-at-home or hospital. The lack of data on the severity of the observed acute and chronic conditions as well as on type of hospitalised care received in the control cohorts means that we cannot rule out the risk of residual confounding, and the findings should be interpreted with caution.

## Competing interests

All authors have completed the ICMJE uniform disclosure form. GE is leading one of the hospital-at-home services in this study. All other authors declare no support from any organisation for the submitted work; no financial relationships with any organisations that might have an interest in the submitted work in the previous three years; no other relationships or activities that could appear to have influenced the submitted work.

## Details of contributors

AT, GE, and SS were responsible for study concept, GE and SB facilitated the acquisition of data; AT and SS led the writing of the protocol, study design and drafting of the manuscript; AT performed the

For peer review only

statistical analysis. PL and DS provided clinical expertise and commented on previous versions of the manuscript. All authors interpreted the data, critically revised the manuscript for important intellectual content and approved the final version for submission. AT and SS are guarantors.

## Acknowledgement

We would like to thank Charmaine Walker, Jenny Boyd, Alistair Smith and Josh Matthews from ISD Scotland for providing us with the data as well as Christine McGregor (economist in the Scottish Government) for her insightful views and expertise. We are also indebted to Dr Mike Gardner and Prof Alastair Gray (both University of Oxford), Prof Stavros Petrou (University of Warwick), and Dr Matthew Sperrin (University of Manchester) for commenting on previous drafts of the manuscript. Our thanks also to Prof Gillian Parker (University of York), Dr Angela Coulter (University of Oxford) and Prof Stuart Parker (University of Newcastle) for their useful reflection on the study findings. Finally, we would like to thank all healthcare staff in all three sites who made this study happen.

## Ethical approval

We obtained local data transfer agreements and signed release forms from each Health Board's Caldicott guardian. Further approval from an ethics committee was not required because the study was part of a service audit and the data provided to the researchers was de-identified.

## Funding

NIHR, UK. (12/5003//01; "How to Implement Cost-Effective Comprehensive Geriatric Assessment")

## Data sharing agreement

No additional data are available.

## References

1. World Health Organization. Noncommunicable diseases progress monitor 2015. Geneva: World Health Organization, 2015.
2. Shepperd S, Iliffe S, Doll HA, et al. Admission avoidance hospital at home. *Cochrane Database Syst Rev* 2016;9:CD007491. doi: 10.1002/14651858.CD007491.pub2
3. Sherman RE, Anderson SA, Dal Pan GJ, et al. Real-World Evidence - What Is It and What Can It Tell Us? *N Engl J Med* 2016;375(23):2293-97. doi: 10.1056/NEJMsb1609216


For peer review only - http://bmjopen.bmj.com/site/about/guidelines.xhtml

4. Johns B, Baltussen R, Hutubessy R. Programme costs in the economic evaluation of health interventions. *Cost effectiveness and resource allocation : C/E* 2003;1(1):1.
5. Stuart EA. Matching methods for causal inference: A review and a look forward. *Statistical science : a review journal of the Institute of Mathematical Statistics* 2010;25(1):1-21. doi: 10.1214/09-STS313
6. Garrido MM, Kelley AS, Paris J, et al. Methods for constructing and assessing propensity scores. *Health Serv Res* 2014;49(5):1701-20. doi: 10.1111/1475-6773.12182
7. Baser O. Too much ado about propensity score models? Comparing methods of propensity score matching. *Value Health* 2006;9(6):377-85. doi: 10.1111/j.1524-4733.2006.00130.x
8. Craig P, Cooper C, Gunnell D, et al. Using natural experiments to evaluate population health interventions: new Medical Research Council guidance. *J Epidemiol Community Health* 2012;66(12):1182-6. doi: 10.1136/jech-2011-200375
9. Rubin DB. Using propensity scores to help design observational studies: application to the tobacco litigation. *Health Services & Outcomes Research Methodology* 2001;2:169-88.
10. Funk MJ, Westreich D, Wiesen C, et al. Doubly robust estimation of causal effects. *Am J Epidemiol* 2011;173(7):761-7. doi: 10.1093/aje/kwq439
11. Leist AK. Social Inequalities in Dementia Care, Cure, and Research. *J Am Geriatr Soc* 2017;65(5):1100-01. doi: 10.1111/jgs.14893
12. World Health Organization. Draft global action plan on the public health response to dementia, 2016.
13. VanderWeele TJ, Ding P. Sensitivity Analysis in Observational Research: Introducing the E-Value. *Ann Intern Med* 2017;167(4):268-74. doi: 10.7326/M16-2607
14. Liu W, Kuramoto SJ, Stuart EA. An introduction to sensitivity analysis for unobserved confounding in nonexperimental prevention research. *Prev Sci* 2013;14(6):570-80. doi: 10.1007/s11121-012-0339-5
15. Iliffe S. Hospital at home: from red to amber?. Data that will reassure advocates-but without satisfying the sceptics. *Bmj* 1998;316(7147):1761-2.
16. Steventon A, Bardsley M, Billings J, et al. The role of matched controls in building an evidence base for hospital-avoidance schemes: a retrospective evaluation. *Health Serv Res* 2012;47(4):1679-98. doi: 10.1111/j.1475-6773.2011.01367.x
17. Lewis G, Vaithianathan R, Wright L, et al. Integrating care for high-risk patients in England using the virtual ward model: lessons in the process of care integration from three case sites. *Int J Integr Care* 2013;13:e046.
18. Young J, Gladman JR, Forsyth DR, et al. The second national audit of intermediate care. *Age Ageing* 2015;44(2):182-4. doi: 10.1093/ageing/afu174
19. NHS Benchmarking Network. Summary report- England. National audit of intermediate care: NHS Benchmarking Network, 2017.
20. Freemantle N, Marston L, Walters K, et al. Making inferences on treatment effects from real world data: propensity scores, confounding by indication, and other perils for the unwary in observational research. *Bmj* 2013;347:f6409. doi: 10.1136/bmj.f6409
21. Wong AY, Root A, Douglas IJ, et al. Cardiovascular outcomes associated with use of clarithromycin: population based study. *Bmj* 2016;352:h6926. doi: 10.1136/bmj.h6926
22. Clegg A, Bates C, Young J, et al. Development and validation of an electronic frailty index using routine primary care electronic health record data. *Age Ageing* 2017 doi: 10.1093/ageing/afx001
23. Ham C, York N, Sutch S, et al. Hospital bed utilisation in the NHS, Kaiser Permanente, and the US Medicare programme: analysis of routine data. *Bmj* 2003;327(7426):1257. doi: 10.1136/bmj.327.7426.1257
24. Shepperd S, Cradduck-Bamford A, Butler C, et al. A multi-centre randomised trial to compare the effectiveness of geriatrician-led admission avoidance hospital at home versus inpatient admission. *Trials* 2017;18(1):491. doi: 10.1186/s13063-017-2214-y


For peer review only - http://bmjopen.bmj.com/site/about/guidelines.xhtml

For peer review only

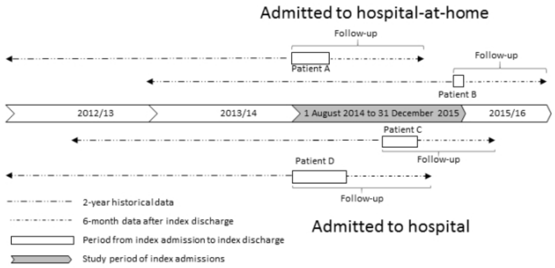


Figure 1. Illustration of obtained data from site one

338x190mm (96 x 96 DPI)

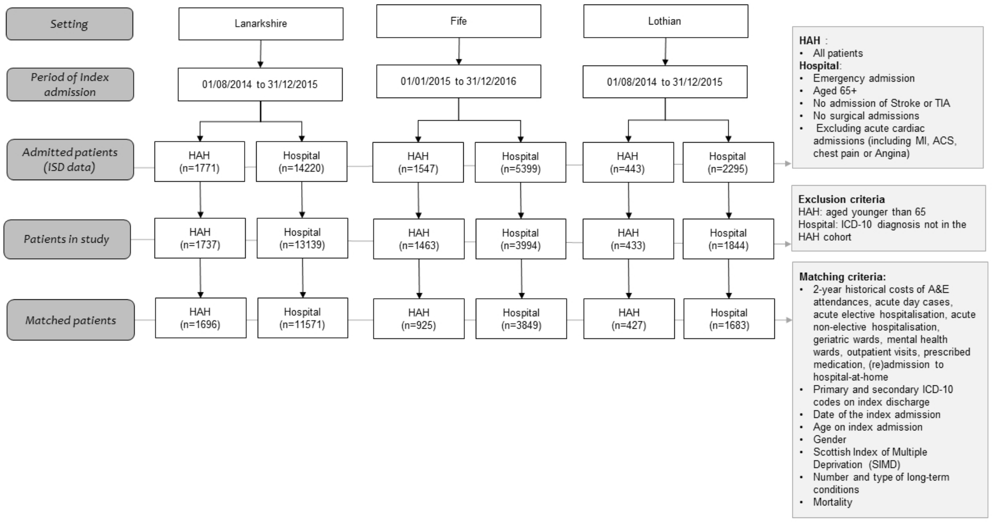


Figure 2 Flowchart of study population

338x190mm (96 x 96 DPI)

Figure 3. Survival curves before and after propensity score matching

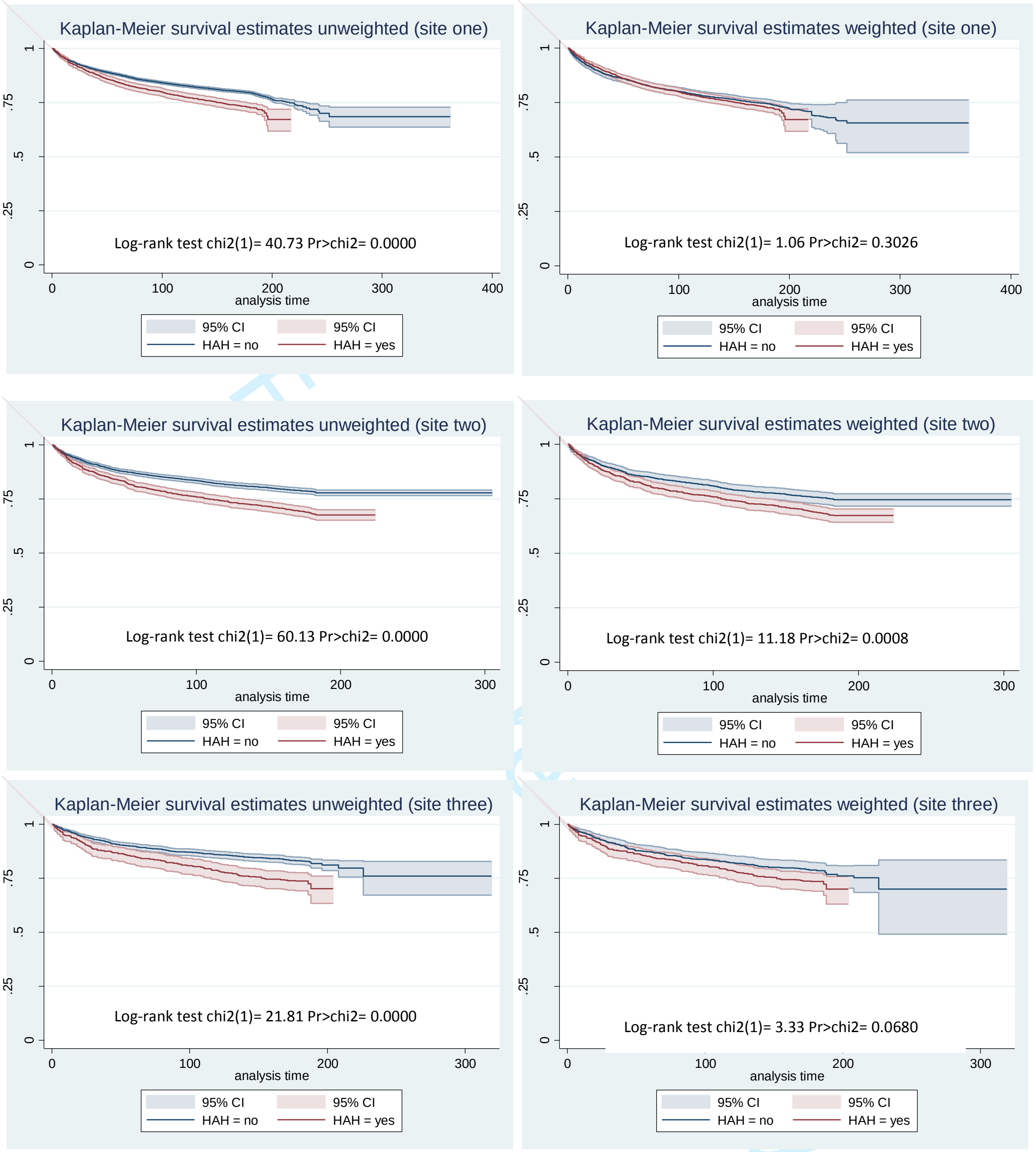


Note: The cohorts in each site were matched on age, gender, socio-economic status, primary and secondary ICD-10 codes of index admission, type of long-term condition, 2-year costs prior to the index admission (by cost category as listed in Box 1); Weighted refers to weighting the observation of each patient based on the propensity score to be in the hospital-at-home cohort as described in the propensity score matching section.

## Appendix 1. Calculation of admission avoidance hospital-at-home in each site

**Site one**

| | | | | | |
|---|---|---|---|---|---|
| | | | **PERIOD** | | |
| **from:** | 01/08/2014 | | **Until:** | 01/01/2016 | **17** |
| | (dd/mm/yyyy) | | (dd/mm/yyyy) | | Months |

| | | | |
|---|---|---|---|
| | | | Source of |
| **Number of HAH admissions (in period)** | 1771 | | ISD IPD data (1/8/14-31/12/15) |
| **Length of HAH stay per episode (in** | 5.53886 | Mean | ISD IPD data (1/8/14-31/12/15) |
| | 0.125605 | Standard error | |
| **HAH bed days (period)** | 9809 | | |

| | | | | | | |
|---|---|---|---|---|---|---|
| **A.1.** | **Staff costs** | | | | | |
| No | Profession | WTEs | Gross | Summary salary | Source of | **Total** |
| a) | Medical staff | | | | | |
| 1 | Consultant | 1.50 | £151,596 | | Business | £227,394 |
| 2 | Agency consultant | 0.16 | £156,926 | | Business | £25,651 |
| 3 | Consultant | 1.07 | £119,710 | | Business | £127,767 |
| b) | Nursing and pharmacy services | | | | | |
| 1 | Band 3 nurse | 3.00 | £24,790 | | Business | £74,369 |
| 2 | Band 6 nurse | 1.49 | £41,425 | | Business | £61,740 |
| 3 | Band 5 Bank nurse | 0.71 | £32,885 | | Business | £23,399 |
| 4 | Band 6 Bank nurse | 0.36 | £38,471 | | Business | £13,687 |
| 5 | Band 7 pharmacist | 0.71 | £55,491 | | Business | £39,484 |
| 6 | Band 5 nurse | 0.16 | £37,036 | | Business | £6,054 |
| 7 | Band 6 nurse | 1.42 | £42,342 | | Business | £60,303 |
| 8 | Band 7 nurse | 1.00 | £42,444 | | Business | £42,444 |
| 9 | Band 8a nurse | 0.71 | £53,126 | | Business | £37,801 |
| c) | Allied health professions | | | | | |
| 1 | Band 6 occupational therapist | 2.59 | £35,489 | | Business | £91,793 |
| 2 | Band 6 physiotherapist | 1.16 | £46,585 | | Business | £54,200 |
| 3 | Band 4 assistant practitioners for rehab | 3.59 | £24,660 | | Business | £88,444 |
| 4 | Band 6 physiotherapy | 0.71 | £46,848 | | Business | £33,334 |
| d) | Administration, ICT and management | | | | | |
| 1 | Band 2 admin/clerical | 0.30 | £19,346 | | Business | £5,804 |
| 2 | Band 3 admin/clerical | 1.00 | £23,948 | | Business | £23,948 |
| 3 | Band 3 admin/clerical | 0.71 | £21,353 | | Business | £15,193 |
| e) | Support services staff | | | | | |
| 1 | | | | | | £0 |
| | **Total** | | | | | £1,052,80 |

| | | | | | | |
|---|---|---|---|---|---|---|
| **A.2.** | **Trainning costs**<br>Note: the time to attend a course should be included in | | | | | |
| No. | Profession | Number of | Cost per | Summary costs | Source of | **Total** |
| 1 | Acute urgent care course | 20 | £250 | | | £5,000 |
| 2 | Prescribing course | 3 | £310 | | | £930 |
| | **Total** | | | | | £5,930 |

| | | | | | | |
|---|---|---|---|---|---|---|
| **A.3.** | **Transport costs** | | | | | |
| No. | Cost item | Number of | Cost per item | Summary costs | Source of | **Total** |
| 1 | Travel and subsistence | | | £37,918 | Business | £37,918 |
| | **Total** | | | | | £37,918 |

| | | | | | | |
|---|---|---|---|---|---|---|
| **A.4.** | **Information and communication costs**<br>**(e.g. brochures and leaflets for patients and their** | | | | | |
| No. | Cost item | Number of | Cost per item | Summary costs | Source of | **Total** |
| 1 | | | | | | £0 |
| | **Total** | | | | | £0 |

| | | | | | | |
|---|---|---|---|---|---|---|
| **A.5.** | **Clinical materials/equipment and** | | | | | |
| No. | Cost item | Number of | Cost per item | Summary costs | Source of | **Total** |
| 1 | Instruments and sundries | | | £2,867 | Business | £2,867 |

| No. | Cost item | Number of | Cost per item | Summary costs | Source of | Total |
|---|---|---|---|---|---|---|
| 2 | Equipment repairs clinical | | | £585 | Business | £585 |
| 3 | Surgical appliances | | | £104 | Business | £104 |
| 4 | Drugs | | | £1,693 | Business | £1,693 |
| 5 | Equipment purchase clinical | | | £298 | Business | £298 |
| | **Total** | | | | | £5,546 |
| **A.6.** | **Support services supplies** | | | | | |
| No. | Cost item | Number of | Cost per item | Summary costs | Source of | **Total** |
| 1 | Catering | | | £177 | Business | £177 |
| 2 | Uniforms | | | £552 | Business | £552 |
| 3 | Printing and stationery | | | £737 | Business | £737 |
| 4 | Dressings | | | £473 | Business | £473 |
| 5 | general services | | | £16 | Business | £16 |
| | **Total** | | | | | £1,955 |
| **A.7.** | **Labs and diagnostics** | | | | | |
| No. | Cost item | Number of | Cost per item | Summary costs | Source of | **Total** |
| 1 | Diagnostic supplies | | | £559 | Business | £559 |
| | | | | | | £559 |
| **A.8.** | **Overhead costs** | | | | | |
| No. | Cost item | Number of | Cost per item | Summary costs | Source of | **Total** |
| 1 | Telephone | | | £3,794 | Business | £3,794 |
| 2 | Building | | | £119 | Business | £119 |
| 3 | Miscellaneous | | | £34 | Business | £34 |
| | **Total** | | | | | £3,947 |
| **A.9.** | **Other costs** | | | | | |
| No. | Cost item | Number of | Cost per item | Summary costs | Source of | **Total** |
| 1 | Equipment purchase non medical | | | £3,354 | Business | £3,354 |
| 2 | postage | | | £772 | Business | £772 |
| | **Total** | | | | | £4,126 |
| **A.10.** | **Additional costs** | | | | | |
| No. | Cost item | Number of | Cost per item | Summary costs | Source of | **Total** |
| 1 | | | | | | £0 |
| | **Total** | | | | | £0 |
| | | | | | **TOTAL** | **£1,112,79** |
| | | | | **Unit cost of HAH admission** | | **£628.34** |
| | | | | **Unit cost of HAH bed day** | | **£113.44** |

For peer review only

**Site two**

| | | | | | | |
|---|---|---|---|---|---|---|
| | | | | **PERIOD** | | |
| | **from:** | 01/01/2015 | | **Until:** | 01/01/2017 | **24** |
| | | (dd/mm/yyyy) | | (dd/mm/yyyy) | | Months |
| | | | | Source of | | |
| **Number of HAH admissions (in period)** | 1547 | | ISD IPD data | | | |
| **Length of HAH stay per episode (in** | 7.35 | Mean | ISD IPD data | | | |
| | 0.14 | Standard error | | | | |
| **HAH bed days (period)** | 11376 | | | | | |

| | | | | | | |
|---|---|---|---|---|---|---|
| **A.1.** | **Staff costs** | | | | | |
| No | Profession | WTEs | Gross | Summary salary | Source of | **Total** |
| a) | Medical staff | | | | | |
| 1 | Senior medical | | | £82,099 | Business | £82,099 |
| 2 | Professional fees and charges | | | £124,391 | Business | £124,391 |
| b) | Nursing and pharmacy services | | | | | |
| 1 | Nursing & Midwifery-trained | | | £2,904,576 | Business | £2,904,57 |
| 2 | Nursing & Midwifery-untrained | | | £627,532 | Business | £627,532 |
| 3 | Pharmacists | | | £43,715 | Business | £43,715 |
| 4 | Pharmacy Technicians | | | £14,471 | Business | £14,471 |
| c) | Allied health professions | | | | | |
| 1 | | | | | Business | £0 |
| d) | Administration, ICT and management | | | | | |
| 1 | Admin Clerical | | | £126,018 | Business | £126,018 |
| e) | Support services staff | | | | | |
| 1 | | | | | | £0 |
| | **Total** | | | | | £3,922,80 |
| **A.2.** | **Trainning costs**<br>Note: the time to attend a course should be included in | | | | | |
| No. | Profession | Number of | Cost per | Summary costs | Source of | **Total** |
| 1 | Trainning costs | | | £1,512 | | £1,512 |
| | **Total** | | | | | £1,512 |
| **A.3.** | **Transport costs** | | | | | |
| No. | Cost item | Number of | Cost per item | Summary costs | Source of | **Total** |
| 1 | Transport | | | £25,711 | Business | £25,711 |
| 2 | Travel And Subsistence | | | £340,388 | | £340,388 |
| | **Total** | | | | | £366,099 |
| **A.4.** | **Information and communication costs**<br>**(e.g. brochures and leaflets for patients and their** | | | | | |
| No. | Cost item | Number of | Cost per item | Summary costs | Source of | **Total** |
| 1 | | | | | | £0 |
| | **Total** | | | | | £0 |
| **A.5.** | **Clinical materials/equipment and** | | | | | |
| No. | Cost item | Number of | Cost per item | Summary costs | Source of | **Total** |
| 1 | Drugs | | | £203,900 | Business | £203,900 |
| 2 | Equipment | | | £14,589 | Business | £14,589 |
| 3 | Paramedical Supplies | | | £3,015 | Business | £3,015 |
| 4 | Surgical Appliances | | | £18 | Business | £18 |
| 5 | Surgical Sundries | | | £80,855 | Business | £80,855 |
| | **Total** | | | | | £302,377 |
| **A.6.** | **Support services supplies** | | | | | |
| No. | Cost item | Number of | Cost per item | Summary costs | Source of | **Total** |
| 1 | Bedding And Linen | | | £112 | Business | £112 |
| 2 | Cleaning | | | £8,251 | Business | £8,251 |
| 3 | General Services | | | £2,595 | | £2,595 |
| | **Total** | | | | | £10,958 |
| **A.7.** | **Labs and diagnostics** | | | | | |
| No. | Cost item | Number of | Cost per item | Summary costs | Source of | **Total** |
| 1 | Cssd/diagnostic Supplies | | | £3,783 | | £3,783 |

| | | | | | | |
|---|---|---|---|---|---|---|
| | | | | | | £3,783 |
| A.8. | Overhead costs | | | | | |
| No. | Cost item | Number of | Cost per item | Summary costs | Source of | Total |
| 1 | Post Carriage And Telephones | | | £5,224 | | £5,224 |
| 2 | Printing And Stationery | | | £5,737 | Business | £5,737 |
| 3 | Property Maintenance | | | £1,174 | | £1,174 |
| 4 | Miscellaneous | | | £25 | Business | £25 |
| | Total | | | | | £12,160 |
| A.9. | Other costs | | | | | |
| No. | Cost item | Number of | Cost per item | Summary costs | Source of | Total |
| 1 | Provisions | | | £6 | Business | £6 |
| 2 | Uniforms | | | £334 | Business | £334 |
| | Total | | | | | £340 |
| A.10. | Additional costs | | | | | |
| No. | Cost item | Number of | Cost per item | Summary costs | Source of | Total |
| 1 | Other Operating Income** | | | -£92,377 | | -£92,377 |
| | Total | | | | | -£92,377 |
| | | | | | TOTAL | £4,527,65 |
| | | | | Unit cost of HAH admission | | £2,926.73 |
| | | | | Unit cost of HAH bed day | | £398.01 |

For peer review only

**Site three**

| | | | | | | |
|---|---|---|---|---|---|---|
| | | | | PERIOD | | |
| | from: | 01/01/2015 | | Until: | 01/01/2016 | 12 |
| | | (dd/mm/yyyy) | | (dd/mm/yyyy) | | Months |
| | | | | Source of | | |
| Number of HAH admissions (in period) | 598 | | | ISD IPD data | | |
| | 598 | | | business case | | |
| Length of HAH stay per episode (in days) | 7.35 | Mean | | ISD IPD data | | |
| | 0.14 | Standard error | | | | |
| HAH bed days (period) | 4397 | | | | | |

| No. | Profession | WTEs | Gross annual | Summary salary cost | Source of | Total |
|---|---|---|---|---|---|---|
| A.1. | Staff costs | | | | | |
| a) | Medical staff | | | | | |
| 1 | Consultant | 1 | | £114,776 | Business | £114,77 |
| 2 | Specialty doctor | 1 | | £79,224 | Business | £79,224 |
| 3 | | | | | Business | £0 |
| 4 | | | | | | £0 |
| 5 | | | | | | £0 |
| b) | Nursing and pharmacy services | | | | | |
| 1 | Nurse (Band 6) | 3 | | £125,484 | Business | £125,48 |
| 2 | Nurse (Band 5) | 1.6 | | £53,256 | Business | £53,256 |
| c) | Allied health professions | | | | | |
| 1 | Occupational therapist | 1 | | £45,156 | Business | £45,156 |
| 2 | Physiotherapist | 1 | | £45,156 | Business | £45,156 |
| d) | Administration, ICT and management | | | | | |
| 1 | Admin Clerical | 1 | | £23,664 | Business | £23,664 |
| e) | Support services staff | | | | | |
| 1 | | | | | | £0 |
| | Total | | | | | £486,71 |

| No. | Profession | Number of | Cost per | Summary costs | Source of | Total |
|---|---|---|---|---|---|---|
| A.2. | Trainning costs<br>Note: the time to attend a course should be included in | | | | | |
| 1 | Trainning costs | | | £1,000 | | £1,000 |
| | Total | | | | | £1,000 |

| No. | Cost item | Number of | Cost per item | Summary costs | Source of | Total |
|---|---|---|---|---|---|---|
| A.3. | Transport costs | | | | | |
| 1 | Transport/travel | | | £20,000 | Business | £20,000 |
| | Total | | | | | £20,000 |

| No. | Cost item | Number of | Cost per item | Summary costs | Source of | Total |
|---|---|---|---|---|---|---|
| A.4. | Information and communication costs<br>(e.g. brochures and leaflets for patients and their family) | | | | | |
| 1 | | | | | | £0 |
| | Total | | | | | £0 |

| No. | Cost item | Number of | Cost per item | Summary costs | Source of | Total |
|---|---|---|---|---|---|---|
| A.5. | Clinical materials/equipment and drugs | | | | | |
| 1 | Drugs | | | £4,840 | Business | £4,840 |
| 2 | Medical supplies | | | £2,393 | Business | £2,393 |
| | Total | | | | | £7,233 |

| No. | Cost item | Number of | Cost per item | Summary costs | Source of | Total |
|---|---|---|---|---|---|---|
| A.6. | Support services supplies | | | | | |
| 1 | | | | | | £0 |
| | Total | | | | | £0 |

| No. | Cost item | Number of | Cost per item | Summary costs | Source of | Total |
|---|---|---|---|---|---|---|
| A.7. | Labs and diagnostics | | | | | |
| 1 | | | | | | £0 |
| | | | | | | £0 |

| No. | Cost item | Number of | Cost per item | Summary costs | Source of | Total |
|---|---|---|---|---|---|---|
| A.8. | Overhead costs | | | | | |

| | | | | | | |
|---|---|---|---|---|---|---|
| 1 | Phones, stationary etc. | | | £1,796 | Business | £1,796 |
| | Total | | | | | £1,796 |
| A.9. | Other costs | | | | | |
| No. | Cost item | Number of | Cost per item | Summary costs | Source of | Total |
| 1 | Mischellaneous | | | £250 | | £250 |
| | Total | | | | | £250 |
| A.10 | Additional costs | | | | | |
| No. | Cost item | Number of | Cost per item | Summary costs | Source of | Total |
| 1 | | | | | | £0 |
| | Total | | | | | £0 |

| | |
|---|---|
| TOTAL | £516,99 |
| Unit cost of HAH admission | £864.54 |
| Unit cost of HAH bed day | £117.57 |

For peer review only

Appendix 2 Results of selecting PSM technique and plots of covariance balance before and after propensity score matching

| | Site one | | Site two | | Site three | |
|---|---|---|---|---|---|---|
| *Variable* | *Costs* mean/median bias;Rubin's B/R | *Survival* mean/median bias;Rubin's B/R | *Costs* mean/median bias;Rubin's B/R | *Survival* mean/median bias;Rubin's B/R | *Costs* mean/median bias;Rubin's B/R | *Survival* mean/median bias;Rubin's B/R |
| Mahalanobis | 7.5/4.2;51.4/1.56 | 7.2/3.7;48.6/1.54 | 7.6/6.7;46.1/1.54 | 7.3/6.7;43.9/1.53 | 6.3/4.7/38.4/1.69 | 6.3/3.5/38.4/1.52 |
| 1-to-1 | 2.9/2.8;14.1/0.90 | 1.9/1.6;12.1/0.84 | 1.4/1.4;9.4/0.97 | 2.2/2.2;14.6/1.14 | 2.7/2.7/14.6/1.02 | 2.3/2.6/14.9/0.73 |
| K-to-1 | 1.9/1.6;11.3/0.76 | 1.9/1.5;12.0/0.81 | 1.8/1.5;11.0/0.83 | 2.4/2.4;13.6/0.76 | 3.6/2.9/16.5/0.99 | 2.8/2.0/16.5/0.94 |
| Kernel | 1.6/1.1;9.8/0.97 | 1.5/1.2;8.9/0.92 | 1.1/0.9;6.9/1.02 | 0.9/0.7;6.5/1.01 | 2.2/1.6/12.3/1.22 | 1.9/1.2/11.2/1.21 |
| Local linear regression | 1.5/1.2;9.4/0.89 | 1.6/1.4;9.4/0.89 | 1.7/1.0;11.0/0.32 | 2.3/1.4;12.8/0.43 | 1.8/1.6/9.6/1.27 | 1.6/1.2/8.5/1.35 |
| Spline | 2.9/2.6;15.7/0.94 | 2.4/2.0;14.9/0.91 | 3.2/2.6;17.5/0.46 | 3.2/2.3;21.0/1.07 | 3.9/3.1/21.6/0.47 | 3.9/2.3/25.7/1.02 |
| IPW | 11.5/5.8;83.2/0.76 | 11.5/5.6;83.1/0.75 | 11.6/8.3;61.3/0.92 | 11.2/7.8;60.2/0.89 | 10.5/8.5/52.2/0.77 | 10.2/8.5/50.9/0.77 |

Rubin's B: the absolute standardized difference of the means of the linear index of the propensity score in the treated and (matched) non-treated group; Rubin's R: the ratio of treated to (matched) non-treated variances of the propensity score index; Samples sufficiently balanced if B less than 25 and that R between 0.5 and 2.

Standardised percentage bias before and after local linear regression propensity score matching for costs in site one

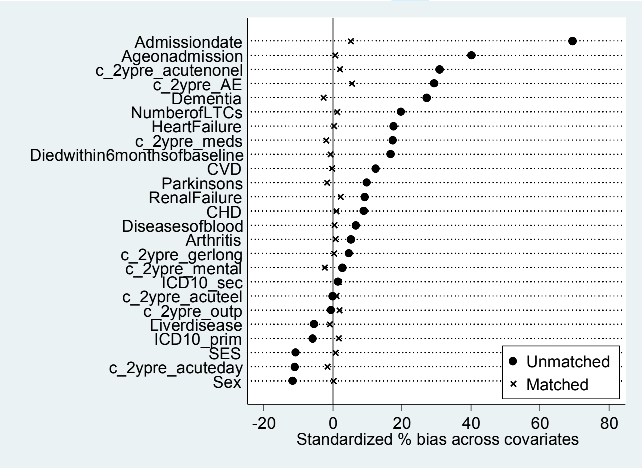


Standardised percentage bias before and after local linear regression propensity score matching for survival in site one

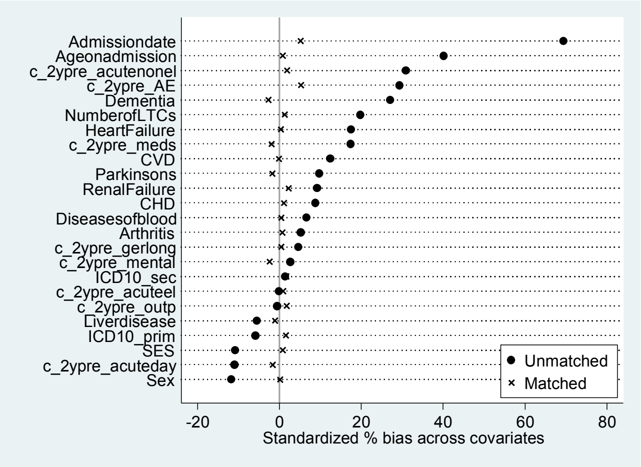


For peer review only

Standardised percentage bias before and after Kernel propensity score matching for costs in site two

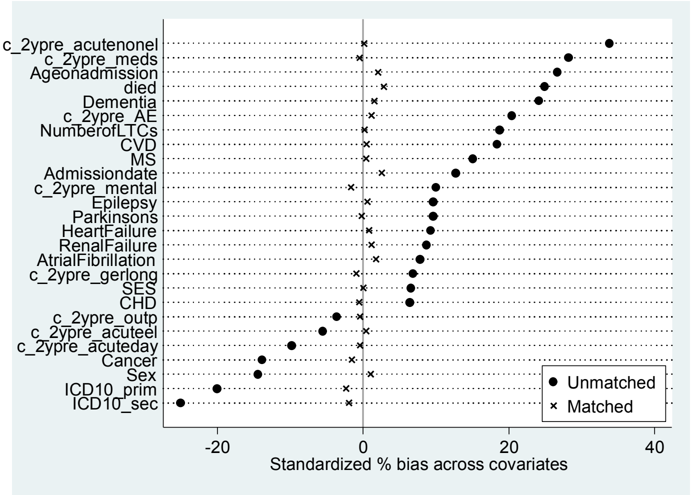


Standardised percentage bias before and after Kernel propensity score matching for survival in site two

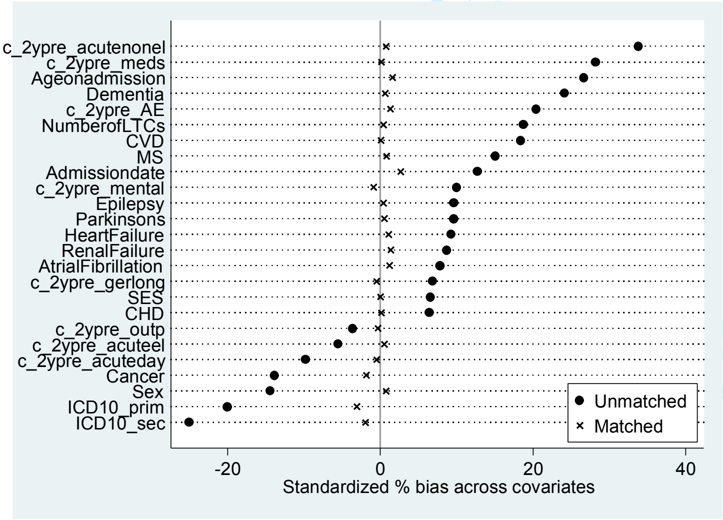


For peer review only

Standardised percentage bias before and after local linear regression propensity score matching for costs in site three

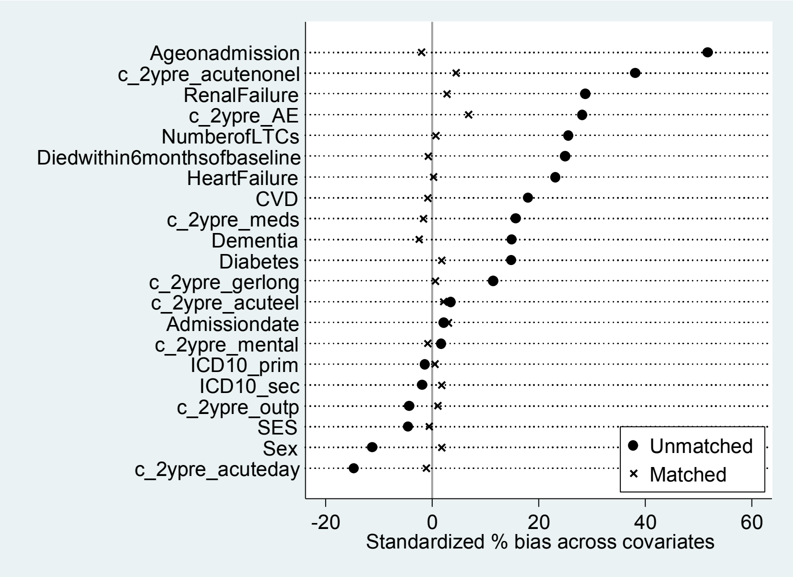


Standardised percentage bias before and after local linear regression propensity score matching for survival in site three

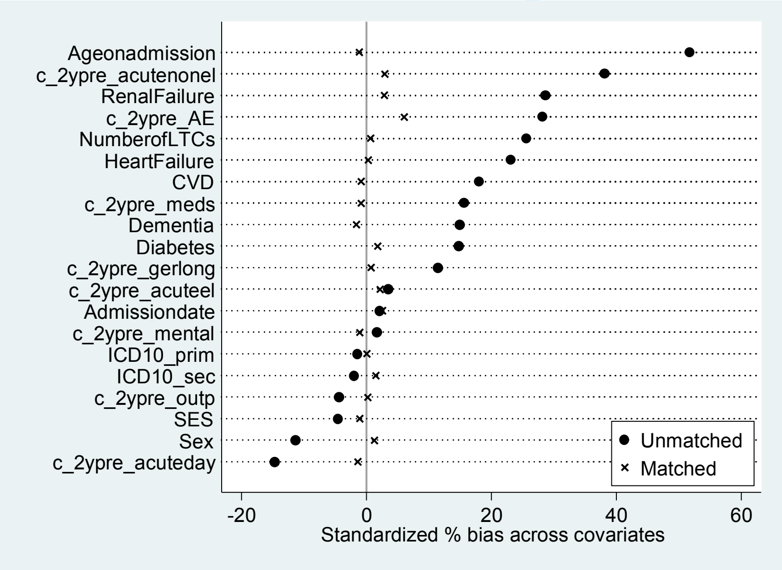

Propensity score distributions by cohort in each site

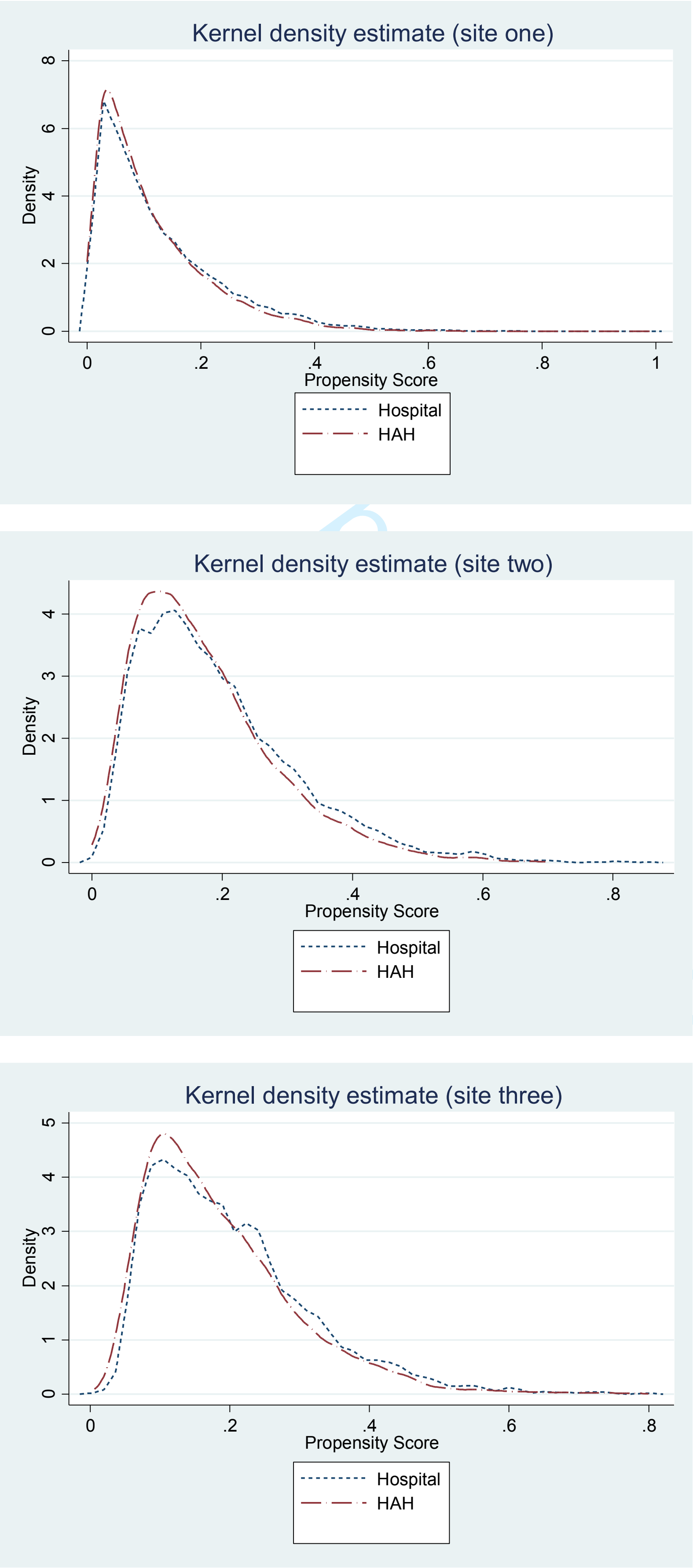

Patient characteristics at index admission after propensity score matching

| | Site one | | Site two | | Site three | |
|---|---|---|---|---|---|---|
| *Variable* | Control | HAH | Control | HAH | Control | HAH |
| Mean age on admission (sd) | 81.2 (7.95) | 81.2 (7.20) | 82.2 (8.03) | 82.4 (7.68) | 81.6 (7.96) | 81.4 (7.10) |
| Female | 63% | 63% | 62% | 62% | 62% | 61% |
| Higher than 4 on the SIMD | 35% | 35% | 53% | 52% | 44% | 44% |
| More than 4 chronic conditions | 44% | 45% | 48% | 50% | 43% | 43% |
| Arthritis | 29% | 29% | 38% | 38% | 33% | 36% |
| Asthma | 10% | 11% | 13% | 14% | 9% | 11% |
| Atrial fibrillation | 29% | 28% | 32% | 33% | 30% | 29% |
| Cancer | 28% | 28% | 28% | 27% | 30% | 28% |
| CVD | 27% | 27% | 26% | 26% | 27% | 26% |
| Liver disease | 3% | 3% | 4% | 4% | 4% | 5% |
| COPD | 27% | 29% | 26% | 28% | 26% | 30% |
| Dementia | 26% | 25% | 26% | 26% | 18% | 17% |
| Diabetes | 23% | 23% | 23% | 24% | 26% | 26% |
| Epilepsy | 4% | 4% | 5% | 5% | 2% | 2% |
| CHD | 42% | 42% | 40% | 40% | 37% | 32% |
| Heart failure | 23% | 23% | 22% | 23% | 25% | 25% |
| MS | 0% | 0% | 1% | 1% | 1% | 1% |
| Parkinson's | 4% | 4% | 3% | 3% | 3% | 5% |
| Renal failure | 22% | 23% | 24% | 24% | 25% | 25% |
| Congenital problems | 2% | 2% | 4% | 4% | 2% | 2% |
| Diseases of blood | 32% | 32% | 30% | 27% | 29% | 29% |
| Endocrine metabolic disease | 36% | 36% | 46% | 45% | 39% | 35% |
| Disease of digestive system | 70% | 72% | 70% | 70% | 64% | 66% |

HAH: hospital-at-home; SIMD ranges from 1 (most deprived) to 10 (most affluent); Note: a patient could be registered with more than one ICD-10 codes

For peer review only

Appendix 3. Full results of the regression analyses

Association of hospital at home with total costs (after propensity score matching)

| | site one (n=13,267) | | site two (n=4,769) | | site three (n=2110) | |
|---|---|---|---|---|---|---|
| | Follow-up period coefficient (se) [95%CI] p value | 6 months after discharge coefficient (se) [95%CI] p value | Follow-up period coefficient (se) [95%CI] p value | 6 months after discharge coefficient (se) [95%CI] p value | Follow-up period coefficient (se) [95%CI] p value | 6 months after discharge coefficient (se) [95%CI] p value |
| HAH | 0.82 (0.03) [0.76;0.89] <0.001 | 1.27 (0.07) [1.14;1.41] <0.001 | 1.00 (0.05) [0.92;1.09] 0.982 | 1.09 (0.07) [0.95;1.24] 0.219 | 1.15 (0.09) [0.99;1.33] 0.073 | 1.70 (0.17) [1.4;2.07] <0.001 |
| Admission date | 1.00 (0.00) [1.00;1.00] 0.058 | 1.00 (0.00) [1.00;1.00] 0.009 | 1.00 (0.00) [1.00;1.00] 0.386 | 1.00 (0.00) [1.00;1.00] 0.824 | 1.00 (0.00) [1.00;1.00] 0.009 | 1.00 (0.00) [1.00;1.00] 0.056 |
| ICD10 primary | 1.00 (0.00) [1.00;1.00] 0.660 | 1.00 (0.00) [1.00;1.00] 0.230 | 1.00 (0.00) [1.00;1.00] 0.001 | 1.00 (0.00) [1.00;1.00] <0.001 | 1.00 (0.00) [1.00;1.00] 0.162 | 1.00 (0.00) [1.00;1.00] 0.101 |
| ICD10 secondary | 1.00 (0.00) [1.00;1.00] 0.641 | 1.00 (0.00) [1.00;1.00] 0.988 | 1.00 (0.00) [1.00;1.00] 0.146 | 1.00 (0.00) [1.00;1.00] 0.238 | 1.00 (0.00) [1.00;1.00] 0.897 | 1.00 (0.00) [1.00;1.00] 0.971 |
| 2yrs pre AE costs | 1.00 (0.00) [1.00;1.00] 0.240 | 1.00 (0.00) [1.00;1.00] 0.018 | 1.00 (0.00) [1.00;1.00] 0.624 | 1.00 (0.00) [1.00;1.00] 0.309 | 1.00 (0.00) [1.00;1.00] 0.284 | 1.00 (0.00) [1.00;1.00] 0.42 |
| 2yrs pre elective costs | 1.00 (0.00) [1.00;1.00] 0.906 | 1.00 (0.00) [1.00;1.00] 0.919 | 1.00 (0.00) [1.00;1.00] 0.588 | 1.00 (0.00) [1.00;1.00] 0.435 | 1.00 (0.00) [1.00;1.00] 0.865 | 1.00 (0.00) [1.00;1.00] 0.931 |
| 2yrs pre non-elective costs | 1.00 (0.00) [1.00;1.00] <0.001 | 1.00 (0.00) [1.00;1.00] 0.001 | 1.00 (0.00) [1.00;1.00] 0.694 | 1.00 (0.00) [1.00;1.00] 0.697 | 1.00 (0.00) [1.00;1.00] 0.018 | 1.00 (0.00) [1.00;1.00] 0.015 |
| 2yrs pre day case costs | 1.00 (0.00) [1.00;1.00] 0.098 | 1.00 (0.00) [1.00;1.00] 0.020 | 1.00 (0.00) [1.00;1.00] 0.005 | 1.00 (0.00) [1.00;1.00] <0.001 | 1.00 (0.00) [1.00;1.00] 0.14 | 1.00 (0.00) [1.00;1.00] 0.100 |
| 2yrs pre geriatric ward costs | 1.00 (0.00) [1.00;1.00] 0.005 | 1.00 (0.00) [1.00;1.00] 0.054 | 1.00 (0.00) [1.00;1.00] 0.001 | 1.00 (0.00) [1.00;1.00] 0.003 | 1.00 (0.00) [1.00;1.00] 0.634 | 1.00 (0.00) [1.00;1.00] 0.342 |
| 2yrs pre mental ward costs | 1.00 (0.00) [1.00;1.00] 0.880 | 1.00 (0.00) [1.00;1.00] 0.911 | 1.00 (0.00) [1.00;1.00] 0.009 | 1.00 (0.00) [1.00;1.00] 0.014 | 1.00 (0.00) [1.00;1.00] 0.111 | 1.00 (0.00) [1.00;1.00] 0.382 |
| 2yrs pre outpatient costs | 1.00 (0.00) [1.00;1.00] 0.087 | 1.00 (0.00) [1.00;1.00] 0.056 | 1.00 (0.00) [1.00;1.00] 0.026 | 1.00 (0.00) [1.00;1.00] 0.043 | 1.00 (0.00) [1.00;1.00] 0.683 | 1.00 (0.00) [1.00;1.00] 0.656 |
| 2yrs pre medication costs | 1.00 (0.00) [1.00;1.00] 0.798 | 1.00 (0.00) [1.00;1.00] 0.750 | 1.00 (0.00) [1.00;1.00] 0.172 | 1.00 (0.00) [1.00;1.00] 0.369 | 1.00 (0.00) [1.00;1.00] 0.687 | 1.00 (0.00) [1.00;1.00] 0.935 |
| Died during follow-up | 1.03 (0.04) [0.95;1.11] 0.530 | 0.91 (0.05) [0.82;1.01] 0.089 | 1.05 (0.05) [0.96;1.15] 0.302 | 0.90 (0.06) [0.78;1.05] 0.143 | 1.06 (0.09) [0.90;1.24] 0.498 | 0.97 (0.11) [0.78;1.21] 0.784 |
| Number of LTCs | 1.09 (0.02) [1.05;1.12] <0.001 | 1.12 (0.02) [1.07;1.16] <0.001 | 1.04 (0.02) [1.00;1.07] 0.054 | 1.06 (0.03) [1.00;1.11] 0.035 | 1.06 (0.03) [1.01;1.11] 0.017 | 1.10 (0.03) [1.03;1.17] 0.003 |
| Age on admission | 1.00 (0.00) [0.99;1.01] 0.383 | 1.00 (0.00) [0.99;1.01] 0.981 | 1.00 (0.00) [0.99;1.01] 0.984 | 1.00 (0.00) [1.00;1.00] 0.349 | 1.01 (0.01) [1.00;1.02] 0.045 | 1.01 (0.01) [0.99;1.02] 0.41 |
| Male | 1.09 (0.05) [1.01;1.19] 0.034 | 1.08 (0.06) [0.97;1.19] 0.136 | 0.95 (0.05) [0.86;1.05] 0.340 | 0.99 (0.08) [0.85;1.15] 0.859 | 0.97 (0.08) [0.83;1.13] 0.709 | 0.98 (0.10) [0.81;1.2] 0.875 |
| SES | 1.00 (0.01) [0.98;1.02] 0.988 | 1.00 (0.01) [0.98;1.03] 0.741 | 1.01 (0.01) [1.00;1.03] 0.182 | 1.03 (0.01) [1.00;1.05] 0.033 | 1.00 (0.02) [0.97;1.03] 0.899 | 1.01 (0.02) [0.97;1.05] 0.779 |
| Arthritis | 0.96 (0.04) [0.88;1.05] 0.398 | 0.95 (0.05) [0.85;1.06] 0.346 | | | ------------ | ------------ |
| Atrial Fibrillation | ------------ | ------------ | 1.09 (0.06) [0.98;1.2] 0.098 | 1.13 (0.08) [0.97;1.30] 0.113 | ------------ | ------------ |
| Cancer | ------------ | ------------ | 1.04 (0.05) [0.94;1.15] 0.485 | 1.07 (0.08) [0.92;1.24] 0.403 | ------------ | ------------ |
| CVD | 1.01 (0.06) [0.91;1.13] 0.767 | 0.99 (0.07) [0.86;1.13] 0.903 | 1.08 (0.06) [0.97;1.2] 0.168 | 1.11 (0.09) [0.95;1.29] 0.199 | 1.10 (0.11) [0.90;1.34] 0.339 | 1.07 (0.13) [0.84;1.37] 0.585 |
| Liver disease | 1.21 (0.13) [0.98;1.50] 0.074 | 1.20 (0.14) [0.95;1.51] 0.130 | ------------ | ------------ | ------------ | ------------ |
| Dementia | 1.06 (0.05) [0.97;1.17] 0.179 | 1.07 (0.07) [0.95;1.21] 0.236 | 1.00 (0.05) [0.91;1.11] 0.942 | 1.03 (0.08) [0.89;1.19] 0.683 | 1.14 (0.11) [0.95;1.38] 0.166 | 1.17 (0.15) [0.91;1.5] 0.211 |
| Epilepsy | ------------ | ------------ | 1.04 (0.11) [0.85;1.27] 0.734 | 1.04 (0.15) [0.78;1.38] 0.803 | ------------ | ------------ |
| CHD | 0.85 (0.05) [0.77;0.95] 0.004 | 0.83 (0.06) [0.73;0.95] 0.008 | 1.01 (0.06) [0.9;1.13] 0.871 | 1.02 (0.08) [0.88;1.20] 0.766 | ------------ | ------------ |
| Heart Failure | 1.09 (0.06) [0.98;1.20] 0.102 | 1.10 (0.07) [0.97;1.24] 0.154 | 1.08 (0.06) [0.96;1.21] 0.186 | 1.08 (0.09) [0.92;1.28] 0.363 | 1.01 (0.10) [0.83;1.23] 0.919 | 0.98 (0.13) [0.76;1.26] 0.879 |
| Multiple sclerosis | ------------ | ------------ | 0.74 (0.10) [0.57;0.98] 0.033 | 0.59 (0.15) [0.36;0.97] 0.035 | ------------ | ------------ |
| Parkinson's | 1.24 (0.11) [1.03;1.48] 0.019 | 1.20 (0.14) [0.95;1.51] 0.120 | 1.09 (0.15) [0.83;1.42] 0.554 | 1.09 (0.20) [0.75;1.57] 0.664 | ------------ | ------------ |
| Renal Failure | 1.03 (0.05) [0.94;1.13] 0.513 | 1.06 (0.06) [0.94;1.19] 0.362 | 1.05 (0.06) [0.94;1.17] 0.420 | 1.08 (0.09) [0.92;1.26] 0.348 | 1.12 (0.12) [0.9;1.38] 0.306 | 1.14 (0.16) [0.87;1.49] 0.346 |
| Diseases of blood | 1.05 (0.05) [0.96;1.15] 0.275 | 1.05 (0.06) [0.94;1.18] 0.363 | ------------ | ------------ | ------------ | ------------ |
| Diabetes | ------------ | ------------ | ------------ | ------------ | 1.21 (0.11) [1.01;1.45] 0.043 | 1.24 (0.14) [0.99;1.55] 0.061 |
| Constant | 15.93 (46.90) [0.05;5098.92] 0.347 | 0.19 (0.68) [0.00;224.04] 0.644 | 285486.5 (1267507) [47.47; 1.72E+09] 0.005 | 899.53 (5743.23) [0.00;0.00] 0.287 | 20700000000000 (186000000000000) [500612.1;8.6E+20] 0.001 | 2230000000000 (25100000000000) [559.85;8.85E+21] 0.012 |

# driven mainly by non-elective hospital care; Note the HAH unit costs in site one were £628.34 per admission to HAH and have been added to the costs during the episode.

For peer review only

Association of hospital-at-home with mortality risk during study period (after propensity score matching)

| | site one (n=13,267) | site two (n=4,771) | site three (n=2110) |
|---|---|---|---|
| | coefficient (se) [95%CI] p value | coefficient (se) [95%CI] p value | coefficient (se) [95%CI] p value |
| HAH | 1.09 (0.05) [1.00;1.19] 0.059 | 1.29 (0.07) [1.15;1.44] <0.0010 | 1.27 (0.12) [1.06;1.54] 0.011 |
| Admission date | 1.00 (0.00) [1.00;1.00] 0.842 | 1.00 (0.00) [1.00;1.00] 0.100 | 1 (0) [1;1] 0.687 |
| ICD10 primary | 1.00 (0.00) [1.00;1.00] <0.001 | 1.00 (0.00) [1.00;1.00] 0.001 | 1 (0) [1;1] 0.006 |
| ICD10 secondary | 1.00 (0.00) [1.00;1.00] <0.001 | 1.00 (0.00) [1.00;1.00] 0.023 | 1 (0) [1;1] 0.359 |
| 2yrs pre AE costs | 1.00 (0.00) [1.00;1.00] 0.640 | 1.00 (0.00) [1.00;1.00] 0.153 | 1 (0) [1;1] 0.027 |
| 2yrs pre elective costs | 1.00 (0.00) [1.00;1.00] 0.487 | 1.00 (0.00) [1.00;1.00] 0.462 | 1 (0) [1;1] 0.079 |
| 2yrs pre non-elective costs | 1.00 (0.00) [1.00;1.00] 0.001 | 1.00 (0.00) [1.00;1.00] 0.007 | 1 (0) [1;1] 0.052 |
| 2yrs pre day case costs | 1.00 (0.00) [1.00;1.00] <0.001 | 1.00 (0.00) [1.00;1.00] 0.001 | 1 (0) [1;1] 0.903 |
| 2yrs pre geriatric ward costs | 1.00 (0.00) [1.00;1.00] 0.022 | 1.00 (0.00) [1.00;1.00] <0.001 | 1 (0) [1;1] 0.338 |
| 2yrs pre mental ward costs | 1.00 (0.00) [1.00;1.00] 0.419 | 1.00 (0.00) [1.00;1.00] 0.943 | 1 (0) [1;1] 0 |
| 2yrs pre outpatient costs | 1.00 (0.00) [1.00;1.00] 0.091 | 1.00 (0.00) [1.00;1.00] 0.882 | 1 (0) [1;1] 0.001 |
| 2yrs pre medication costs | 1.00 (0.00) [1.00;1.00] 0.044 | 1.00 (0.00) [1.00;1.00] 0.037 | 1 (0) [1;1] 0 |
| Number of LTCs | 1.03 (0.02) [0.99;1.07] 0.120 | 0.96 (0.02) [0.92;1.01] 0.107 | 1.07 (0.04) [1;1.14] 0.048 |
| Age on admission | 1.04 (0) [1.03;1.04] <0.001 | 1.03 (0.00) [1.02;1.04] <0.001 | 1.04 (0.01) [1.02;1.05] 0 |
| Male | 1.12 (0.05) [1.01;1.22] 0.017 | 1.23 (0.08) [1.09;1.39] 0.001 | 1.37 (0.14) [1.12;1.67] 0.002 |
| SES | 0.97 (0.01) [0.95;0.99] 0.001 | 0.98 (0.01) [0.96;1.00] 0.088 | 1.01 (0.02) [0.98;1.05] 0.483 |
| Arthritis | 0.86 (0.05) [0.77;0.97] 0.008 | ------------ | ------------ |
| Atrial Fibrillation | ------------ | 1.11 (0.08) [0.97;1.28] 0.133 | ------------ |
| Cancer | ------------ | 1.86 (0.12) [1.64;2.11] <0.001 | ------------ |
| CVD | 0.94 (0.06) [0.83;1.05] 0.276 | 1.06 (0.08) [0.92;1.22] 0.438 | 0.95 (0.12) [0.74;1.21] 0.673 |
| Liver disease | 1.33 (0.16) [1.04;1.67] 0.015 | ------------ | ------------ |
| Dementia | 1.11 (0.06) [1.00;1.25] 0.058 | 1.59 (0.11) [1.39;1.82] <0.001 | 1.31 (0.16) [1.03;1.67] 0.025 |
| Epilepsy | | 1.19 (0.17) [0.91;1.57] 0.207 | ------------ |
| CHD | 0.91 (0.05) [0.82;1.03] 0.114 | 0.93 (0.07) [0.80;1.08] 0.345 | ------------ |
| Heart Failure | 1.13 (0.07) [1.00;1.28] 0.052 | 1.35 (0.11) [1.15;1.57] <0.001 | 1.16 (0.15) [0.9;1.5] 0.256 |
| Multiple sclerosis | ------------ | 1.54 (0.39) [0.94;2.52] 0.086 | ------------ |
| Parkinson's | 1.11 (0.13) [0.86;1.39] 0.374 | 0.93 (0.17) [0.65;1.33] 0.678 | ------------ |
| Renal Failure | 1.07 (0.07) [0.95;1.21] 0.292 | 1.35 (0.10) [1.16;1.56] <0.001 | 0.93 (0.12) [0.72;1.2] 0.571 |
| Diseases of blood | 0.93 (0.05) [0.85;1.06] 0.201 | ------------ | ------------ |
| Diabetes | ------------ | ------------ | 0.74 (0.1) [0.57;0.97] 0.026 |
| Constant | 0.01 (0.04) [0.00;7.06] 0.174 | 0.00 (0.00) [0.00;0.18] 0.025 | 0 (0) [0;319640.8] 0.405 |

For peer review only

Results of the subgroup analysis including only patients with dementia (costs)

| | site one (n=2,321) | | site two (n=1,053) | | site three (n=280) | |
|---|---|---|---|---|---|---|
| | Follow-up period<br>coefficient (se) [95%CI] p value | Total costs in 6 months after discharge<br>coefficient (se) [95%CI] p value | Follow-up period<br>coefficient (se) [95%CI] p value | Total costs in 6 months after discharge<br>coefficient (se) [95%CI] p value | Follow-up period<br>coefficient (se) [95%CI] p value | Total costs in 6 months after discharge<br>coefficient (se) [95%CI] p value |
| HAH (hospital) | 0.76 (0.05) [0.66;0.87] 0 | 1.18 (0.11) [0.99;1.41] 0.071 | 0.76 (0.06) [0.66;0.88] 0 | 0.75 (0.09) [0.59;0.96] 0.021 | 0.87 (0.15) [0.63;1.21] 0.409 | 1.58 (0.41) [0.95;2.63] 0.078 |
| Admission date | 1 (0) [1;1] 0.528 | 1.00 (0.00) [1.00;1.00] 0.329 | 1 (0) [1;1] 0.513 | 1.00 (0.00) [1.00;1.00] 0.532 | 1 (0) [1;1] 0.002 | 1 (0) [0.99;1] 0.003 |
| ICD10 primary | 1 (0) [1;1] 0.025 | 1.00 (0.00) [1.00;1.00] 0.003 | 1 (0) [1;1] 0.079 | 1.00 (0.00) [1.00;1.00] 0.008 | 1 (0) [1;1] 0.666 | 1 (0) [1;1] 0.123 |
| ICD10 secondary | 1 (0) [1;1] 0.027 | 1.00 (0.00) [1.00;1.00] 0.086 | ------------ | ------------ | 1 (0) [1;1] 0.946 | 1 (0) [1;1] 0.594 |
| 2yrs pre AE costs | 1 (0) [1;1] 0.063 | 1.00 (0.00) [1.00;1.00] 0.021 | 1 (0) [1;1] 0.979 | 1.00 (0.00) [1.00;1.00] 0.93 | 1 (0) [1;1] 0.57 | 1 (0) [1;1] 0.331 |
| 2yrs pre elective costs | 1 (0) [1;1] 0.913 | 1.00 (0.00) [1.00;1.00] 0.708 | 1 (0) [1;1] 0.979 | 1.00 (0.00) [1.00;1.00] 0.889 | 1 (0) [1;1] 0.115 | 1 (0) [1;1] 0.208 |
| 2yrs pre non-elective costs | 1 (0) [1;1] 0.564 | 1.00 (0.00) [1.00;1.00] 0.605 | 1 (0) [1;1] 0.031 | 1.00 (0.00) [1.00;1.00] 0.008 | 1 (0) [1;1] 0.888 | 1 (0) [1;1] 0.639 |
| 2yrs pre day case costs | 1 (0) [1;1] 0.455 | 1.00 (0.00) [1.00;1.00] 0.632 | 1 (0) [1;1] 0.725 | 1.00 (0.00) [1.00;1.00] 0.307 | 1 (0) [1;1] 0.1 | 1 (0) [1;1] 0.279 |
| 2yrs pre geriatric ward costs | 1 (0) [1;1] 0.233 | 1.00 (0.00) [1.00;1.00] 0.566 | 1 (0) [1;1] 0.012 | 1.00 (0.00) [1.00;1.00] 0.003 | 1 (0) [1;1] 0.907 | 1 (0) [1;1] 0.952 |
| 2yrs pre mental ward costs | 1 (0) [1;1] 0.343 | 1.00 (0.00) [1.00;1.00] 0.335 | 1 (0) [1;1] 0.084 | 1.00 (0.00) [1.00;1.00] 0.042 | 1 (0) [1;1] 0.01 | 1 (0) [1;1] 0.021 |
| 2yrs pre outpatient costs | 1 (0) [1;1] 0.066 | 1.00 (0.00) [1.00;1.00] 0.082 | 1 (0) [1;1] 0.001 | 1.00 (0.00) [1.00;1.00] 0.001 | 1 (0) [1;1] 0.685 | 1 (0) [1;1] 0.403 |
| 2yrs pre medication costs | 1 (0) [1;1] 0.306 | 1.00 (0.00) [1.00;1.00] 0.316 | 1 (0) [1;1] 0.13 | 1.00 (0.00) [1.00;1.00] 0.265 | 1 (0) [1;1] 0.042 | 1 (0) [1;1] 0.044 |
| Died within 6months | 0.81 (0.06) [0.7;0.94] 0.005 | 0.70 (0.07) [0.58;0.85] <0.001 | 0.89 (0.07) [0.76;1.03] 0.118 | 0.73 (0.09) [0.58;0.93] 0.011 | 0.66 (0.13) [0.45;0.96] 0.031 | 0.44 (0.13) [0.25;0.77] 0.004 |
| Number of LTCs | 1.06 (0.03) [1;1.12] 0.069 | 1.07 (0.04) [1.00;1.16] 0.063 | 1.08 (0.03) [1.02;1.14] 0.006 | 1.15 (0.05) [1.05;1.26] 0.003 | 1.04 (0.06) [0.94;1.16] 0.443 | 1.01 (0.08) [0.86;1.18] 0.935 |
| Age on admission | 0.99 (0.01) [0.98;1] 0.094 | 0.98 (0.01) [0.97;1.00] 0.015 | 0.98 (0.01) [0.97;1] 0.007 | 0.97 (0.01) [0.95;0.99] 0.003 | 1 (0.01) [0.98;1.03] 0.933 | 1 (0.02) [0.97;1.03] 0.946 |
| Male | 1.13 (0.08) [0.99;1.31] 0.076 | 1.14 (0.11) [0.95;1.37] 0.151 | 0.95 (0.07) [0.82;1.11] 0.511 | 0.95 (0.12) [0.74;1.22] 0.679 | 1.05 (0.17) [0.76;1.43] 0.78 | 1.07 (0.26) [0.67;1.71] 0.774 |
| SES | 1.01 (0.01) [0.98;1.04] 0.693 | 1.01 (0.02) [0.97;1.04] 0.77 | 1.03 (0.01) [1;1.05] 0.053 | 1.06 (0.02) [1.01;1.10] 0.010 | 1.03 (0.03) [0.97;1.09] 0.3 | 1.04 (0.04) [0.96;1.12] 0.3 |
| Atrial Fibrillation | ------------ | ------------ | 1.03 (0.09) [0.87;1.23] 0.722 | 1.00 (0.14) [0.77;1.31] 0.986 | ------------ | ------------ |
| Arthritis | 1.02 (0.09) [0.86;1.2] 0.833 | 1.02 (0.11) [0.83;1.25] 0.862 | ------------ | ------------ | ------------ | ------------ |
| Cancer | ------------ | ------------ | 1.04 (0.1) [0.87;1.24] 0.679 | 1.06 (0.16) [0.79;1.43] 0.688 | ------------ | ------------ |
| CVD | 0.92 (0.07) [0.78;1.08] 0.3 | 0.91 (0.1) [0.74;1.12] 0.374 | 0.98 (0.08) [0.83;1.16] 0.845 | 0.95 (0.14) [0.72;1.26] 0.741 | 1.39 (0.28) [0.94;2.06] 0.103 | 1.65 (0.48) [0.93;2.91] 0.085 |
| Liver disease | 0.8 (0.12) [0.59;1.08] 0.138 | 0.8 (0.16) [0.54;1.20] 0.286 | ------------ | ------------ | ------------ | ------------ |
| CHD | 1.01 (0.09) [0.85;1.2] 0.917 | 1.05 (0.12) [0.84;1.30] 0.688 | 0.94 (0.09) [0.78;1.12] 0.482 | 0.98 (0.14) [0.74;1.30] 0.891 | ------------ | ------------ |
| Epilepsy | ------------ | ------------ | 0.97 (0.15) [0.72;1.3] 0.842 | 0.78 (0.16) [0.53;1.16] 0.221 | ------------ | ------------ |
| Heart Failure | 1.03 (0.11) [0.83;1.27] 0.818 | 1.02 (0.14) [0.79;1.33] 0.878 | 0.92 (0.11) [0.73;1.15] 0.452 | 0.90 (0.17) [0.62;1.29] 0.558 | 0.83 (0.19) [0.53;1.3] 0.409 | 1.16 (0.42) [0.57;2.37] 0.687 |
| Multiple sclerosis | ------------ | ------------ | 0.4 (0.06) [0.29;0.54] 0 | 0.18 (0.07) [0.09;0.37] <0.001 | ------------ | ------------ |
| Parkinson's | 1.13 (0.15) [0.88;1.46] 0.333 | 1.00 (0.17) [0.72;1.39] 0.992 | 0.87 (0.14) [0.63;1.18] 0.365 | 0.68 (0.20) [0.39;1.20] 0.188 | ------------ | ------------ |
| Renal Failure | 1.03 (0.1) [0.85;1.24] 0.769 | 1.12 (0.14) [0.88;1.42] 0.354 | 0.9 (0.09) [0.75;1.09] 0.296 | 0.82 (0.13) [0.60;1.12] 0.203 | 1.2 (0.24) [0.81;1.78] 0.354 | 1.25 (0.35) [0.72;2.17] 0.435 |
| Diseases of blood | 0.93 (0.08) [0.79;1.11] 0.437 | 0.90 (0.1) [0.73;1.11] 0.337 | ------------ | ------------ | ------------ | ------------ |
| Diabetes | ------------ | ------------ | ------------ | ------------ | 0.85 (0.18) [0.55;1.3] 0.449 | 0.92 (0.26) [0.52;1.6] 0.756 |
| Constant | 469.5 (2319.98) [0.03;7547051] 0.213 | 22.71 (140.52) [0;4194325] 0.614 | 2796754 (19900000) [2.38;3290000000000] 0.037 | 40500000 (472000000) [0;329000000000000000] 0.132 | 2.82E+29 (5.36E+30) [18000000000000;4.43E+45] 0 | 3.34E+38 (9.1E+39) [2100000000000000;5.29E+61] 0.001 |

For peer review only

Results of the subgroup analysis including only patients with dementia (mortality risk)

| | site one (n=2,321) | site two (n=1,053) | site three (n=280) |
|---|---|---|---|
| | Mortality rate during follow-up coefficient (se) [95%CI] p value | Mortality rate during follow-up coefficient (se) [95%CI] p value | Mortality rate during follow-up coefficient (se) [95%CI] p value |
| HAH (hospital) | 1.05 (0.09) [0.89;1.24] 0.594 | 1.41 (0.12) [1.19;1.67] <0.001 | 1.65 (0.32) [1.12;2.41] 0.011 |
| Admission date | 1.00 (0.00) [1.00;1.00] 0.19 | 1.00 (0.00) [1.00;1.00] 0.001 | 1 (0) [1;1] 0.788 |
| ICD10 primary | 1.00 (0.00) [1.00;1.00] <0.001 | 1.00 (0.00) [1.00;1.00] 0.001 | 1 (0) [1;1] 0.14 |
| ICD10 secondary | 1.00 (0.00) [1.00;1.00] 0.207 | - - - - - - - - - - - - | 1 (0) [1;1] 0.979 |
| 2yrs pre AE costs | 1.00 (0.00) [1.00;1.00] 0.251 | 1.00 (0.00) [1.00;1.00] 0.609 | 1 (0) [1;1] 0.029 |
| 2yrs pre elective costs | 1.00 (0.00) [1.00;1.00] 0.735 | 1.00 (0.00) [1.00;1.00] 0.129 | 1 (0) [1;1] 0.554 |
| 2yrs pre non-elective costs | 1.00 (0.00) [1.00;1.00] 0.173 | 1.00 (0.00) [1.00;1.00] 0.484 | 1 (0) [1;1] 0.814 |
| 2yrs pre day case costs | 1.00 (0.00) [1.00;1.00] 0.088 | 1.00 (0.00) [1.00;1.00] 0.004 | 1 (0) [1;1] 0.896 |
| 2yrs pre geriatric ward costs | 1.00 (0.00) [1.00;1.00] 0.644 | 1.00 (0.00) [1.00;1.00] <0.001 | 1 (0) [1;1] 0.783 |
| 2yrs pre mental ward costs | 1.00 (0.00) [1.00;1.00] 0.569 | 1.00 (0.00) [1.00;1.00] 0.112 | 1 (0) [1;1] 0 |
| 2yrs pre outpatient costs | 1.00 (0.00) [1.00;1.00] 0.070 | 1.00 (0.00) [1.00;1.00] 0.167 | 1 (0) [1;1] 0 |
| 2yrs pre medication costs | 1.00 (0.00) [1.00;1.00] 0.004 | 1.00 (0.00) [1.00;1.00] 0.156 | 1 (0) [1;1] 0.011 |
| Died within 6months | - - - - - - - - - - - - | - - - - - - - - - - - - | - - - - - - - - - - - - |
| Number of LTCs | 0.94 (0.03) [0.88;1.01] 0.113 | 0.95 (0.03) [0.89;1.01] 0.115 | 0.98 (0.07) [0.86;1.13] 0.827 |
| Age on admission | 1.04 (0.01) [1.02;1.05] <0.001 | 1.03 (0.01) [1.01;1.04] <0.001 | 1.04 (0.02) [1;1.07] 0.024 |
| Male | 1.19 (0.11) [0.99;1.42] 0.063 | 1.17 (0.10) [0.99;1.38] 0.070 | 1.18 (0.25) [0.78;1.79] 0.43 |
| SES | 0.97 (0.02) [0.94;1.01] 0.134 | 1.00 (0.02) [0.97;1.03] 0.991 | 0.96 (0.04) [0.88;1.04] 0.3 |
| Atrial Fibrillation | - - - - - - - - - - - - | 1.03 (0.11) [0.85;1.26] 0.75 | - - - - - - - - - - - - |
| Arthritis | 1.06 (0.11) [0.86;1.30] 0.600 | - - - - - - - - - - - - | - - - - - - - - - - - - |
| Cancer | - - - - - - - - - - - - | 1.40 (0.13) [1.16;1.68] <0.001 | - - - - - - - - - - - - |
| CVD | 1.55 (0.41) [0.92;2.61] 0.099 | 1.14 (0.11) [0.94;1.39] 0.176 | 1.02 (0.25) [0.63;1.65] 0.925 |
| Liver disease | 0.98 (0.11) [0.79;1.21] 0.845 | - - - - - - - - - - - - | - - - - - - - - - - - - |
| CHD | 1.21 (0.16) [0.94;1.56] 0.135 | 0.99 (0.10) [0.81;1.20] 0.885 | - - - - - - - - - - - - |
| Epilepsy | - - - - - - - - - - - - | 1.26 (0.19) [0.94;1.70] 0.120 | - - - - - - - - - - - - |
| Heart Failure | 1.21 (0.16) [0.94;1.56] 0.135 | 1.33 (0.17) [1.04;1.70] 0.023 | 1.88 (0.49) [1.12;3.14] 0.017 |
| Multiple sclerosis | - - - - - - - - - - - - | 0.96 (0.51) [0.34;2.72] 0.932 | - - - - - - - - - - - - |
| Parkinson's | 1.26 (0.22) [0.9;1.78] 0.180 | 1.04 (0.20) [0.71;1.51] 0.848 | - - - - - - - - - - - - |
| Renal Failure | 1.06 (0.12) [0.84;1.32] 0.637 | 1.15 (0.12) [0.93;1.41] 0.192 | 0.56 (0.16) [0.32;0.97] 0.037 |
| Diseases of blood | 0.96 (0.11) [0.77;1.19] 0.709 | - - - - - - - - - - - - | - - - - - - - - - - - - |
| Diabetes | - - - - - - - - - - - - | - - - - - - - - - - - - | 0.6 (0.2) [0.32;1.15] 0.123 |
| Constant | 0.00 (0.00) [0.00;1.37] 0.057 | 0.00 (0.00) [0.00;0.00] <0.001 | 0 (0) [0;1810000000000000] 0.652 |

For peer review only

Results of the subgroup analysis excluding those who had died

| | site one (n=10,132) | | site two (n=3,584) | | site three (n=1691) | |
|---|---|---|---|---|---|---|
| | Follow-up period | Total costs in 6 months after discharge | Follow-up period | Total costs in 6 months after discharge | Follow-up period | Total costs in 6 months after discharge |
| | coefficient (se) [95%CI] p value | coefficient (se) [95%CI] p value | coefficient (se) [95%CI] p value | coefficient (se) [95%CI] p value | coefficient (se) [95%CI] p value | coefficient (se) [95%CI] p value |
| HAH (hospital) | 0.85 (0.04) [0.77;0.94] 0.002 | 1.23 (0.08) [1.08;1.4] 0.002 | 1.11 (0.06) [1;1.25] 0.058 | 1.17 (0.10) [0.99;1.38] 0.070 | 1.20 (0.11) [1;1.43] 0.046 | 1.71 (0.20) [1.36;2.15] <0.001 |
| Admission date | 1 (0) [1;1] 0.076 | 1.00 (0.00) [1.00;1.00] 0.032 | 1 (0) [1;1] 0.833 | 1.00 (0.00) [1.00;1.00] 0.337 | 1 (0) [1;1] 0.075 | 1 (0) [1;1] 0.282 |
| ICD10 primary | 1 (0) [1;1] 0.692 | 1.00 (0.00) [1.00;1.00] 0.993 | 1 (0) [1;1] 0.126 | 1.00 (0.00) [1.00;1.00] 0.038 | 1 (0) [1;1] 0.282 | 1 (0) [1;1] 0.279 |
| ICD10 secondary | 1 (0) [1;1] 0.817 | 1.00 (0.00) [1.00;1.00] 0.473 | 1 (0) [1;1] 0.014 | 1.00 (0.00) [1.00;1.00] 0.024 | 1 (0) [1;1] 0.724 | 1 (0) [1;1] 0.801 |
| 2yrs pre AE costs | 1 (0) [1;1] 0.08 | 1.00 (0.00) [1.00;1.00] 0.012 | 1 (0) [1;1] 0.461 | 1.00 (0.00) [1.00;1.00] 0.135 | 1 (0) [1;1] 0.435 | 1 (0) [1;1] 0.761 |
| 2yrs pre elective costs | 1 (0) [1;1] 0.015 | 1.00 (0.00) [1.00;1.00] 0.046 | 1 (0) [1;1] 0.576 | 1.00 (0.00) [1.00;1.00] 0.429 | 1 (0) [1;1] 0.63 | 1 (0) [1;1] 0.725 |
| 2yrs pre non-elective costs | 1 (0) [1;1] 0 | 1.00 (0.00) [1.00;1.00] <0.001 | 1 (0) [1;1] 0.651 | 1.00 (0.00) [1.00;1.00] 0.700 | 1 (0) [1;1] 0.199 | 1 (0) [1;1] 0.01 |
| 2yrs pre day case costs | 1 (0) [1;1] 0.416 | 1.00 (0.00) [1.00;1.00] 0.158 | 1 (0) [1;1] 0.057 | 1.00 (0.00) [1.00;1.00] 0.023 | 1 (0) [1;1] 0.068 | 1 (0) [1;1] 0.064 |
| 2yrs pre geriatric ward costs | 1 (0) [1;1] 0.031 | 1.00 (0.00) [1.00;1.00] 0.029 | 1 (0) [1;1] 0.625 | 1.00 (0.00) [1.00;1.00] 0.806 | 1 (0) [1;1] 0.484 | 1 (0) [1;1] 0.103 |
| 2yrs pre mental ward costs | 1 (0) [1;1] 0.206 | 1.00 (0.00) [1.00;1.00] 0.166 | 1 (0) [1;1] 0.009 | 1.00 (0.00) [1.00;1.00] 0.020 | 1 (0) [1;1] 0.01 | 1 (0) [1;1] 0.004 |
| 2yrs pre outpatient costs | 1 (0) [1;1] 0.236 | 1.00 (0.00) [1.00;1.00] 0.187 | 1 (0) [1;1] 0.748 | 1.00 (0.00) [1.00;1.00] 0.802 | 1 (0) [1;1] 0.798 | 1 (0) [1;1] 0.908 |
| 2yrs pre medication costs | 1 (0) [1;1] 0.399 | 1.00 (0.00) [1.00;1.00] 0.383 | 1 (0) [1;1] 0.011 | 1.00 (0.00) [1.00;1.00] 0.016 | 1 (0) [1;1] 0.37 | 1 (0) [1;1] 0.77 |
| Number of LTCs | 1.08 (0.02) [1.04;1.12] 0 | 1.12 (0.03) [1.07;1.18] <0.001 | 1.03 (0.02) [0.99;1.08] 0.169 | 1.06 (0.04) [0.99;1.13] 0.076 | 1.06 (0.03) [1.01;1.13] 0.032 | 1.09 (0.04) [1.01;1.17] 0.026 |
| Age on admission | 1.01 (0) [1;1.01] 0.025 | 1.01 (0.00) [1.00;1.02] 0.048 | 1.01 (0) [1;1.01] 0.054 | 1.01 (0.01) [1.00;1.02] 0.254 | 1.02 (0.01) [1;1.03] 0.019 | 1.01 (0.01) [0.99;1.03] 0.171 |
| Male | 1.11 (0.06) [1;1.22] 0.051 | 1.12 (0.07) [0.99;1.26] 0.085 | 0.94 (0.06) [0.83;1.07] 0.353 | 0.97 (0.09) [0.80;1.17] 0.752 | 0.97 (0.09) [0.8;1.16] 0.716 | 1 (0.12) [0.79;1.26] 0.974 |
| SES | 1 (0.01) [0.98;1.02] 0.965 | 1 (0.01) [0.98;1.03] 0.778 | 1.02 (0.01) [1;1.04] 0.081 | 1.03 (0.01) [1.00;1.06] 0.023 | 1 (0.02) [0.96;1.03] 0.822 | 1 (0.02) [0.95;1.05] 0.951 |
| Atrial Fibrillation | ------------ | ------------ | 1.07 (0.07) [0.94;1.21] 0.305 | 1.09 (0.10) [0.92;1.29] 0.335 | ------------ | ------------ |
| Arthritis | 0.99 (0.05) [0.89;1.1] 0.889 | 0.96 (0.06) [0.85;1.1] 0.584 | ------------ | ------------ | ------------ | ------------ |
| Cancer | ------------ | ------------ | 1 (0.07) [0.88;1.15] 0.961 | 1.01 (0.10) [0.84;1.23] 0.899 | ------------ | ------------ |
| CVD | 1.04 (0.07) [0.91;1.2] 0.552 | 1.00 (0.09) [0.85;1.19] 0.956 | 1.14 (0.08) [1;1.3] 0.058 | 1.14 (0.11) [0.95;1.36] 0.174 | 1.12 (0.14) [0.88;1.43] 0.367 | 1.1 (0.17) [0.81;1.5] 0.531 |
| Liver disease | 1.35 (0.2) [1.01;1.8] 0.045 | 1.31 (0.21) [0.95;1.81] 0.097 | ------------ | ------------ | ------------ | ------------ |
| Dementia | 1.16 (0.07) [1.04;1.3] 0.009 | 1.17 (0.08) [1.01;1.35] 0.033 | 1.08 (0.07) [0.96;1.22] 0.195 | 1.11 (0.10) [0.93;1.31] 0.244 | 1.37 (0.16) [1.09;1.73] 0.008 | 1.49 (0.23) [1.09;2.02] 0.011 |
| CHD | 0.82 (0.06) [0.72;0.94] 0.004 | 0.79 (0.07) [0.67;0.93] 0.004 | 1.01 (0.07) [0.87;1.16] 0.941 | 1.03 (0.10) [0.85;1.24] 0.799 | ------------ | ------------ |
| Epilepsy | ------------ | ------------ | 1.08 (0.12) [0.86;1.35] 0.518 | 1.09 (0.17) [0.80;1.48] 0.581 | ------------ | ------------ |
| Heart Failure | 1.1 (0.07) [0.97;1.25] 0.131 | 1.08 (0.08) [0.93;1.26] 0.293 | 1.08 (0.08) [0.94;1.24] 0.287 | 1.07 (0.11) [0.88;1.31] 0.491 | 1.05 (0.13) [0.82;1.34] 0.719 | 1.01 (0.16) [0.74;1.39] 0.932 |
| Multiple sclerosis | ------------ | ------------ | 0.72 (0.14) [0.49;1.06] 0.095 | 0.66 (0.21) [0.35;1.25] 0.202 | ------------ | ------------ |
| Parkinson's | 1.19 (0.1) [1;1.41] 0.05 | 1.15 (0.13) [0.93;1.43] 0.19 | 1.22 (0.18) [0.91;1.64] 0.193 | 1.34 (0.27) [0.91;1.98] 0.139 | ------------ | ------------ |
| Renal Failure | 1.01 (0.06) [0.89;1.14] 0.911 | 1.00 (0.07) [0.87;1.16] 0.949 | 1.06 (0.08) [0.92;1.22] 0.443 | 1.06 (0.11) [0.86;1.29] 0.602 | 1.12 (0.15) [0.86;1.46] 0.411 | 1.19 (0.2) [0.85;1.66] 0.317 |
| Diseases of blood | 1.04 (0.06) [0.94;1.16] 0.414 | 1.04 (0.07) [0.92;1.19] 0.516 | ------------ | ------------ | ------------ | ------------ |
| Diabetes | ------------ | ------------ | ------------ | ------------ | 1.33 (0.15) [1.07;1.65] 0.01 | 1.37 (0.19) [1.04;1.81] 0.026 |
| Constant | 3.67 (13.85) [0;5959] 0.73 | 0.07 (0.31) [0;592.13] 0.558 | 1064.79 (5943.4) [0.02;60000000] 0.212 | 0.89 (6.96) [0;4301665] 0.988 | 101000000000 (1050000000000) [149.57;68100000000000000000] 0.015 | 1320000000 (18000000000) [0;5.67E+20] 0.124 |



For peer review only - http://bmjopen.bmj.com/site/about/guidelines.xhtml

Results of the sensitivity analysis

| | site one (n=13,267) | | site two (n=4,769) | | site three (n=2110) | |
|---|---|---|---|---|---|---|
| | Total costs in follow-up (50% higher HAH unit costs) coefficient (se) [95%CI] p value | Total costs in follow-up (50% lower HAH unit costs) coefficient (se) [95%CI] p value | Total costs in follow-up (50% higher HAH unit costs) coefficient (se) [95%CI] p value | Total costs in follow-up (50% lower HAH unit costs) coefficient (se) [95%CI] p value | Total costs in follow-up (50% higher HAH unit costs) coefficient (se) [95%CI] p value | Total costs in follow-up (50% lower HAH unit costs) coefficient (se) [95%CI] p value |
| HAH (hospital) | 0.87 (0.03) [0.81;0.94] 0.001 | 0.77 (0.03) [0.71;0.84] 0 | 1.18 (0.05) [1.09;1.28] 0 | 0.81 (0.04) [0.74;0.9] 0 | 1.23 (0.09) [1.07;1.42] 0.004 | 1.07 (0.09) [0.91;1.25] 0.399 |
| Admission date | 1 (0) [1;1] 0.071 | 1 (0) [1;1] 0.048 | 1 (0) [1;1] 0.489 | 1 (0) [1;1] 0.3 | 1 (0) [1;1] 0.007 | 1 (0) [1;1] 0.012 |
| ICD10 primary | 1 (0) [1;1] 0.649 | 1 (0) [1;1] 0.671 | 1 (0) [1;1] 0.001 | 1 (0) [1;1] 0.001 | 1 (0) [1;1] 0.167 | 1 (0) [1;1] 0.16 |
| ICD10 secondary | 1 (0) [1;1] 0.588 | 1 (0) [1;1] 0.701 | 1 (0) [1;1] 0.148 | 1 (0) [1;1] 0.145 | 1 (0) [1;1] 0.875 | 1 (0) [1;1] 0.909 |
| 2yrs pre AE costs | 1 (0) [1;1] 0.223 | 1 (0) [1;1] 0.261 | 1 (0) [1;1] 0.687 | 1 (0) [1;1] 0.561 | 1 (0) [1;1] 0.307 | 1 (0) [1;1] 0.267 |
| 2yrs pre elective costs | 1 (0) [1;1] 0.909 | 1 (0) [1;1] 0.904 | 1 (0) [1;1] 0.537 | 1 (0) [1;1] 0.657 | 1 (0) [1;1] 0.896 | 1 (0) [1;1] 0.813 |
| 2yrs pre non-elective costs | 1 (0) [1;1] 0 | 1 (0) [1;1] 0 | 1 (0) [1;1] 0.919 | 1 (0) [1;1] 0.458 | 1 (0) [1;1] 0.015 | 1 (0) [1;1] 0.021 |
| 2yrs pre day case costs | 1 (0) [1;1] 0.099 | 1 (0) [1;1] 0.097 | 1 (0) [1;1] 0.006 | 1 (0) [1;1] 0.004 | 1 (0) [1;1] 0.131 | 1 (0) [1;1] 0.148 |
| 2yrs pre geriatric ward costs | 1 (0) [1;1] 0.006 | 1 (0) [1;1] 0.005 | 1 (0) [1;1] 0.002 | 1 (0) [1;1] 0 | 1 (0) [1;1] 0.562 | 1 (0) [1;1] 0.713 |
| 2yrs pre mental ward costs | 1 (0) [1;1] 0.905 | 1 (0) [1;1] 0.854 | 1 (0) [1;1] 0.005 | 1 (0) [1;1] 0.02 | 1 (0) [1;1] 0.09 | 1 (0) [1;1] 0.132 |
| 2yrs pre outpatient costs | 1 (0) [1;1] 0.086 | 1 (0) [1;1] 0.088 | 1 (0) [1;1] 0.027 | 1 (0) [1;1] 0.026 | 1 (0) [1;1] 0.699 | 1 (0) [1;1] 0.675 |
| 2yrs pre medication costs | 1 (0) [1;1] 0.713 | 1 (0) [1;1] 0.892 | 1 (0) [1;1] 0.136 | 1 (0) [1;1] 0.236 | 1 (0) [1;1] 0.713 | 1 (0) [1;1] 0.663 |
| Died within 6months | 1.03 (0.04) [0.95;1.11] 0.492 | 1.02 (0.04) [0.94;1.12] 0.572 | 1.05 (0.04) [0.97;1.14] 0.252 | 1.05 (0.05) [0.95;1.16] 0.38 | 1.06 (0.08) [0.91;1.23] 0.474 | 1.06 (0.09) [0.89;1.25] 0.517 |
| Number of LTCs | 1.08 (0.02) [1.05;1.11] 0 | 1.09 (0.02) [1.05;1.13] 0 | 1.04 (0.02) [1;1.07] 0.033 | 1.04 (0.02) [0.99;1.08] 0.093 | 1.06 (0.02) [1.01;1.1] 0.016 | 1.06 (0.03) [1.01;1.11] 0.019 |
| Age on admission | 1 (0) [1;1.01] 0.323 | 1 (0) [1;1.01] 0.452 | 1 (0) [1;1.01] 0.788 | 1 (0) [0.99;1.01] 0.789 | 1.01 (0.01) [1;1.02] 0.037 | 1.01 (0.01) [1;1.02] 0.055 |
| Male | 1.09 (0.04) [1.01;1.18] 0.035 | 1.1 (0.05) [1.01;1.2] 0.034 | 0.96 (0.04) [0.88;1.04] 0.311 | 0.95 (0.06) [0.85;1.07] 0.382 | 0.97 (0.07) [0.84;1.12] 0.686 | 0.97 (0.08) [0.82;1.14] 0.704 |
| SES | 1 (0.01) [0.98;1.02] 0.979 | 1 (0.01) [0.98;1.02] 0.954 | 1.01 (0.01) [1;1.02] 0.17 | 1.01 (0.01) [0.99;1.03] 0.205 | 1 (0.01) [0.97;1.03] 0.887 | 1 (0.02) [0.97;1.03] 0.917 |
| Atrial Fibrillation | - - - - - - - - - - - - | - - - - - - - - - - - - | 1.08 (0.05) [0.98;1.18] 0.104 | 1.1 (0.06) [0.98;1.23] 0.094 | - - - - - - - - - - - - | - - - - - - - - - - - - |
| Arthritis | 0.96 (0.04) [0.89;1.05] 0.392 | 0.96 (0.05) [0.88;1.05] 0.403 | - - - - - - - - - - - - | - - - - - - - - - - - - | - - - - - - - - - - - - | - - - - - - - - - - - - |
| Cancer | - - - - - - - - - - - - | - - - - - - - - - - - - | 1.04 (0.05) [0.95;1.14] 0.426 | 1.03 (0.06) [0.92;1.16] 0.566 | - - - - - - - - - - - - | - - - - - - - - - - - - |
| CVD | 1.02 (0.05) [0.92;1.13] 0.743 | 1.02 (0.06) [0.91;1.14] 0.794 | 1.07 (0.05) [0.98;1.18] 0.146 | 1.08 (0.07) [0.96;1.22] 0.199 | 1.09 (0.11) [0.91;1.32] 0.352 | 1.11 (0.12) [0.9;1.37] 0.324 |
| Liver disease | 1.21 (0.13) [0.98;1.48] 0.073 | 1.23 (0.14) [0.98;1.53] 0.074 | - - - - - - - - - - - - | - - - - - - - - - - - - | - - - - - - - - - - - - | - - - - - - - - - - - - |
| Dementia | 1.07 (0.05) [0.97;1.17] 0.16 | 1.07 (0.05) [0.97;1.18] 0.2 | 1.02 (0.05) [0.93;1.11] 0.738 | 0.99 (0.06) [0.88;1.1] 0.795 | 1.14 (0.11) [0.95;1.37] 0.153 | 1.14 (0.12) [0.94;1.4] 0.18 |
| CHD | 0.86 (0.05) [0.77;0.95] 0.004 | 0.85 (0.05) [0.76;0.95] 0.005 | 1.07 (0.05) [0.97;1.18] 0.174 | 1.02 (0.06) [0.9;1.15] 0.785 | - - - - - - - - - - - - | - - - - - - - - - - - - |
| Epilepsy | - - - - - - - - - - - - | - - - - - - - - - - - - | 1.04 (0.1) [0.86;1.26] 0.664 | 1.02 (0.12) [0.82;1.28] 0.841 | - - - - - - - - - - - - | - - - - - - - - - - - - |
| Heart Failure | 1.09 (0.05) [0.99;1.2] 0.095 | 1.09 (0.06) [0.98;1.21] 0.11 | 1.07 (0.06) [0.96;1.19] 0.201 | 1.09 (0.07) [0.96;1.24] 0.177 | 1.01 (0.1) [0.83;1.22] 0.947 | 1.02 (0.11) [0.82;1.25] 0.885 |
| Multiple sclerosis | - - - - - - - - - - - - | - - - - - - - - - - - - | 0.76 (0.1) [0.59;0.98] 0.033 | 0.73 (0.11) [0.54;0.99] 0.046 | - - - - - - - - - - - - | - - - - - - - - - - - - |
| Parkinson’s | 1.23 (0.11) [1.04;1.45] 0.018 | 1.24 (0.12) [1.03;1.49] 0.021 | 1.07 (0.14) [0.84;1.37] 0.582 | 1.11 (0.18) [0.81;1.52] 0.512 | - - - - - - - - - - - - | - - - - - - - - - - - - |
| Renal Failure | 1.04 (0.05) [0.95;1.13] 0.436 | 1.03 (0.05) [0.93;1.13] 0.601 | 1.04 (0.05) [0.94;1.15] 0.408 | 1.06 (0.07) [0.94;1.2] 0.366 | 1.11 (0.11) [0.91;1.36] 0.3 | 1.12 (0.13) [0.9;1.39] 0.317 |
| Diseases of blood | 1.05 (0.05) [0.97;1.14] 0.246 | 1.05 (0.05) [0.96;1.15] 0.308 | - - - - - - - - - - - - | - - - - - - - - - - - - | - - - - - - - - - - - - | - - - - - - - - - - - - |
| Diabetes | - - - - - - - - - - - - | - - - - - - - - - - - - | - - - - - - - - - - - - | - - - - - - - - - - - - | 1.2 (0.11) [1;1.42] 0.044 | 1.22 (0.12) [1.01;1.48] 0.042 |
| Constant | 26.62 (74.48) [0.11;6410.63] 0.241 | 8.84 (27.52) [0.02;3945.99] 0.484 | 295178.8 (1199605) [102.52;850000000] 0.002 | 1223534 (6192074) [60.23;24900000000] 0.006 | 14800000000000 (127000000000000) [776224.7;2.84E+20] 0 | 31000000000(2920000000) [292677.5;3.28E+21] 0.001 |



For peer review only - http://bmjopen.bmj.com/site/about/guidelines.xhtml

Appendix 4 Estimated E-values

**Site one**

Total costs during follow-up period E-value for point estimate: 1.73 and for confidence interval: 1.49;

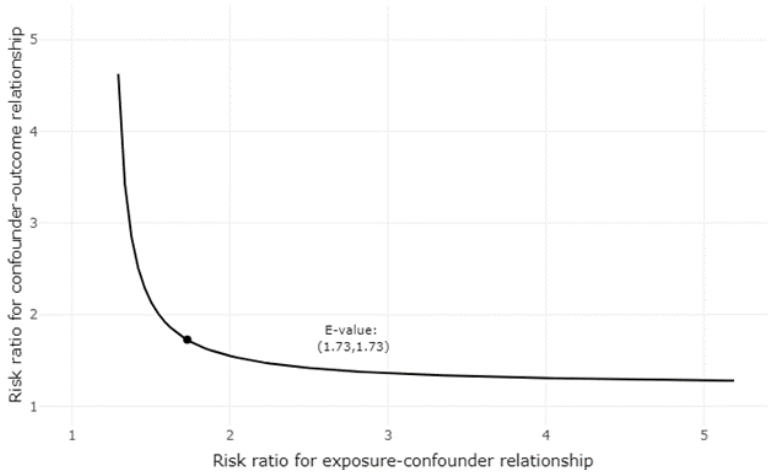


Total costs in 6 months after discharge E-value for point estimate: 1.86 and for confidence interval: 1.55;

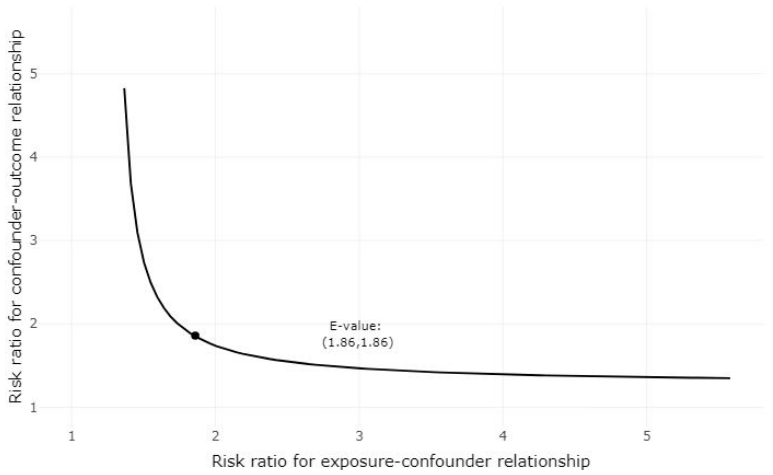




For peer review only - http://bmjopen.bmj.com/site/about/guidelines.xhtml

Mortality rate during follow-up: E-value for point estimate: 1.42 and for confidence interval: 1.04;

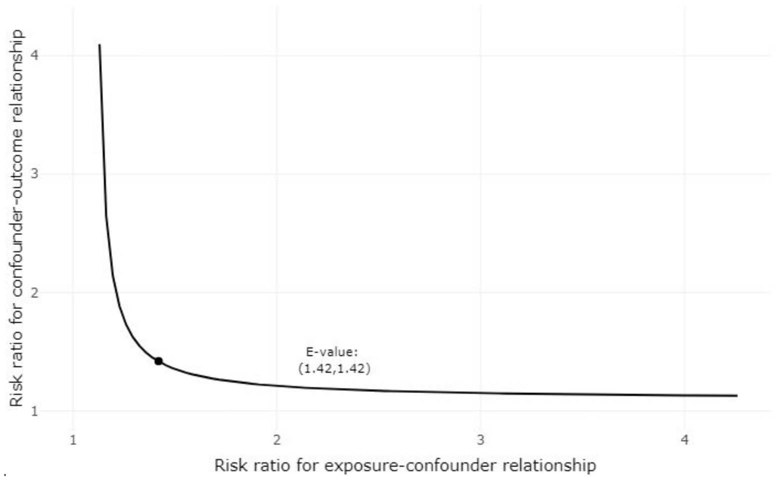


**Site two**

Total costs during follow-up period: E-value for point estimate: 1.03 and for confidence interval: 1

For peer review only

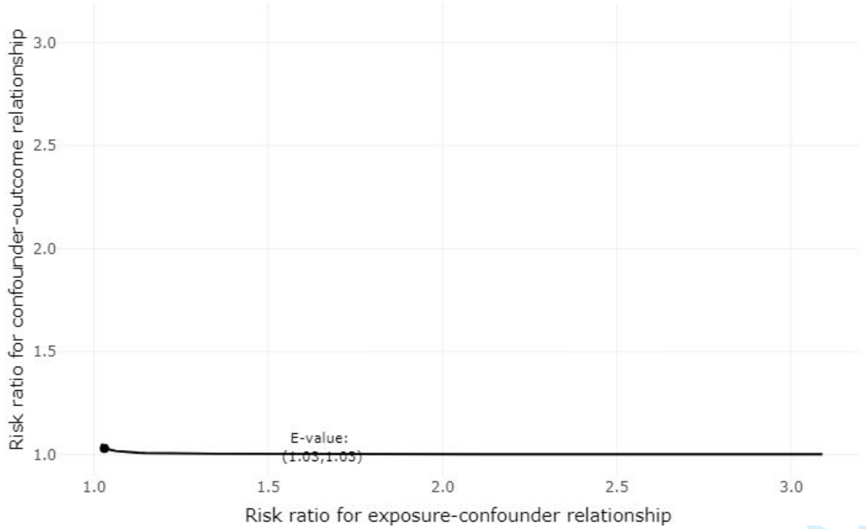


Total costs in 6 months after discharge: E-value for point estimate: 1.4 and for confidence interval: 1

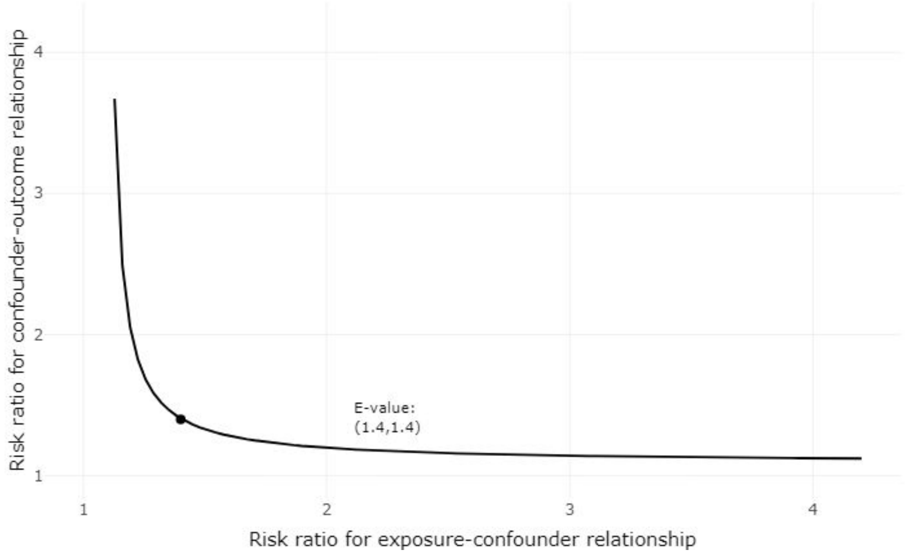


Mortality rate during follow-up: E-value for point estimate: 1.9 and for confidence interval: 1.57


For peer review only - http://bmjopen.bmj.com/site/about/guidelines.xhtml

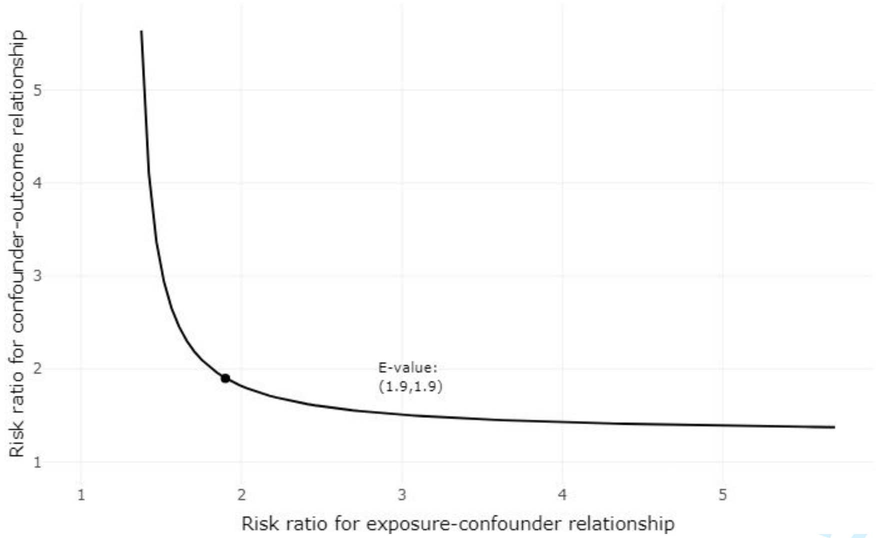


**Site three**

Total costs during follow-up period: E-value for point estimate: 1.57 and for confidence interval: 1

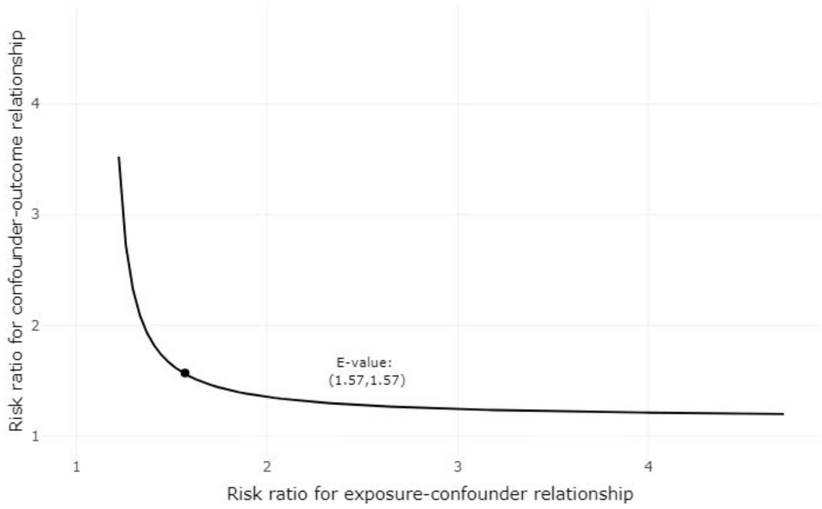



For peer review only - http://bmjopen.bmj.com/site/about/guidelines.xhtml

Total costs in 6 months after discharge: E-value for point estimate: 2.79 and for confidence interval: 2.15

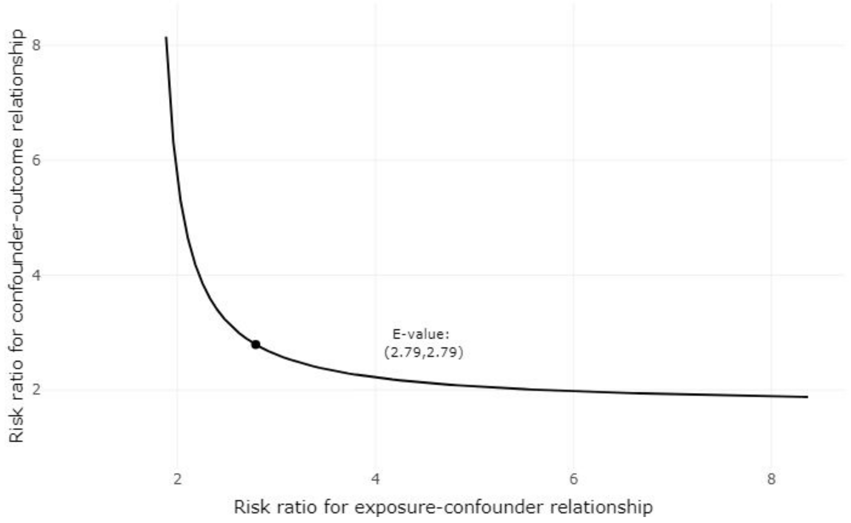


Mortality rate during follow-up: E-value for point estimate: 1.86 and for confidence interval: 1.31

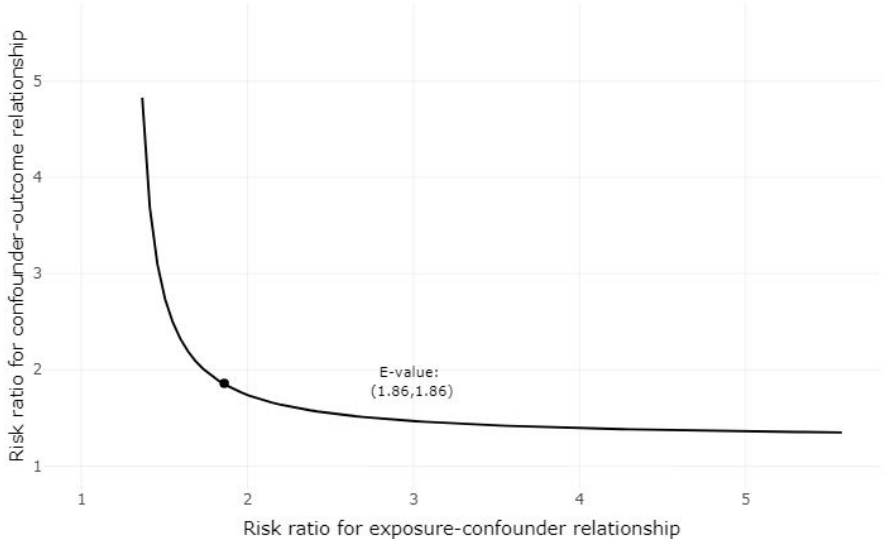



For peer review only - http://bmjopen.bmj.com/site/about/guidelines.xhtml

**The RECORD statement – checklist of items, extended from the STROBE statement, that should be reported in observational studies using routinely collected health data.**

| | Item No. | STROBE items | Location in manuscript where items are reported | RECORD items | Location in manuscript where items are reported |
|---|---|---|---|---|---|
| **Title and abstract** | | | | | |
| | 1 | (a) Indicate the study's design with a commonly used term in the title or the abstract (b) Provide in the abstract an informative and balanced summary of what was done and what was found | 1a: page 1<br><br>1b: page 2 | RECORD 1.1: The type of data used should be specified in the title or abstract. When possible, the name of the databases used should be included.<br><br>RECORD 1.2: If applicable, the geographic region and timeframe within which the study took place should be reported in the title or abstract.<br><br>RECORD 1.3: If linkage between databases was conducted for the study, this should be clearly stated in the title or abstract. | 1.1: page 1<br><br>1.2: page 2<br><br>1.3: page 2 |
| **Introduction** | | | | | |
| Background rationale | 2 | Explain the scientific background and rationale for the investigation being reported | page 4 | | |
| Objectives | 3 | State specific objectives, including any prespecified hypotheses | page 4 | | |
| **Methods** | | | | | |
| Study Design | 4 | Present key elements of study | page 5 | | |

For peer review only

| | | | | | |
|---|---|---|---|---|---|
| | | design early in the paper | | | |
| Setting | 5 | Describe the setting, locations, and relevant dates, including periods of recruitment, exposure, follow-up, and data collection | page 5-6 | | |
| Participants | 6 | *(a) Cohort study* - Give the eligibility criteria, and the sources and methods of selection of participants. Describe methods of follow-up<br>*Case-control study* - Give the eligibility criteria, and the sources and methods of case ascertainment and control selection. Give the rationale for the choice of cases and controls<br>*Cross-sectional study* - Give the eligibility criteria, and the sources and methods of selection of participants<br><br>*(b) Cohort study* - For matched studies, give matching criteria and number of exposed and unexposed<br>*Case-control study* - For matched studies, give matching criteria and the number of controls per case | 6a: NA<br><br>6b: page 6-8 | RECORD 6.1: The methods of study population selection (such as codes or algorithms used to identify subjects) should be listed in detail. If this is not possible, an explanation should be provided.<br><br>RECORD 6.2: Any validation studies of the codes or algorithms used to select the population should be referenced. If validation was conducted for this study and not published elsewhere, detailed methods and results should be provided.<br><br>RECORD 6.3: If the study involved linkage of databases, consider use of a flow diagram or other graphical display to demonstrate the data linkage process, including the number of individuals with linked data at each stage. | 6.1: page 5-6<br><br>6.2 NA<br><br>6.3 NA |
| Variables | 7 | Clearly define all outcomes, exposures, predictors, potential confounders, and effect | page 7-8 | RECORD 7.1: A complete list of codes and algorithms used to classify exposures, outcomes, confounders, and | page 5, 7-8 |

| | | | | | |
|---|---|---|---|---|---|
| | | modifiers. Give diagnostic criteria, if applicable. | | effect modifiers should be provided. If these cannot be reported, an explanation should be provided. | |
| Data sources/ measurement | 8 | For each variable of interest, give sources of data and details of methods of assessment (measurement).<br>Describe comparability of assessment methods if there is more than one group | page 6 | | |
| Bias | 9 | Describe any efforts to address potential sources of bias | page 6-8 | | |
| Study size | 10 | Explain how the study size was arrived at | page 6-8 | | |
| Quantitative variables | 11 | Explain how quantitative variables were handled in the analyses. If applicable, describe which groupings were chosen, and why | page 7-8 | | |
| Statistical methods | 12 | (a) Describe all statistical methods, including those used to control for confounding<br>(b) Describe any methods used to examine subgroups and interactions<br>(c) Explain how missing data were addressed<br>(d) *Cohort study* - If applicable, explain how loss to follow-up was addressed<br>*Case-control study* - If applicable, explain how | 12a: page 7-8<br>12b: page 8<br>12c: page 8<br>12d: 6-8 | | |

For peer review only

| | | | | | |
|---|---|---|---|---|---|
| | | matching of cases and controls was addressed<br>*Cross-sectional study* - If applicable, describe analytical methods taking account of sampling strategy<br>(e) Describe any sensitivity analyses | 12e: page 8 | | |
| Data access and cleaning methods | | .. | | RECORD 12.1: Authors should describe the extent to which the investigators had access to the database population used to create the study population.<br><br>RECORD 12.2: Authors should provide information on the data cleaning methods used in the study. | 12.1: page 5<br><br>12.2: page 6 |
| Linkage | | .. | | RECORD 12.3: State whether the study included person-level, institutional-level, or other data linkage across two or more databases. The methods of linkage and methods of linkage quality evaluation should be provided. | page 6 |
| **Results** | | | | | |
| Participants | 13 | (a) Report the numbers of individuals at each stage of the study (*e.g.*, numbers potentially eligible, examined for eligibility, confirmed eligible, included in the study, completing follow-up, and analysed) | 13a: page 9-10 | RECORD 13.1: Describe in detail the selection of the persons included in the study (*i.e.,* study population selection) including filtering based on data quality, data availability and linkage. The selection of included persons can be described in the text and/or by | page 6, 8, 10 |

For peer review only

| | | | | | |
|---|---|---|---|---|---|
| | | (b) Give reasons for non-participation at each stage.<br>(c) Consider use of a flow diagram | 13b: page 9-10<br><br>13c: page 10 | means of the study flow diagram. | |
| Descriptive data | 14 | (a) Give characteristics of study participants (*e.g.*, demographic, clinical, social) and information on exposures and potential confounders<br>(b) Indicate the number of participants with missing data for each variable of interest<br>(c) *Cohort study* - summarise follow-up time (*e.g.*, average and total amount) | 14a: page 9,12<br><br>14b: NA<br><br>14c: page 6,13 | | |
| Outcome data | 15 | *Cohort study* - Report numbers of outcome events or summary measures over time<br>*Case-control study* - Report numbers in each exposure category, or summary measures of exposure<br>*Cross-sectional study* - Report numbers of outcome events or summary measures | page 13 | | |
| Main results | 16 | (a) Give unadjusted estimates and, if applicable, confounder-adjusted estimates and their precision (e.g., 95% confidence interval). Make clear which confounders were adjusted for and why they were included | 16a: page 13, 15 | | |

For peer review only

| | | | | | |
|---|---|---|---|---|---|
| | | (b) Report category boundaries when continuous variables were categorized<br>(c) If relevant, consider translating estimates of relative risk into absolute risk for a meaningful time period | 16b: NA<br><br>16c: NA | | |
| Other analyses | 17 | Report other analyses done—e.g., analyses of subgroups and interactions, and sensitivity analyses | page 15 | | |
| **Discussion** | | | | | |
| Key results | 18 | Summarise key results with reference to study objectives | page 15 | | |
| Limitations | 19 | Discuss limitations of the study, taking into account sources of potential bias or imprecision. Discuss both direction and magnitude of any potential bias | page 17 | RECORD 19.1: Discuss the implications of using data that were not created or collected to answer the specific research question(s). Include discussion of misclassification bias, unmeasured confounding, missing data, and changing eligibility over time, as they pertain to the study being reported. | page 17 |
| Interpretation | 20 | Give a cautious overall interpretation of results considering objectives, limitations, multiplicity of analyses, results from similar studies, and other relevant evidence | page 18-19 | | |
| Generalisability | 21 | Discuss the generalisability (external validity) of the study | page 15-16 | | |

For peer review only

| | | | | | |
|---|---|---|---|---|---|
| | | results | | | |
| **Other Information** | | | | | |
| Funding | 22 | Give the source of funding and the role of the funders for the present study and, if applicable, for the original study on which the present article is based | page 19 | | |
| Accessibility of protocol, raw data, and programming code | | .. | | RECORD 22.1: Authors should provide information on how to access any supplemental information such as the study protocol, raw data, or programming code. | page 19 |

*Reference: Benchimol EI, Smeeth L, Guttmann A, Harron K, Moher D, Petersen I, Sørensen HT, von Elm E, Langan SM, the RECORD Working Committee. The REporting of studies Conducted using Observational Routinely-collected health Data (RECORD) Statement. *PLoS Medicine* 2015; in press.

*Checklist is protected under Creative Commons Attribution (CC BY) license.

For peer review only
